# Synthesis and Biological Evaluation of Novel Dehydroabietic Acid-Oxazolidinone Hybrids for Antitumor Properties

**DOI:** 10.3390/ijms19103116

**Published:** 2018-10-11

**Authors:** Xiu Wang, Fu-Hua Pang, Lin Huang, Xin-Ping Yang, Xian-Li Ma, Cai-Na Jiang, Fang-Yao Li, Fu-Hou Lei

**Affiliations:** 1 College of Pharmacy, Guilin Medical University, 109 North 2nd Huancheng Road, Guilin 541004, China; wangxiumt2015@163.com (X.W.); pangfuhua1994@iCloud.com (F.-H.P.); 14795985531@163.com (L.H.); yxinpkin@163.com (X.-P.Y.); mxl78@glmc.edu.cn (X.-L.M.); fyli2006@glmc.edu.cn (C.-N.J.); 2 Guangxi Key Laboratory of Chemistry and Engineering of Forest Products, Nanning 530006, China; leifuhou@gmail.com

**Keywords:** dehydroabietic acid, oxazolidinone, antitumor activity, cell cycle, apoptosis

## Abstract

Novel representatives of the important group of biologically-active, dehydroabietic acid-bearing oxazolidinone moiety were synthesized to explore more efficacious and less toxic antitumor agents. Structures of all the newly target molecules were confirmed by IR, ^1^H-NMR, ^13^C-NMR, and HR-MS. The inhibitory activities of these compounds against different human cancer cell lines (MGC-803, CNE-2, SK-OV-3, NCI-H460) and human normal liver cell line LO2 were evaluated and compared with the commercial anticancer drug cisplatin, using standard MTT (methyl thiazolytetrazolium) assay in vitro. The pharmacological screening results revealed that most of the hybrids showed significantly improved antiproliferative activities over dehydroabietic acid and that some displayed better inhibitory activities compared to cisplatin. In particular, compound **4j** exhibited promising cytotoxicity with IC_50_ values ranging from 3.82 to 17.76 µM against all the test cell lines and displayed very weak cytotoxicity (IC_50_ > 100 µM) on normal cells, showing good selectivity between normal and malignant cells. Furthermore, the action mechanism of the representative compound **4j** was preliminarily investigated by Annexin-V/PI dual staining, Hoechst 33258 staining, which indicated that the compound can induce cell apoptosis in MGC-803 cells in a dose-dependent manner and arrest the cell cycle in G1 phase. Therefore, **4j** may be further exploited as a novel pharmacophore model for the development of anticancer agents.

## 1. Introduction

Cancer is a multifaceted and multi-mechanistic disease characterized by the uncontrolled growth and spread of abnormal cells [1]. Nowadays, represents one of the most common life-threatening diseases with rising incidence and mortality [2,3]. The development of the prevention and treatment of cancer has faced the problem of adverse effects and the developing resistance of cancer cells to drugs, which have made many chemotherapeutic regimens ineffective. Hence, there is an urgent need to search for novel anticancer drugs and therapies with more efficacy, less side effects, and a broader spectrum.

Natural products have been the most productive source of leads for drug discovery. Among them, terpenoids exhibit extensive potential as pharmaceutical products for the treatment of different diseases. Dehydroabietic acid (DHAA) is a naturally occurring tricyclic diterpenic resin acid and can be easily isolated from *pinus* rosin or disproportionate rosin. DHAA and its derivatives have been reported to have a wide range of biological activities, such as antibacterial [4,5], antiviral [6], insecticidal [7], antifungal [8], anti-aging [9], antiprotozoal [10] and anti-inflammatory activity [11]. In particular, previous research has shown that a large number of DHAA derivatives possess a variety of anticancer activities in many human cancers, and could act at various stages of tumor development to inhibit tumor cell proliferation, as well as to induce tumor cell apoptosis [12,13,14,15]. These results suggest that DHAA is a promising starting material for novel potential antitumor agents.

Currently, in the field of medicinal chemistry, five membered ring heterocyclic compounds are receiving special attention. The oxazolidinone scaffold was considered an important pharmacophore existing in numerous compounds to elicit diverse pharmacological properties, including antibacterial [16,17,18], antifungal [19], antidiabetic [20], and anticonvulsant [21] properties. Recently, oxazolidinones were developed as a new class of antibacterial drug against MRSA (methicillin-resistant staphylococcus aureus), and VRE (vancomycin-resistant enterococcus) [22]. Furthermore, it has been reported that some of the oxazolidinones showed anticancer activity and were in early clinical trials [23,24,25,26,27,28,29]. The aforementioned findings indicate that the introduction of an oxazolidinone moiety to the skeleton of DHAA may produce novel derivatives with significant anticancer properties.

Molecular hybridization is a potent strategy in drug design and development to obtain new hybrid compounds with improved affinity and efficacy compared to the parent drugs. In previous work, we found that hybrid molecules of DHAA with various acylhydrazone moieties exhibited remarkable anticancer activity [30]. In continuation of our interest in searching for terpenoid derivatives with anticancer pharmacological effects, we have designed and synthesized a series of new hybrid molecules containing DHAA and the oxazolidinone scaffold. Their antiproliferative activities in vitro against four tested tumor cell lines were evaluated. The results showed that the intermediates and target compounds inhibited the proliferation of these four tumor cell lines at moderate to high levels. Moreover, our results clearly demonstrated that compound **4j** can induce apoptosis in MGC-803 cells in a dose-dependent manner and arrested the cell cycle in G1 phase.

## 2. Results

### 2.1. Chemistry

**Scheme 1:**
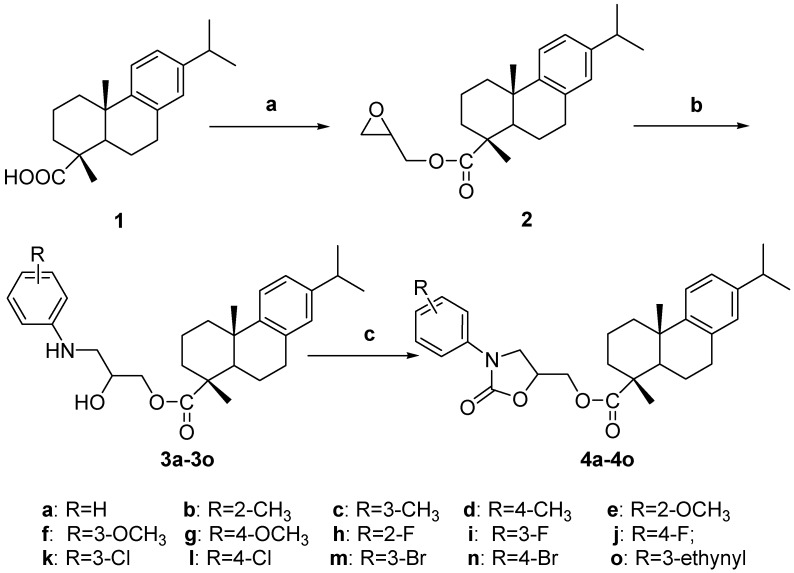
Synthetic pathways for compounds **3a**–**o** and **4a**–**o**. Reagents and conditions: (**a**) epibromohydrin, acetone, 60 °C reflux 4 h; (**b**) aromatic primary amines, Zn(ClO_4_)_2_·6H_2_O, EtOH, 80 °C, reflux 1 h; (**c**) BTC (bis(trichloromethyl)carbonate), NaOH, THF (tetrahydrofuran), DCM, 2 h.

The synthetic route for the target DHAA-oxazolidinone hybrids is shown in Scheme 1. Briefly, the key intermediate epoxide **2** was prepared in good yield by the reaction of the parent compound DHAA **1** with epibromohydrin in the presence of anhydrous K_2_CO_3_ and acetone according to the literature [31]. The epoxide **2** was then ring-opened with the proper aromatic amines in absolute ethanol to afford the corresponding amino alcohols **3** [32]. The cyclization of compound **3** with BTC (bis(trichloromethyl)carbonate) yielded the final product **4** in the presence of NaOH and THF (tetrahydrofuran) in an ice bath [33]. The structures of all the derivatives were then confirmed by IR, ^1^H-NMR, ^13^C-NMR and high resolution mass spectrometry (HR-MS).

### 2.2. Biological Assays

#### 2.2.1. Cytotoxicity Measurement

The in vitro antiproliferative activity of the DHAA derivatives **3a**–**o** and **4a**–**o** against four human cancer cell lines (MGC-803 human gastric cancer cell line, CNE-2 human nasopharyngeal carcinoma cell line, SK-OV-3 human ovarian carcinoma cell line, NCI-H460 human lung cancer cell line) and the human normal liver cell line LO2 were evaluated by MTT (methyl thiazolytetrazolium) assay and compared with the parent compound DHAA and the positive control cisplatin in each panel. The results are shown in Table 1.

It was found that most of the target compounds exhibited inhibitory activity against the tested tumor cell lines, indicating that the introduction of oxazolidinone and the amino alcohol moiety on the DHAA skeleton markedly increased antitumor activity. In addition, the activity of the oxazolidinone derivative **4** was better than those of the amino alcohol derivative **3**. In particular, compounds **4g** and **4j** displayed preferable cytotoxic activity against the four cancer cells than the positive control cisplatin. In this regard, compound **4j** with a F moiety at para-positions of the benzene ring showing the most potent inhibitory activity against four of the tested cells, with IC_50_ values of 3.82 ± 0.18, 17.76 ± 0.69, 4.66 ± 2.13 and 8.44 ± 0.36 µM against the MGC-803, CNE-2, SK-OV-3 and NCI-H460 cancer cells, respectively. Compounds **4d**, **4g** and **4j** displayed 2.5 to 8.2 times more potent cytotoxicity than the positive control cisplatin against certain cancer cell lines. Notably, all the compounds showed lower cytotoxicity on non-tumor live LO2 cells than on these four cancer cell lines. Overall, the inhibitory effect of compounds on gastric and lung cancer cells was better than on nasopharyngeal and ovarian carcinoma cells. All of these results indicated that the target compounds had selective inhibitory effects on tumor cells.

From the above results, some interesting structure–activity relationships could be concluded: The introduction of amino-alcohol and oxazolidinone was significant for improving their activity. Comparing the antitumor activity of compounds **3** and **4**, it was found that the antitumor activity of compound **4** was better than that of **3**. Different substitutions and positions of the aryl group affected the cytotoxicity of target compounds. The activity of the compounds **3** with -CH_3_, -OCH_3_ and -Cl groups at the three-position seemed to be slightly better than that of substituents at other positions, while the introduction of the -F and -Br groups at the four-position was better than other positions. Compared to **3i** and **3j**, the introduction of fluorine atoms at the four-position enhanced the inhibitory activity against all of tested cell lines, while the presence of fluorine atoms on the three-position led to the activity being eliminated. In the MGC-803 and CNE-2 assays, the antitumor activity of compound **4** was found to be in the order of para > meta. It is clear that compound **4** with electron-donating groups exhibited better inhibitory activity against NCI-H460 than the presence of electron withdrawing groups.

#### 2.2.2. Cell Cycle Analysis

**Figure 1:**
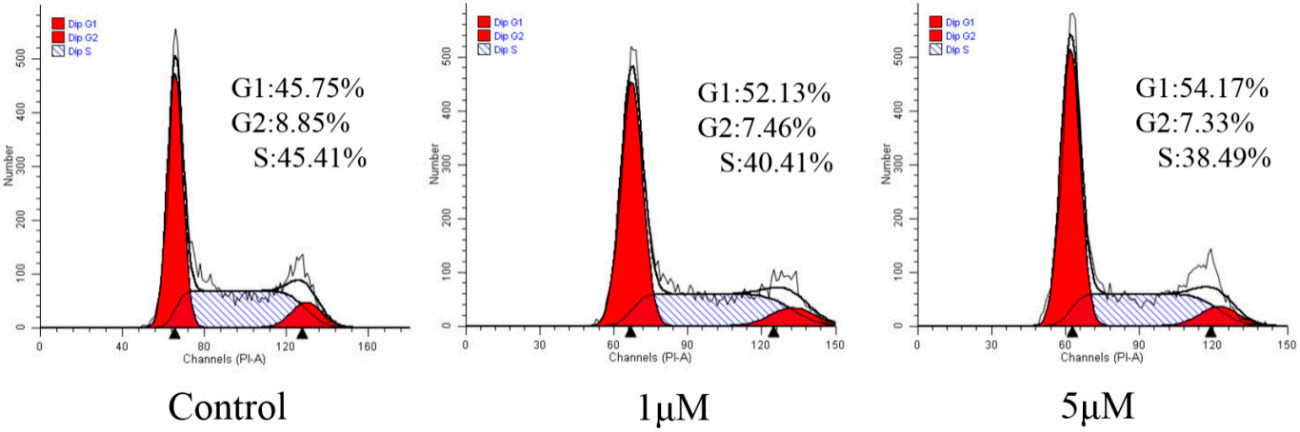
Effect of compound **4j** on the cell cycle of human gastric MGC-803 cells.

To determine the possible role of cell cycle arrest in **4j**-induced growth inhibition, MGC-803 cells were treated with different concentrations of compound **4j** for 48 h. The cell cycle distribution was investigated by flow cytometric analysis following the staining of DNA with propidium iodide (PI). It was observed that the S phase cells gradually decreased and the G2 phase cells did not change significantly, while the G1 phase cells compared with the control cells gradually increased, respectively (Figure 1). These results suggested that the target compound **4j** mainly arrested the MGC-803 cells in the G1 phase.

#### 2.2.3. Compound **4j** Induces Apoptosis in MGC-803 Cells

**Figure 2:**
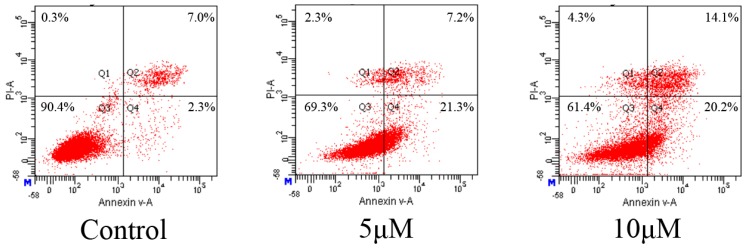
Apoptosis ratio detection of **4j** by flow cytometry. Cells were treated with compound **4j** for 48 h.

In order to further confirm whether the **4j**-induced reduction in cell viability was responsible for the induction of apoptosis, MGC-803 cells were investigated using the PI and Annexin-V/FITC (fluorescein isothiocyanate) and the number of apoptotic cells was estimated by flow cytometry, after the treatment of the MGC-803 cells at concentrations of 5 and 10 µM for 48 h. The results are illustrated in Figure 2. The percentage of all apoptotic cells (early and late) (9.3%) was present in the control panel, in contrast, the percentage increased from 28.5% (5 µM) to 34.3% (10 µM). These results clearly confirmed that compound **4j** effectively induced apoptosis in MGC-803 cells in a dose-dependent manner.

#### 2.2.4. Hoechst 33258 Staining Assay

**Figure 3:**
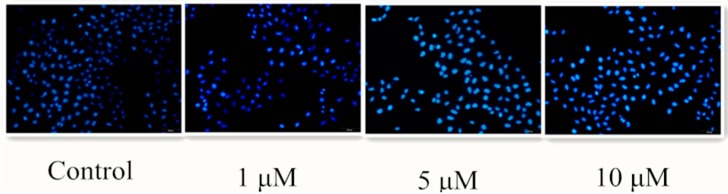
Effect of compound **4j** on the nuclear morphological changes of MGC-803 cells. Images were acquired using an OLYMPUS 1X73 fluorescence microscope (magnification 200×).

Hoechst 33258 is a dye that is utilized to identify the transitions of a cell’s nuclear morphology [34]. To determine the morphological changes induced by compound **4j** in MGC-803 cells, Hoechst 33258 staining was carried out. MGC-803 cells were treated with various concentrations (0, 1.0, 5.0, 10 µM) of compound **4j** for 48 h, and stained with Hoechst 33258. The results showed that most of the cells exhibited the weak blue fluorescence in the control cells. In the 1.0 and 5.0 µM groups, some exhibited bright-blue fluorescence due to chromatin condensation. The number of apoptotic nuclei significantly increased along with the increase in the concentration of **4j** to 10.0 µM (Figure 3).

## 3. Materials and Methods

### 3.1. Chemistry

All the chemical reagents and solvents obtained were of analytical grade. Routine thin-layer chromatography (TLC) was performed on silica gel plates (silica gel GF254 from Qingdao Haiyang Chemical Co., Ltd., Qingdao, China). Preparative flash column chromatography was performed on the 200–300 mesh silica gel (Qingdao Haiyang Chemical Co. Ltd.). The melting points were recorded on an X-4 microscope melting point apparatus (Beijing Tech Instrument, Beijing, China) without calibration. FT-IR spectra were carried out using a Prestige-21 FT-IR spectrometer (Shimazu, Kyoto, Japan). NMR spectra were recorded on a BRUKER AV-600/400 spectrometer (Bruker, Rheinstetten, Germany) with TMS (tetramethylsilane) as an internal standard in (dimethyl sulfoxide) DMSO-*d_6_*. Mass spectra were determined on an FTMS ESI spectrometer (Thermo, Waltham, MA, USA).

### 3.2. Synthesis: General Procedure for Compounds **3a**–**o**

Dehydroabietic acid (29.95 mmol) and dry K_2_CO_3_ (1.39 mmol) were added to acetone (40 mL) in a round bottom flask with magnetic stirring and stirred at room temperature. After that, epibromohydrin (32.5 mmol) was dripped into the mixture and the mixture was refluxed at 60 °C for 4 h. After cooling to room temperature and being washed with ethyl acetate, the solvent was evaporated under reduced pressure. The crude product was purified by chromatography on silica gel eluted with petroleum ether/ethylacetate (*v*:*v* = 10:1) to afford glycidyl dehydroabiate **2**. Compound **2** (0.34 mmol), aromatic primary amines (0.41 mmol) and Zn(ClO_4_)_2_·6H_2_O (5 mg) as a catalyst were added to absolute ethanol (10 mL) and the mixture was refluxed at 80 °C for 1 h. After the reaction was completed, the solvent was evaporated under reduced pressure, and the crude product was purified by chromatography on silica gel eluted with petroleum ether/ethyl acetate (*v*:*v* = 10: 1) to obtain compounds **3a**–**o**. The structures were confirmed by ^1^H-NMR, ^13^C-NMR and HR-MS (see supplementary materials).

#### 3.2.1. (1R,4aS)-2-Hydroxy-3-(Phenylamino)Propyl-7-Isopropyl-1,4a-Dimethyl-1,2,3,4,4a,9,10,10a-Octahydrophenanthrene-1-Carboxylate (**3a**)

Yield 74.4%, as a white solid. Mp: 74.6–78.4 °C. ^1^H-NMR (600 MHz, DMSO-*d_6_*) *δ*: 7.15 (s, 1H), 6.97 (d, *J* = 7.4 Hz, 3H), 6.81 (s, 1H), 6.56 (d, *J* = 3.9 Hz, 2H), 6.50 (s, 1H), 5.52 (s, 1H), 5.13 (s, 1H), 4.07 (s, 1H), 3.97 (s, 1H), 3.84 (s, 1H), 3.11 (s, 1H), 3.00 (s, 1H), 2.80–2.72 (m, 3H), 2.50 (s, 1H), 2.28 (s, 1H), 2.12 (s, 1H), 1.72 (d, *J* = 9.3 Hz, 3H), 1.64 (s, 1H), 1.58 (s, 1H), 1.32 (s, 1H), 1.21 (s, 3H), 1.15 (d, *J* = 3.7 Hz, 3H), 1.14 (d, *J* = 3.7 Hz, 3H), 1.13 (s, 3H).^13^C-NMR (150 MHz, DMSO-*d_6_*) *δ*: 177.6, 148.7, 146.7, 145.1, 134.2, 128.9, 126.5, 124.28, 123.8, 115.8, 112.1, 67.0, 66.6, 47.2, 46.4, 44.8, 37.7, 36.6, 36.2, 33.0, 29.6, 25.0, 24.0, 21.2, 18.2, 16.4. IR (KBr), *ν*/cm^−1^: 3377, 2951, 1701, 1606, 1500, 1463, 1384. HR-MS-ESI (*m*/*z*): calcd for C_29_H_39_NO_3_ [M+H]^+^: 450.3010; found: 450.3009.

#### 3.2.2. (1R,4aS)-2-Hydroxy-3-(2-Methylphenylamino)Propyl-7-Isopropyl-1,4a-Dimethyl-1,2,3,4,4a,9,10, 10a-Octahydrophenanthrene-1-Carboxylate (**3b**)

Yield 80.5%, as a white solid. Mp: 92.7–95.9 °C. ^1^H-NMR (600 MHz, DMSO-*d_6_*) *δ*: 7.14 (s, 1H), 7.02 –6.90 (m, 3H), 6.80 (s, 1H), 6.47 (d, *J* = 6.9 Hz, 2H), 5.16 (s, 1H), 4.68 (s, 1H), 4.08 (s, 1H), 3.99 (s, 1H), 3.88 (s, 1H), 3.16 (s, 1H), 3.03 (s, 1H), 2.81–2.72 (m, 3H), 2.48 (s, 1H), 2.27 (s, 1H), 2.11 (s, 1H), 2.04 (s, 3H), 1.71 (d, *J* = 9.6 Hz, 3H), 1.62 (s, 1H), 1.57 (s, 1H), 1.32 (s, 1H), 1.20 (s, 3H), 1.14 (s, 3H), 1.13 (s, 3H), 1.11 (s, 3H).^13^C-NMR (150 MHz, DMSO-*d_6_*) *δ*: 178.1, 147.2, 146.8, 145.6, 134.7, 130.3, 127.3, 127.0, 124.7, 124.3, 122.3, 116.4, 109.6, 67.2, 47.7, 47.1, 45.3, 38.2, 37.1, 36.7, 33.4, 30.1, 25.5, 24.5, 21.7, 18.7, 18.1, 16.9. IR (KBr), ν/cm^−1^: 3334, 2866, 1722, 1604, 1500, 1454, 1382. HR-MS-ESI (*m*/*z*): calcd for C_30_H_41_NO_3_ [M+H]^+^: 464.3166; found: 464.3167.

#### 3.2.3. (1R,4aS)-2-Hydroxy-3-(3-Methylphenylamino)Propyl-7-Isopropyl-1,4a-Dimethyl-1,2,3,4,4a,9,10, 10a-Octahydrophenanthrene-1-Carboxylate (**3c**)

Yield 69.3%, as a Yellow oily liquid. ^1^H-NMR (600 MHz, DMSO-*d_6_*) *δ*: 7.15 (s, 1H), 6.97 (s, 1H), 6.89 (s, 1H), 6.80 (s, 1H), 6.32 (t, *J* = 7.3 Hz, 3H), 5.39 (s, 1H), 5.11 (s, 1H), 4.06 (s, 1H), 3.96 (s, 1H), 3.82 (s, 1H), 3.09 (s, 1H), 2.98 (s, 1H), 2.80–2.73 (m, 3H), 2.50 (s, 1H), 2.30 (s, 1H), 2.13 (d, *J* = 9.3 Hz, 4H), 1.72 (d, *J* = 9.2 Hz, 3H), 1.64 (s, 1H), 1.58 (s, 1H), 1.32 (s, 1H), 1.21 (s, 3H), 1.15 (s, 3H), 1.14 (s, 3H), 1.12 (s, 3H).^13^C-NMR (150 MHz, DMSO-*d_6_*) *δ*: 177.6, 148.7, 146.7, 145.2, 137.9, 134.2, 128.8, 126.5, 124.2, 123.8, 116.7, 114.2, 112.7, 109.5, 66.9, 66.7, 47.2, 46.3, 44.8, 37.1, 36.6, 36.2, 32.9, 29.6, 25.0, 24.0, 21.4, 18.2, 16.4, 14.1. IR(KBr), *ν*/cm^−1^: 3404, 2956, 1722, 1606, 1494, 1462, 1382. HR-MS-ESI (*m*/*z*): calcd for C_30_H_41_NO_3_ [M+H]^+^: 464.3166; found: 464.3169.

#### 3.2.4. (1R,4aS)-2-Hydroxy-3-(4-Methylphenylamino)Propyl-7-Isopropyl-1,4a-Dimethyl-1,2,3,4,4a,9,10, 10a-Octahydrophenanthrene-1-Carboxylate (**3d**)

Yield 73.5%, as a white solid. Mp: 90.1–96.2 °C. ^1^H-NMR (600 MHz, DMSO-*d_6_*) *δ*: 7.15 (s, 1H), 6.97 (s, 1H), 6.83 (d, *J* = 8.2 Hz, 3H), 6.48 (d, *J* = 6.7 Hz, 2H), 5.33 (s, 1H), 5.11 (s, 1H), 4.06 (s, 1H), 3.95 (s, 1H), 3.82 (s, 1H), 3.07 (s, 1H), 2.97 (s, 1H), 2.82–2.72 (m, 3H), 2.50 (s, 1H), 2.28 (s, 1H), 2.12 (d, *J* = 9.0 Hz, 4H), 1.72 (d, *J* = 9.0 Hz, 3H), 1.64 (s, 1H), 1.57 (s, 1H), 1.32 (s, 1H), 1.21 (s, 3H), 1.15 (s, 3H), 1.14 (s, 3H), 1.12 (s, 3H).^13^C-NMR (150 MHz, DMSO-*d_6_*) *δ*: 177.6, 146.7, 146.4, 145.1, 134.2, 129.4, 126.5, 124.2, 123.8, 112.4, 67.0, 66.6, 47.2, 46.7, 44.8, 37.7, 36.6, 36.2, 33.0, 29.6, 25.0, 24.0, 21.2, 20.1, 18.2, 16.4. IR (KBr), *ν*/cm^−1^: 3369, 2866, 1701, 1616, 1521, 1462, 1384. HR-MS-ESI (*m*/*z*): calcd for C_30_H_41_NO_3_ [M+H]^+^: 464.3166; found: 464.3168.

#### 3.2.5. (1R,4aS)-2-Hydroxy-3-(2-Methoxyphenylamino)Propyl-7-Isopropyl-1,4a-Dimethyl-1,2,3,4,4a,9, 10,10a-Octahydrophenanthrene-1-Carboxylate (**3e**)

Yield 64.6%, as a Yellow oily liquid. ^1^H-NMR (600 MHz, DMSO-*d_6_*) *δ*: 7.15 (s, 1H), 6.98 (s, 1H), 6.79 (d, *J* = 3.8 Hz, 2H), 6.71 (s, 1H), 6.52 (d, *J* = 4.6 Hz, 2H), 5.23 (s, 1H), 4.05 (s, 1H), 3.98 (s, 1H), 3.87 (s, 1H), 3.36 (s, 4H), 3.17 (s, 1H), 2.99 (s, 1H), 2.77 (d, *J* = 6.8 Hz, 3H), 2.50 (s, 1H), 2.29 (s, 1H), 2.11 (s, 1H), 1.73 (d, *J* = 9.4 Hz, 3H), 1.63 (s, 1H), 1.58 (s, 1H), 1.33 (s, 1H), 1.21 (s, 3H), 1.16 (s, 3H), 1.15 (s, 3H), 1.13 (s, 3H).^13^C-NMR (150 MHz, DMSO-*d_6_*) *δ*:177.5, 146.7, 145.1, 134.2, 126.5, 124.2, 123.8, 121.1, 109.9, 66.8, 66.6, 55.3, 47.2, 46.2, 44.8, 37.7, 36.6, 36.2, 33.0, 29.6, 25.0, 24.0, 21.3, 18.2, 16.4. IR (KBr), *ν*/cm^−1^: 3417, 2931, 1722, 1600, 1516, 1460, 1381. HR-MS-ESI (*m*/*z*): calcd for C_30_H_41_NO_4_ [M+H]^+^: 480.3116; found: 480.3118.

#### 3.2.6. (1R,4aS)-2-Hydroxy-3-(3-Methoxyphenylamino)Propyl-7-Isopropyl-1,4a-Dimethyl-1,2,3,4,4a,9, 10,10a-Octahydrophenanthrene-1-Carboxylate (**3f**)

Yield 69.6%, as a Yellow oily liquid. ^1^H-NMR (400 MHz, DMSO-*d_6_*) *δ*: 6.92 (s, 1H), 6.80–6.57 (m, 3H), 6.14–5.74 (m, 3H), 5.08 (s, 1H), 3.91–3.66 (m, 2H), 3.61 (s, 1H), 3.15 (s, 4H), 2.86 (s, 1H), 2.76 (s, 1H), 2.55 (d, *J* = 6.6 Hz, 3H), 2.28 (s, 1H), 2.06 (s, 1H), 1.88 (s, 1H), 1.50 (d, *J* = 9.4 Hz, 3H), 1.42 (s, 1H), 1.36 (s, 1H), 1.10 (s, 1H), 0.99 (s, 3H), 0.94 (s, 3H), 0.92 (s, 3H), 0.90 (s, 3H).^13^C-NMR (100 MHz, DMSO-*d_6_*) *δ*: 178.0, 160.8, 150.4, 147.1, 145.5, 134.6, 130.0, 126.9, 124.6, 124.2, 105.6, 101.9, 98.4, 67.4, 67.0, 60.2, 55.1, 47.6, 46.8, 45.2, 38.1, 37.0, 36.6, 33.4, 30.1, 25.4, 24.4, 21.2, 18.6, 16.8. IR (KBr), *ν*/cm^−1^: 3404, 2954, 1722, 1612, 1498, 1462, 1381. HR-MS-ESI (*m*/*z*): calcd for C_30_H_41_NO_4_ [M+H]^+^: 480.3116; found: 480.3116.

#### 3.2.7. (1R,4aS)-2-Hydroxy-3-(4-Methoxyphenylamino)Propyl-7-Isopropyl-1,4a-Dimethyl-1,2,3,4,4a,9, 10,10a-Octahydrophenanthrene-1-Carboxylate (**3g**)

Yield 70.8%, as a Yellow oily liquid. ^1^H-NMR (600 MHz, DMSO-*d_6_*) *δ*: 7.15 (s, 1H), 6.98 (s, 1H), 6.82 (s, 1H), 6.67 (d, *J* = 4.1 Hz, 2H), 6.55 (d, *J* = 8.7 Hz, 2H), 5.11 (s, 1H), 4.07 (s, 1H), 3.96 (s, 1H), 3.83 (s, 1H), 3.35 (s, 4H), 3.06 (s, 1H), 2.95 (s, 1H), 2.77 (d, *J* = 6.4 Hz, 3H), 2.50 (s, 1H), 2.29 (s, 1H), 2.11 (s, 1H), 1.72 (d, *J* =9.8 Hz, 3H), 1.63 (s, 1H), 1.58 (s, 1H), 1.32 (s, 1H), 1.21 (s, 3H), 1.15 (s, 3H), 1.14 (s, 3H), 1.13 (s, 3H).^13^C-NMR (150 MHz, DMSO-*d_6_*) *δ*: 177.6, 151.1, 146.7, 145.2, 134.2, 126.5, 124.2, 123.8, 114.6, 113.6, 66.9, 66.7, 55.4, 47.4, 47.2, 44.8, 37.7, 36.6, 36.2, 32.9, 29.6, 25.0, 24.0, 21.3, 18.2, 16.4. IR (KBr), *ν*/cm^−1^: 3363, 2937, 1697, 1612, 1516, 1462, 1384. HR-MS-ESI (*m*/*z*): calcd for C_30_H_41_NO_4_ [M+H]^+^: 480.3116; found: 480.3118.

#### 3.2.8. (1R,4aS)-2-Hydroxy-3-(2-Fluorophenylamino)Propyl-7-Isopropyl-1,4a-Dimethyl-1,2,3,4,4a,9,10, 10a-Octahydrophenanthrene-1-Carboxylate (**3h**)

Yield 78.3%, as a Yellow oily liquid. ^1^H-NMR (600 MHz, DMSO-*d_6_*) *δ*: 7.15 (s, 1H), 6.97 (d, *J* = 1.6 Hz, 2H), 6.84 (d, *J* = 6.2 Hz, 2H), 6.70 (s, 1H), 6.52 (s, 1H), 5.26 (s, 1H), 4.75 (s, 1H), 4.04 (s, 1H), 3.99 (s, 1H), 3.88 (s, 1H), 3.05 (s, 1H), 2.83–2.70 (m, 3H), 2.50 (s, 2H), 2.29 (s, 1H), 2.11 (s, 1H), 1.73 (d, *J* = 9.6 Hz, 3H), 1.63 (s, 1H), 1.58 (s, 1H), 1.33 (m, 1H), 1.20 (s, 3H), 1.16 (s, 3H), 1.14 (s, 3H), 1.13 (s, 3H).^13^C-NMR (150 MHz, DMSO-*d_6_*) *δ*: 178.0, 152.3, 150.7, 147.2, 145.6, 137.3, 134.7, 127.0, 125.2, 124.3, 116.2, 114.8, 112.6, 68.9, 67.3, 66.4, 47.7, 46.6, 45.3, 38.2, 37.1, 33.4, 30.1, 24.5, 21.7, 18.7, 16.9, 15.6. IR (KBr), *ν*/cm^−1^: 3410, 2931, 1722, 1620, 1519, 1454, 1382. HR-MS-ESI (*m*/*z*): calcd for C_29_H_38_FNO_3_ [M+H]^+^: 468.2916; found: 468.2918.

#### 3.2.9. (1R,4aS)-2-Hydroxy-3-(3-Fluorophenylamino)Propyl-7-Isopropyl-1,4a-Dimethyl-1,2,3,4,4a,9,10, 10a-Octahydrophenanthrene-1-Carboxylatev (**3i**)

Yield 73.2%, as a Yellow oily liquid. ^1^H-NMR (600 MHz, DMSO-*d_6_*) *δ*: 7.15 (s, 1H), 6.97 (d, *J* = 7.9 Hz, 2H), 6.81 (s, 1H), 6.38 (s, 1H), 6.35 (s, 1H), 6.25 (s, 1H), 5.94 (s, 1H), 5.15 (s, 1H), 4.07 (s, 1H), 3.97 (s, 1H), 3.82 (s, 1H), 3.11 (s, 1H), 3.01 (s, 1H), 2.81–2.72 (m, 3H), 2.50 (s, 1H), 2.30 (s, 1H), 2.10 (s, 1H), 1.73 (d, *J* = 8.8 Hz, 3H), 1.64 (s, 1H), 1.58 (s, 1H), 1.32 (s, 1H), 1.20 (s, 3H), 1.15 (s, 3H), 1.14 (s, 3H), 1.13 (s, 3H).^13^C-NMR (150MHz, DMSO-*d_6_*) *δ*: 177.6, 164.4, 162.8, 150.9, 146.7, 145.1, 134.2, 130.2, 126.5, 124.2, 123.8, 108.3, 101.7, 98.1, 66.9, 66.5, 65.9, 47.2, 46.2, 44.7, 37.7, 36.6, 33.0, 29.6, 25.0, 24.0, 18.2, 16.4. IR (KBr), ν/cm^−1^: 3410, 2870, 1722, 1620, 1498, 1462, 1382. HR-MS-ESI (*m*/*z*): calcd for C_29_H_38_FNO_3_ [M+H]^+^: 468.2916; found: 468.2919.

#### 3.2.10. (1R,4aS)-2-Hydroxy-3-(4-Fluorophenylamino)Propyl-7-Isopropyl-1,4a-Dimethyl-1,2,3,4,4a,9,10, 10a-Octahydrophenanthrene-1-Carboxylate (**3j**)

Yield 72.5%, as a Yellow oily liquid. ^1^H-NMR (600 MHz, DMSO-*d_6_*) *δ*: 7.15 (s, 1H), 6.98 (s, 1H), 6.82 (d, *J* = 2.6 Hz, 3H), 6.54 (d, *J* = 4.3 Hz, 2H), 5.48 (s, 1H), 5.11 (s, 1H), 4.04 (s, 1H), 3.96 (s, 1H), 3.82 (s, 1H), 3.08 (s, 1H), 2.96 (s, 1H), 2.76 (t, *J* = 8.9 Hz, 3H), 2.50 (s, 1H), 2.29 (s, 1H), 2.12 (s, 1H), 1.72 (d, *J* = 9.8 Hz, 3H), 1.64 (s, 1H), 1.57 (s, 1H), 1.32 (s, 1H), 1.20 (s, 3H), 1.15 (s, 3H), 1.14 (s, 3H), 1.13 (s, 3H).^13^C-NMR (150 MHz, DMSO-*d_6_*) *δ*: 177.5, 155.1, 153.5, 146.7, 145.5, 145.2, 134.2, 126.5, 124.2, 123.8, 115.3, 112.8, 66.9, 66.6, 47.2, 46.9, 44.8, 39.6, 37.7, 36.6, 36.2, 33.0, 29.6, 25.0, 24.0, 21.3, 18.2, 16.4. IR(KBr), *ν*/cm^−1^: 3398, 2929, 1722, 1612, 1516, 1463, 1381. HR-MS-ESI (*m*/*z*): calcd for C_29_H_38_FNO_3_ [M+H]^+^: 468.2916; found: 468.2918.

#### 3.2.11. (1R,4aS)-2-Hydroxy-3-(3-Chlorophenylamino)Propyl-7-Isopropyl-1,4a-Dimethyl-1,2,3,4,4a,9,10, 10a-Octahydrophenanthrene-1-Carboxylate (**3k**)

Yield 75.0%, as a Yellow oily liquid. ^1^H-NMR (600 MHz, DMSO-*d_6_*) *δ*: 7.15 (s, 1H), 6.97 (d, *J* = 4.0 Hz, 2H), 6.81 (s, 1H), 6.59 (s, 1H), 6.50 (d, *J* = 7.9 Hz, 2H), 5.96 (s, 1H), 5.14 (s, 1H), 4.07 (s, 1H), 3.96 (s, 1H), 3.82 (s, 1H), 3.10 (s, 1H), 3.00 (s, 1H), 2.77 (d, *J* = 3.0 Hz, 3H), 2.50 (s, 1H), 2.28 (s, 1H), 2.09 (s, 1H), 1.73 (d, *J* = 8.5 Hz, 3H), 1.64 (s, 1H), 1.58 (s, 1H), 1.33 (s, 1H), 1.21 (s, 3H), 1.15 (s, 3H), 1.14 (s, 3H), 1.13 (s, 3H).^13^C-NMR (150 MHz, DMSO-*d_6_*) *δ*: 177.5, 150.3, 146.7, 145.2, 134.2, 133.7, 130.3, 126.5, 124.2, 123.8, 115.0, 111.1, 110.8, 66.9, 66.5, 47.2, 46.0, 44.8, 37.7, 36.6, 36.2, 32.9, 29.7, 25.0, 24.0, 21.2, 18.2, 16.4. IR(KBr), *ν*/cm^−1^: 3404, 2929, 1722, 1598, 1496, 1463, 1381. HR-MS-ESI (*m*/*z*): calcd for C_29_H_38_ClNO_3_ [M+H]^+^: 484.2620; found: 484.2624.

#### 3.2.12. (1R,4aS)-2-Hydroxy-3-(4-Chlorophenylamino)Propyl-7-Isopropyl-1,4a-Dimethyl-1,2,3,4,4a,9,10, 10a-Octahydrophenanthrene-1-Carboxylate (**3l**)

Yield 70.2%, as a white solid. Mp: 137.5–139.7 °C. ^1^H-NMR (600 MHz, DMSO-*d_6_*) *δ*: 7.15 (s, 1H), 7.05–6.96 (m, 3H), 6.81 (s, 1H), 6.56 (d, *J* = 8.7 Hz, 2H), 5.75 (s, 1H), 5.11 (s, 1H), 4.07 (s, 1H), 3.96 (s, 1H), 3.82 (s, 1H), 3.09 (s, 1H), 2.99 (s, 1H), 2.81–2.73 (m, 3H), 2.50 (s, 1H), 2.31 (s, 1H), 2.12 (s, 1H), 1.73 (d, *J* = 9.2 Hz, 3H), 1.63 (s, 1H), 1.58 (s, 1H), 1.32 (s, 1H), 1.21 (s, 3H), 1.16 (s, 3H), 1.15 (s, 3H), 1.13 (s, 3H).^13^C-NMR (150 MHz, DMSO-*d_6_*) *δ*: 178.0, 148.2, 147.2, 145.7, 134.7, 129.5, 127.0, 124.7, 124.3, 119.4, 113.9, 67.5, 67.0, 47.7, 46.8, 45.3, 38.2, 37.1, 36.7, 33.4, 30.1, 25.5, 24.5, 21.7, 18.7, 16.9. IR(KBr), *ν*/cm^−1^: 3367, 2956, 1699, 1602, 1500, 1462, 1386. HR-MS-ESI (*m*/*z*): calcd for C_29_H_38_ClNO_3_ [M+H]^+^: 484.2620; found: 484.2620.

#### 3.2.13. (1R,4aS)-2-Hydroxy-3-(3-Bromophenylamino)Propyl-7-Isopropyl-1,4a-Dimethyl-1,2,3,4,4a,9,10, 10a-Octahydrophenanthrene-1-Carboxylate (**3m**)

Yield 69.1%, as a Yellow oily liquid. ^1^H-NMR (600 MHz, DMSO-*d_6_*) *δ*: 7.15 (s, 1H), 6.97 (d, *J* = 8.2 Hz, 2H), 6.82 (s, 1H), 6.74 (s, 1H), 6.62 (s, 1H), 6.55 (s, 1H), 5.95 (s, 1H), 5.05 (s, 1H), 4.06 (s, 1H), 3.96 (s, 1H), 3.81 (s, 1H), 3.10 (s, 1H), 3.00 (s, 1H), 2.76 (d, *J* = 6.7 Hz, 3H), 2.50 (s, 1H), 2.28 (s, 1H), 2.10 (s, 1H), 1.73 (d, *J* = 9.0 Hz, 3H), 1.63 (s, 1H), 1.58 (s, 1H), 1.33 (s, 1H), 1.21 (s, 3H), 1.15 (s, 3H), 1.14 (s, 3H), 1.13 (s, 3H).^13^C-NMR (150 MHz, DMSO-*d_6_*) *δ*: 177.6, 150.5, 146.7, 145.2, 134.2, 130.7, 126.5, 124.2, 123.8, 122.4, 117.9, 114.0, 111.1, 66.9, 66.5, 47.2, 46.0, 44.5, 37.7, 36.6, 36.2, 32.9, 29.7, 25.0, 24.0, 21.3, 18.2, 16.4. IR(KBr), *ν*/cm^−1^: 3402, 2929, 1722, 1597, 1500, 1381. HR-MS-ESI (*m*/*z*): calcd for C_29_H_38_BrNO_3_ [M+H]^+^: 528.2115; found: 528.2118.

#### 3.2.14. (1R,4aS)-2-Hydroxy-3-(4-Bromophenylamino)Propyl-7-Isopropyl-1,4a-Dimethyl-1,2,3,4,4a,9,10, 10a-Octahydrophenanthrene-1-Carboxylate (**3n**)

Yield 67.6%, as a white solid. Mp: 98.3–104.4 °C. ^1^H-NMR (600 MHz, DMSO-*d_6_*) *δ*: 7.15 (s, 1H), 7.11 (d, *J* = 8.5 Hz, 2H), 6.97 (s, 1H), 6.80 (s, 1H), 6.51 (d, *J* = 8.5 Hz, 2H), 5.80 (s, 1H), 5.14 (s, 1H), 4.06 (s, 1H), 3.95 (s, 1H), 3.81 (s, 1H), 3.08 (s, 1H), 2.98 (s, 1H), 2.80–2.70 (m, 3H), 2.50 (s, 1H), 2.28 (s, 1H), 2.10 (s, 1H), 1.71 (d, *J* = 9.8 Hz, 3H), 1.63 (s, 1H), 1.57 (s, 1H), 1.31 (s, 1H), 1.20 (s, 3H), 1.15 (s, 3H), 1.14 (s, 3H), 1.12 (s, 3H).^13^C-NMR (150 MHz, DMSO-*d_6_*) *δ*: 177.6, 148.1, 146.7, 145.2, 134.2, 131.4, 126.5, 124.2, 123.8, 114.0, 106.2, 66.8, 66.5, 47.2, 46.2, 46.1, 44.8, 37.7, 36.6, 36.2, 32.9, 29.6, 25.0, 24.0, 21.3, 18.2, 16.4. IR (KBr), *ν*/cm^−1^: 3375, 2954, 1701, 1597, 1498. 1462, 1388. HR-MS-ESI (*m*/*z*): calcd for C_29_H_38_BrNO_3_ [M+H]^+^: 528.2115; found: 528.2116.

#### 3.2.15. (1R,4aS)-2-Hydroxy-3-(3-Ethynylphenylamino)Propyl-7-Isopropyl-1,4a-Dimethyl-1,2,3,4,4a,9, 10,10a-Octahydrophenanthrene-1-Carboxylate **3o**

Yield 76.6%, as a Yellow oily liquid. ^1^H-NMR (600 MHz, DMSO-*d_6_*) *δ*: 7.15 (s, 1H), 6.97 (d, *J* = 7.7 Hz, 2H), 6.82 (s, 1H), 6.65 (s, 1H), 6.61 (d, *J* = 7.6 Hz, 2H), 5.79 (s, 1H), 5.14 (s, 1H), 4.04 (s, 1H), 3.99 (d, *J* = 6.0 Hz, 2H), 3.82 (s, 1H), 3.10 (s, 1H), 3.00 (s, 1H), 2.79–2.75 (m, 3H), 2.50 (s, 1H), 2.28 (s, 1H), 2.11 (s, 1H), 1.73 (d, *J* = 8.7 Hz, 3H), 1.64 (s, 1H), 1.57 (s, 1H), 1.32 (s, 1H), 1.21 (s, 3H), 1.15 (s, 3H), 1.14 (s, 3H), 1.13 (s, 3H).^13^C-NMR (150 MHz, DMSO-*d_6_*) *δ*: 177.6, 148.8, 146.7, 145.2, 134.2, 129.2, 126.5, 124.2, 123.82, 122.2, 119.1, 114.5, 113.1, 84.5, 79.4, 66.6, 47.2, 46.0, 44.8, 37.7, 36.6, 33.0, 29.6, 25.0, 24.0, 21.2, 18.2, 16.4. IR (KBr), *ν*/cm^−1^: 3402, 2929, 1722, 1600, 1492, 1462, 1381. HR-MS-ESI (*m*/*z*): calcd for C_31_H_39_NO_3_ [M+H]^+^: 474.3010; found: 474.3015.

### 3.3. Synthesis: General Procedure for Compounds **4a**–**o**

Compounds **3** (0.22 mmol) and 0.22 mL 6M sodium hydroxide were added to tetrahydrofuran (5 mL) and stirred at ice bath temperature. BTC (0.1 mmol) in DCM (5 mL) was dripped into the mixture and stirred at ice bath temperature for 2 h. After the reaction, water (10 mL) was poured into the mixture and extracted with DCM (3 × 15 mL). The organic layer was combined, dried over anhydrous Na_2_SO_4_, filtered and concentrated under reduced pressure. Then the residue was purified by chromatography on silica gel eluted with petroleum ether/ethyl acetate (*v*:*v* = 4:1~2:1) to offer compounds **4a**–**o**.

#### 3.3.1. (1R,4aS)-(2-Oxo-3-Phenyloxazolidin-5-yl)Methyl-7-Isopropyl-1,4a-Dimethyl-1,2,3,4,4a,9,10,10a-Octahydrophenanthrene-1-Carboxylate (**4a**)

Yield 58.0%, as a white solid. Mp: 127.5–136.7 °C. ^1^H-NMR (600 MHz, DMSO-*d_6_*) *δ*: 7.48 (d, *J* = 7.8 Hz, 2H), 7.25 (d, *J* = 7.6 Hz, 2H), 7.07 (s, 1H), 7.02 (s, 1H), 6.95 (s, 1H), 6.76 (s, 1H), 4.96 (s, 1H), 4.35 (s, 1H), 4.27 (d, *J* = 4.3 Hz, 2H), 3.82 (s, 1H), 2.81–2.65 (m, 3H), 2.50 (s, 1H), 2.19 (s, 1H), 1.96 (s, 1H), 1.67 (d, *J* = 3.6 Hz, 3H), 1.51 (d, *J* = 9.3 Hz, 2H), 1.31 (s, 1H), 1.16 (s, 3H), 1.15 (s, 3H), 1.14 (s, 3H), 1.07 (s, 3H).^13^C-NMR (150 MHz, DMSO-*d_6_*) *δ*: 177.3, 154.1, 146.4, 145.0, 138.1, 134.0, 128.8, 126.5, 124.1, 123.8, 123.5, 117.8, 70.2, 65.0, 47.3, 46.3, 44.9, 37.5, 36.6, 36.3, 36.1, 32.9, 29.7, 29.4, 25.1, 24.0, 21.2, 18.1, 16.3. IR (KBr), *ν*/cm^−1^: 3427, 2951, 1755, 1649, 1598, 1496, 1408, 1379. HR-MS-ESI (*m*/*z*): calcd for C_30_H_37_NO_4_ [M+H]^+^: 476.2803; found: 476.2803.

#### 3.3.2. (1R,4aS)-(3-(2-Methylphenyl)-2-Oxooxazolidin-5-yl)Methyl-7-Isopropyl-1,4a-Dimethyl-1,2,3,4, 4a,9,10,10a-Octahydrophenanthrene-1-Carboxylate (**4b**)

Yield 60.6%, as a Yellow oily liquid. ^1^H-NMR (600 MHz, DMSO-*d_6_*) *δ*: 7.19 (d, *J* = 3.9 Hz, 2H), 7.12 (d, *J* = 5.2 Hz, 2H), 7.01 (s, 1H), 6.91 (s, 1H), 6.80 (s, 1H), 4.97 (s, 1H), 4.34 (s, 1H), 4.22 (s, 1H), 4.00 (s, 1H), 3.68 (s, 1H), 2.84–2.71 (m, 3H), 2.48 (s, 1H), 2.32 (s, 1H), 2.14 (s, 1H), 2.10 (d, *J* = 5.9 Hz, 3H), 1.73 (d, *J* = 9.4 Hz, 3H), 1.60 (d, *J* = 9.7 Hz, 2H), 1.32 (s, 1H), 1.23 (s, 3H), 1.16 (s, 3H), 1.15 (s, 3H),1.13 (s, 3H).^13^C-NMR (150 MHz, DMSO-*d_6_*) *δ*: 177.9, 155.6, 147.0, 145.7, 136.6, 136.1, 134.7, 131.4, 128.3, 127.1, 124.7, 71.4, 65.1, 60.3, 49.0, 47.8, 45.5, 38.2, 37.1, 33.5, 30.0, 25.6, 24.4, 21.8, 21.3, 18.6, 17.9, 16.9, 14.6. IR (KBr), *ν*/cm^−1^: 3514, 2956, 1762, 1606, 1496, 1460, 1411, 1375. HR-MS-ESI (*m*/*z*): calcd for C_31_H_39_NO_4_ [M+H]^+^: 490.2959, found 490.2957.

#### 3.3.3. (1R,4aS)-(3-(3-Methylphenyl)-2-Oxooxazolidin-5-yl)Methyl-7-Isopropyl-1,4a-Dimethyl-1,2,3,4, 4a,9,10,10a-Octahydrophenanthrene-1-Carboxylate (**4c**)

Yield 54.4%, as a Yellow oily liquid. ^1^H-NMR (600 MHz, DMSO-*d_6_*) *δ*: 7.33 (s, 1H), 7.25 (s, 1H), 7.11 (d, *J* = 8.2 Hz, 2H), 6.95 (s, 1H), 6.85 (s, 1H), 6.76 (s, 1H), 4.94 (s, 1H), 4.38 (s, 1H), 4.22 (d, *J* = 4.2 Hz, 2H), 3.78 (s, 1H), 2.80–2.67 (m, 3H), 2.50 (s, 1H), 2.25 (d, *J* = 4.3 Hz, 3H), 2.22 (s, 1H), 1.98 (s, 1H), 1.75–1.57 (m, 3H), 1.52 (d, *J* = 9.4 Hz, 2H), 1.29 (s, 1H), 1.16 (s, 3H), 1.15 (s, 3H), 1.14 (s, 3H), 1.07 (s, 3H).^13^C-NMR (150 MHz, DMSO-*d_6_*) *δ*: 177.3, 154.0, 146.4, 145.0, 138.1, 134.1, 128.6, 126.5, 124.2, 123.8, 118.4, 115.1, 70.2, 65.0, 47.3, 46.4, 44.9, 37.5, 36.6, 36.1, 32.9, 29.7, 25.0, 24.0, 21.3, 18.1, 16.3. IR (KBr), *ν*/cm^−1^: 2929, 1761, 1608, 1494, 1456, 1408. HR-MS-ESI (*m*/*z*): calcd for C_31_H_39_NO_4_ [M+H]^+^: 490.2959; found: 490.2962.

#### 3.3.4. (1R,4aS)-(3-(4-Methylphenyl)-2-Oxooxazolidin-5-yl)Methyl-7-Isopropyl-1,4a-Dimethyl-1,2,3,4, 4a,9,10,10a-Octahydrophenanthrene-1-Carboxylate (**4d**)

Yield 57.7%, as a white solid. Mp: 156.5–158.2 °C. ^1^H-NMR (600 MHz, DMSO-*d_6_*) *δ*: 7.30 (d, *J* = 8.5 Hz, 2H), 7.11–7.06 (m, 3H), 6.97 (s, 1H), 6.74 (s, 1H), 4.93 (s, 1H), 4.39 (s, 1H), 4.19 (d, *J* = 4.0 Hz, 2H), 3.76 (s, 1H), 2.78–2.68 (m, 3H), 2.50 (s, 1H), 2.25 (s, 3H), 2.23 (s, 1H), 1.97 (s, 1H), 1.66 (d, *J* = 9.3 Hz, 2H), 1.57 (s, 1H), 1.50 (d, *J* = 9.1 Hz, 2H), 1.25 (s, 1H), 1.16 (s, 3H), 1.14 (s, 3H), 1.13 (s, 3H), 1.07 (s, 3H).^13^C-NMR (150 MHz, DMSO-*d_6_*) *δ*: 177.3, 154.1, 146.5, 145.1, 135.8, 134.1, 132.5, 129.3, 126.5, 124.1, 123.7, 117.9, 70.2, 64.8, 47.3, 46.3, 44.6, 37.5, 36.5, 32.9, 29.4, 25.0, 24.0, 21.3, 20.4, 18.0, 16.3. IR (KBr), *ν*/cm^−1^: 3304, 2926, 1747, 1641, 1562, 1514, 1373. HR-MS-ESI (*m*/*z*): calcd for C_31_H_39_NO_4_ [M+H]^+^: 490.2959; found: 490.2955.

#### 3.3.5. (1R,4aS)-(3-(2-Methoxyphenyl)-2-Oxooxazolidin-5-yl)Methyl-7-Isopropyl-1,4a-Dimethyl-1,2,3,4, 4a,9,10,10a-Octahydrophenanthrene-1-Carboxylate (**4e**)

Yield 62.3%, as a Yellow oily liquid. ^1^H-NMR (600 MHz, DMSO-*d_6_*) *δ*: 7.25 (s, 1H), 7.18 (s, 1H), 7.14 (s, 1H), 7.08 (s, 1H), 7.00 (s, 1H), 6.82 (s, 1H), 6.73 (s, 1H), 4.94 (s, 1H), 4.35 (s, 1H), 4.24 (s, 1H), 4.02 (s, 1H), 3.74 (s, 1H), 3.67 (s, 1H), 2.83–2.73 (m, 3H), 2.50 (s, 1H), 2.31 (s, 1H), 2.12 (s, 1H), 1.98 (s, 1H), 1.83–1.68 (m, 3H), 1.63 (d, *J* = 8.6 Hz, 2H), 1.33 (d, *J* = 8.9 Hz, 2H), 1.24 (s, 3H), 1.17 (s, 3H), 1.15 (s, 3H), 1.14 (s, 3H).^13^C-NMR (150 MHz, DMSO-*d_6_*) *δ*: 177.4, 155.7, 154.8, 146.5, 145.2, 134.4, 128.8, 128.3, 126.6, 125.8, 123.9, 120.5, 112.5, 70.9, 64.6, 59.8, 55.7, 48.0, 47.4, 45.0, 39.6, 37.7, 36.6, 33.0, 29.7, 25.1, 24.0, 20.8, 18.1, 16.3, 14.1. IR (KBr), *ν*/cm^−1^: 2954, 1761, 1597, 1504, 1460, 1411. HR-MS-ESI (*m*/*z*): calcd for C_31_H_39_NO_5_ [M+H]^+^: 506.2908; found: 506.2910.

#### 3.3.6. (1R,4aS)-(3-(3-Methoxyphenyl)-2-Oxooxazolidin-5-yl)Methyl-7-Isopropyl-1,4a-Dimethyl-1,2,3,4, 4a,9,10,10a-Octahydrophenanthrene-1-Carboxylate (**4f**)

Yield 69.5%, as a Yellow oily liquid. ^1^H-NMR (600 MHz, DMSO-*d_6_*) *δ*: 7.25 (s, 1H), 7.18 (s, 1H), 7.12 (s, 1H), 7.08 (s, 1H), 7.00 (s, 1H), 6.73 (d, *J* = 7.6 Hz, 2H), 4.94 (s, 1H), 4.35 (s, 1H), 4.22 (s, 1H), 3.98 (s, 1H), 3.74 (s, 2H), 3.66 (s, 1H), 2.84–2.72 (m, 3H), 2.50 (s, 1H), 2.31 (s, 1H), 2.12 (s, 1H), 1.85–1.68 (m, 3H), 1.65 (d, *J* = 3.4 Hz, 2H), 1.35 (d, *J* = 7.6 Hz, 2H), 1.24 (s, 3H), 1.17 (s, 3H), 1.15 (s, 3H), 1.14 (s, 3H).^13^C-NMR (150 MHz, DMSO-*d_6_*) *δ*: 177.4, 155.7, 154.8, 146.5, 145.2, 134.1, 128.8, 128.3, 126.6, 125.8, 124.2, 120.5, 112.5, 70.9, 64.6, 55.7, 48.0, 47.7, 47.4, 45.0, 37.7, 36.7, 36.2, 33.0, 29.7, 25.1, 24.0, 21.3, 18.1, 16.4. IR (KBr), *ν*/cm^−1^: 3481, 2931, 1762, 1597, 1504, 1462, 1411, 1375. HR-MS-ESI (*m*/*z*): calcd for C_31_H_39_NO_5_ [M+H]^+^: 506.2908; found: 506.2906.

#### 3.3.7. (1R,4aS)-(3-(4-Methoxyphenyl)-2-Oxooxazolidin-5-yl)Methyl-7-Isopropyl-1,4a-Dimethyl-1,2,3,4, 4a,9,10,10a-Octahydrophenanthrene-1-Carboxylate (**4g**)

Yield 71.0%, as a white solid. Mp: 110.7–119.8 °C. ^1^H-NMR (600 MHz, DMSO-*d_6_*) *δ*: 7.34 (s, 1H), 7.29 (s, 1H), 7.10 (s, 1H), 6.96 (s, 1H), 6.79 (t, *J* = 9.8 Hz, 3H), 4.92 (s, 1H), 4.38 (s, 1H), 4.19 (d, *J* = 4.0 Hz, 2H), 3.70 (s, 3H), 2.81–2.67 (m, 3H), 2.50 (s, 1H), 2.20 (s, 1H), 1.97 (s, 1H), 1.76–1.54 (m, 3H), 1.52 (d, *J* = 9.5 Hz, 2H), 1.25 (d, *J* = 5.4 Hz, 2H), 1.15 (s, 3H), 1.15 (s, 3H), 1.14 (s, 3H), 1.07 (s, 3H).^13^C-NMR (150 MHz, DMSO-*d_6_*) *δ*: 177.4, 155.5, 154.2, 146.5, 145.1, 134.2, 131.3, 126.5, 124.2, 123.7, 120.1, 119.8, 114.0, 70.1, 64.9, 55.2, 47.3, 46.6, 45.0, 44.6, 37.5, 36.5, 36.3, 32.9, 29.4, 25.0, 23.9, 21.3, 18.0, 16.2. IR (KBr), *ν*/cm^−1^: 2954, 1753, 1610, 1514, 1440, 1409, 1384. HR-MS-ESI (*m*/*z*): calcd for C_31_H_39_NO_5_ [M+H]^+^: 506.2908; found: 506.2910.

#### 3.3.8. (1R,4aS)-(3-(2-Fluorophenyl)-2-Oxooxazolidin-5-yl)Methyl-7-Isopropyl-1,4a-Dimethyl-1,2,3,4,4a, 9,10,10a-Octahydrophenanthrene-1-Carboxylate (**4h**)

Yield 59.6%, as a Yellow oily liquid. ^1^H-NMR (600 MHz, DMSO-*d_6_*) *δ*: 7.39 (s, 1H), 7.26 (s, 1H), 7.16 (d, *J* = 8.2 Hz, 2H), 7.04 (s, 1H), 7.00 (s, 1H), 6.80 (s, 1H), 5.01 (s, 1H), 4.37 (s, 1H), 4.27 (s, 1H), 4.12 (s, 1H), 3.79 (s, 1H), 2.85–2.69 (m, 3H), 2.50 (s, 1H), 2.31 (s, 1H), 2.08 (s, 1H), 1.76 (d, *J* = 6.7 Hz, 3H), 1.61 (d, *J* = 5.4 Hz, 2H), 1.34 (s, 1H), 1.22 (s, 3H), 1.16 (s, 3H), 1.15 (s, 3H), 1.13 (s, 3H). ^13^C-NMR (150 MHz, DMSO-*d_6_*) *δ*: 177.8, 157.8, 156.1, 155.5, 147.0, 145.6, 134.6, 129.1, 127.7, 127.0, 125.7, 125.22, 124.7, 124.4, 117.0, 71.7, 65.1, 47.9, 45.4, 38.1, 37.1, 36.6, 33.4, 30.2, 25.6, 24.5, 21.7, 18.6, 16.8. IR (KBr), *ν*/cm^−1^: 3350, 2929, 1762, 1612, 1506, 1460, 1411. HR-MS-ESI (*m*/*z*): calcd for C_30_H_36_FNO_4_ [M+H]^+^: 494.2708; found: 494.2710.

#### 3.3.9. (1R,4aS)-(3-(3-Fluorophenyl)-2-Oxooxazolidin-5-yl)Methyl-7-Isopropyl-1,4a-Dimethyl-1,2,3,4,4a, 9,10,10a-Octahydrophenanthrene-1-Carboxylate (**4i**)

Yield 68.8%, as a white solid. Mp: 108.2–113.2 °C. ^1^H-NMR (600 MHz, DMSO-*d_6_*) *δ*: 7.46 (s, 1H), 7.25 (d, *J* = 7.1 Hz, 2H), 7.06 (s, 1H), 6.95 (s, 1H), 6.81 (s, 1H), 6.74 (s, 1H), 4.99 (s, 1H), 4.33 (s, 1H), 4.25 (d, *J* = 4.1 Hz, 2H), 3.80 (s, 1H), 2.81–2.64 (m, 3H), 2.50 (s, 1H), 2.19 (s, 1H), 1.91 (s, 1H), 1.68 (d, *J* = 4.7 Hz, 3H), 1.55 (s, 1H), 1.52 (s, 1H), 1.29 (s, 1H), 1.16 (s, 3H), 1.15 (s, 3H), 1.14 (s, 3H), 1.06 (s, 3H).^13^C-NMR (150 MHz, DMSO-*d_6_*) *δ*: 177.2, 163.0, 161.5, 154.0, 146.3, 145.0, 139.9, 133.9, 130.5, 126.5, 123.8, 113.2, 109.8, 104.8, 70.5, 64.8, 47.3, 46.4, 44.9, 37.4, 36.6, 36.3, 36.1, 33.0, 29.7, 25.1, 24.0, 21.2, 18.1, 16.3. IR (KBr), *ν*/cm^−1^: 2958, 1747, 1614, 1587, 1494, 1409, 1381. HR-MS-ESI (*m*/*z*): calcd for C_30_H_36_FNO_4_ [M+H]^+^: 494.2708; found: 494.2713.

#### 3.3.10. (1R,4aS)-(3-(4-Fluorophenyl)-2-Oxooxazolidin-5-yl)Methyl-7-Isopropyl-1,4a-Dimethyl-1,2,3,4, 4a,9,10,10a-Octahydrophenanthrene-1-Carboxylate (**4j**)

Yield 56.4%, as a white solid. Mp: 112.8–117.5 °C. ^1^H-NMR (600 MHz, DMSO-*d_6_*) *δ*: 7.46 (s, 1H), 7.40 (s, 1H), 7.07 (d, *J* = 8.2 Hz, 2H), 7.01 (s, 1H), 6.92 (s, 1H), 6.75 (s, 1H), 4.94 (s, 1H), 4.37 (s, 1H), 4.18 (d, *J* = 2.8 Hz, 2H), 3.77 (s, 1H), 2.82–2.64 (m, 3H), 2.48 (s, 1H), 2.19 (s, 1H), 1.95 (s, 1H), 1.75–1.54 (m, 3H), 1.51 (s, 2H), 1.26 (s, 1H), 1.15 (s, 3H), 1.14 (s, 3H), 1.13 (s, 3H), 1.06 (s, 3H).^13^C-NMR (150 MHz, DMSO-*d_6_*) *δ*: 177.8, 159.5, 157.9, 154.6, 147.0, 145.6, 135.2, 134.7, 127.0, 124.6, 124.2, 120.3, 115.8, 70.7, 65.4, 47.8, 47.0, 45.1, 38.0, 37.0, 36.5, 33.4, 29.9, 25.4, 24.5, 21.8, 18.5, 16.7. IR (KBr), *ν*/cm^−1^: 3475, 2949, 1747, 1604, 1512, 1431, 1368. HR-MS-ESI (*m*/*z*): calcd for C_30_H_36_FNO_4_ [M+H]^+^: 494.2708; found: 494.2701.

#### 3.3.11. (1R,4aS)-(3-(3-Chlorophenyl)-2-Oxooxazolidin-5-yl)Methyl-7-Isopropyl-1,4a-Dimethyl-1,2,3,4, 4a,9,10,10a-Octahydrophenanthrene-1-Carboxylate (**4k**)

Yield 66.6%, as a Yellow oily liquid. ^1^H-NMR (600 MHz, DMSO-*d_6_*) *δ*: 7.71 (s, 1H), 7.23 (d, *J* = 8.1 Hz, 2H), 7.10 (d, *J* = 8.2 Hz, 2H), 6.95 (s, 1H), 6.75 (s, 1H), 4.99 (s, 1H), 4.38 (s, 1H), 4.25 (d, *J* = 4.2 Hz, 2H), 3.81 (s, 1H), 2.82–2.61 (m, 3H), 2.50 (s, 1H), 2.22 (s, 1H), 1.98 (s, 1H), 1.69 (d, *J* = 6.0 Hz, 3H), 1.52 (d, *J* = 8.9 Hz, 2H), 1.27 (s, 1H), 1.16 (s, 3H), 1.15 (s, 3H), 1.14 (s, 3H), 1.07 (s, 3H).^13^C-NMR (150 MHz, DMSO-*d_6_*) *δ*: 177.3, 153.9, 146.4, 145.1, 139.5, 133.8, 133.5, 130.4, 126.5, 124.1, 123.8, 123.1, 117.4, 116.2, 70.5, 64.8, 47.3, 46.3, 44.7, 37.5, 36.5, 36.3, 32.9, 29.5, 25.1, 24.0, 21.2, 18.1, 16.2. IR (KBr), *ν*/cm^−1^: 2956, 1762, 1595, 1485, 1444, 1406. HR-MS-ESI (*m*/*z*): calcd for C_30_H_36_ClNO_4_ [M+H]^+^: 510.2413; found: 510.2412.

#### 3.3.12. (1R,4aS)-(3-(4-Chlorophenyl)-2-Oxooxazolidin-5-yl)Methyl-7-Isopropyl-1,4a-Dimethyl-1,2,3,4, 4a,9,10,10a-Octahydrophenanthrene-1-Carboxylate (**4l**)

Yield 67.7%, as a white solid. Mp: 170.6–172.5 °C. ^1^H-NMR (600 MHz, DMSO-*d_6_*) *δ*: 7.43 (d, *J* = 9.0 Hz, 2H), 7.29 (d, *J* = 9.0 Hz, 2H), 7.09 (s, 1H), 6.98 (s, 1H), 6.75 (s, 1H), 4.97 (s, 1H), 4.39 (s, 1H), 4.22 (d, *J* = 3.6 Hz, 2H), 3.78 (s, 1H), 2.80–2.66 (m, 3H), 2.50 (s, 1H), 2.20 (s, 1H), 1.96 (s, 1H), 1.67 (m, 3H), 1.51 (d, *J* = 5.9 Hz, 2H), 1.25 (s, 1H), 1.17 (s, 3H), 1.16 (s, 3H), 1.13 (s, 3H), 1.07 (s, 3H).^13^C-NMR (150 MHz, DMSO-*d_6_*) *δ*: 177.8, 154.4, 147.0, 145.6, 137.7, 134.6, 129.2, 127.7, 127.0, 124.6, 124.2, 119.8, 70.9, 65.4, 47.8, 46.7, 45.1, 40.1, 37.9, 37.0, 36.8, 33.4, 29.9, 25.4, 24.5, 21.8, 18.5, 16.7. IR (KBr), *ν*/cm^−1^: 3124, 2924, 1751, 1595, 1494, 1365. HR-MS-ESI (*m*/*z*): calcd for C_30_H_36_ClNO_4_ [M+H]^+^: 510.2413; found: 510.2406.

#### 3.3.13. (1R,4aS)-(3-(3-Bromophenyl)-2-Oxooxazolidin-5-yl)Methyl-7-Isopropyl-1,4a-Dimethyl-1,2,3,4, 4a,9,10,10a-Octahydrophenanthrene-1-Carboxylate (**4m**)

Yield 70.7%, as a white solid. Mp: 87.8–90.6 °C. ^1^H-NMR (600 MHz, DMSO-*d_6_*) *δ*: 7.84 (s, 1H), 7.38 (s, 1H), 7.22 (s, 1H), 7.15 (s, 1H), 7.05 (s, 1H), 6.93 (s, 1H), 6.74 (s, 1H), 4.96 (s, 1H), 4.36 (s, 1H), 4.21 (d, *J* = 2.4 Hz, 2H), 3.79 (s, 1H), 2.80–2.64 (m, 3H), 2.48 (s, 1H), 2.19 (s, 1H), 1.97 (s, 1H), 1.69 (d, *J* = 6.3 Hz, 3H), 1.50 (d, *J* = 9.6 Hz, 2H), 1.24 (s, 1H), 1.15 (s, 3H), 1.14 (s, 3H), 1.12 (s, 3H), 1.06 (s, 3H). ^13^C-NMR (150 MHz, DMSO-*d_6_*) *δ*: 177.7, 154.4, 146.8, 145.6, 140.3, 134.6, 131.2, 127.0, 126.5, 124.6, 124.3, 122.3, 120.6, 117.1, 70.9, 65.2, 47.8, 46.8, 45.4, 37.9, 36.6, 33.4, 30.0, 25.4, 24.5, 21.7, 18.5, 16.8. IR (KBr), *ν*/cm^−1^: 3496, 2956, 1762, 1593, 1479, 1440, 1406. HR-MS-ESI (*m*/*z*): calcd for C_30_H_36_BrNO_4_ [M+Na]^+^: 576.1726; found: 576.1727.

#### 3.3.14. (1R,4aS)-(3-(4-Bromophenyl)-2-Oxooxazolidin-5-yl)Methyl-7-Isopropyl-1,4a-Dimethyl-1,2,3,4, 4a,9,10,10a-Octahydrophenanthrene-1-Carboxylate (**4n)**

Yield 65.0%, as a white solid. Mp: 127.1–129.4 °C. ^1^H-NMR (600 MHz, DMSO-*d_6_*) *δ*: 7.41 (d, *J* = 9.1 Hz, 2H), 7.34 (d, *J* = 9.0 Hz, 2H), 7.03 (s, 1H), 6.96 (s, 1H), 6.77 (s, 1H), 4.97 (s, 1H), 4.34 (s, 1H), 4.22 (d, *J* = 4.4 Hz, 2H), 3.80 (s, 1H), 2.85–2.65 (m, 3H), 2.50 (s, 1H), 2.16 (s, 1H), 1.99–1.87 (m, 1H), 1.75–1.60 (m, 3H), 1.56 (m, 1H), 1.50 (m, 1H), 1.28 (s, 1H), 1.17 (s, 3H), 1.16 (s, 3H), 1.12 (s, 3H), 1.05 (s, 3H).^13^C-NMR (150 MHz, DMSO-*d_6_*) *δ*: 177.2, 154.0, 146.3, 145.0, 137.4, 133.9, 131.5, 126.4, 124.1, 119.6, 115.4, 70.3, 65.1, 47.3, 46.2, 45.0, 39.5, 37.4, 36.5, 36.0, 32.9, 31.2, 29.7, 25.0, 24.0, 21.2, 18.0, 16.2. IR (KBr), *ν*/cm^−1^: 3433, 2956, 1726, 1591, 1492, 1460, 1396. HR-MS-ESI (*m*/*z*): calcd for C_30_H_36_BrNO_4_ [M+H]^+^: 554.1908; found: 554.1904.

#### 3.3.15. (1R,4aS)-(3-(3-Ehynylphenyl)-2-Oxooxazolidin-5-yl)Methyl-7-Isopropyl-1,4a-Dimethyl-1,2,3,4, 4a,9,10,10a-Octahydrophenanthrene-1-Carboxylate **4o**

Yield 58.7%, as a white solid. Mp: 125.2–130.4 °C. ^1^H-NMR (600 MHz, DMSO-*d_6_*) *δ*: 7.60 (s, 1H), 7.47 (s, 1H), 7.22 (d, *J* = 8.1 Hz, 2H), 7.11 (s, 1H), 6.95 (s, 1H), 6.75 (s, 1H), 4.97 (s, 1H), 4.34 (s, 1H), 4.21 (d, *J* = 7.6 Hz, 2H), 3.81 (s, 1H), 2.81–2.62 (m, 3H), 2.50 (s, 1H), 2.22 (s, 1H), 1.97 (s, 1H), 1.68 (d, *J* = 4.2 Hz, 3H), 1.52 (d, *J* = 9.5 Hz, 2H), 1.29 (s, 1H), 1.25 (s, 1H), 1.16 (s, 3H), 1.15 (s, 3H), 1.13 (s, 3H), 1.07 (s, 3H).^13^C-NMR (150 MHz, DMSO-*d_6_*) *δ*: 177.3, 154.0, 146.3, 145.0, 138.4, 134.1, 129.3, 126.6, 123.8, 122.3, 120.5, 118.2, 83.3, 81.0, 70.5, 65.0, 47.3, 46.2, 44.9, 37.5, 36.6, 36.1, 32.9, 29.5, 25.1, 24.0, 21.2, 18.1, 16.2. IR (KBr), *ν*/cm^−1^: 3238, 2954, 1749, 1598, 1577, 1487, 1440, 1406. HR-MS-ESI (*m*/*z*): calcd for C_32_H_37_NO_4_ [M+H]^+^: 500.2803; found: 500.2795.

### 3.4. Cytotoxicity Assay

The cytotoxicity of the compounds was evaluated against NCI-H460, MGC-803, SK-OV-3, CNE-2 and LO2 using an MTT assay. Cells were grown on 96-well micro-plates at a density of 4 × 10^3^ cells per well in DMEM (dulbecco’s modified eagle medium) medium with 10% FBS (fetal bovine serum). The plates were incubated at 37 °C with 5% CO_2_. The cells were then exposed to different concentrations of target compounds and cisplatin and incubated for another 48 h. Control wells were formed by culture media with the maximum concentration of DMSO used in each assay (1‰) [35]. The cells were stained with 20 µL of MTT in an incubator for about 4 h. The medium was thrown away and replaced by 150 mL DMSO. The absorbance (OD) value was read at 490 nm on an enzyme labeling instrument. Each experiment was repeated at least three times to obtain the mean values [36].

### 3.5. Cell Cycle Analysis

The MGC-803 cell line was treated with different concentrations of compound **4j**. After 48 h of incubation, cells were washed twice with ice-cold PBS, fixed and permeabilized with ice-cold 70% ethanol at −20 °C overnight. The cells were treated with 100 µg /mL RNase A at 37 °C for 30 min, after being washed with ice-cold PBS, and finally stained with 1 mg/mL propidium iodide (PI) (BD, Pharmingen) in the dark at 4 °C for 30 min. Analysis was performed with the system software (Cell Quest; BD Biosciences, Becton Dickinson FACSAriaIII, NY, USA).

### 3.6. Apoptosis Analysis

MGC-803 cells were seeded at a concentration of 2 × 10^6^ cells/mL of the DMEM medium with 10% FBS on 6-well plates to the final volume of 2 mL, and then treated with compound **4j** at different concentrations for 48 h. After 48 h, the cells were collected and washed twice with PBS and then resuspended in 100 µL 1 × binding buffer, The cells were subjected to 5 µL of FITC Annexin V and 5 µL propidium iodide (PI) staining using an annexin-V FITC apoptosis kit (BD, Pharmingen) and incubated for 30 min at RT (25 °C) in the dark. Then, 100µL 1× binding buffer were added. The apoptosis ratio was quantified by system software (Cell Quest; BD Biosciences, Becton Dickinson FACSAriaIII, NY, USA).

### 3.7. Hoechst 33258 Staining Assay

Cells grown on a sterile cover slip in six-well plates were treated with different concentrations of the test compound for 48 h. The culture medium containing compounds was removed, and the cells were fixed in 4% paraformaldehyde for 10 min. After being washed twice with PBS, the cells were stained with 0.5 mL of Hoechst 33258 (Beyotime, Shanghai, China) for 5 min, and then again washed twice with PBS. The stained nuclei were observed under an OLYMPUS 1X73 fluorescence microscope using 350 nm for excitation and 460 nm for emission.

### 3.8. Statistics

All statistical analysis was performed with SPSS Version 18.0. Data was analyzed by one-way ANOVA. Mean separations were performed using the least significant difference method. Each experiment was replicated thrice, and all experiments yielded similar results. Measurements from all the replicates were combined, and treatment effects were analyzed.

## 4. Conclusions

In summary, a series of novel DHAA-oxazolidinone hybrids were designed and synthesized. All the synthesized compounds were evaluated for their cytotoxic effects against the MGC-803, CNE-2, SK-OV-3, NCI-H460 and LO2 cell lines. The results revealed that some compounds exhibited better inhibitory activity than the commercial anticancer drug cisplatin. In particular, compound **4j** (IC_50_ = 3.82 ± 0.18 µM) exhibited the best anticancer activity against the MGC-803 cell line and displayed very weak cytotoxicity on normal cells. The apoptosis-inducing activity investigated by flow cytometry revealed that compound **4j** markedly induced MGC-803 cell apoptosis. The apoptosis-inducing effect of **4j** was further analyzed by Hoechst 33258 staining. In addition, further mechanistic studies revealed that compound **4j** arrested the MGC-803 cell line in the G1 phase. Our present results indicate that these DHAA derivatives may serve as promising precursors for developing more potent antitumor agents.

## Figures and Tables

**Table 1:** Effect of compounds **3a**–**o** and **4a**–**o** against on the cell viability of different cell lines ^a^.

Compound			IC_50_ (μM)		
	MGC-803	CNE-2	SK-OV-3	NCI-H460	LO2
**3a**	7.10 ± 1.51	27.87 ± 0.25	15.08 ± 0.57	18.56 ± 0.97	>100
**3b**	22.56 ± 2.71	>100	>100	>100	>100
**3c**	12.34 ± 4.87	14.61 ± 0.19	14.81 ± 4.03	20.13 ± 2.95	34.67 ± 0.45
**3d**	21.84 ± 2.66	44.13 ± 2.89	29.95 ± 0.80	44.76 ± 5.26	>100
**3e**	28.47 ± 1.35	27.37 ± 0.51	19.48 ± 2.23	26.14 ± 1.92	32.34 ± 1.56
**3f**	18.37 ± 1.45	17.44 ± 4.32	18.01 ± 0.09	21.87 ± 8.61	>100
**3g**	51.69 ± 1.85	>100	>100	>100	>100
**3h**	17.50 ± 3.51	45.74 ± 5.80	>100	30.24 ± 3.40	>100
**3i**	>100	>100	>100	>100	>100
**3j**	14.21 ± 1.70	20.73 ± 2.31	18.91 ± 1.66	15.59 ± 1.45	28.69 ± 0.55
**3k**	13.10 ± 2.66	18.17 ± 4.06	25.07 ± 4.80	18.14 ± 2.33	35.32 ± 0.57
**3l**	34.48 ± 0.02	>100	>100	>100	>100
**3m**	16.48 ± 5.20	23.17 ± 2.78	30.29 ± 0.59	19.63 ± 1.12	>100
**3n**	9.91 ± 7.00	17.89 ± 4.47	19.03 ± 2.11	19.05 ± 5.23	>100
**3o**	13.38 ± 5.41	20.01 ± 1.09	23.08 ± 2.23	22.30 ± 2.74	>100
**4a**	11.00 ± 4.41	9.69 ± 0.13	>100	4.83 ± 0.77	>100
**4b**	24.99 ± 5.02	>100	>100	27.72 ± 5.03	>100
**4c**	10.50 ± 1.44	31.96 ± 0.23	>100	9.13 ± 2.77	36.22 ± 2.68
**4d**	5.97 ± 0.87	21.14 ± 1.96	19.74 ± 1.33	2.91 ± 2.38	30.55 ± 1.99
**4e**	13.23 ± 1.15	20.51 ± 0.97	>100	7.99 ± 6.35	40.56 ± 1.96
**4f**	19.71 ± 2.80	23.53 ± 1.20	42.97 ± 1.05	17.25 ± 1.38	>100
**4g**	6.10 ± 0.35	19.76 ± 0.30	4.10 ± 2.45	14.05 ± 8.04	26.36 ± 0.15
**4h**	49.90 ± 1.14	>100	>100	>100	>100
**4i**	11.48 ± 0.25	28.02 ± 3.95	9.86 ± 4.30	25.09 ± 4.50	>100
**4j**	3.82 ± 0.18	17.76 ± 4.69	4.66 ± 2.13	8.44 ± 0.36	>100
**4k**	7.43 ± 1.42	31.24 ± 2.50	10.33 ± 5.46	22.96 ± 2.62	37.20 ± 3.14
**4l**	5.82 ± 4.82	27.58 ± 1.50	15.71 ± 2.32	26.55 ± 0.08	44.49 ± 2.77
**4m**	9.80 ± 2.37	>100	>100	25.06 ± 1.39	34.16 ± 2.88
**4n**	5.34 ± 3.45	42.49 ± 5.68	>100	30.24 ± 2.66	>100
**4o**	18.22 ± 2.36	51.36 ± 5.06	23.66 ± 2.02	72.36 ± 1.07	>100
**DHA**	29.81 ± 2.06	62.59 ± 1.60	>100	>100	>100
**cisplatin**	14.9 ± 1.78	21.02 ± 2.25	10.44 ± 0.25	24.14 ± 1.74	36.37 ± 0.79

^a^ IC_50_ values are expressed as the mean ± SD (standard deviation) from three independent experiments.

## Supplementary Materials

Supplementary materials can be found at http://www.mdpi.com/1422-0067/19/10/3116/s1.

## Abbreviations

BTC	bis(trichloromethyl)carbonate
DCM	dichloromethane
DHAA	dehydroabietic acid
DMEM	dulbecco’s modified eagle medium
DMSO	dimethyl sulfoxide
FBS	fetal bovine serum
FITC	fluorescein isothiocyanate
HR-MS	high resolution mass spectrometr
IR	infrared radiation
Mp	melting point
MRSA	methicillin-resistant staphylococcus aureus
MRSE	methicillin-resistant staphylococcus epidermidis
MTT	methyl thiazolytetrazolium
NMR	nuclear magnetic resonance
PI	propidium iodide
THF	tetrahydrofuran
TLC	thin-layer chromatography
TMS	tetramethylsilane
VRE	vancomycin-resistant enterococcus
