# Supplementary material for: Synthesis and Biological Evaluation of Novel Dehydroabietic Acid-Oxazolidinone Hybrids for Antitumor Properties
Source: Int J Mol Sci. 2018 Oct 11;19(10):3116. doi: 10.3390/ijms19103116 (PMC6213879; doi:10.3390/ijms19103116)
Supplement: Supplementary file 1 [file ijms-19-03116-s001.pdf]

Synthesis and Biological Evaluation of Novel Dehydroabietic Acid-Oxazolidinones Hybrids for Antitumor Properties

Xiu Wang , Fu-Hua Pang, Lin Huang, Xin-Ping Yang, Xian-Li Ma, Cai-Na Jiang, Fang-Yao Li and Fu-Hou Lei

Figure S: FTIR ,^{1}H-NMR, ^{13}C-NMR and HRMS-ESI spectrum of compounds **3a-o** and **4a-o**

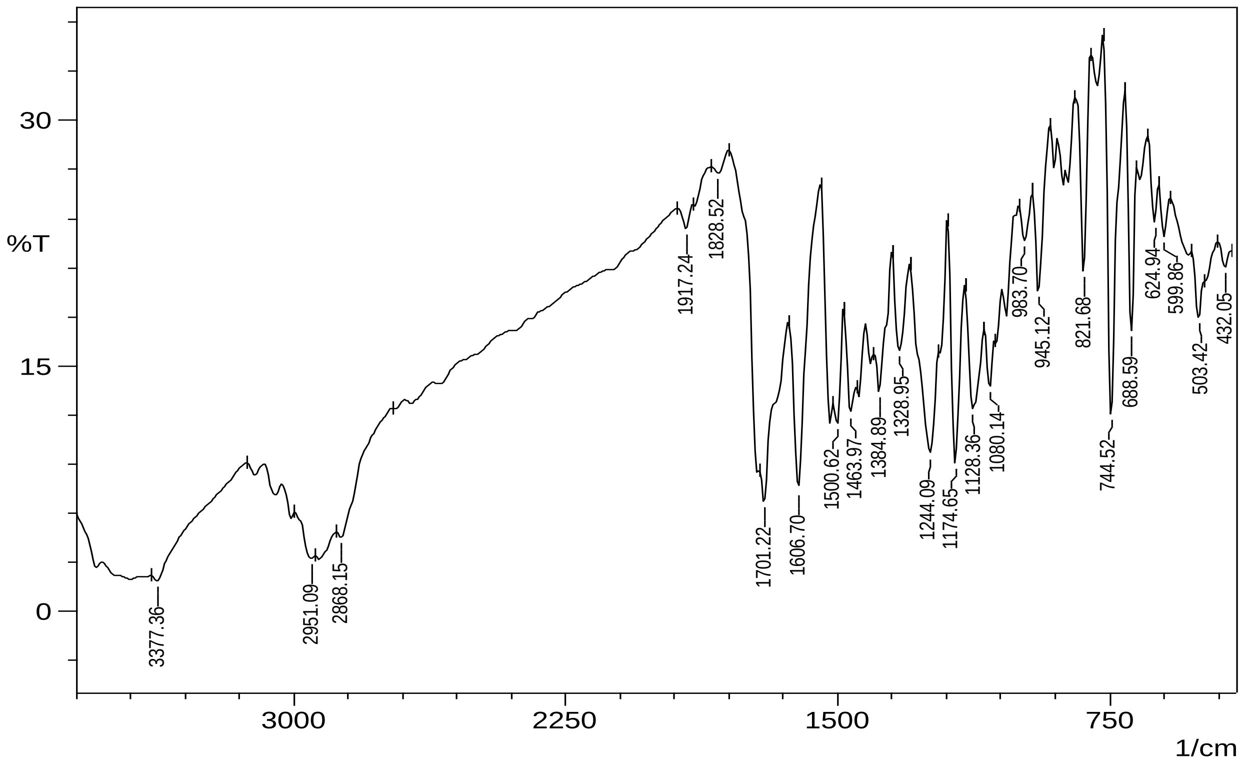

Figure S1. FTIR spectrum of the target compound (**3a**)

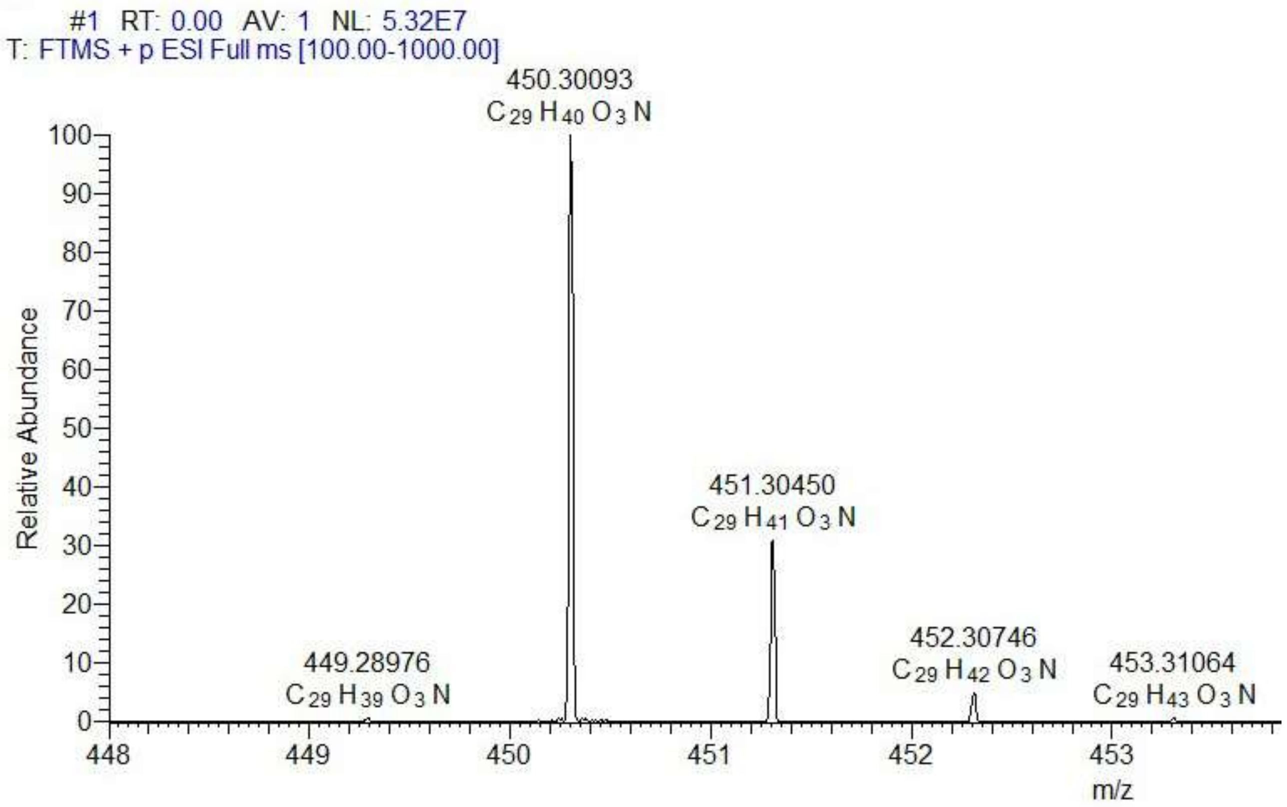

Figure S2. HRMS-ESI spectrum of the target compound (**3a**)

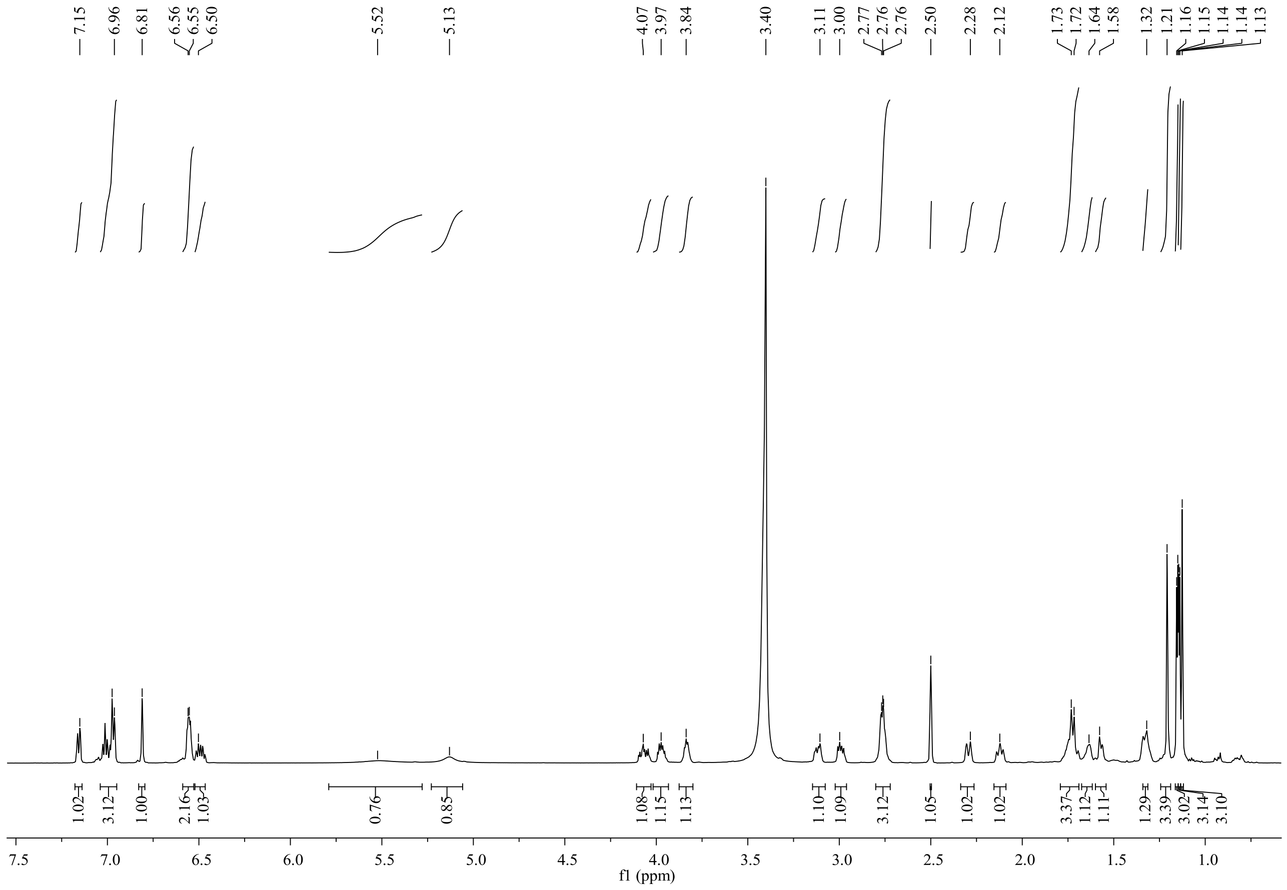

Figure S3. ^{1}H-NMR spectrum of the target compound (**3a**) in DMSO

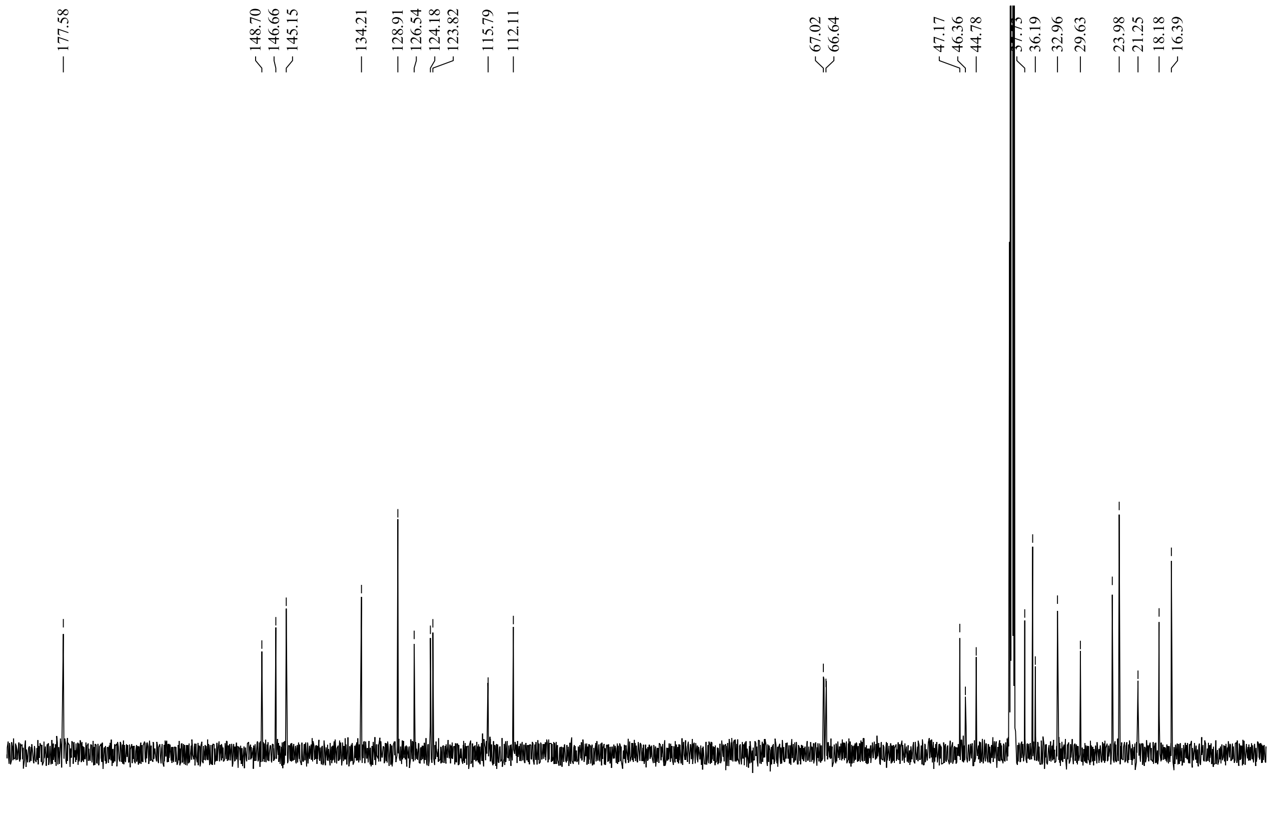

Figure S4. ^{13}C-NMR spectrum of the target compound (**3a**) in DMSO

Int. J. Mol. Sci. 2018, 19, 3116; doi:10.3390/ijms19103116

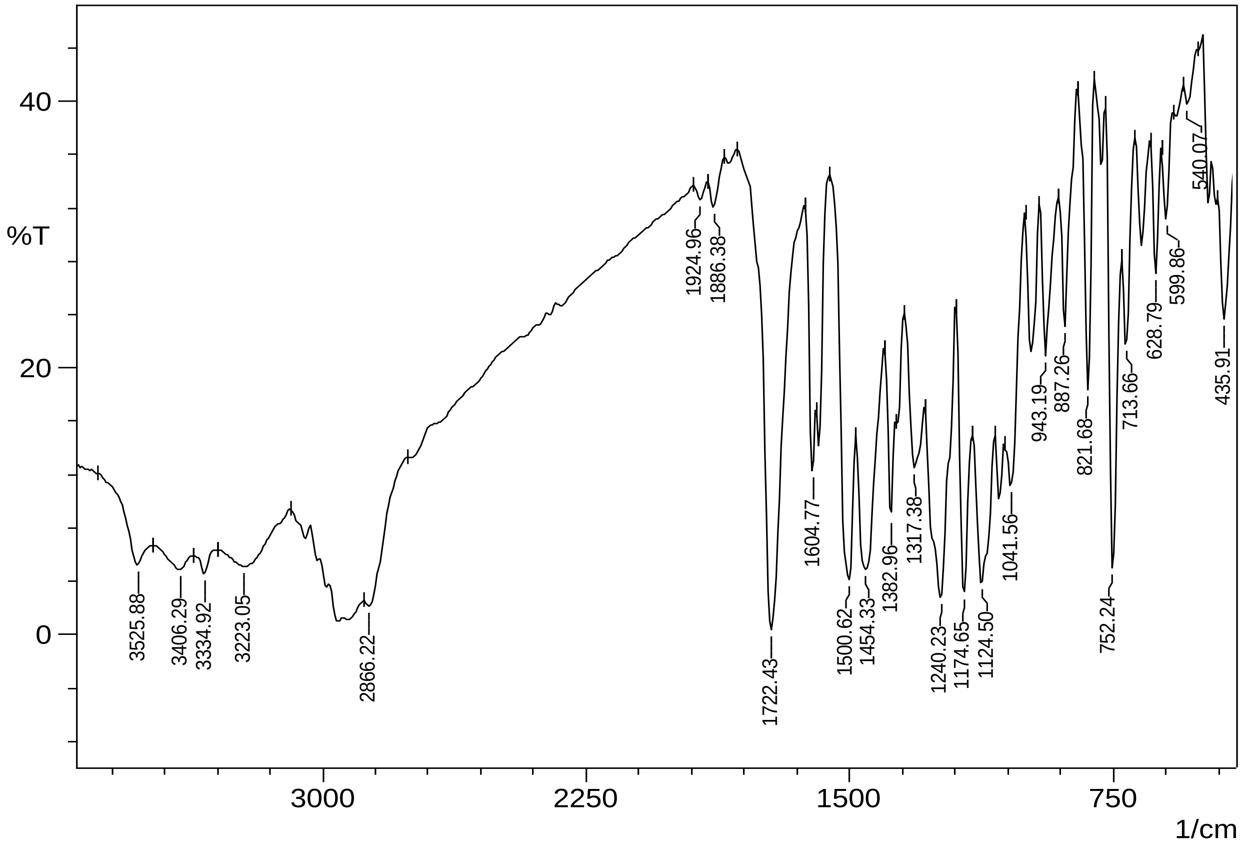

Figure S5. FTIR spectrum of the target compound (**3b**)

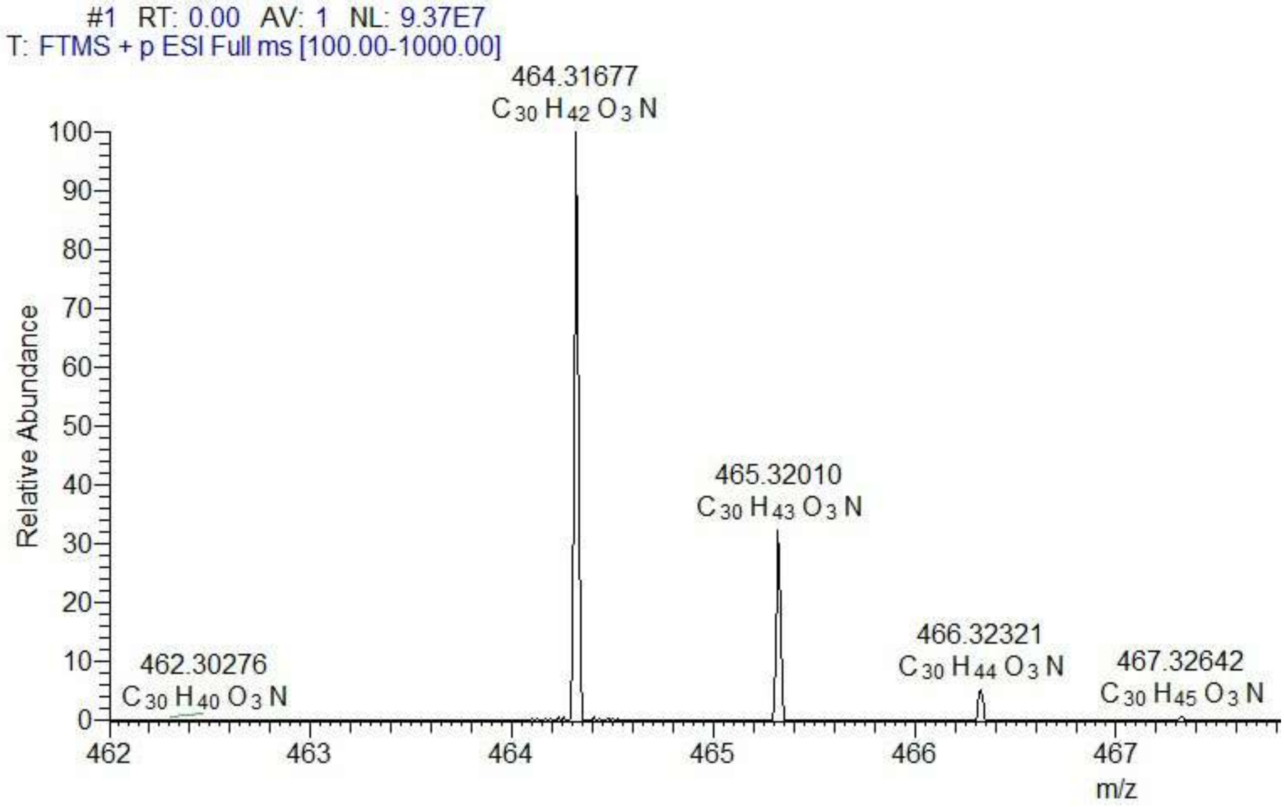

Figure S6. HRMS-ESI spectrum of the target compound (**3b**)

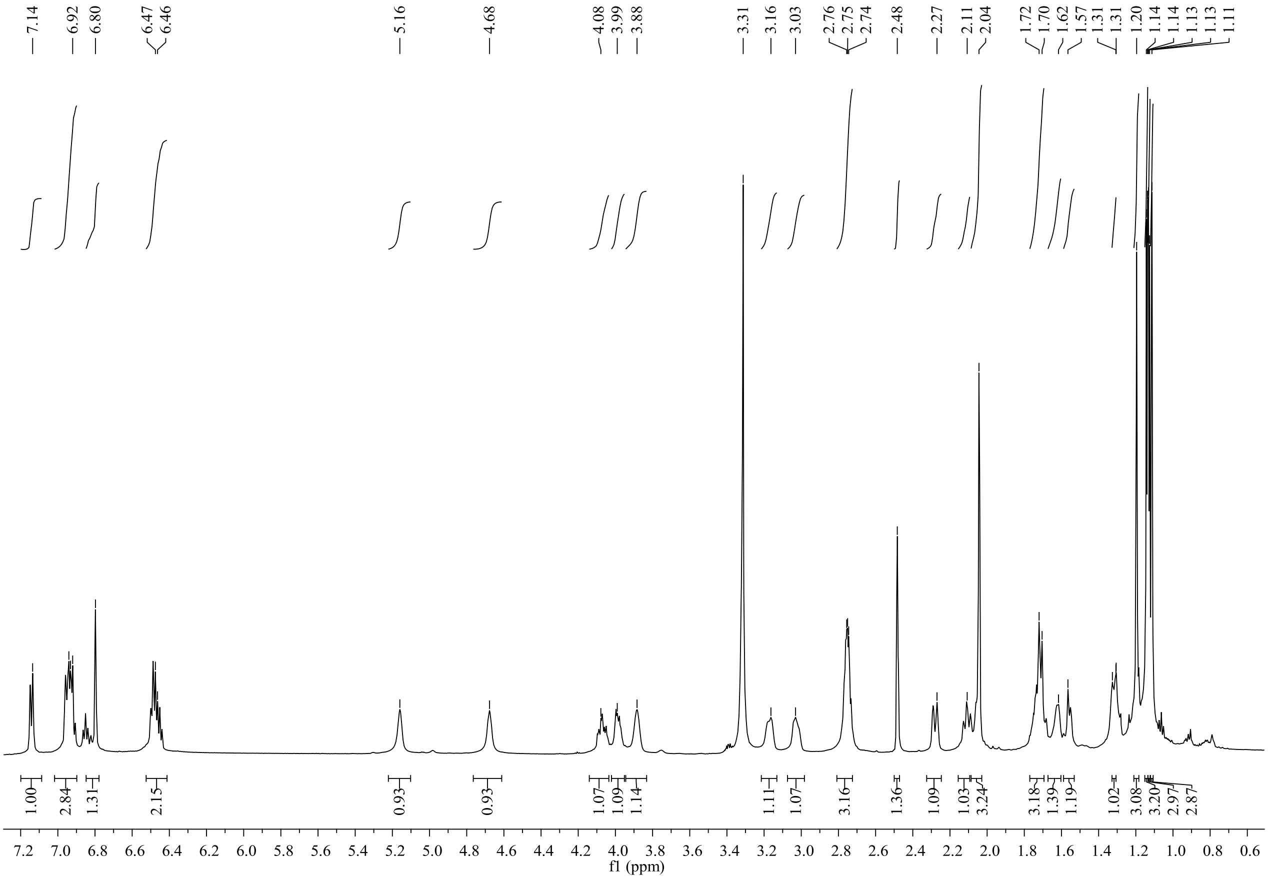

Figure S7. ^{1}H-NMR spectrum of the target compound (**3b**) in DMSO

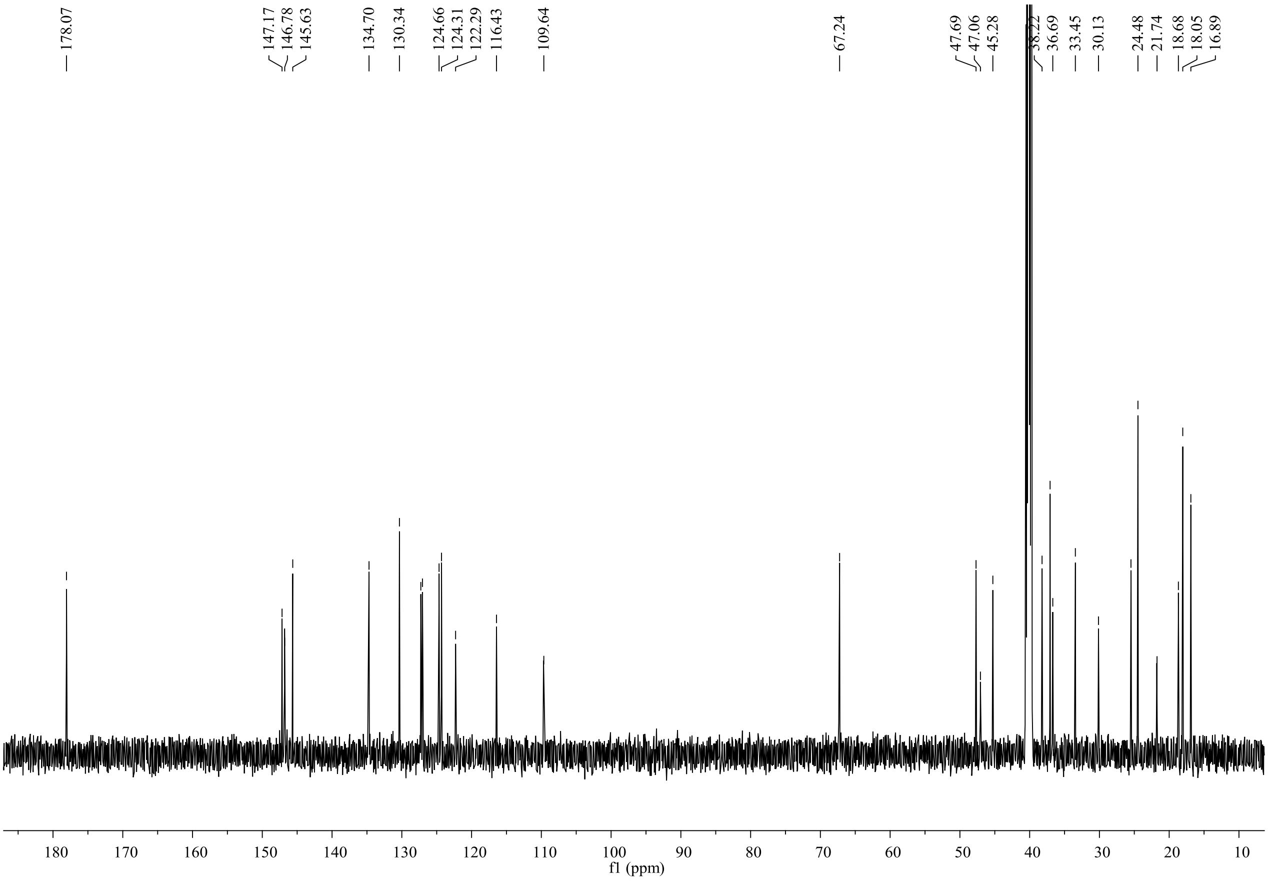

Figure S8. ^{13}C-NMR spectrum of the target compound (**3b**) in DMSO

Int. J. Mol. Sci. 2018, 19, 3116; doi:10.3390/ijms19103116

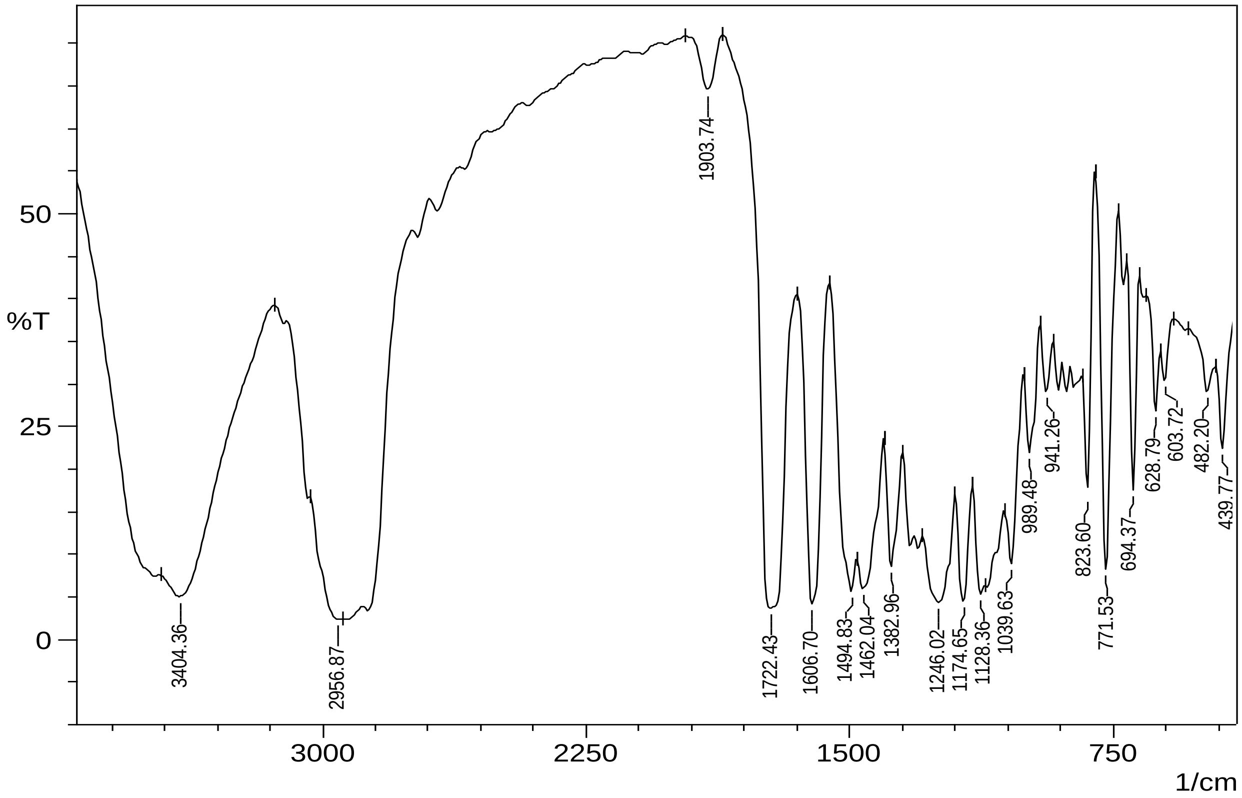

Figure S9. FTIR spectrum of the target compound (**3c**)

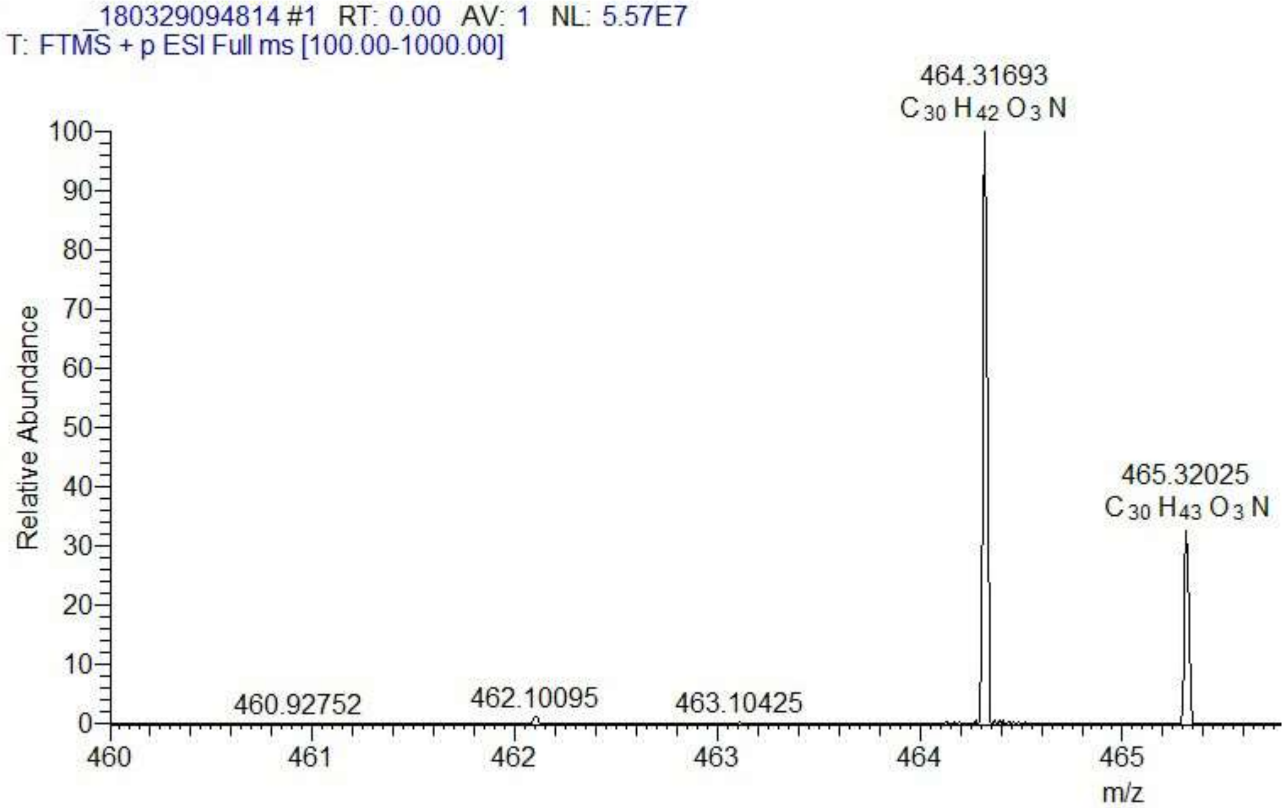

Figure S10. HRMS-ESI spectrum of the target compound (**3c**)

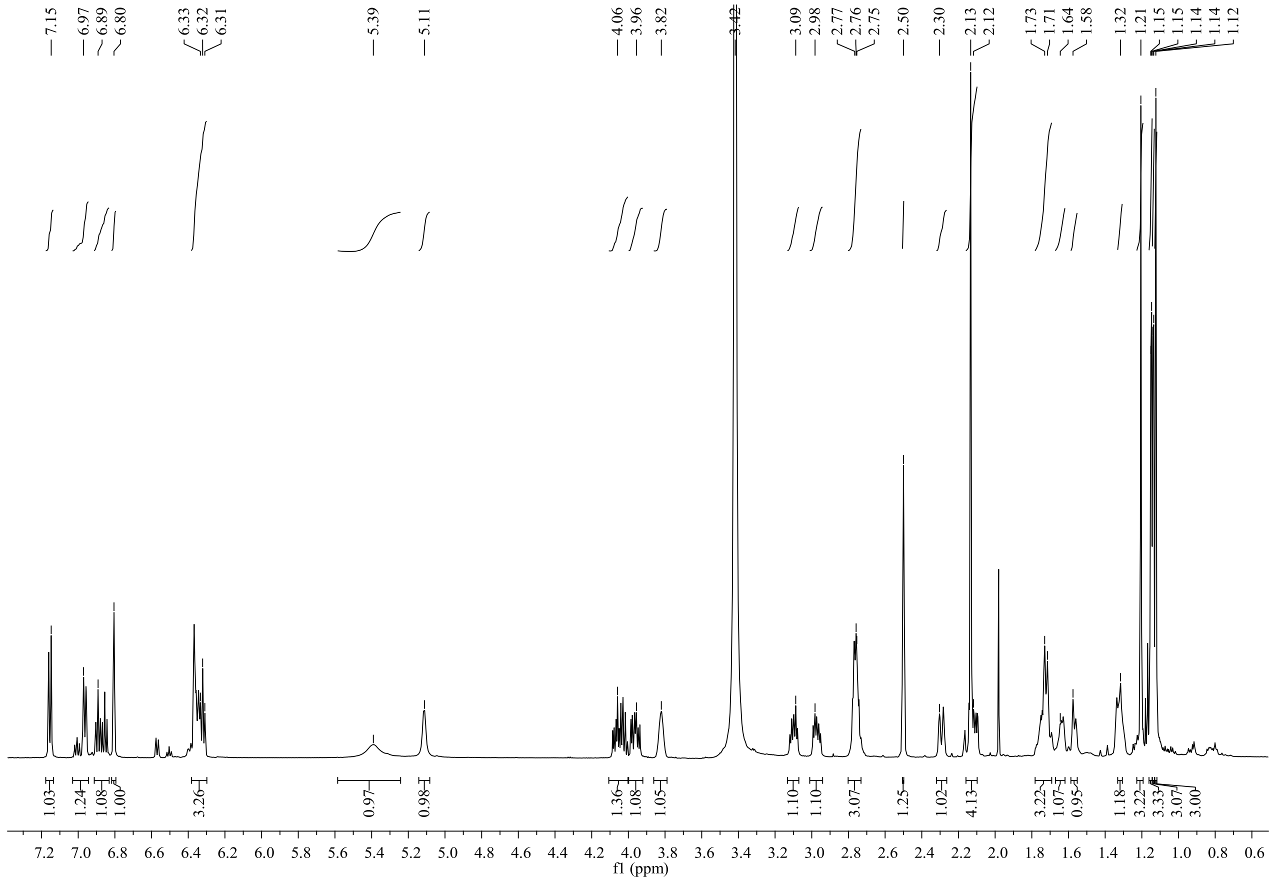

Figure S11. ^{1}H-NMR spectrum of the target compound (**3c**) in DMSO

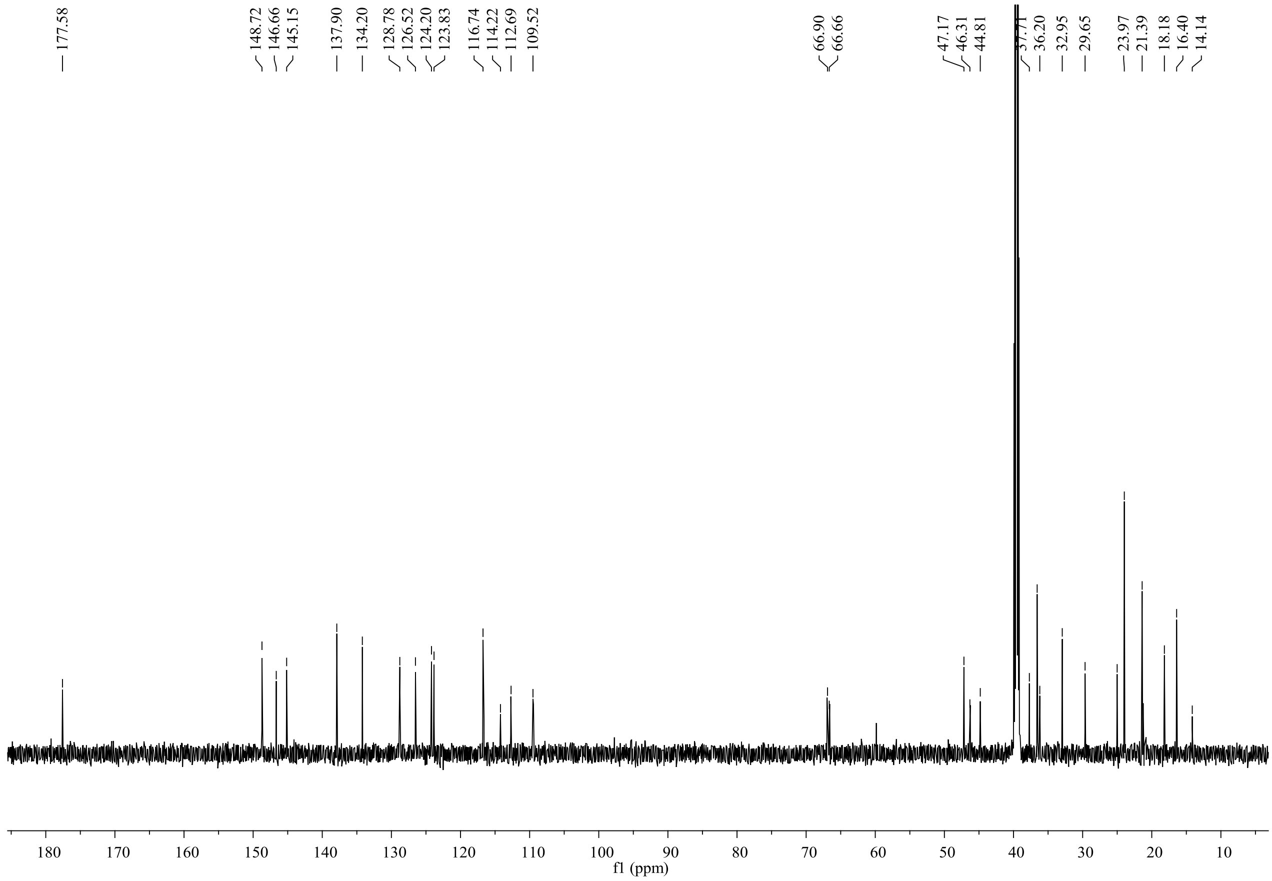

Figure S12. ^{13}C-NMR spectrum of the target compound (**3c**) in DMSO

Int. J. Mol. Sci. 2018, 19, 3116; doi:10.3390/ijms19103116

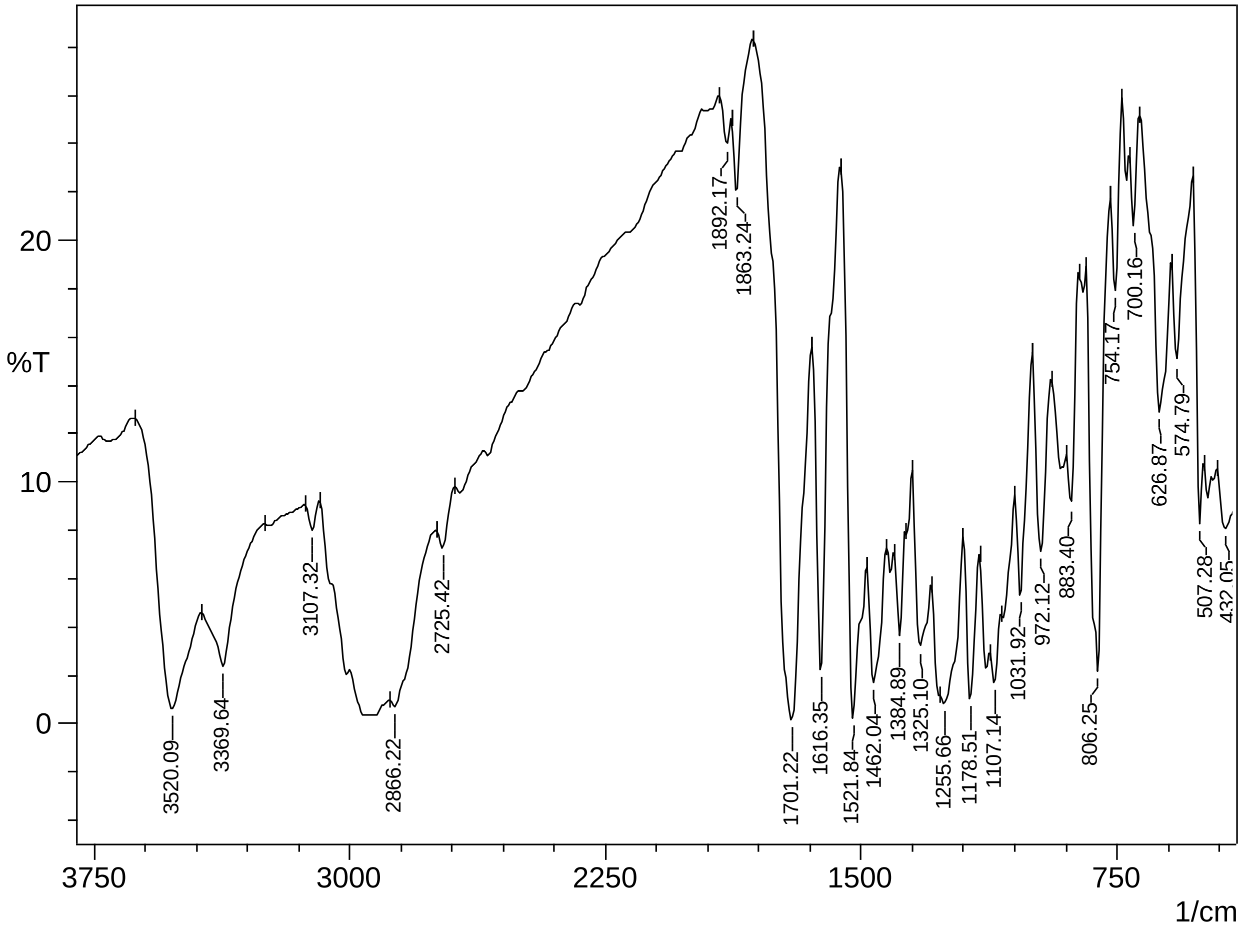

Figure S13. FTIR spectrum of the target compound (**3d**)

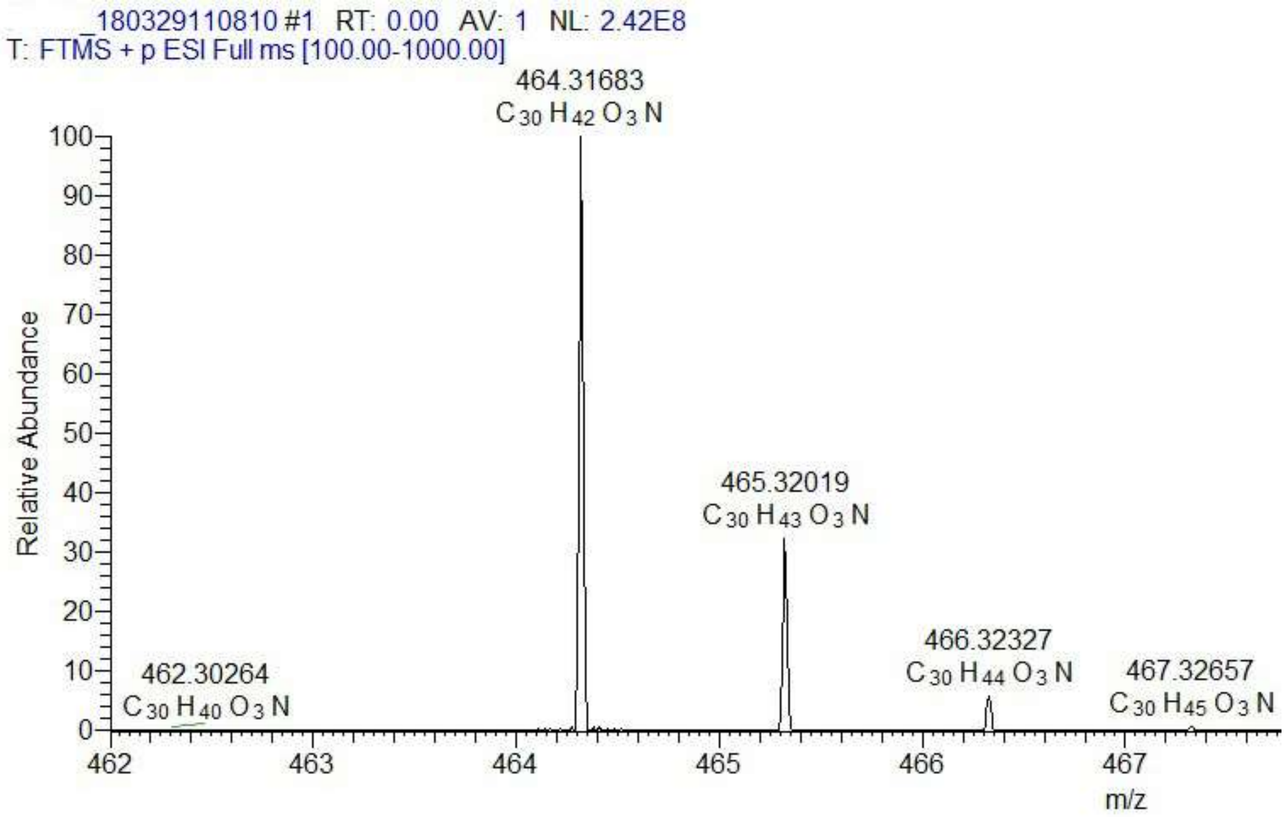

Figure S14. HRMS-ESI spectrum of the target compound (**3d**)

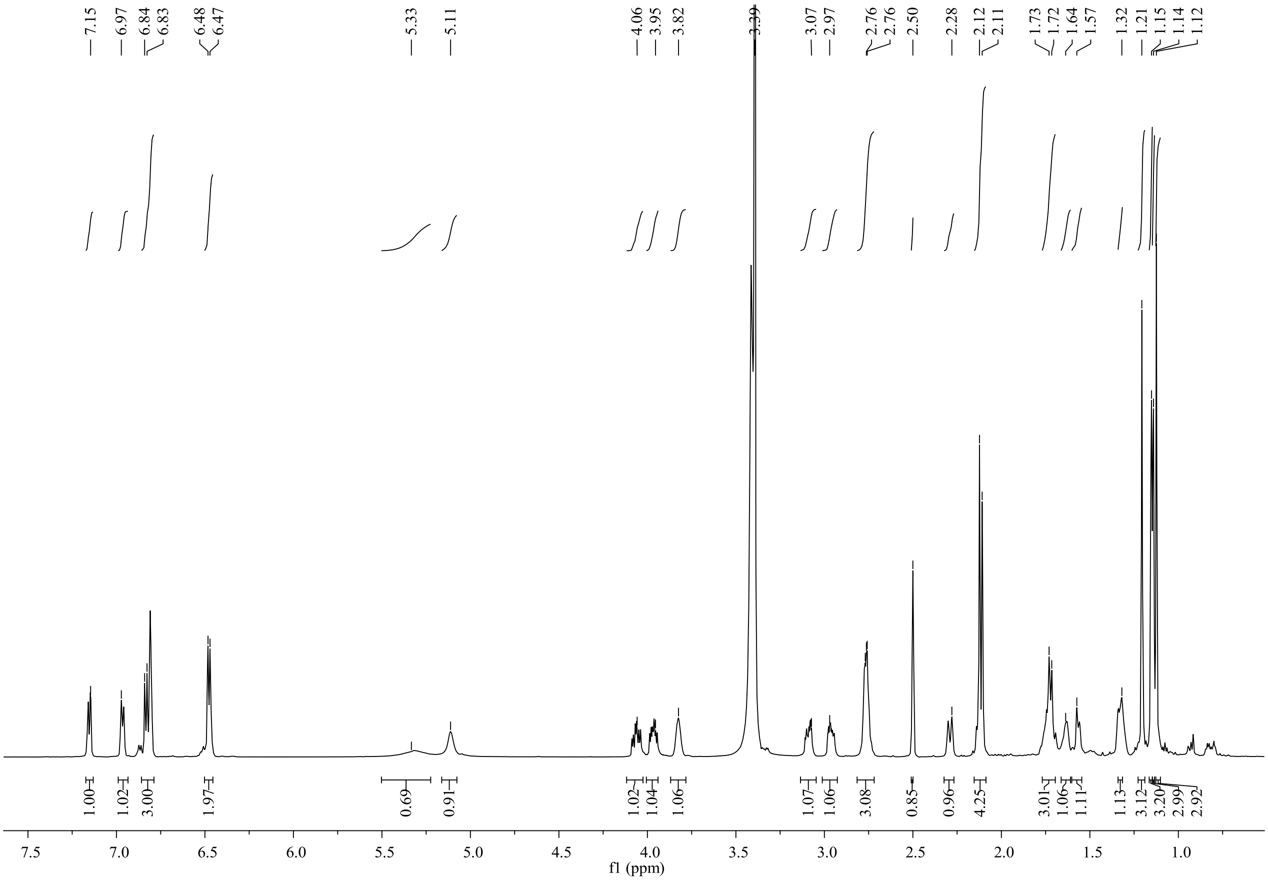

Figure S15. ^{1}H-NMR spectrum of the target compound (**3d**) in DMSO

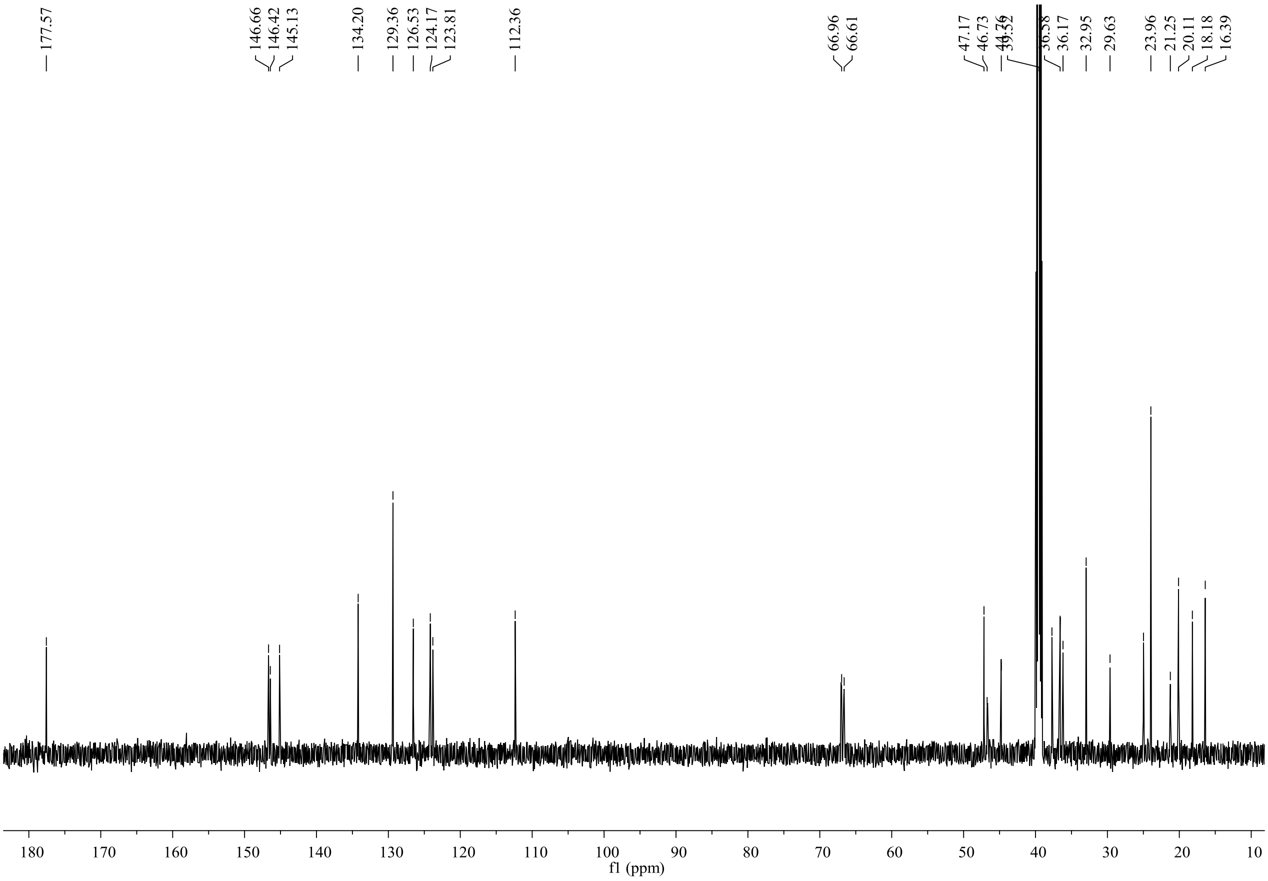

Figure S16. ^{13}C-NMR spectrum of the target compound (**3d**) in DMSO

Int. J. Mol. Sci. 2018, 19, 3116; doi:10.3390/ijms19103116

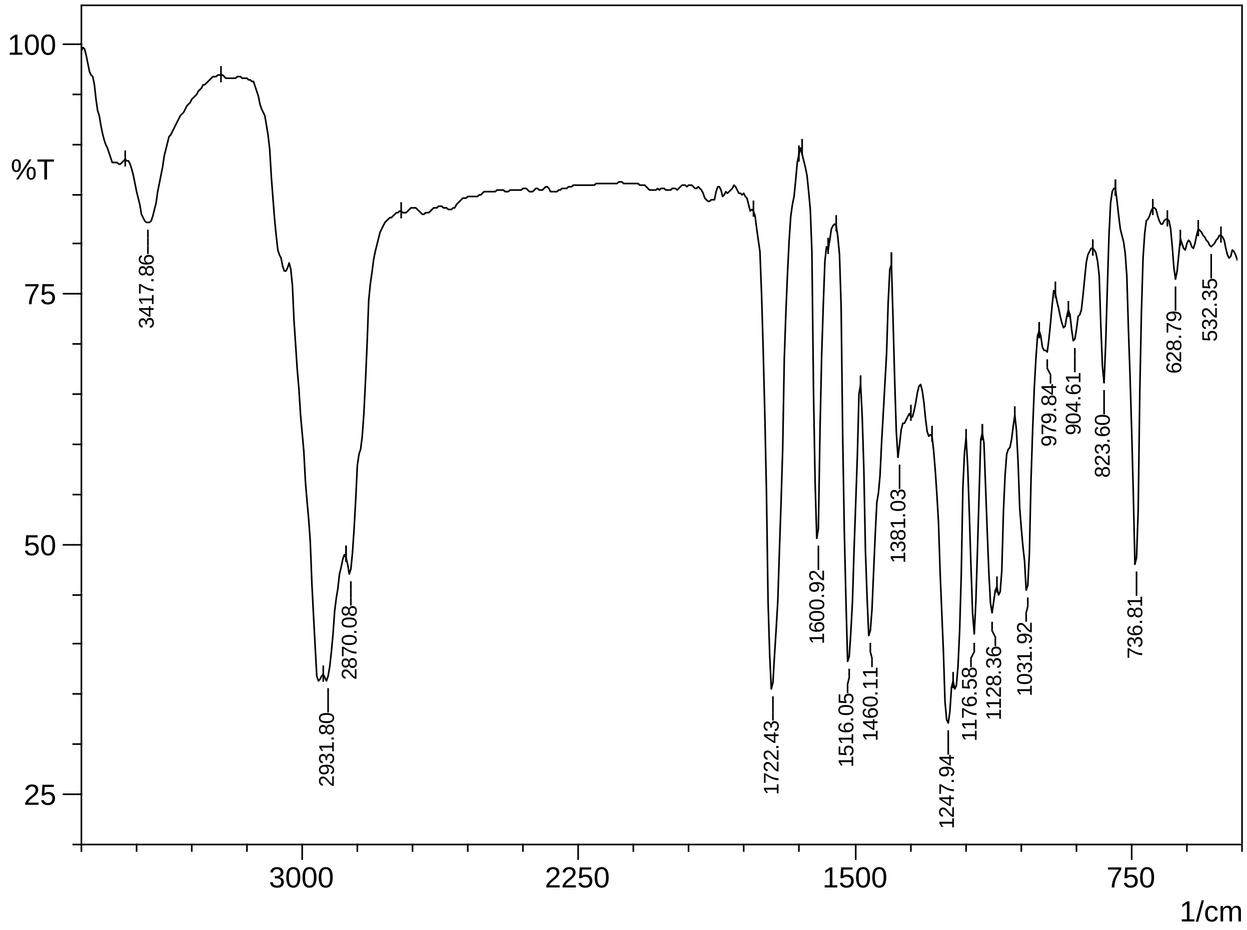

Figure S17. FTIR spectrum of the target compound (**3e**)

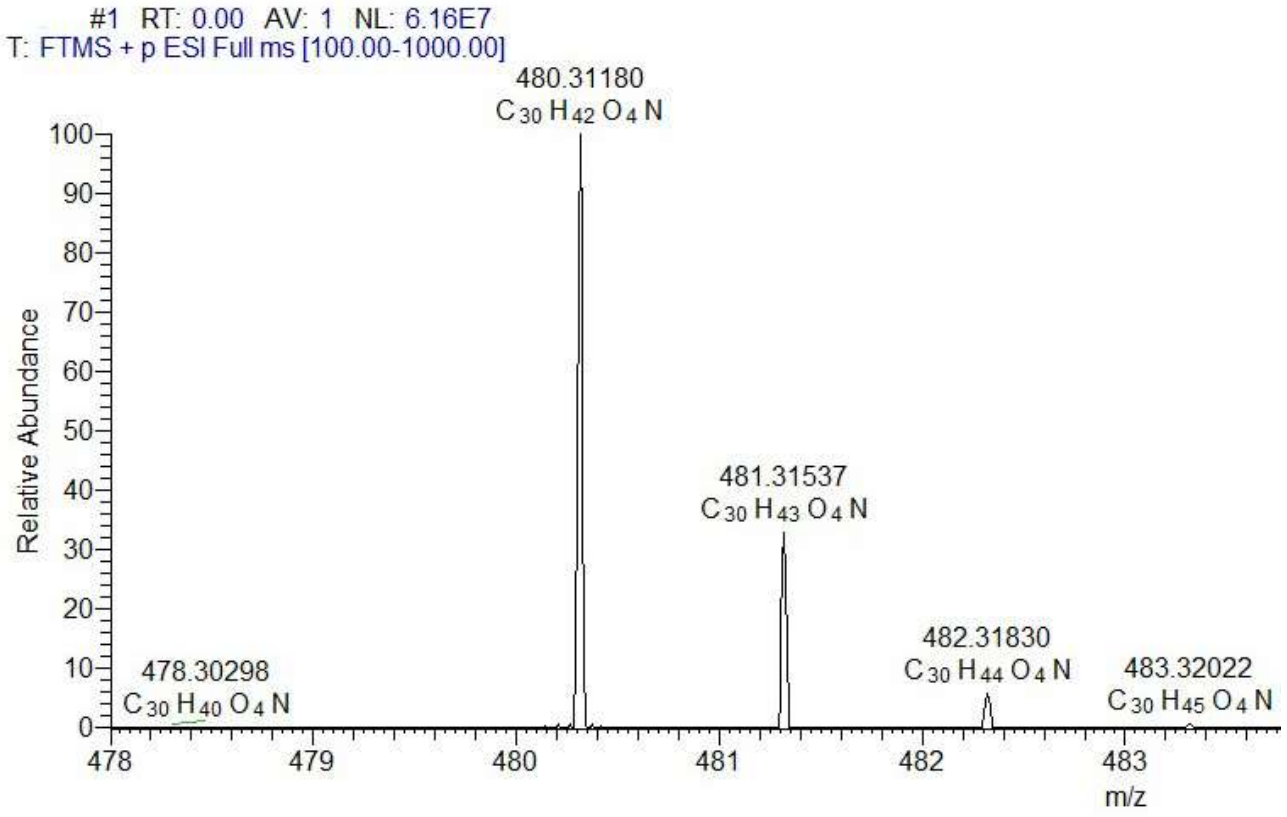

Figure S18. HRMS-ESI spectrum of the target compound (**3e**)

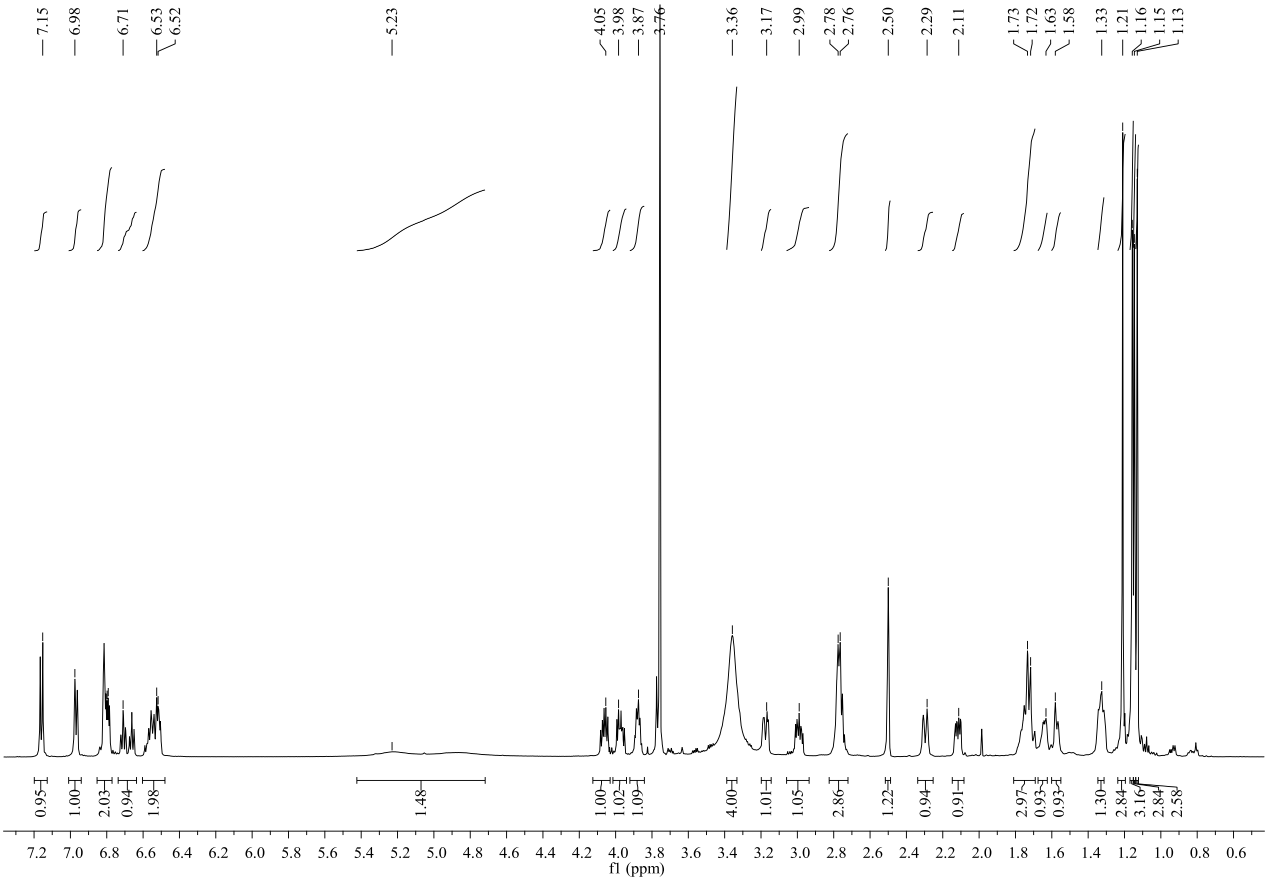

Figure S19. ^{1}H-NMR spectrum of the target compound (**3e**) in DMSO

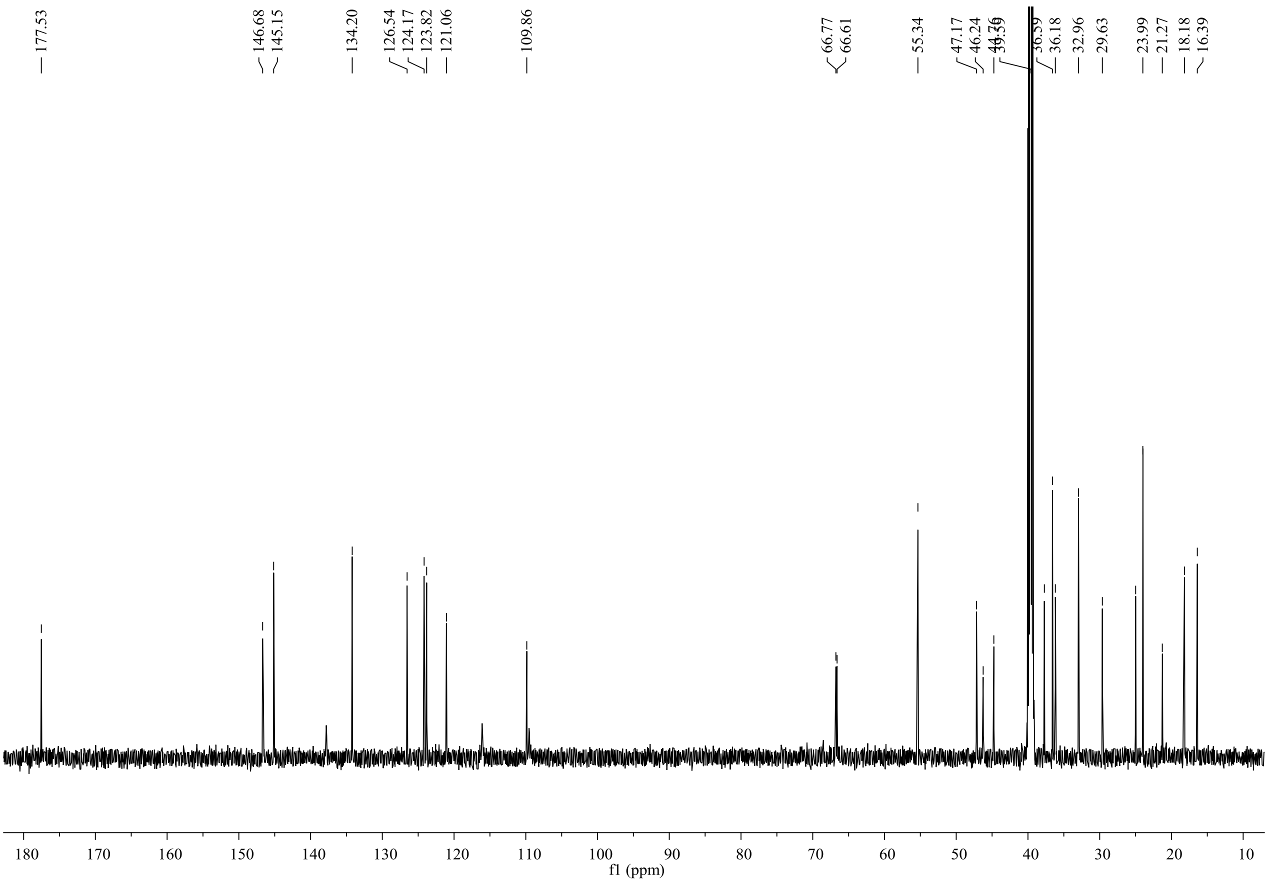

Figure S20. ^{13}C-NMR spectrum of the target compound (**3e**) in DMSO

Int. J. Mol. Sci. 2018, 19, 3116; doi:10.3390/ijms19103116

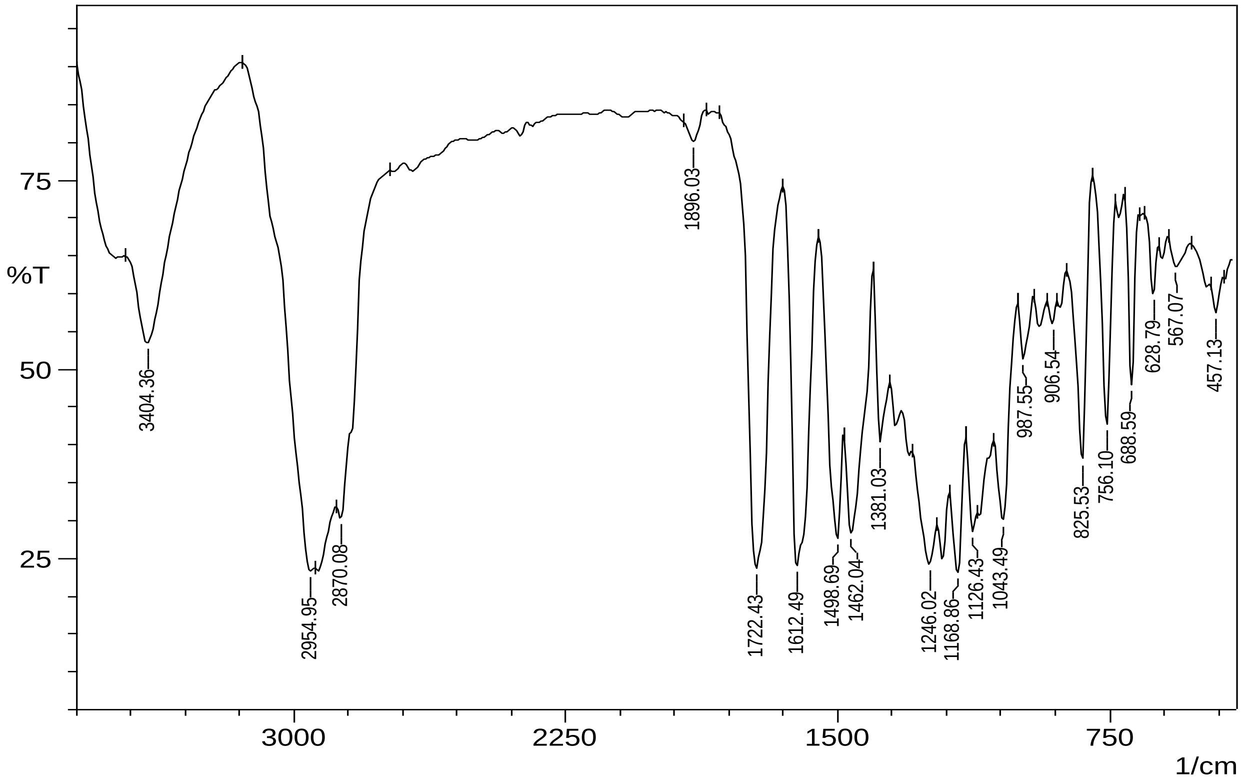

Figure S21. FTIR spectrum of the target compound (**3f**)

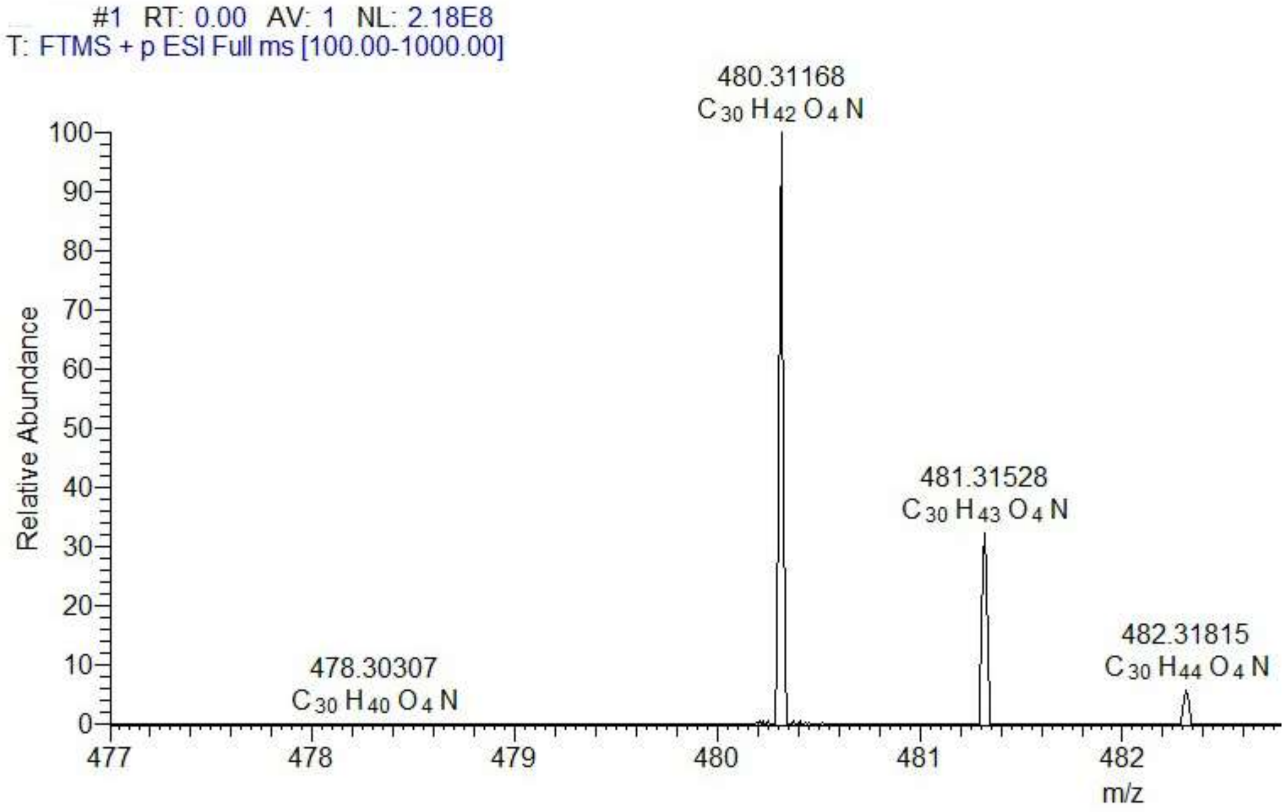

Figure S22. HRMS-ESI spectrum of the target compound (**3f**)

Int. J. Mol. Sci. 2018, 19, 3116; doi:10.3390/ijms19103116

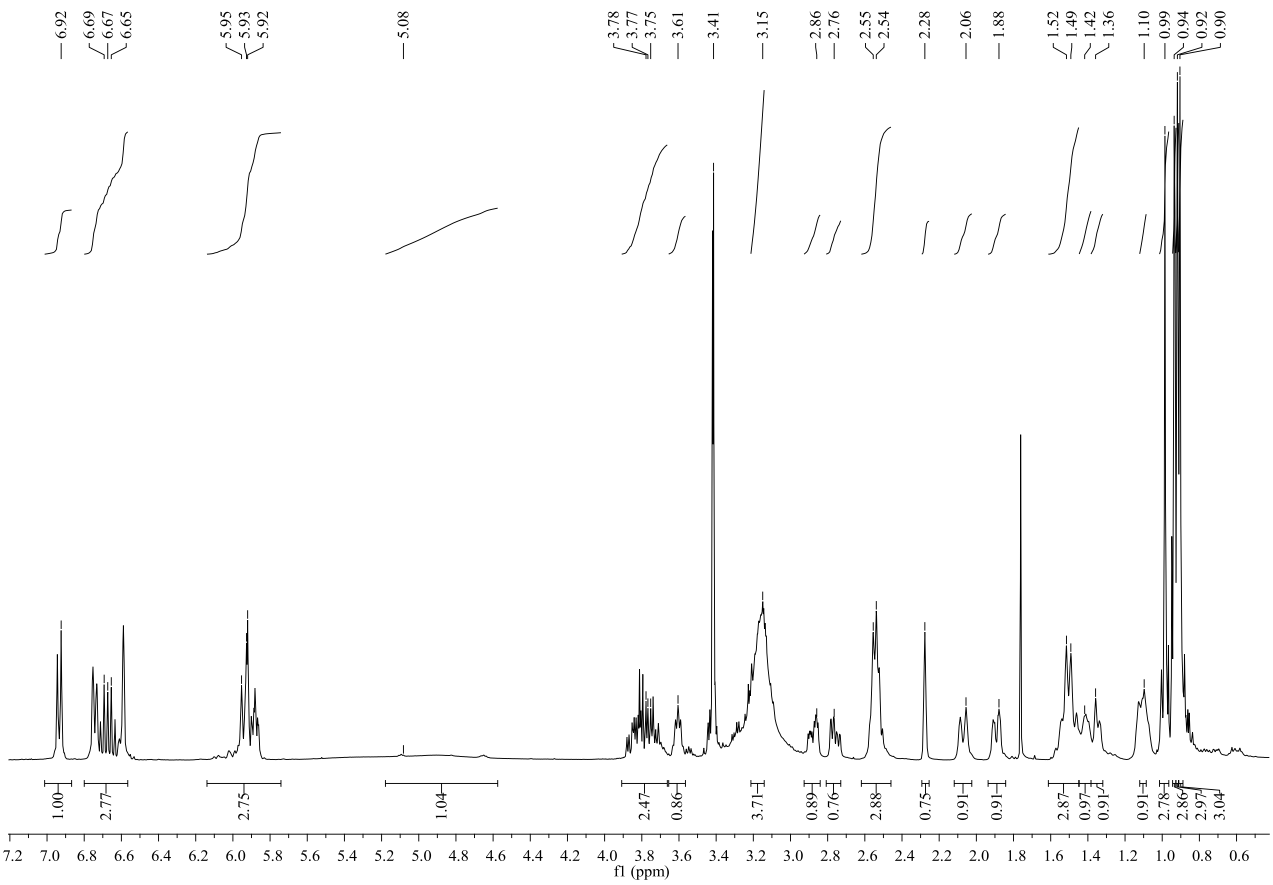

Figure S23. ^{1}H-NMR spectrum of the target compound (**3f**) in DMSO

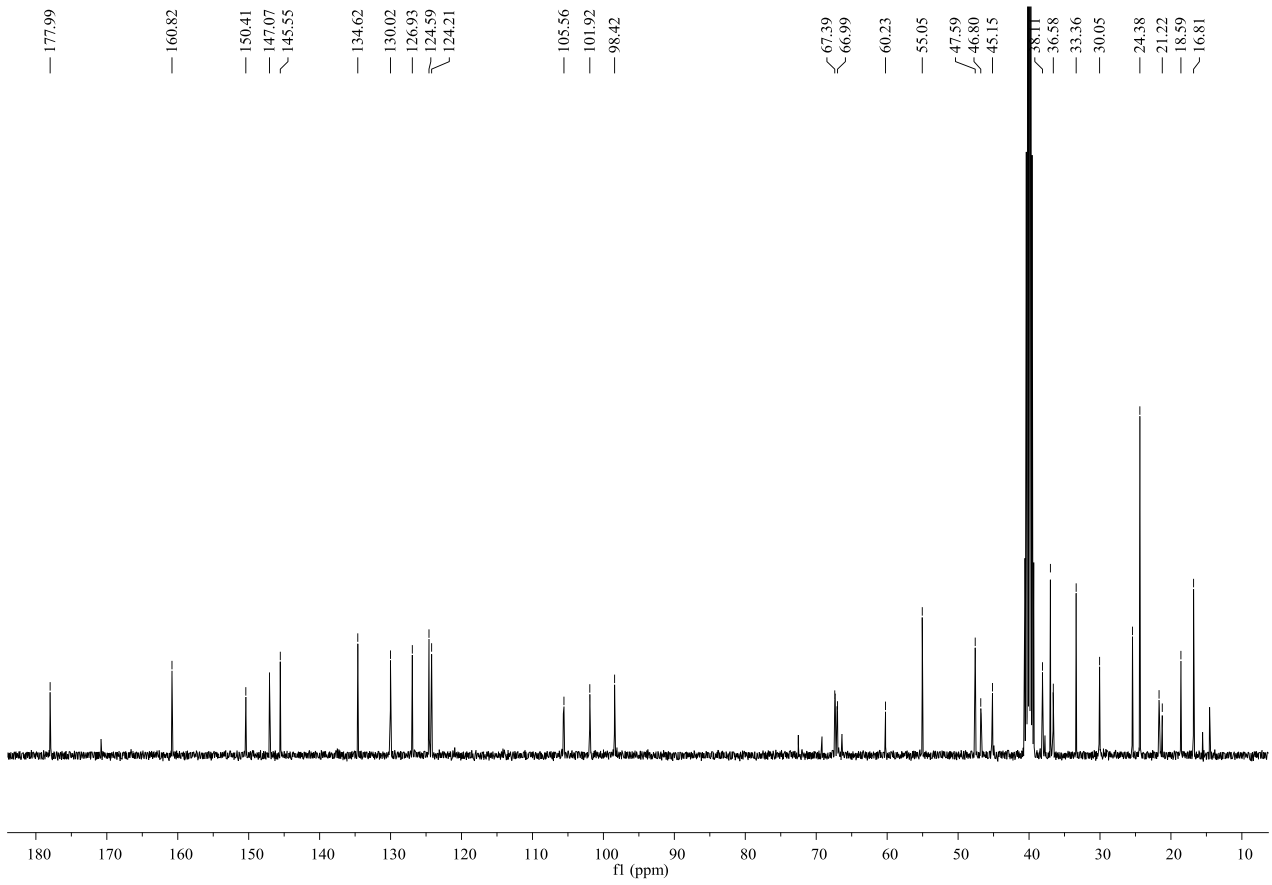

Figure S24. ^{13}C-NMR spectrum of the target compound (**3f**) in DMSO

Int. J. Mol. Sci. 2018, 19, 3116; doi:10.3390/ijms19103116

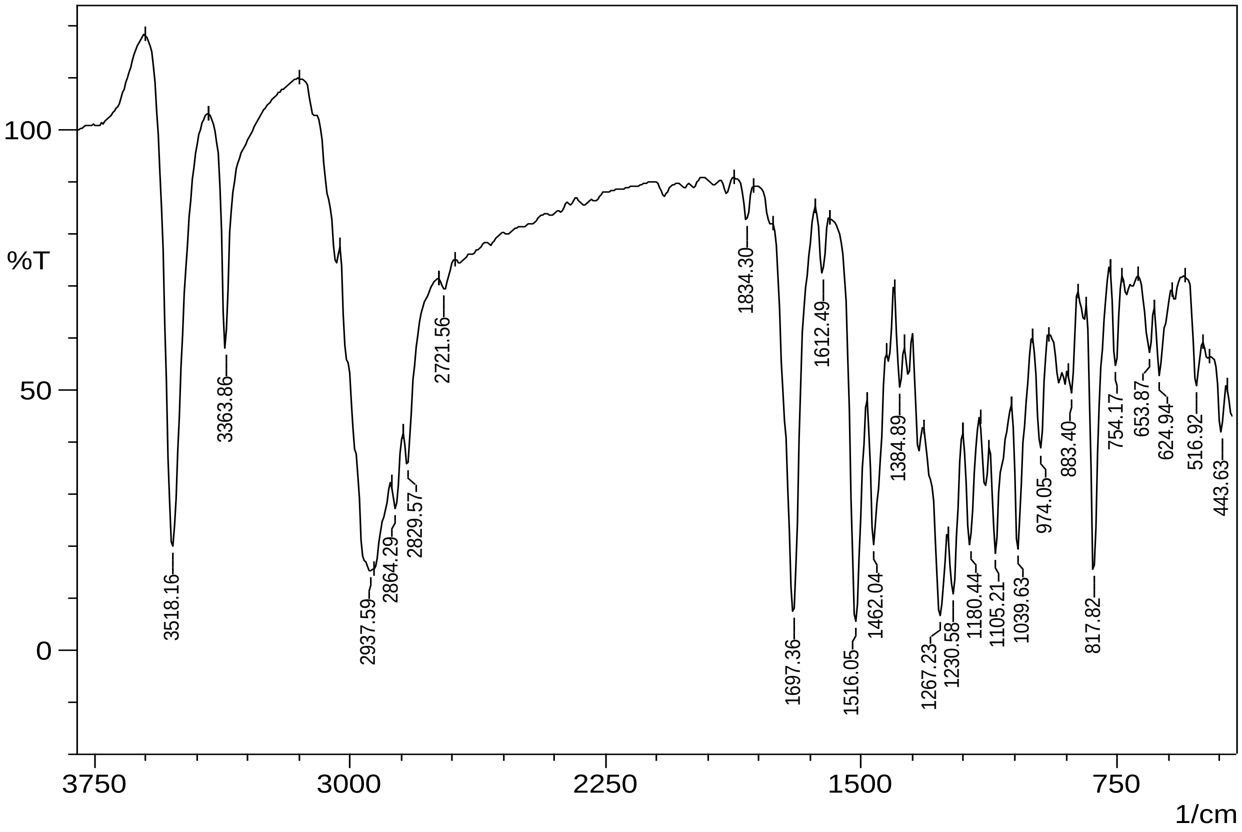

Figure S25. FTIR spectrum of the target compound (**3g**)

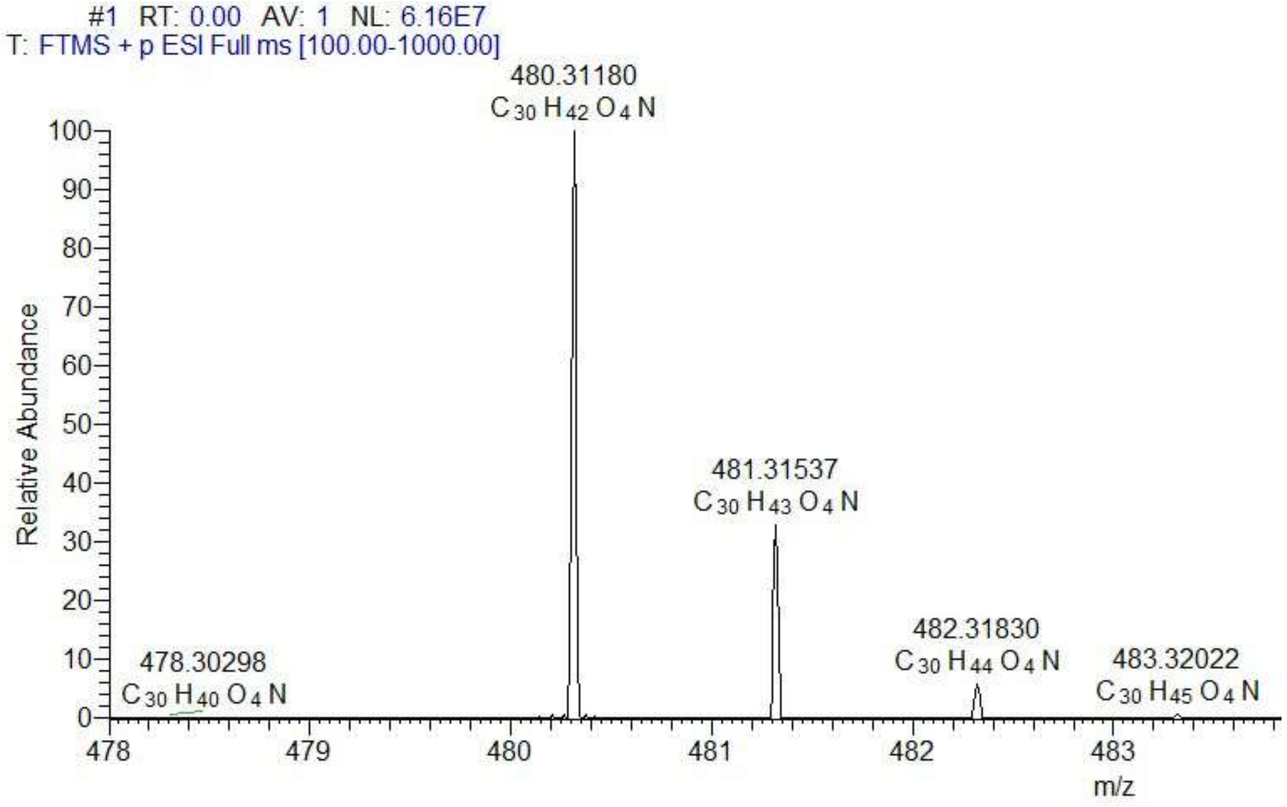

Figure S26. HRMS-ESI spectrum of the target compound (**3g**)

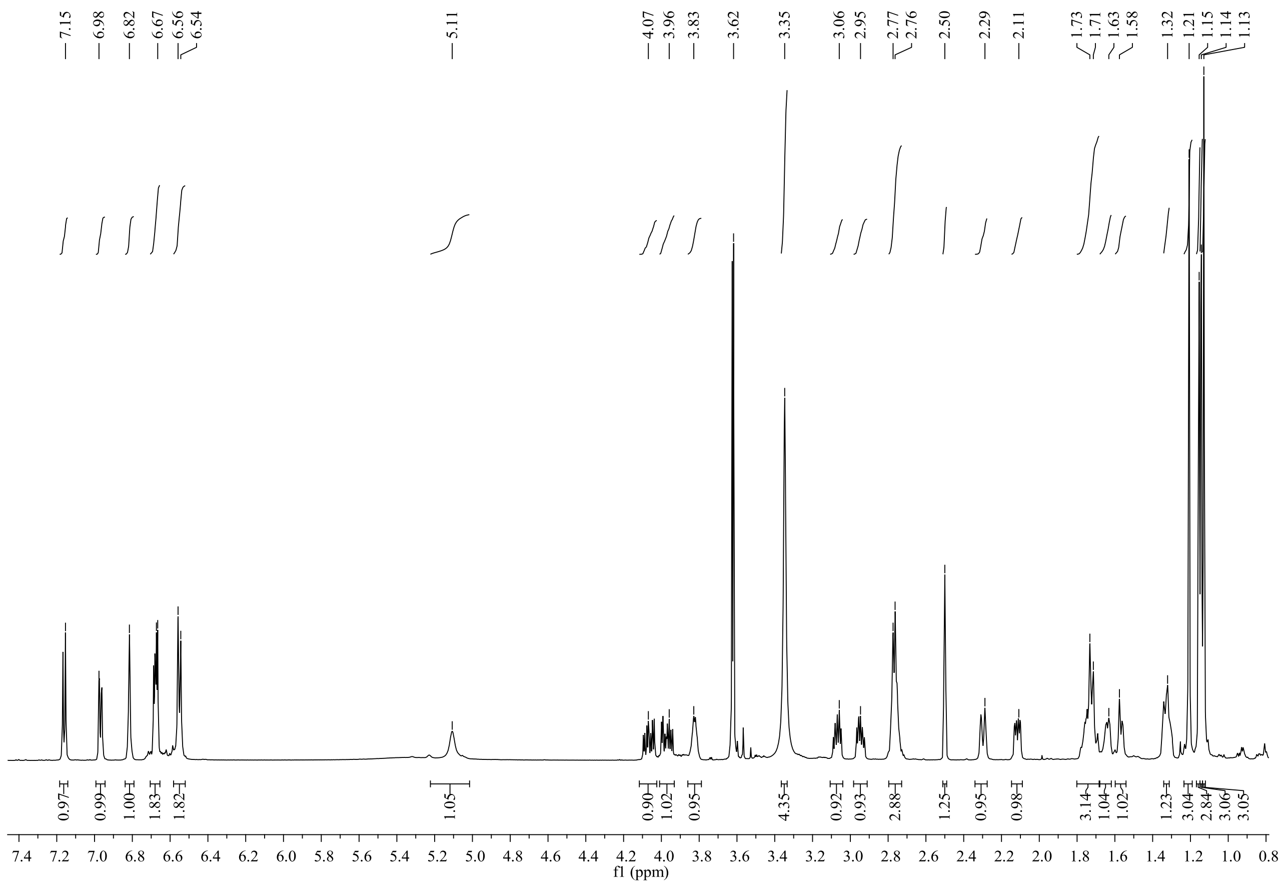

Figure S27. ^{1}H-NMR spectrum of the target compound (**3g**) in DMSO

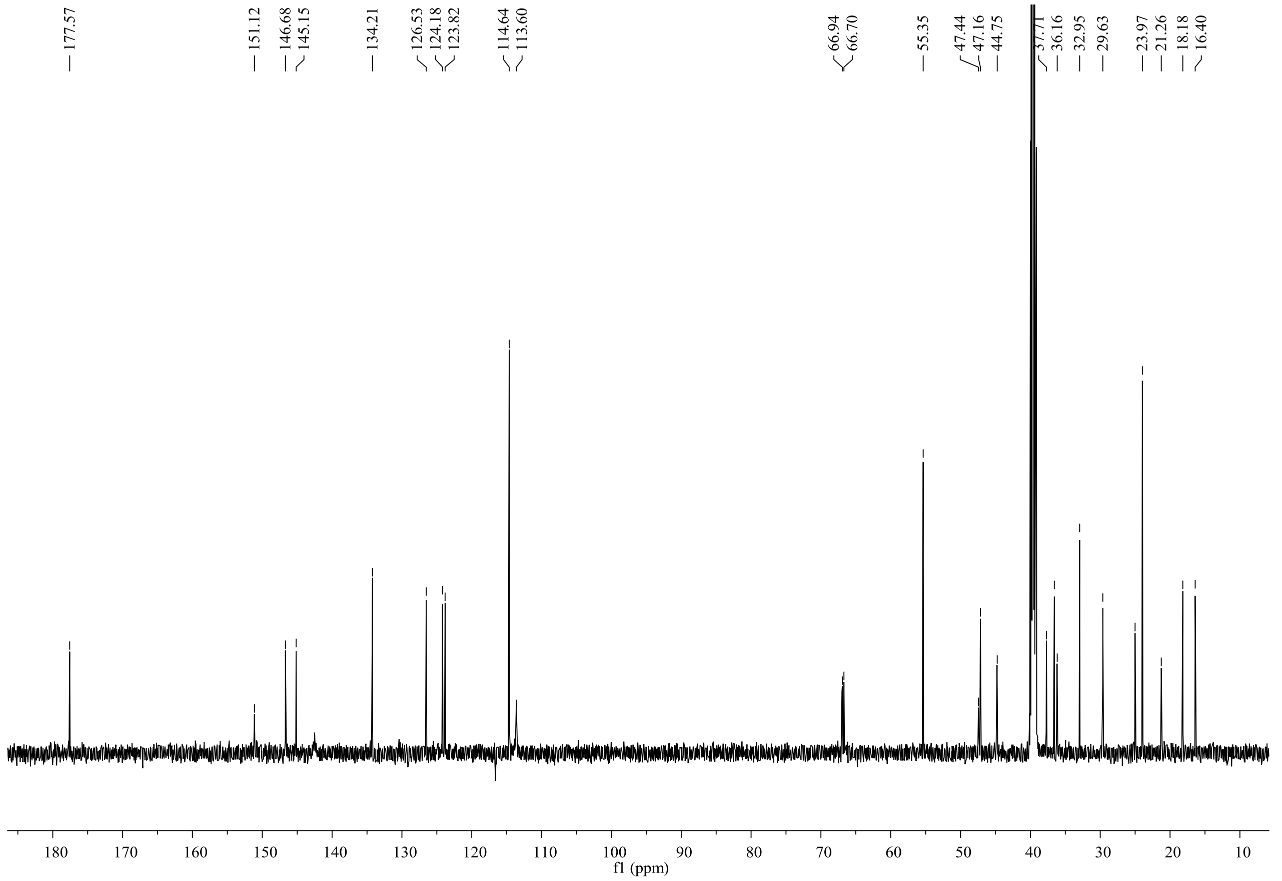

Figure S28. ^{13}C-NMR spectrum of the target compound (**3g**) in DMSO

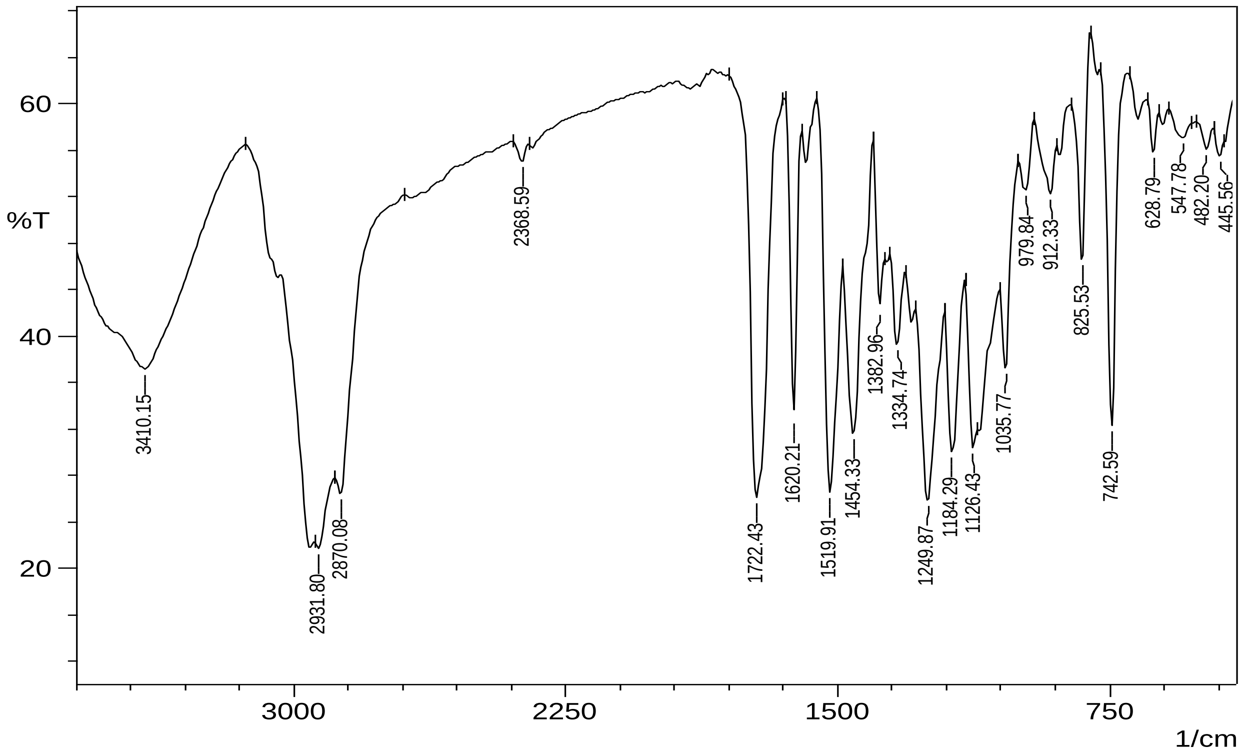

Figure S29. FTIR spectrum of the target compound (**3h**)

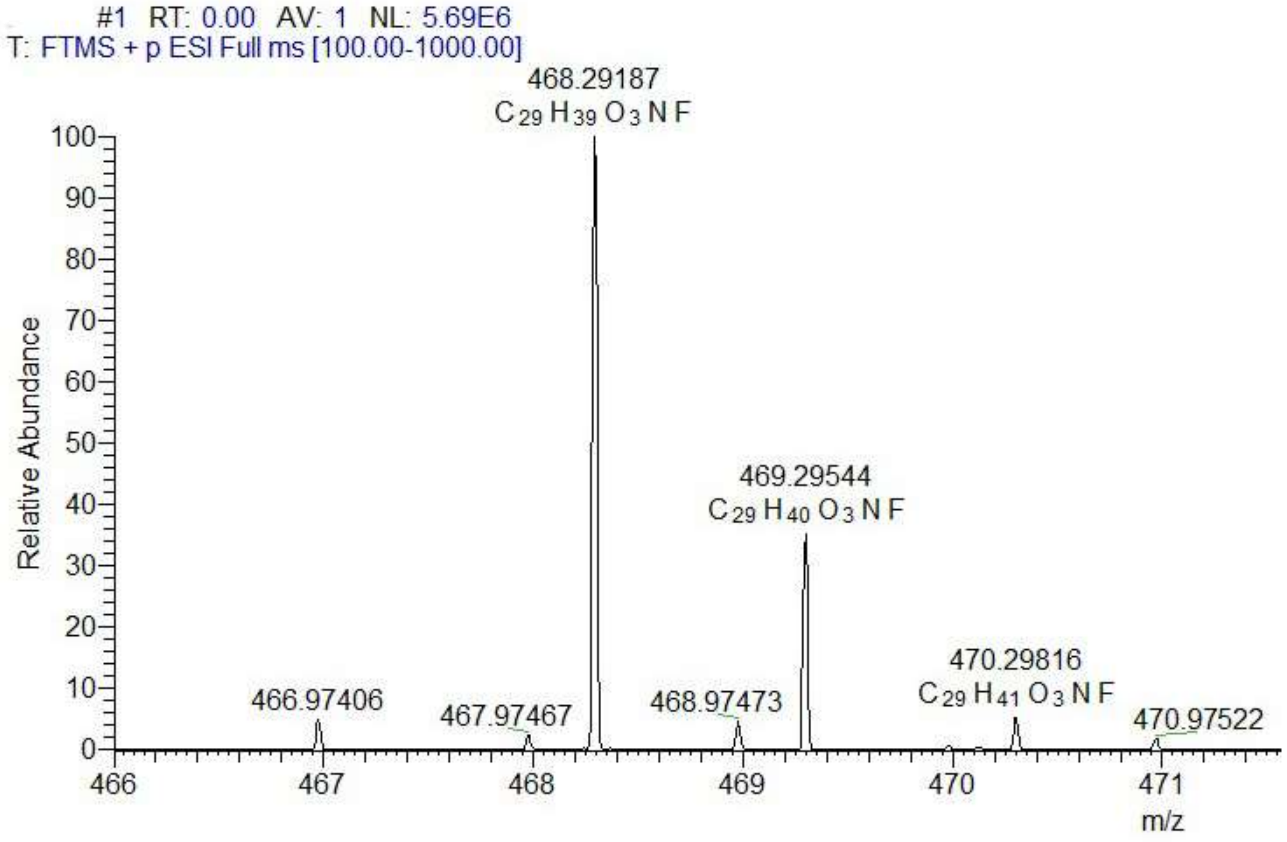

Figure S30. HRMS-ESI spectrum of the target compound (**3h**)

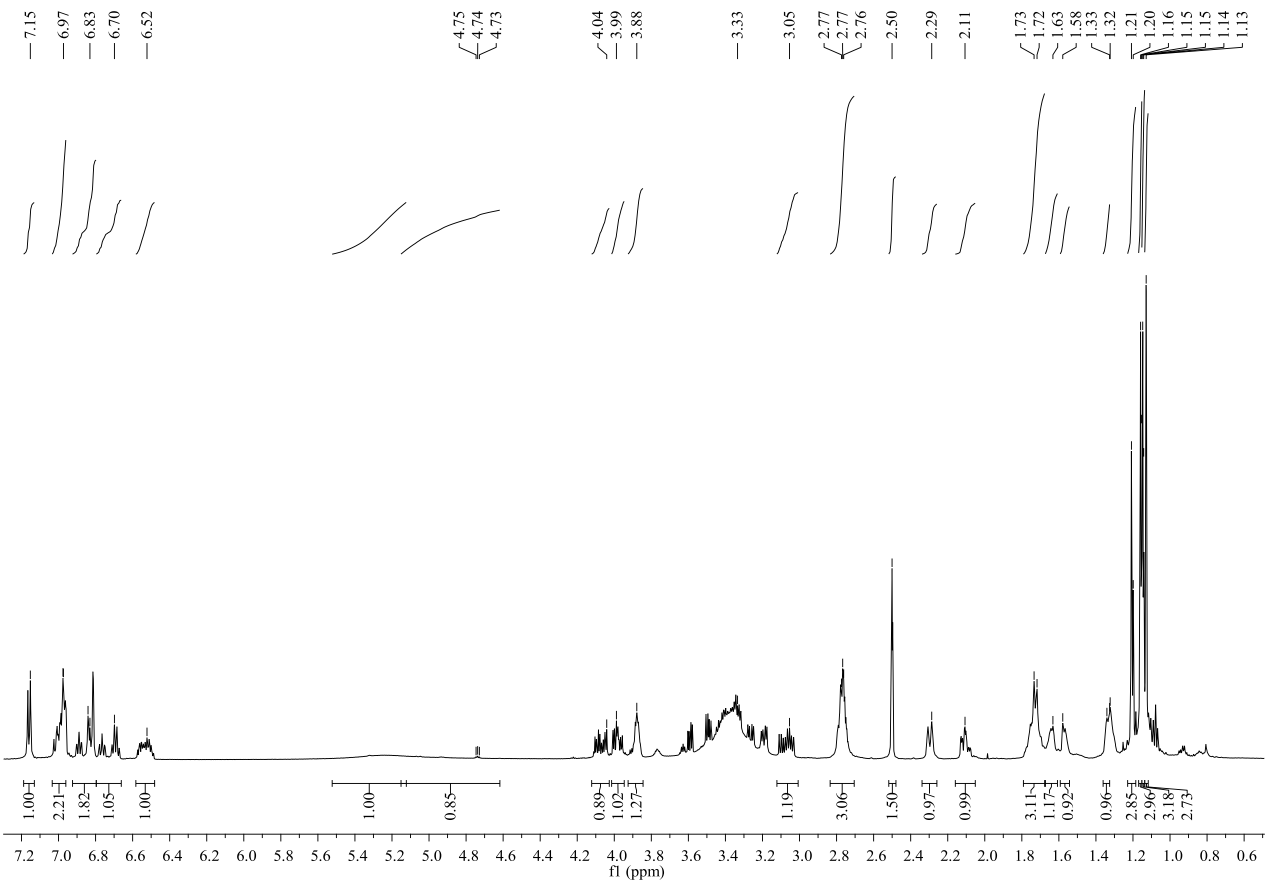

Figure S31. ^{1}H-NMR spectrum of the target compound (**3h**) in DMSO

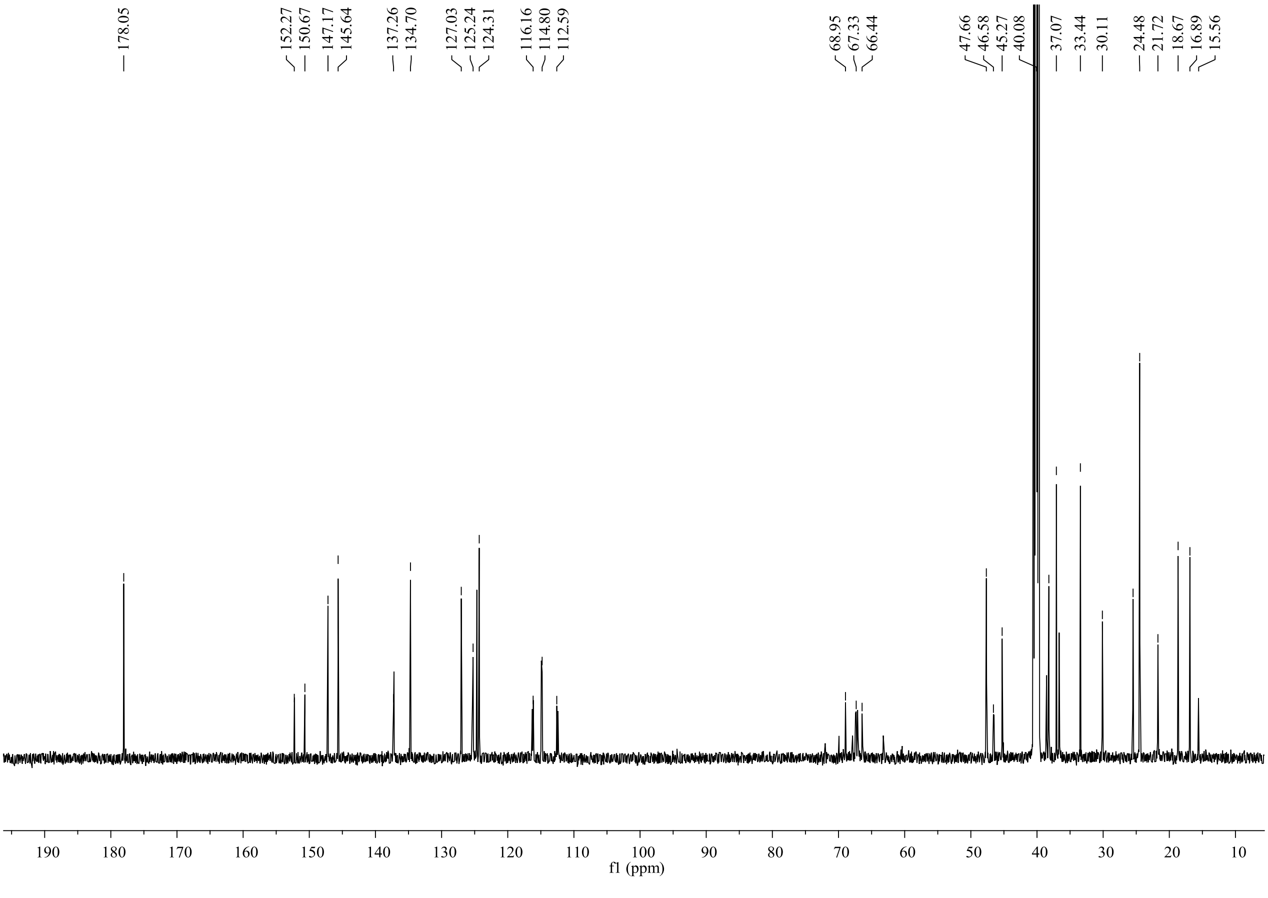

Figure S32. ^{13}C-NMR spectrum of the target compound (**3h**) in DMSO

Int. J. Mol. Sci. 2018, 19, 3116; doi:10.3390/ijms19103116

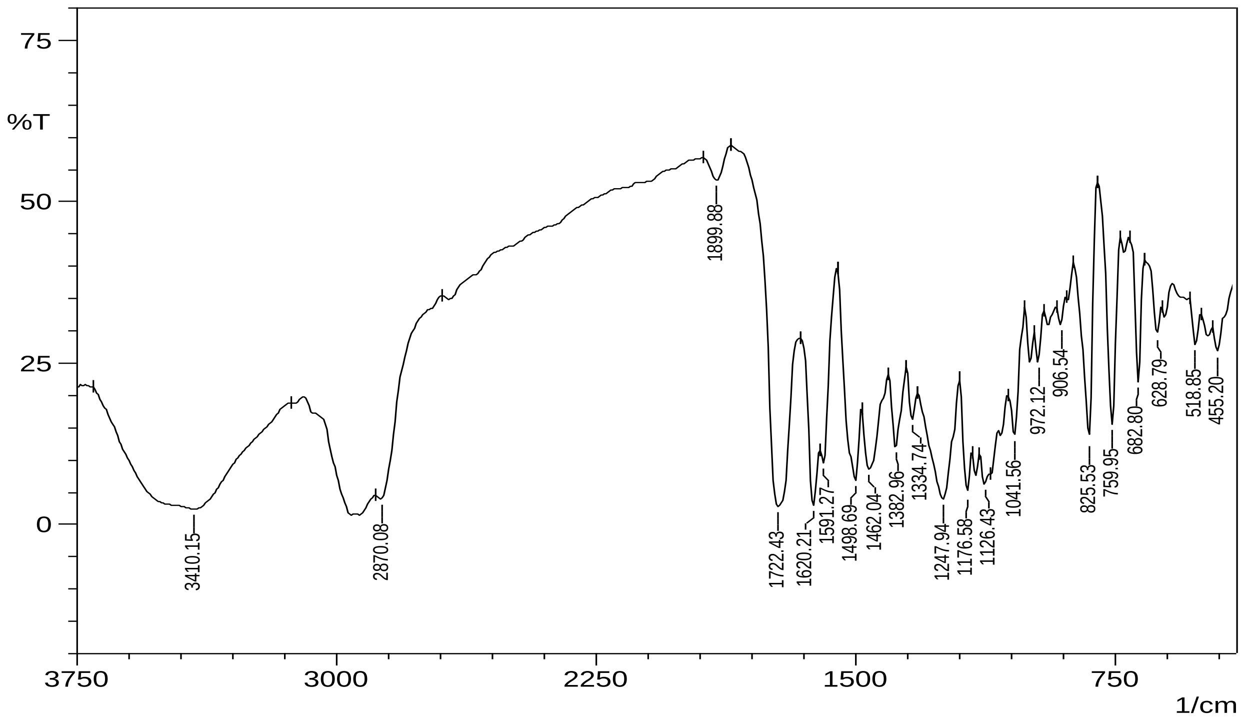

Figure S33. FTIR spectrum of the target compound (**3i**)

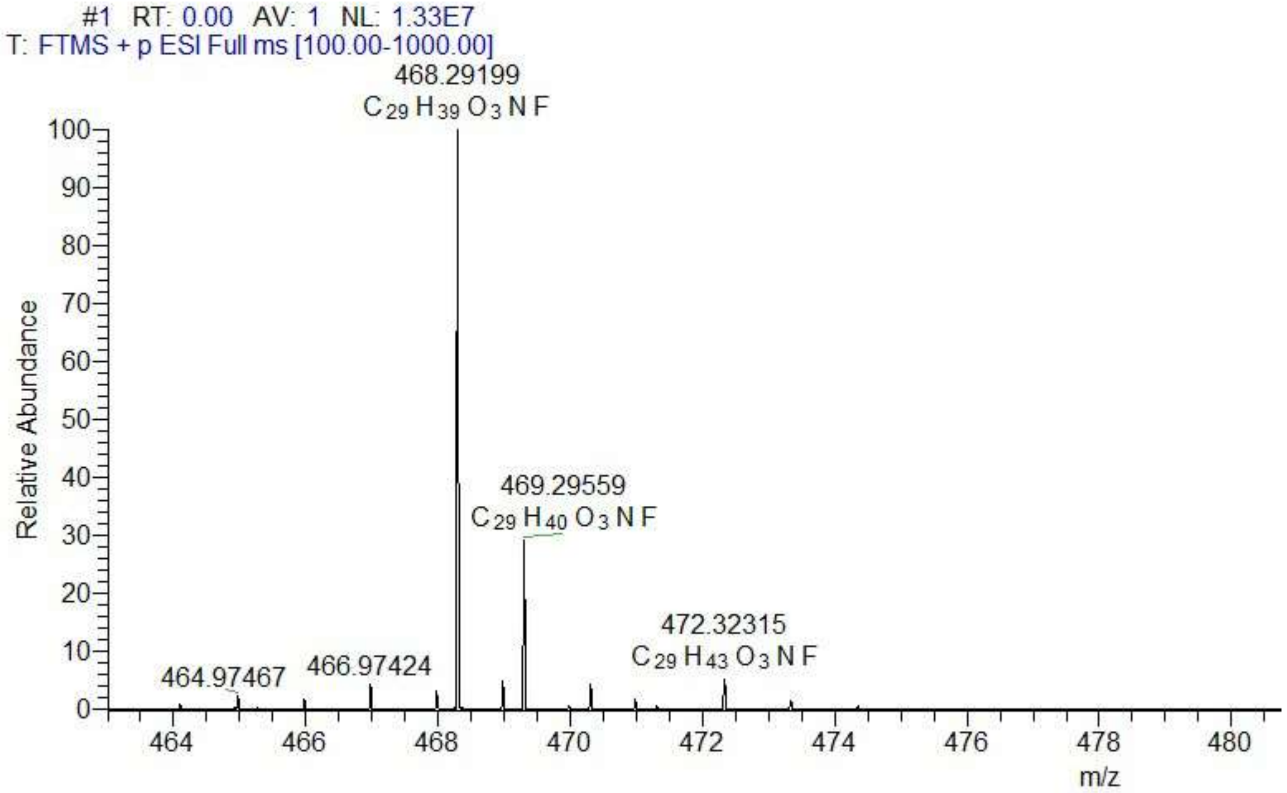

Figure S34. HRMS-ESI spectrum of the target compound (**3i**)

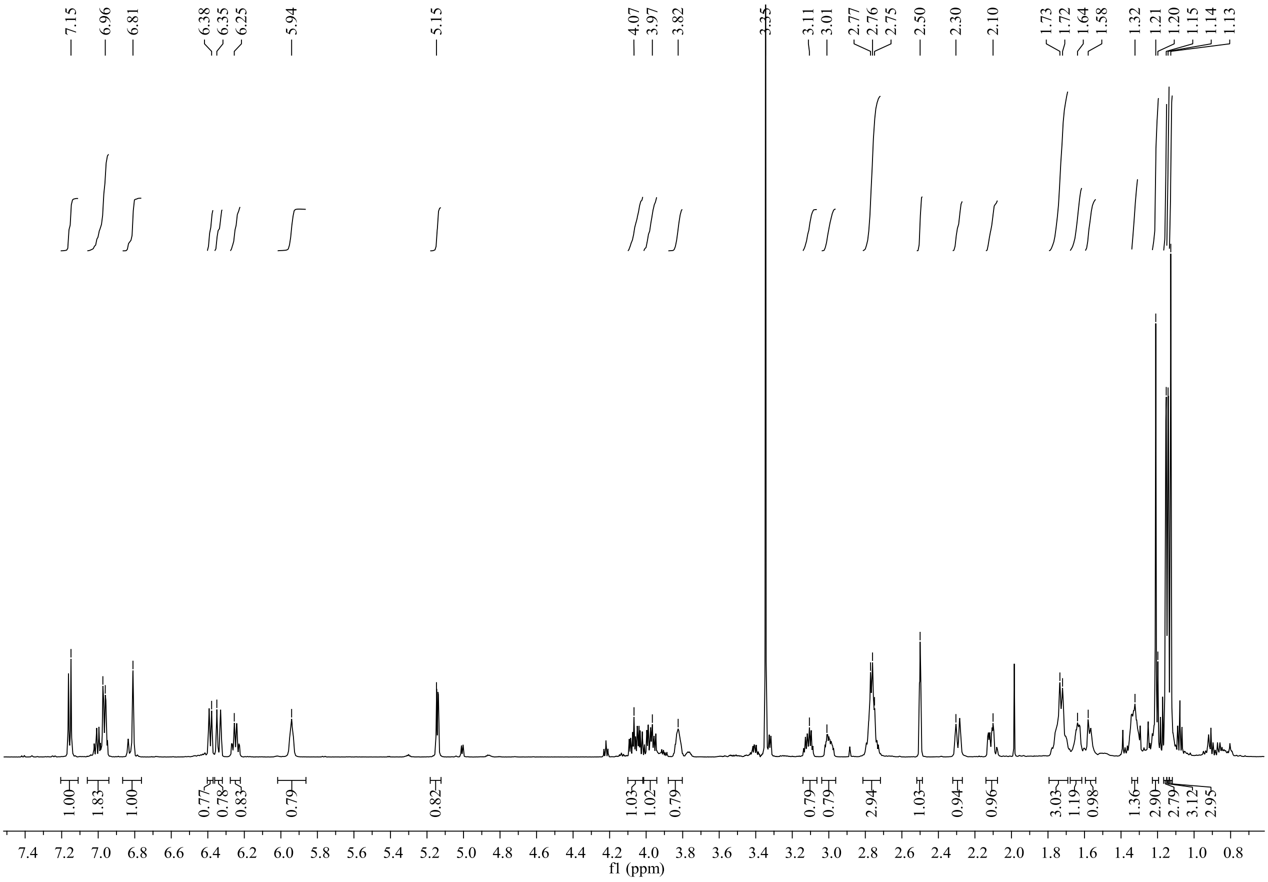

Figure S35. ^{1}H-NMR spectrum of the target compound (**3i**) in DMSO

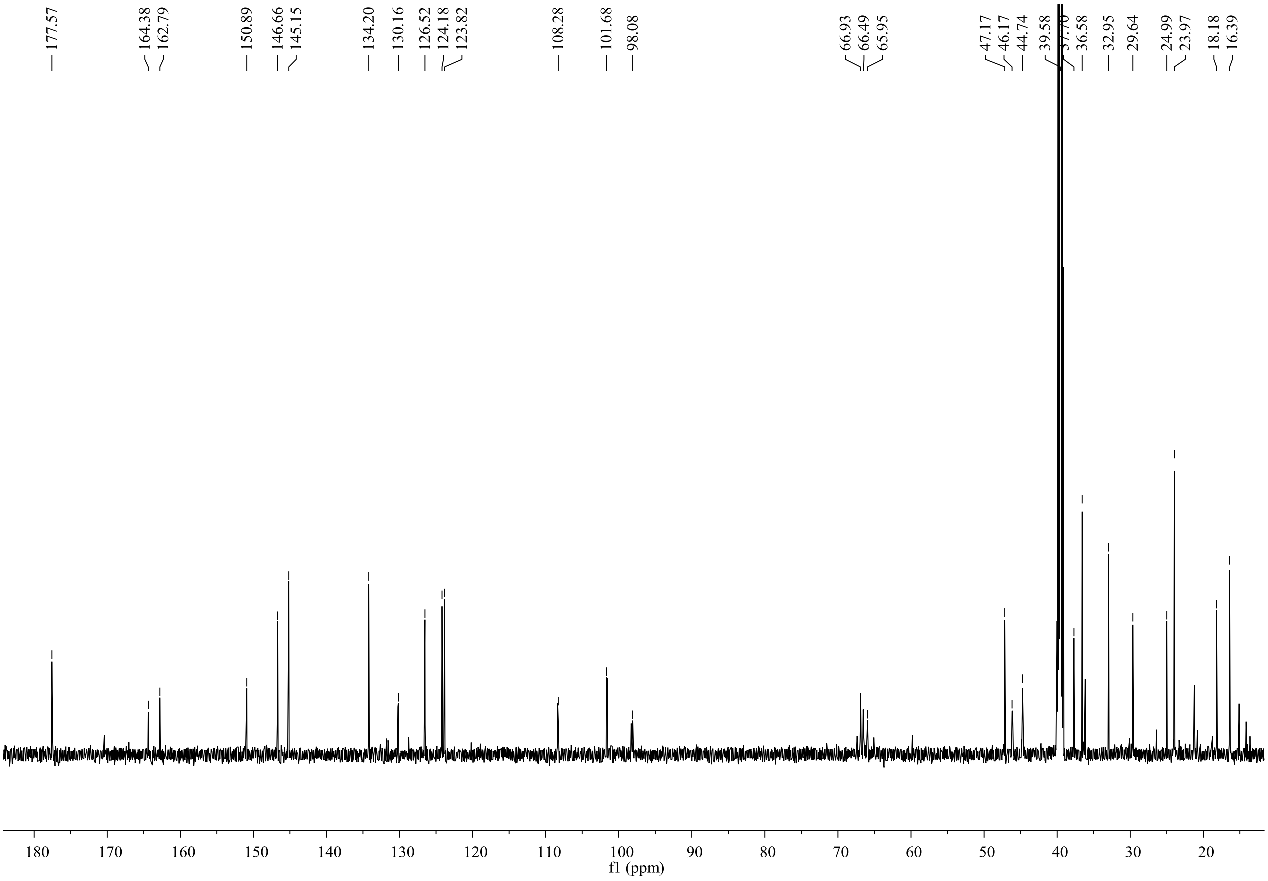

Figure S36. ^{13}C-NMR spectrum of the target compound (**3i**) in DMSO

Int. J. Mol. Sci. 2018, 19, 3116; doi:10.3390/ijms19103116

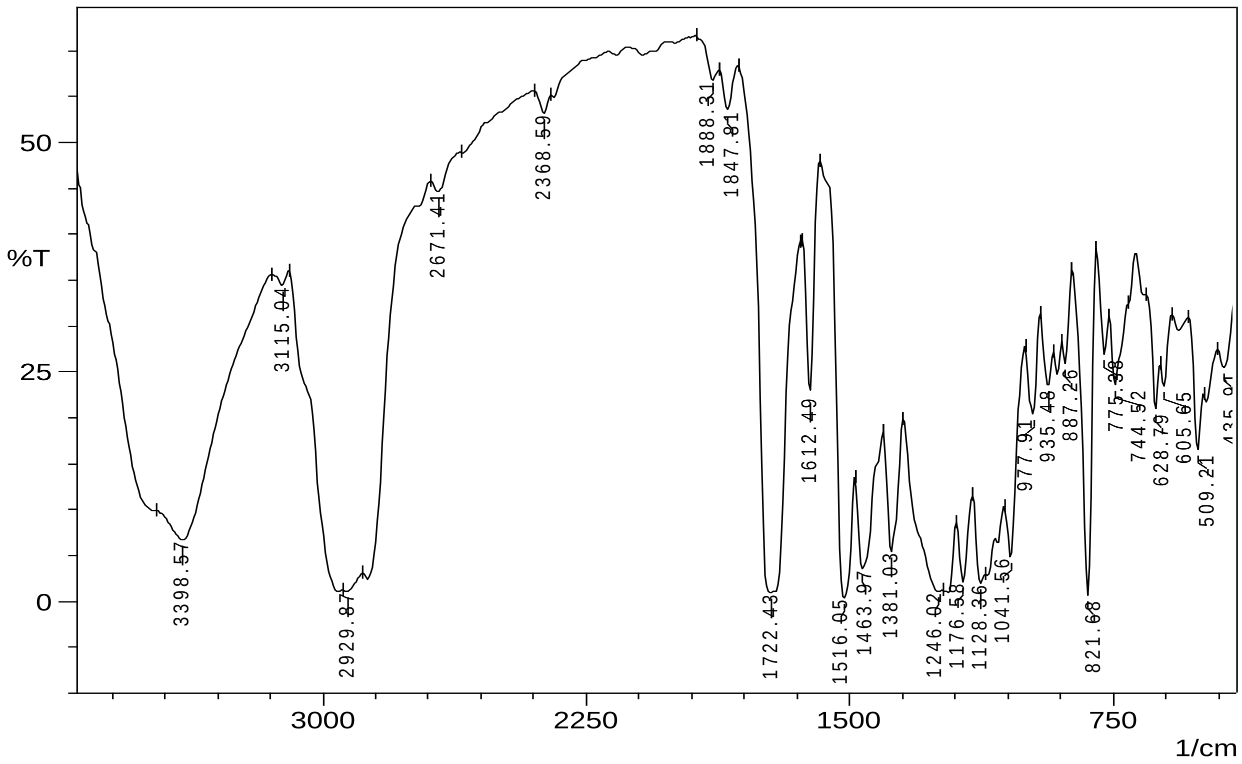

Figure S37. FTIR spectrum of the target compound (**3j**)

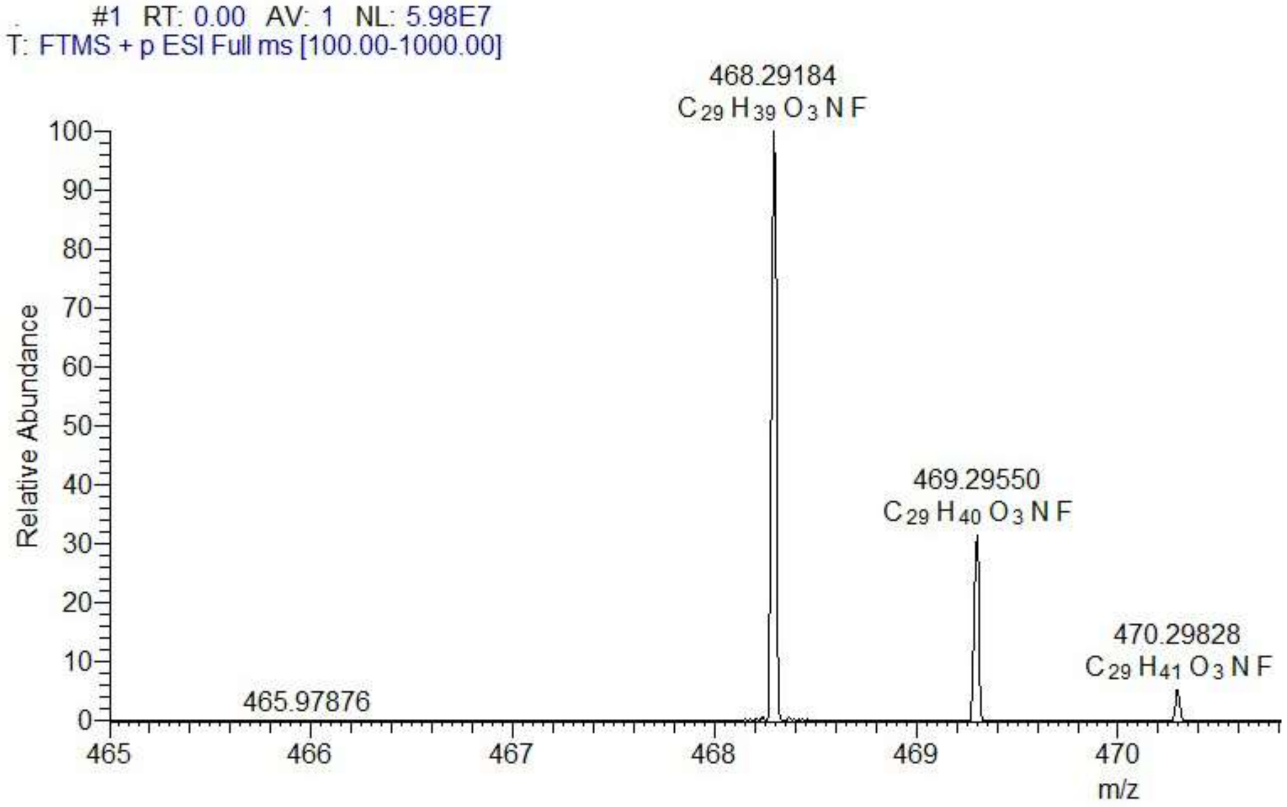

Figure S38. HRMS-ESI spectrum of the target compound (**3j**)

Int. J. Mol. Sci. 2018, 19, 3116; doi:10.3390/ijms19103116

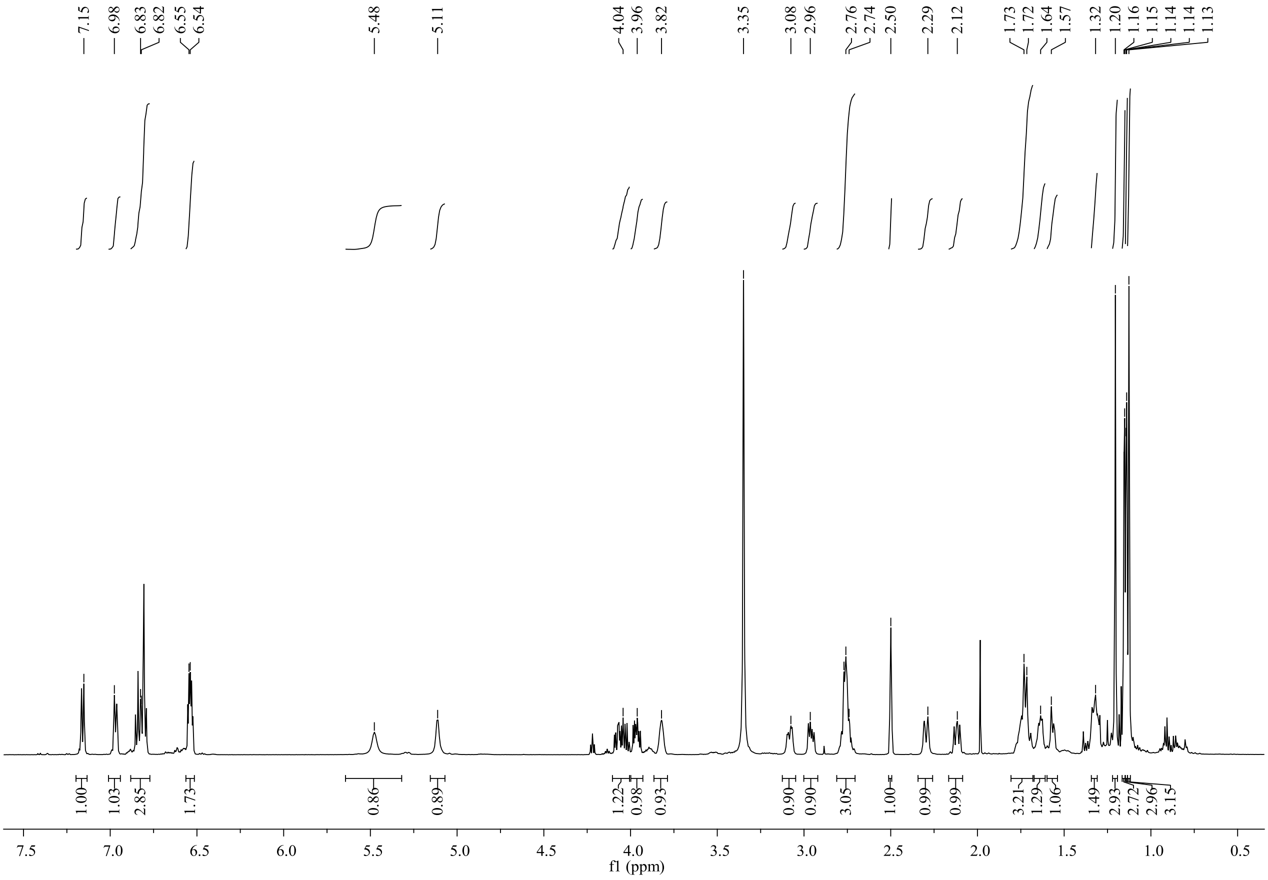

Figure S39. ^{1}H-NMR spectrum of the target compound (**3j**) in DMSO

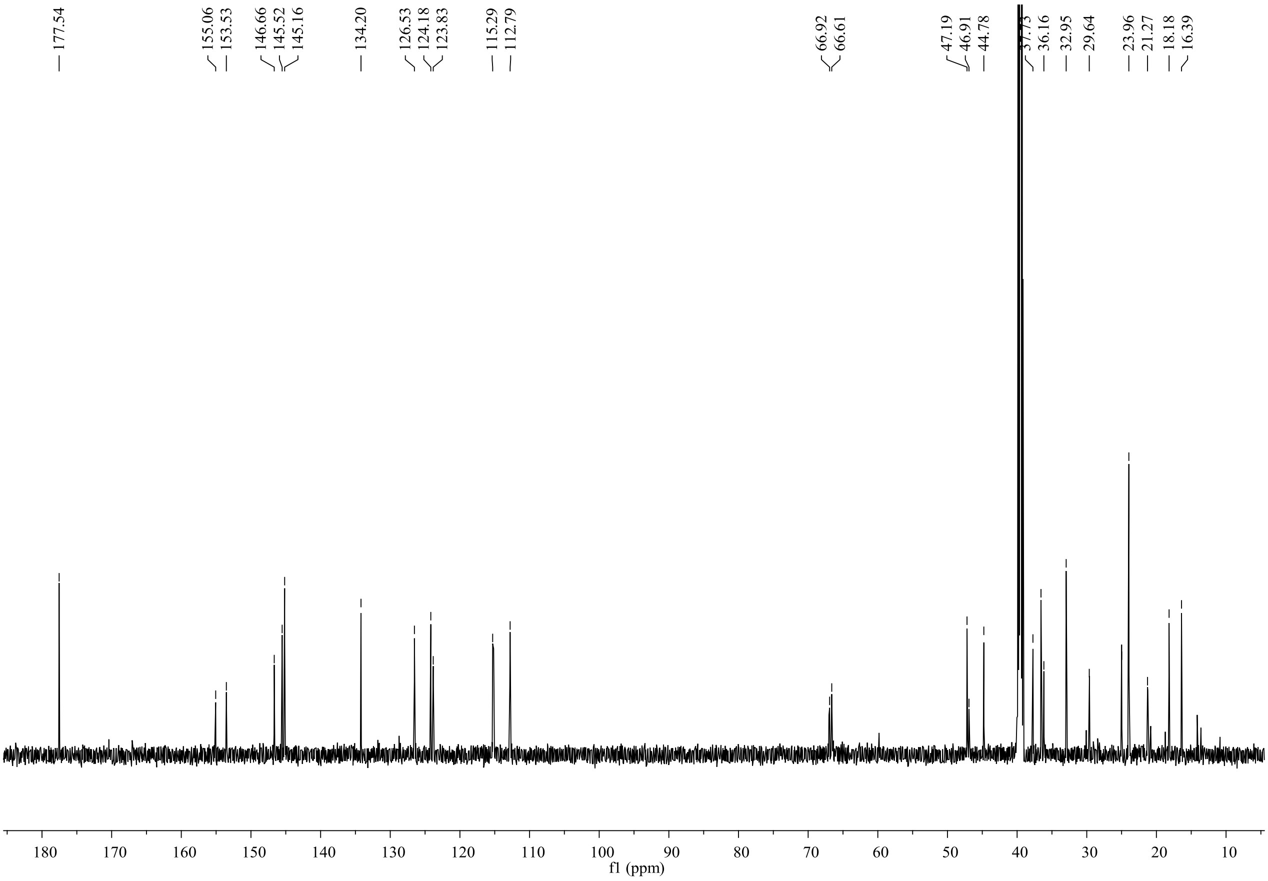

Figure S40. ^{13}C-NMR spectrum of the target compound (**3j**) in DMSO

Int. J. Mol. Sci. 2018, 19, 3116; doi:10.3390/ijms19103116

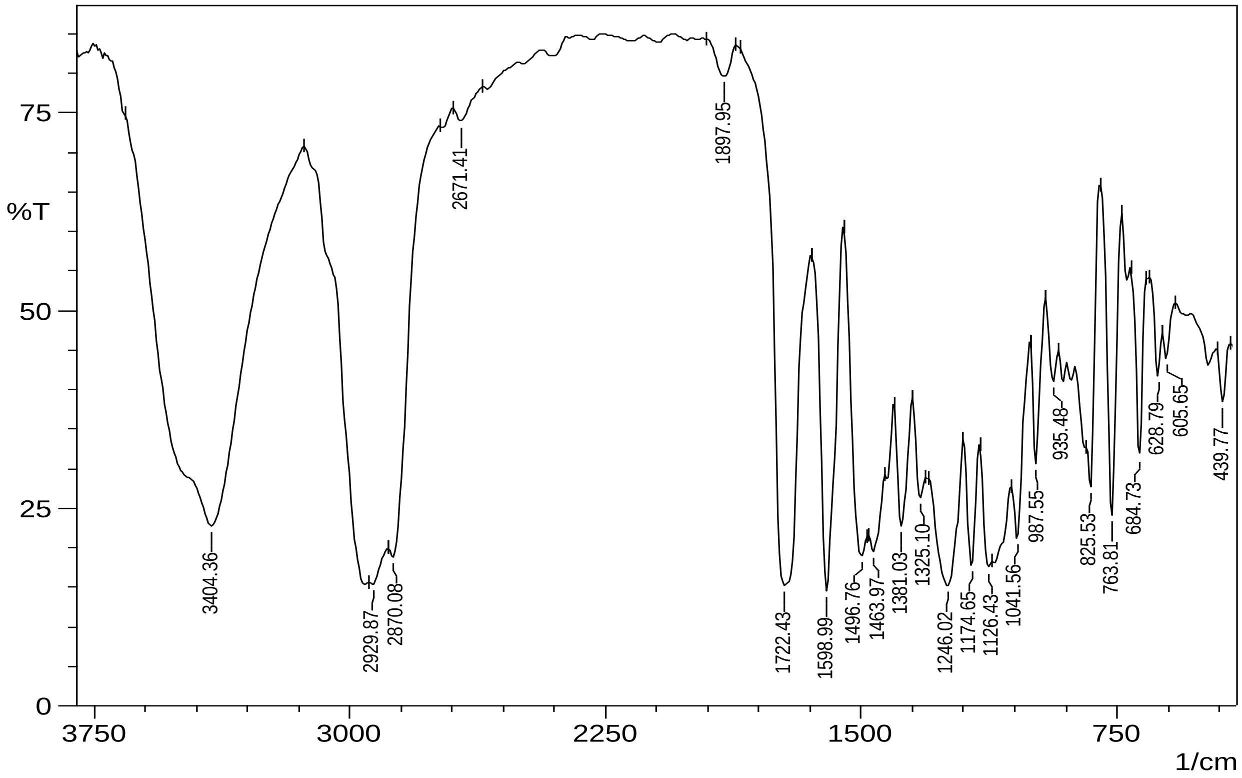

Figure S41. FTIR spectrum of the target compound (**3k**)

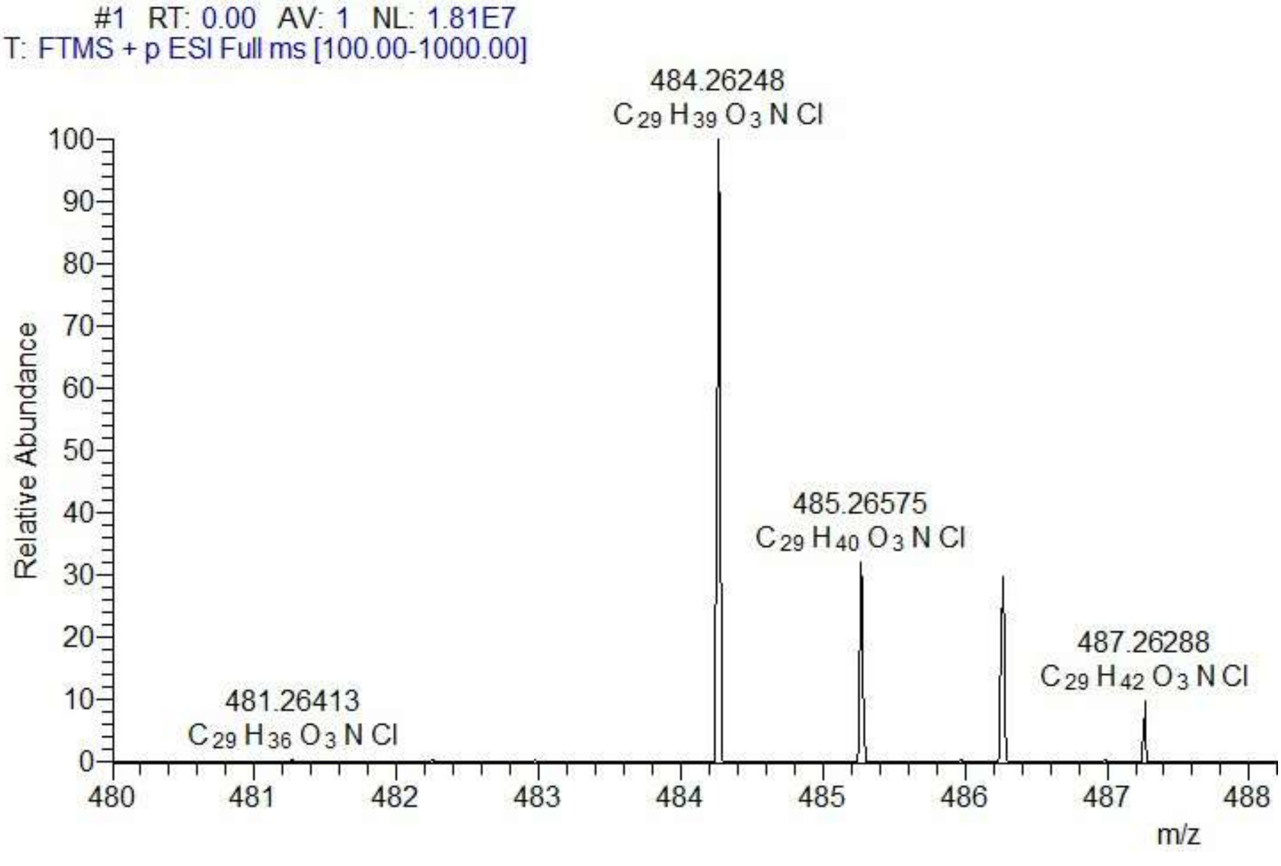

Figure S42. HRMS-ESI spectrum of the target compound (**3k**)

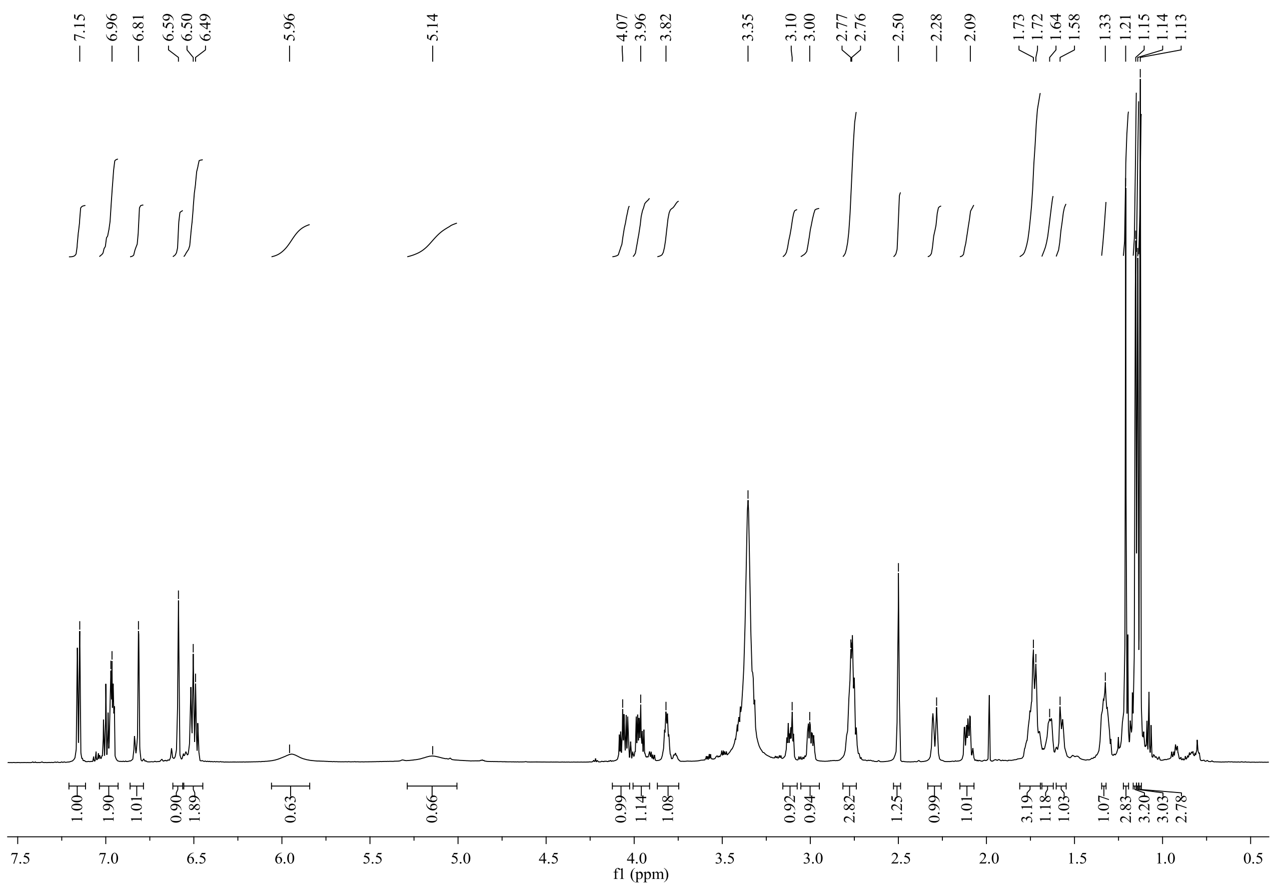

Figure S43. ^{1}H-NMR spectrum of the target compound (**3k**) in DMSO

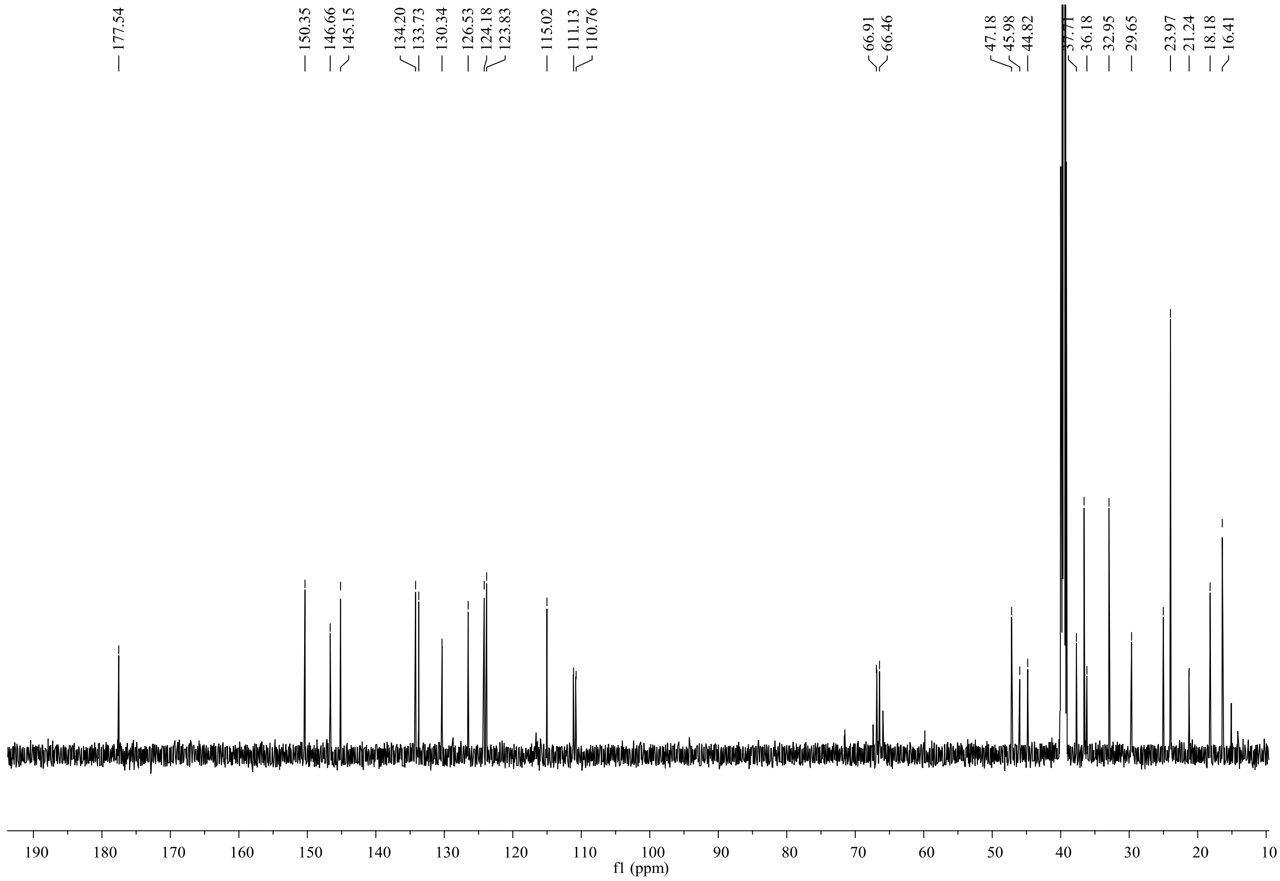

Figure S44. ^{13}C-NMR spectrum of the target compound (**3k**) in DMSO

Int. J. Mol. Sci. 2018, 19, 3116; doi:10.3390/ijms19103116

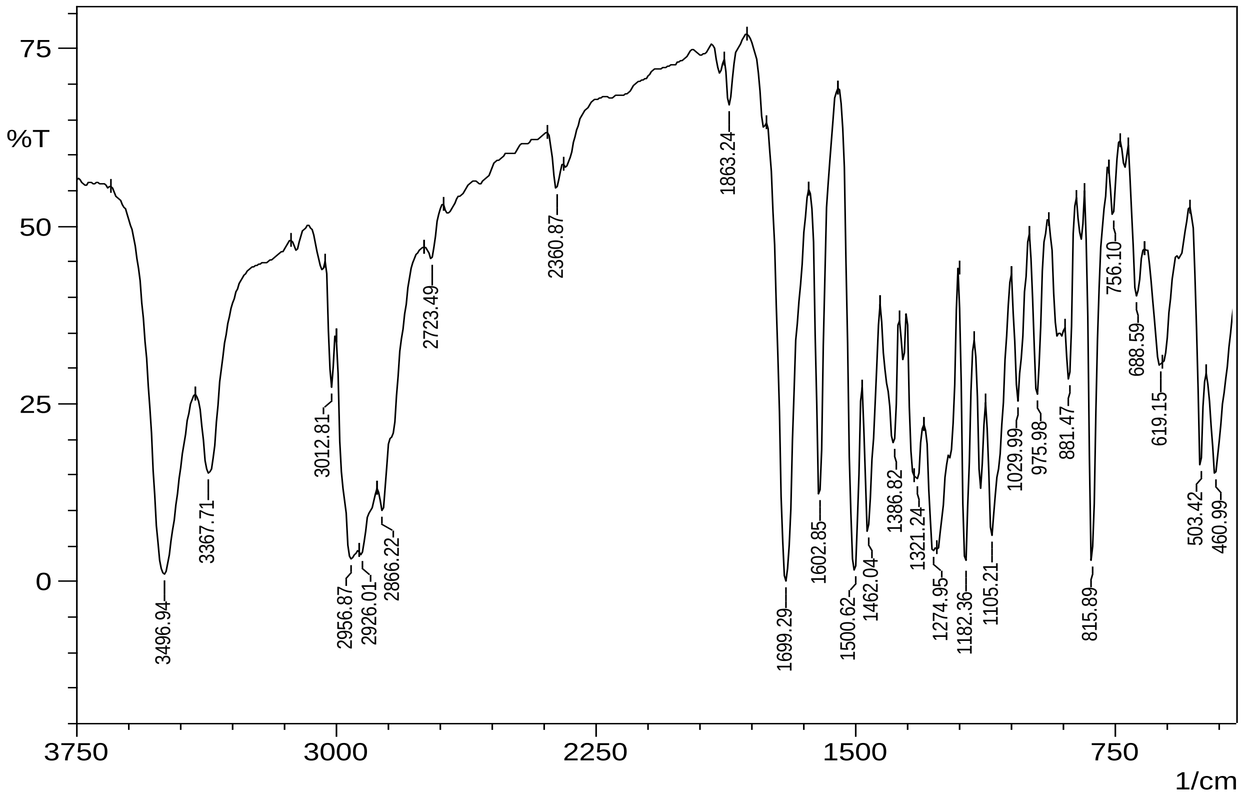

Figure S45. FTIR spectrum of the target compound (**3l**)

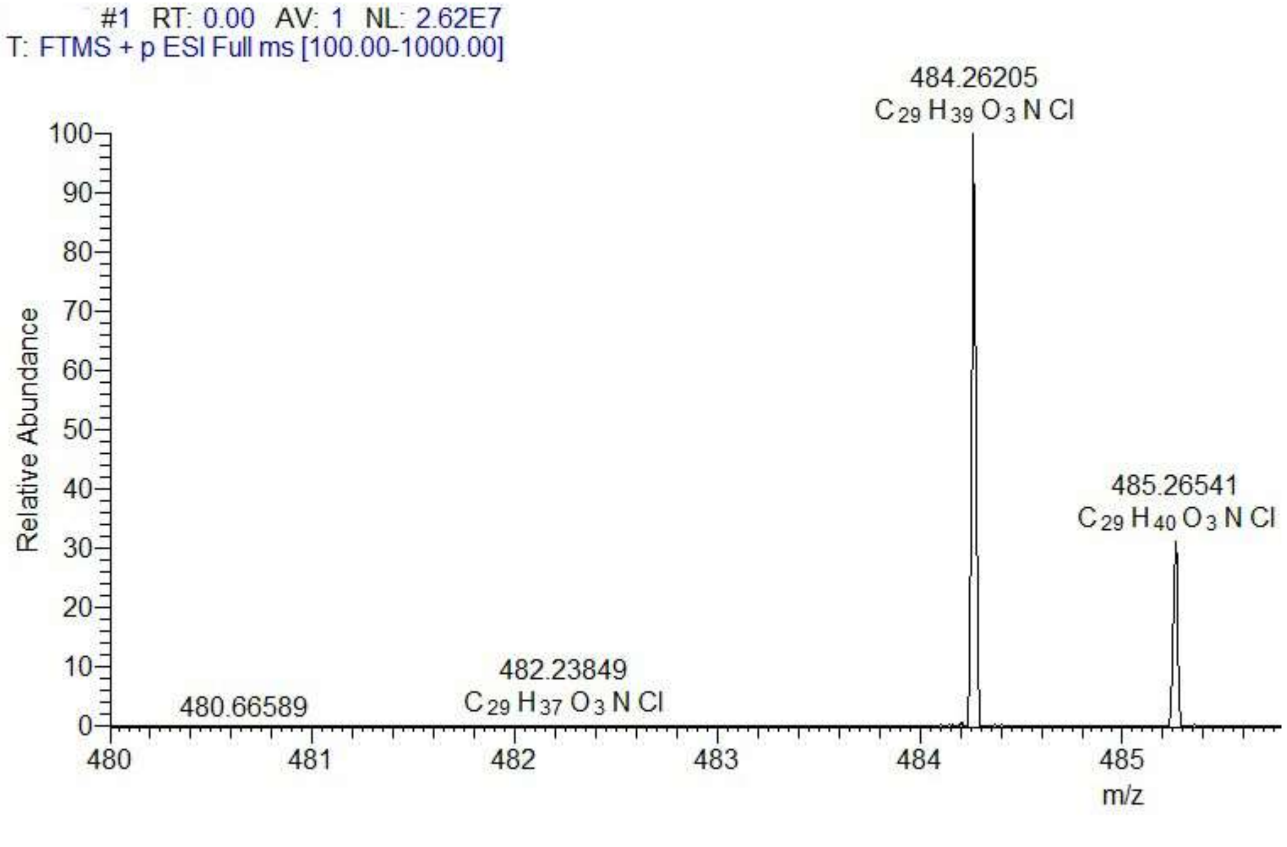

Figure S46. HRMS-ESI spectrum of the target compound (**3l**)

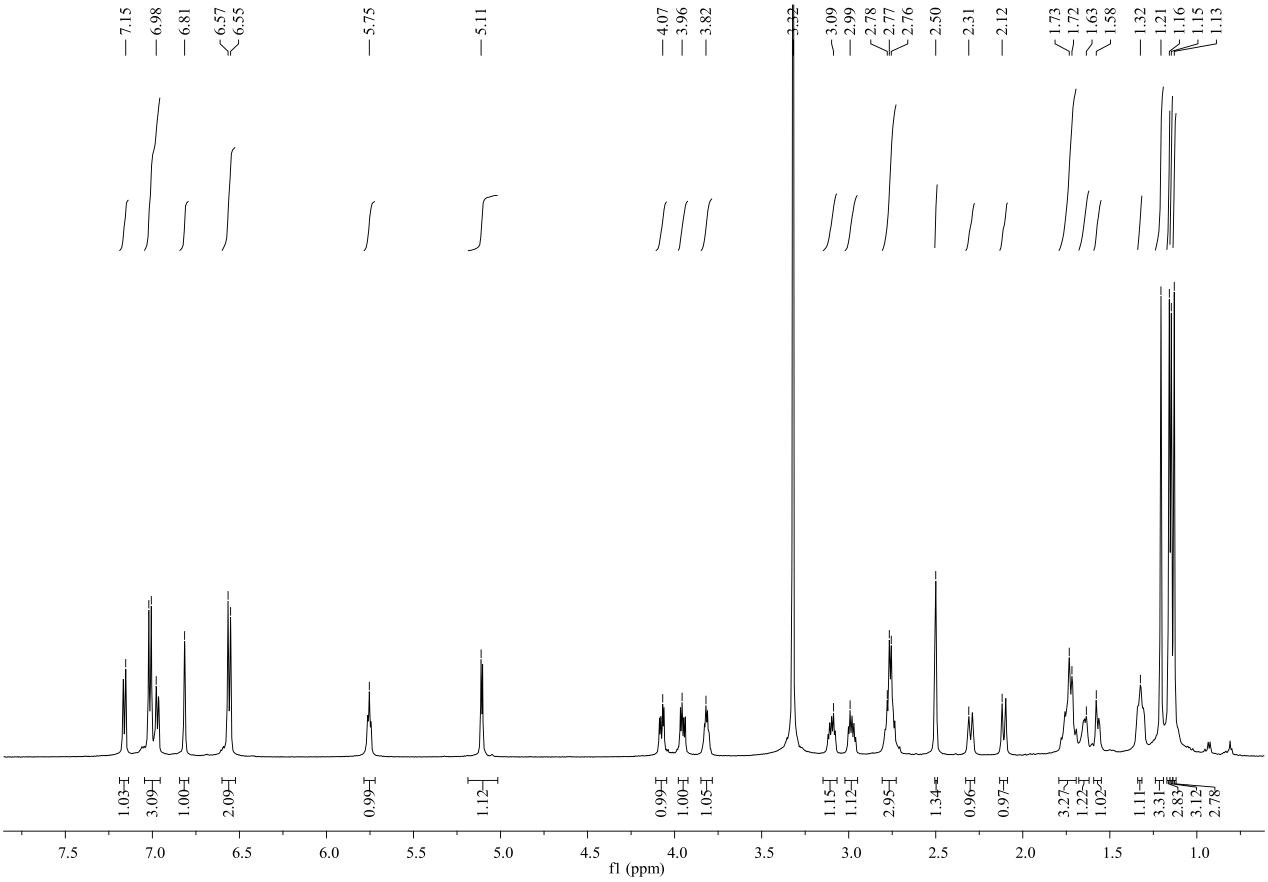

Figure S47. ^{1}H-NMR spectrum of the target compound (**3l**) in DMSO

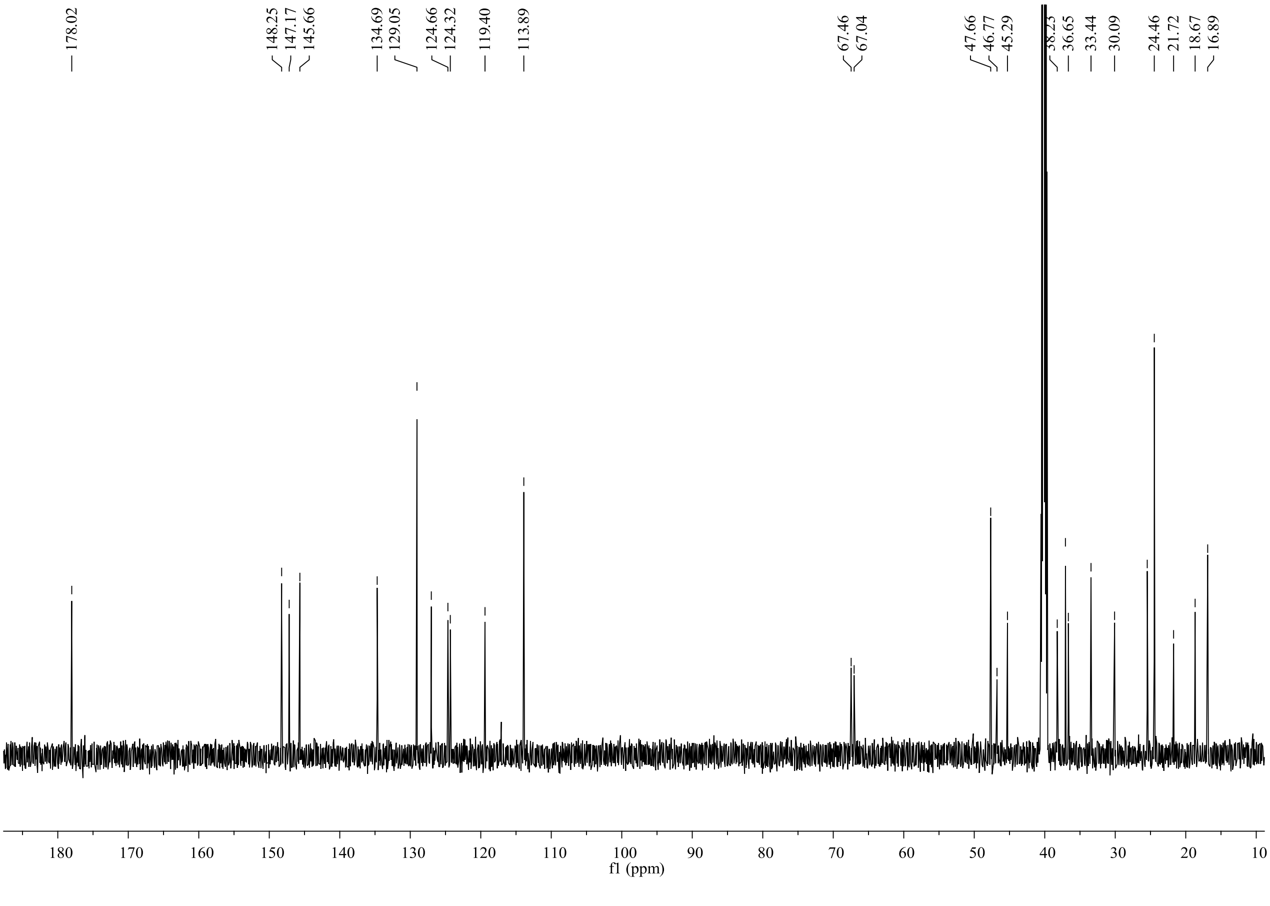

Figure S48. ^{13}C-NMR spectrum of the target compound (**3l**) in DMSO

Int. J. Mol. Sci. 2018, 19, 3116; doi:10.3390/ijms19103116

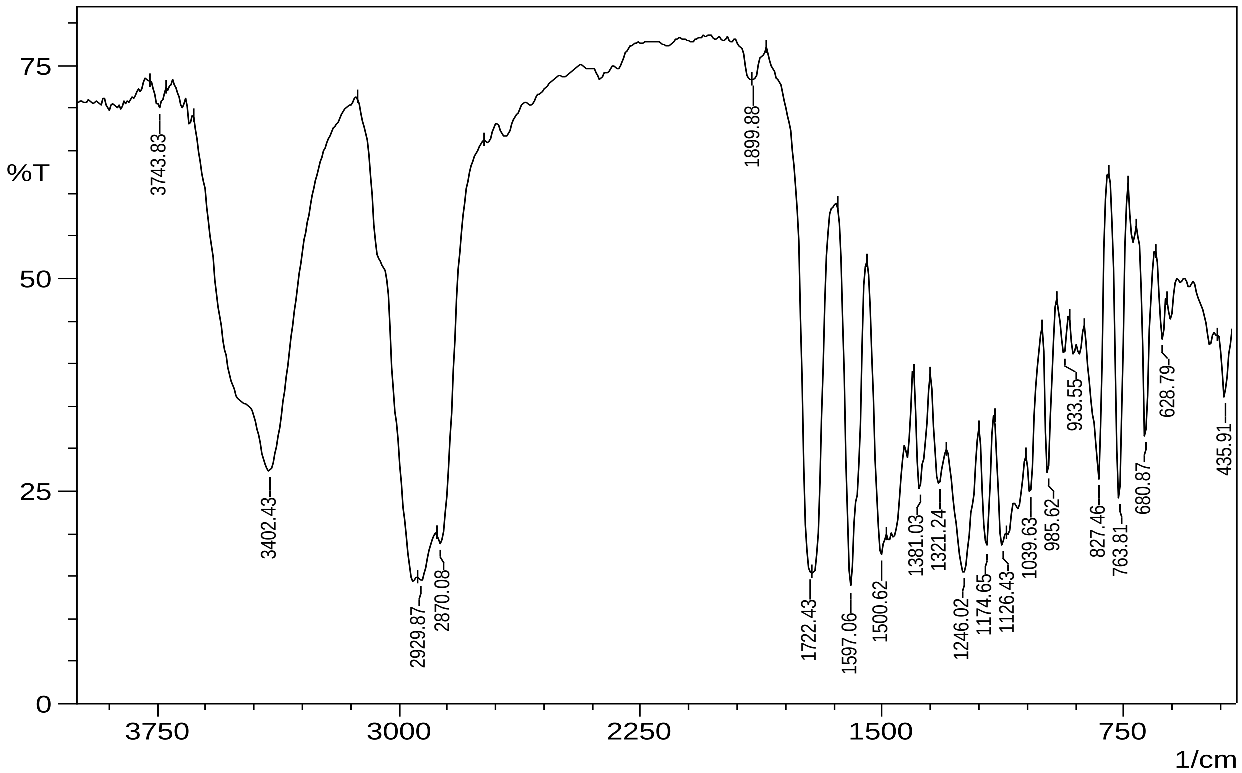

Figure S49. FTIR spectrum of the target compound (**3m**)

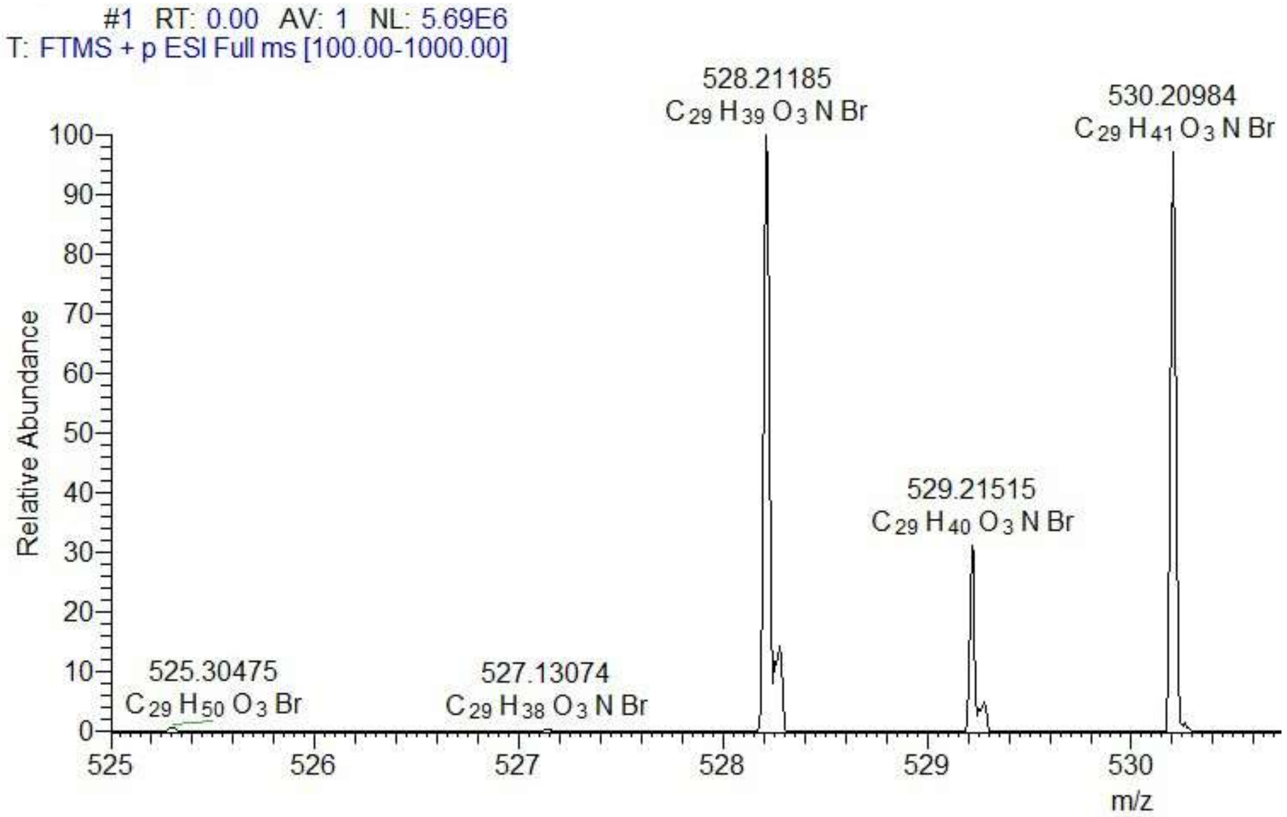

Figure S50. HRMS-ESI spectrum of the target compound (**3m**)

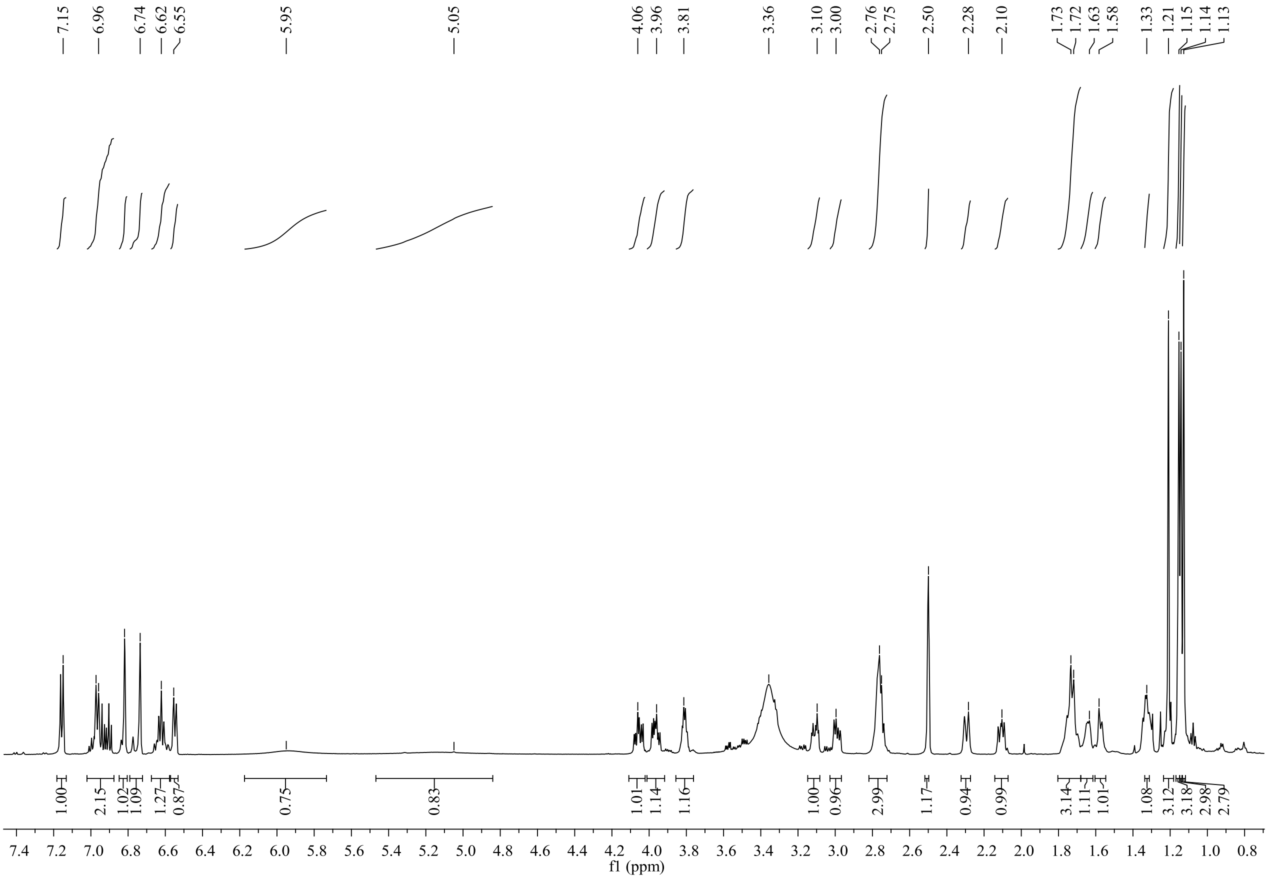

Figure S51. ^{1}H-NMR spectrum of the target compound (**3m**) in DMSO

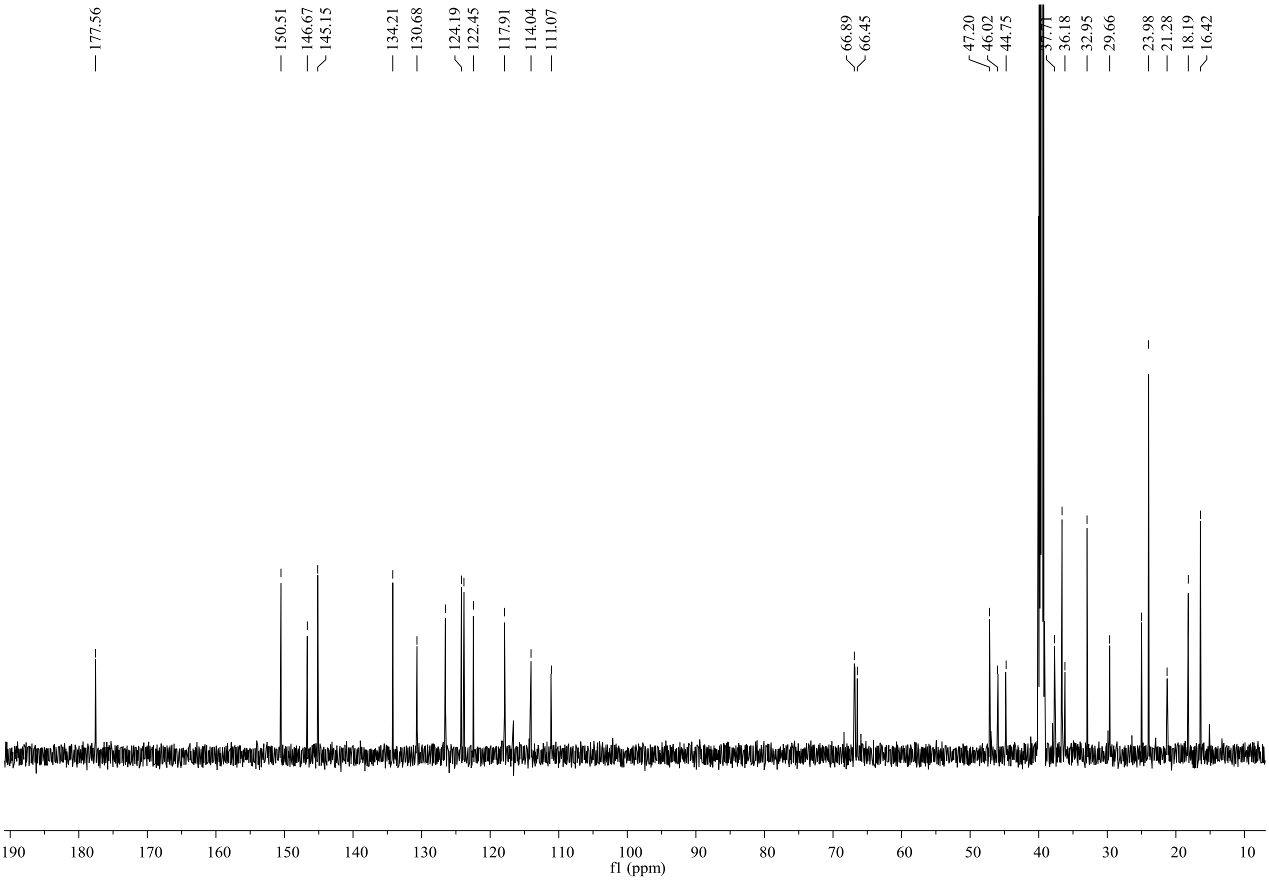

Figure S52. ^{13}C-NMR spectrum of the target compound (**3m**) in DMSO

Int. J. Mol. Sci. 2018, 19, 3116; doi:10.3390/ijms19103116

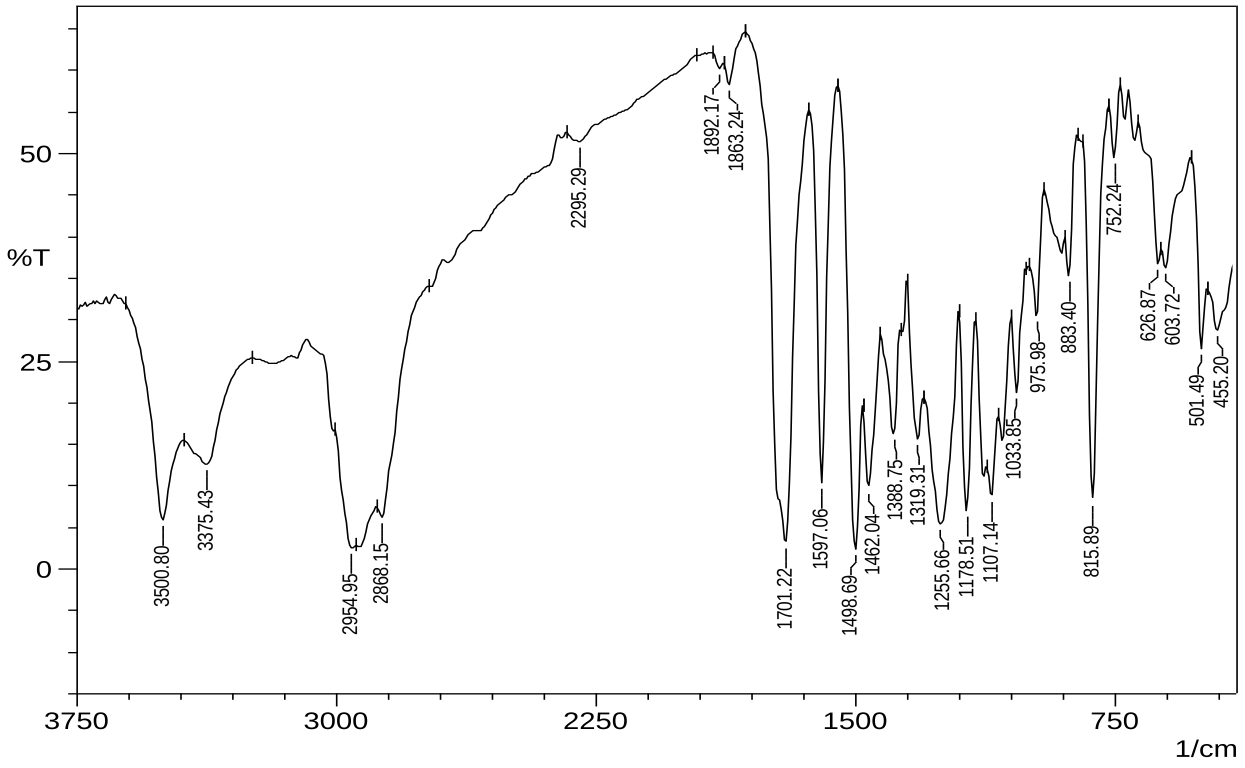

Figure S53. FTIR spectrum of the target compound (**3n**)

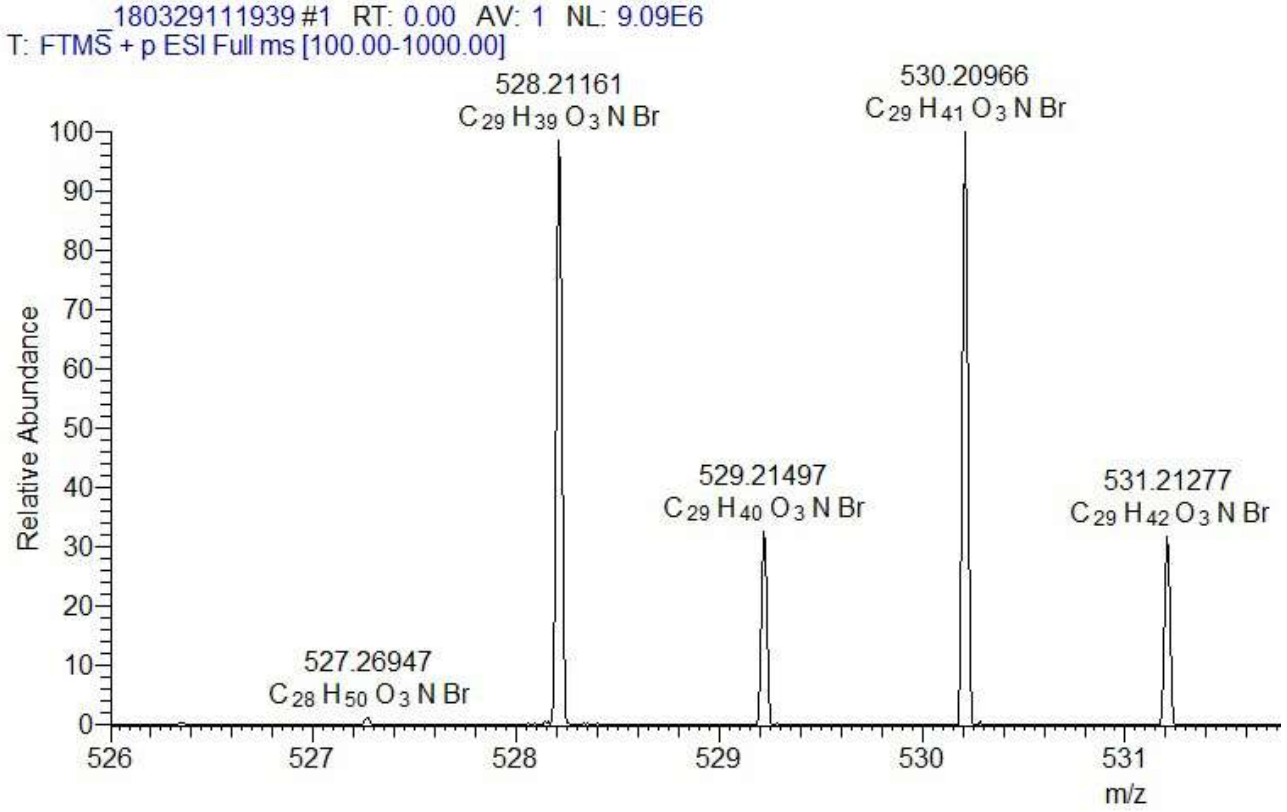

Figure S54. HRMS-ESI spectrum of the target compound (**3n**)

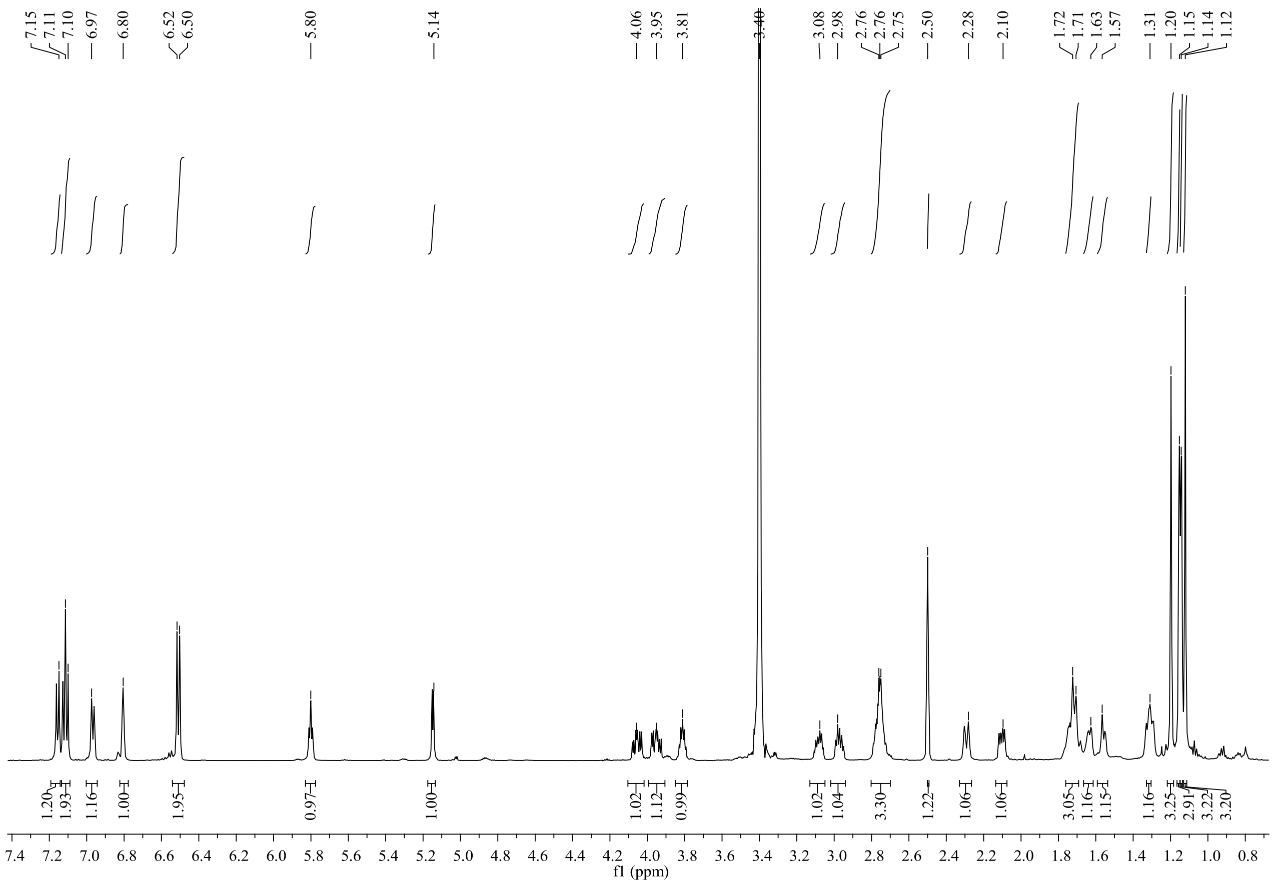

Figure S55. ^{1}H-NMR spectrum of the target compound (**3n**) in DMSO

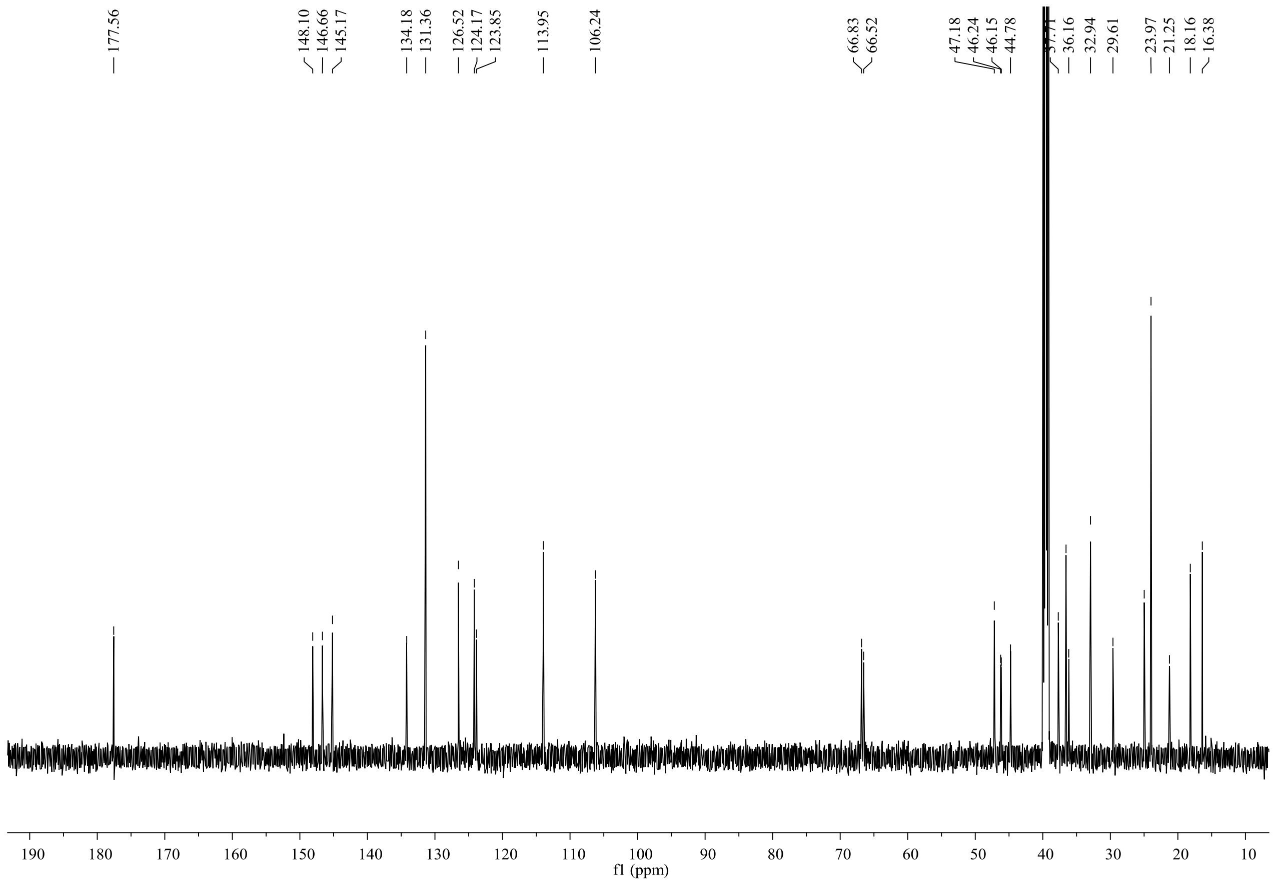

Figure S56. ^{13}C-NMR spectrum of the target compound (**3n**) in DMSO

Int. J. Mol. Sci. 2018, 19, 3116; doi:10.3390/ijms19103116

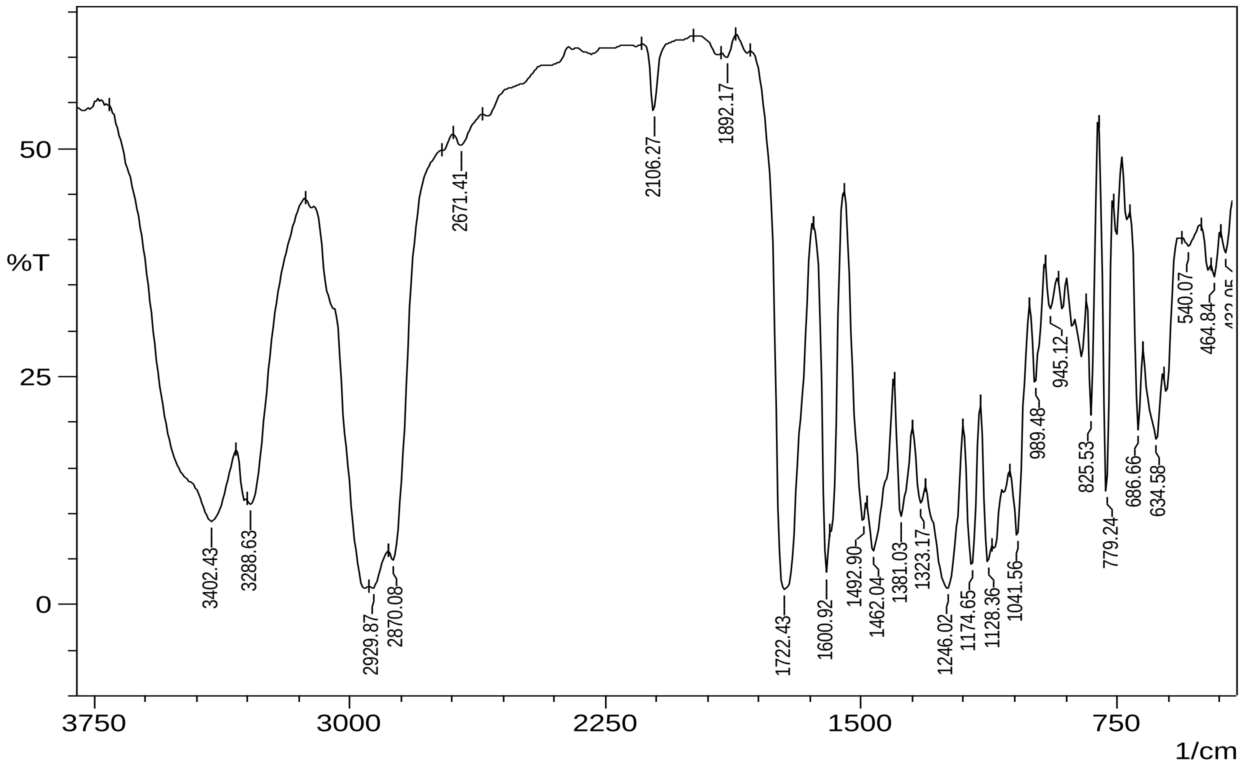

Figure S57. FTIR spectrum of the target compound (**3o**)

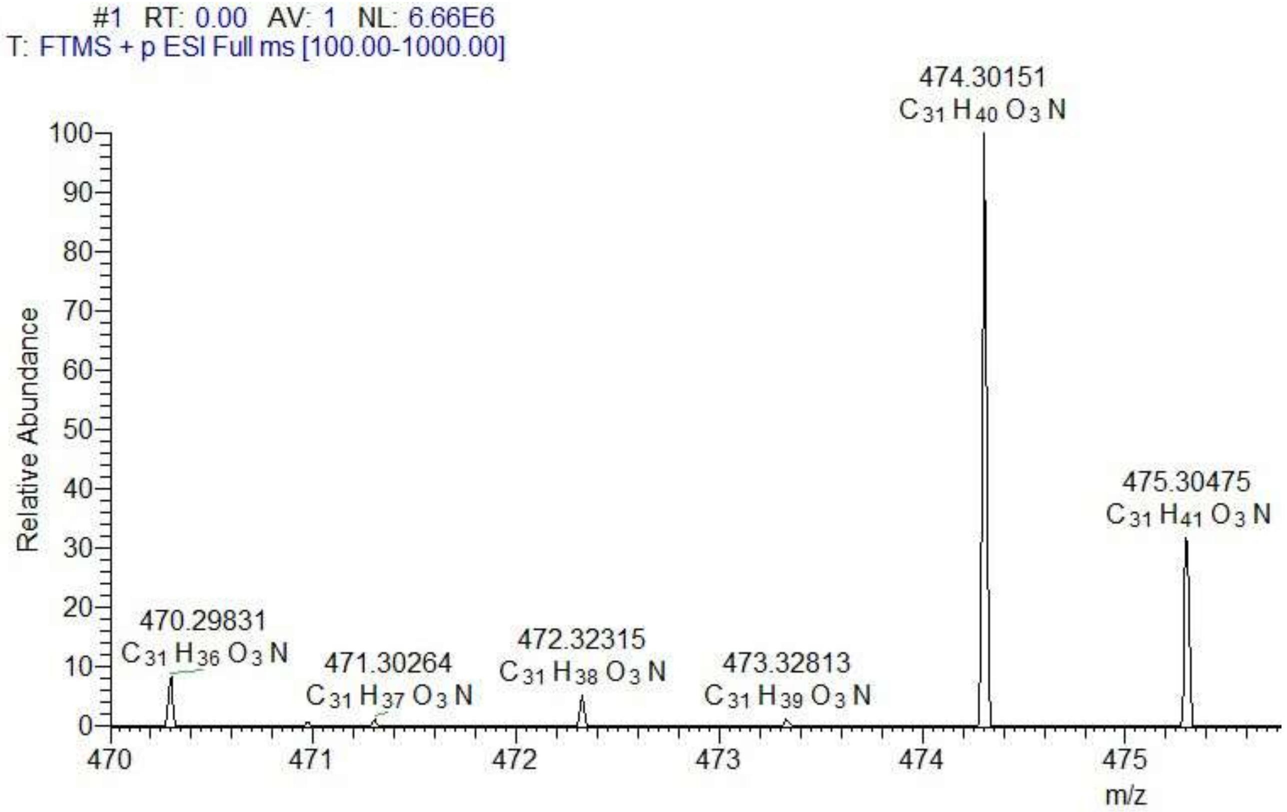

Figure S58. HRMS-ESI spectrum of the target compound (**3o**)

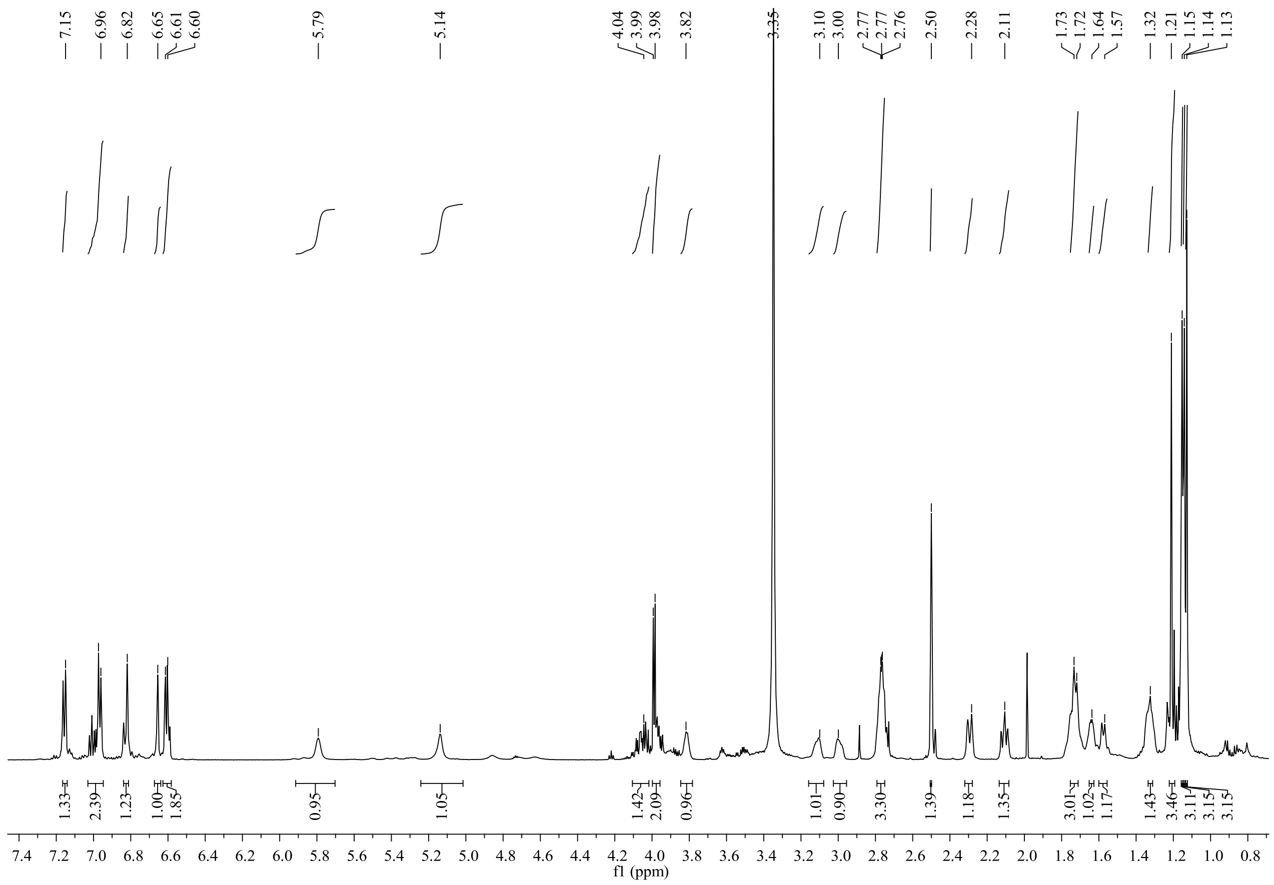

Figure S59. ^{1}H-NMR spectrum of the target compound (**3o**) in DMSO

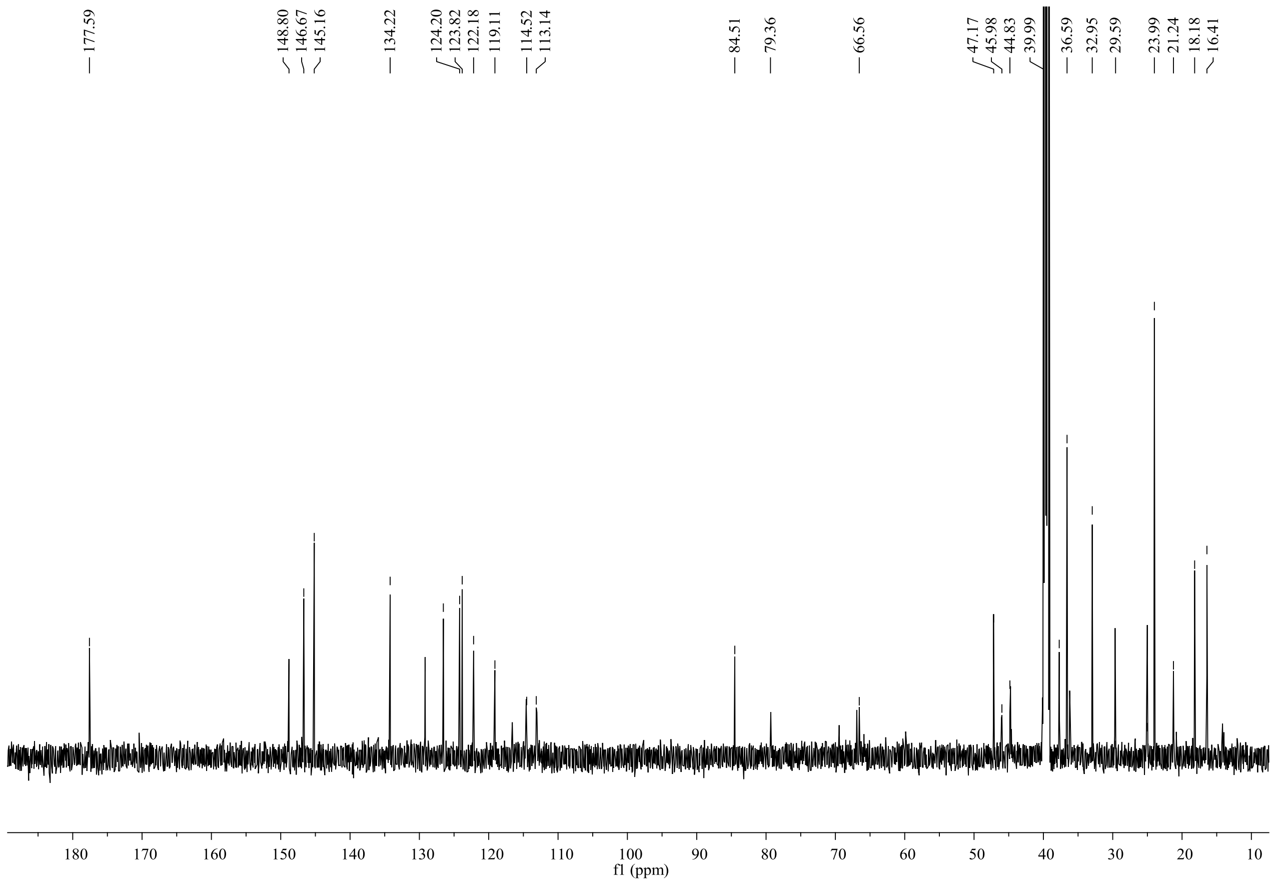

Figure S60. ^{13}C-NMR spectrum of the target compound (**3o**) in DMSO

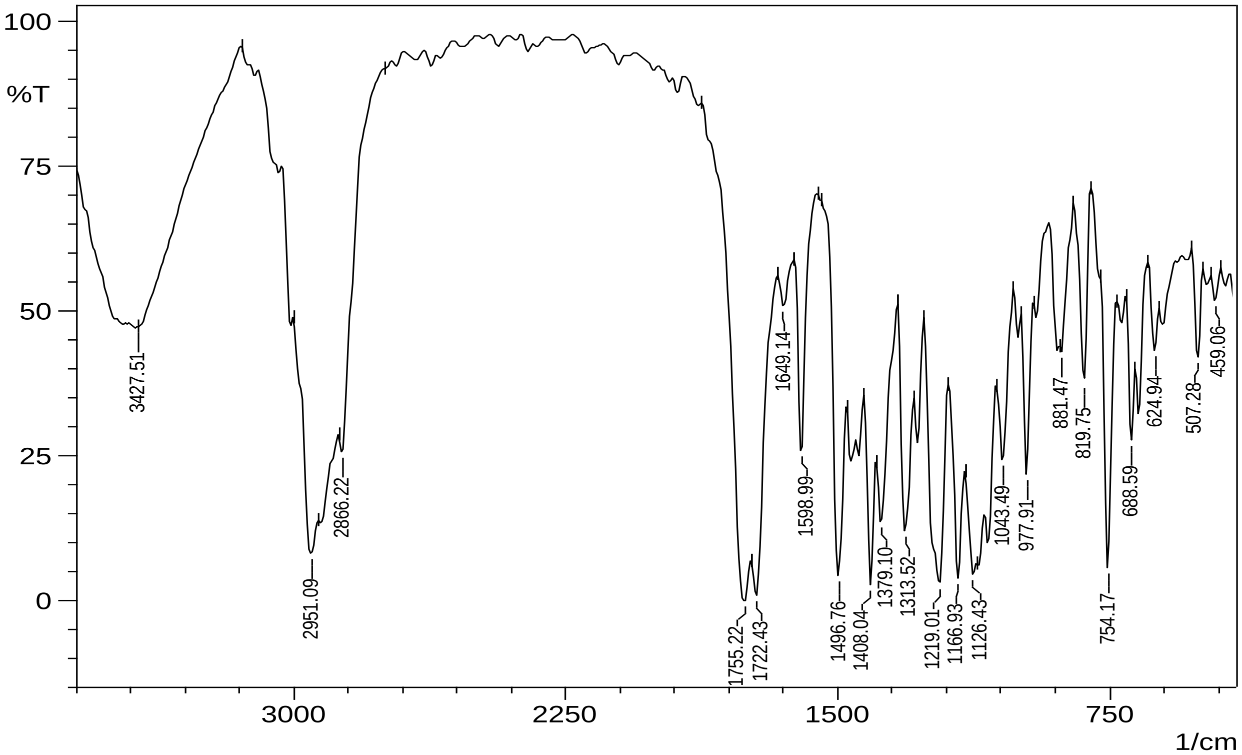

Figure S61. FTIR spectrum of the target compound (**4a**)

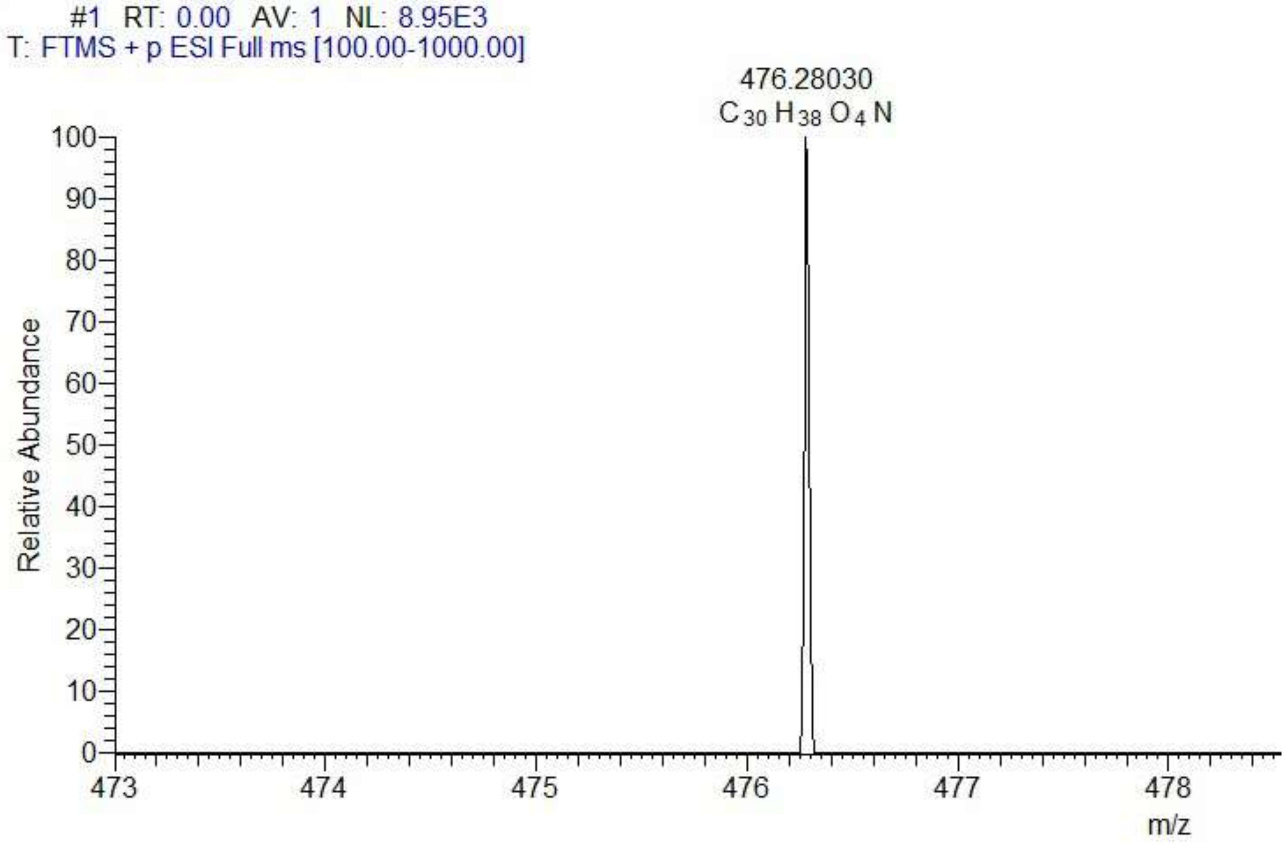

Figure S62. HRMS-ESI spectrum of the target compound (**4a**)

Int. J. Mol. Sci. 2018, 19, 3116; doi:10.3390/ijms19103116

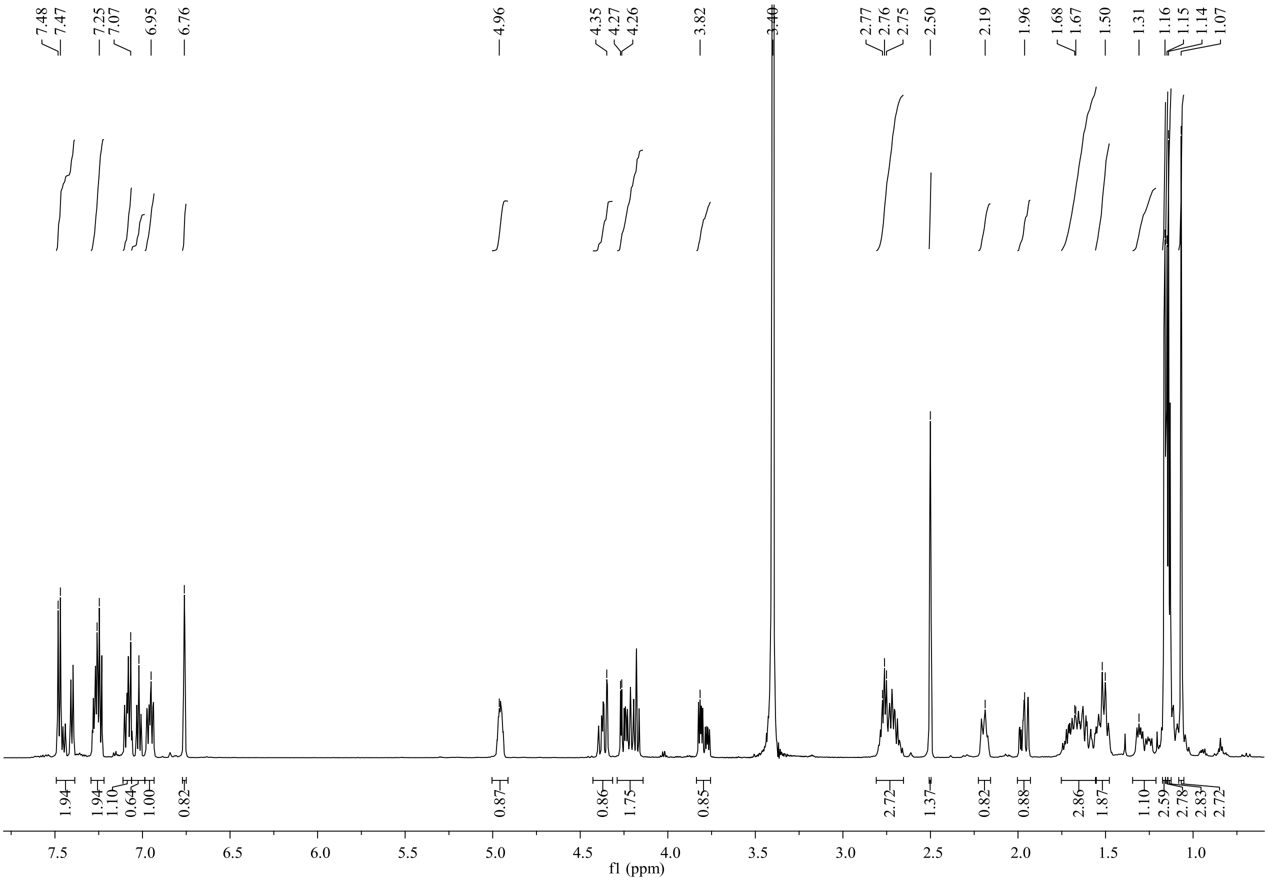

Figure S63. ^{1}H-NMR spectrum of the target compound (**4a**) in DMSO

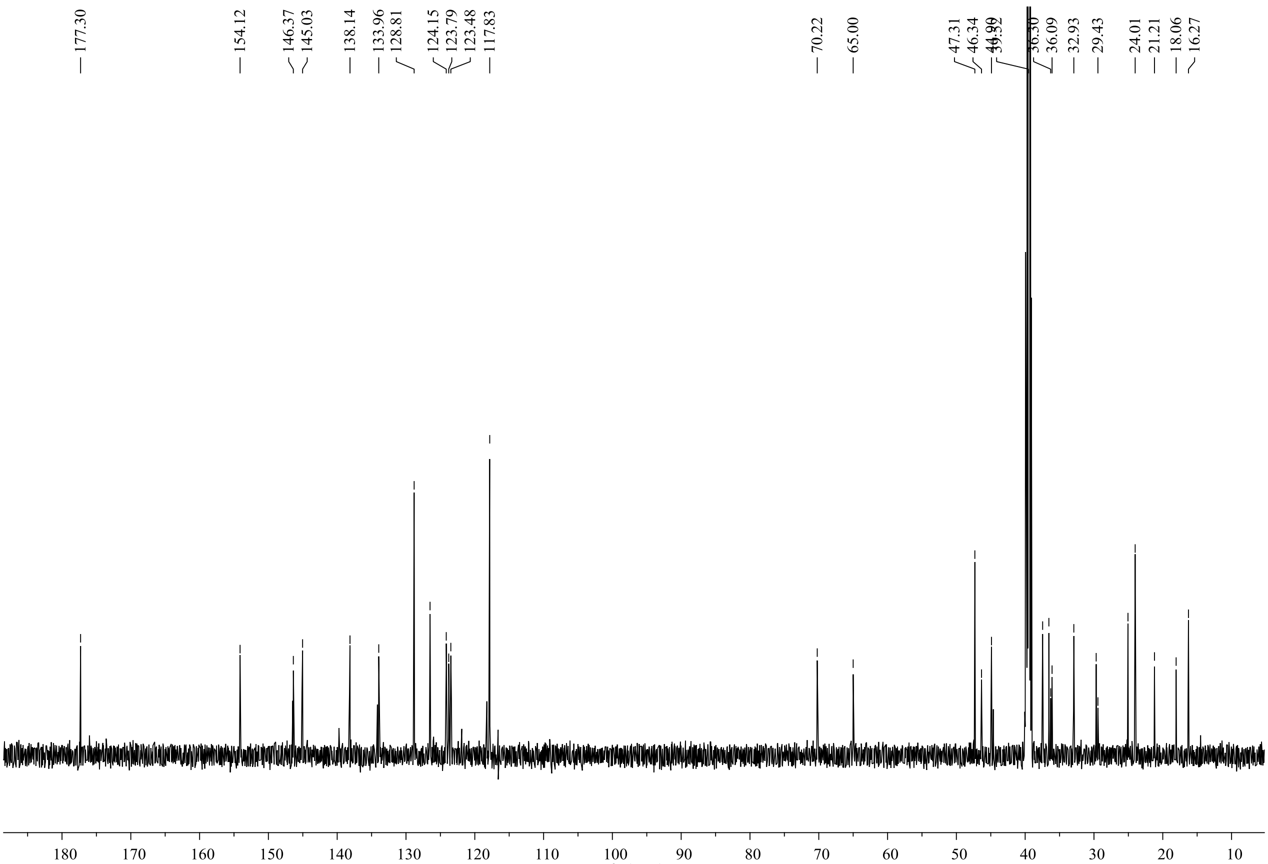

Figure S64. ^{13}C-NMR spectrum of the target compound (**4a**) in DMSO

Int. J. Mol. Sci. 2018, 19, 3116; doi:10.3390/ijms19103116

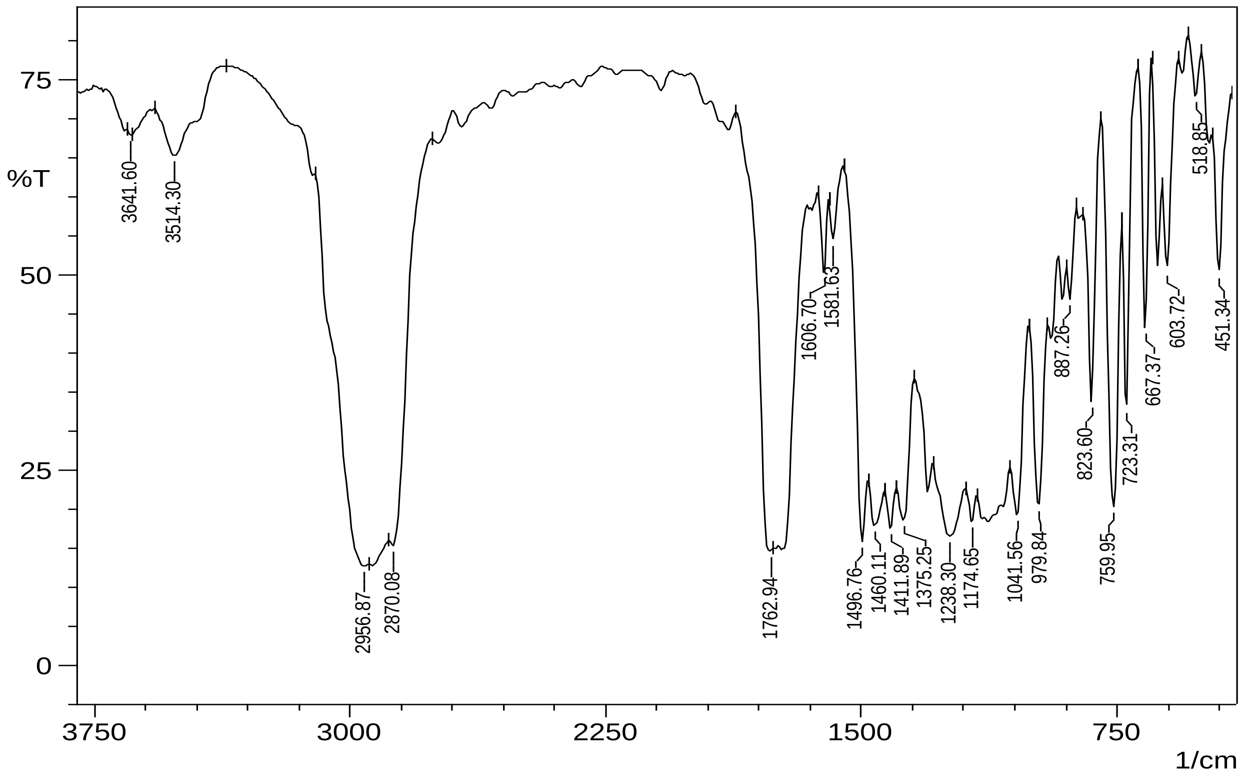

Figure S65. FTIR spectrum of the target compound (**4b**)

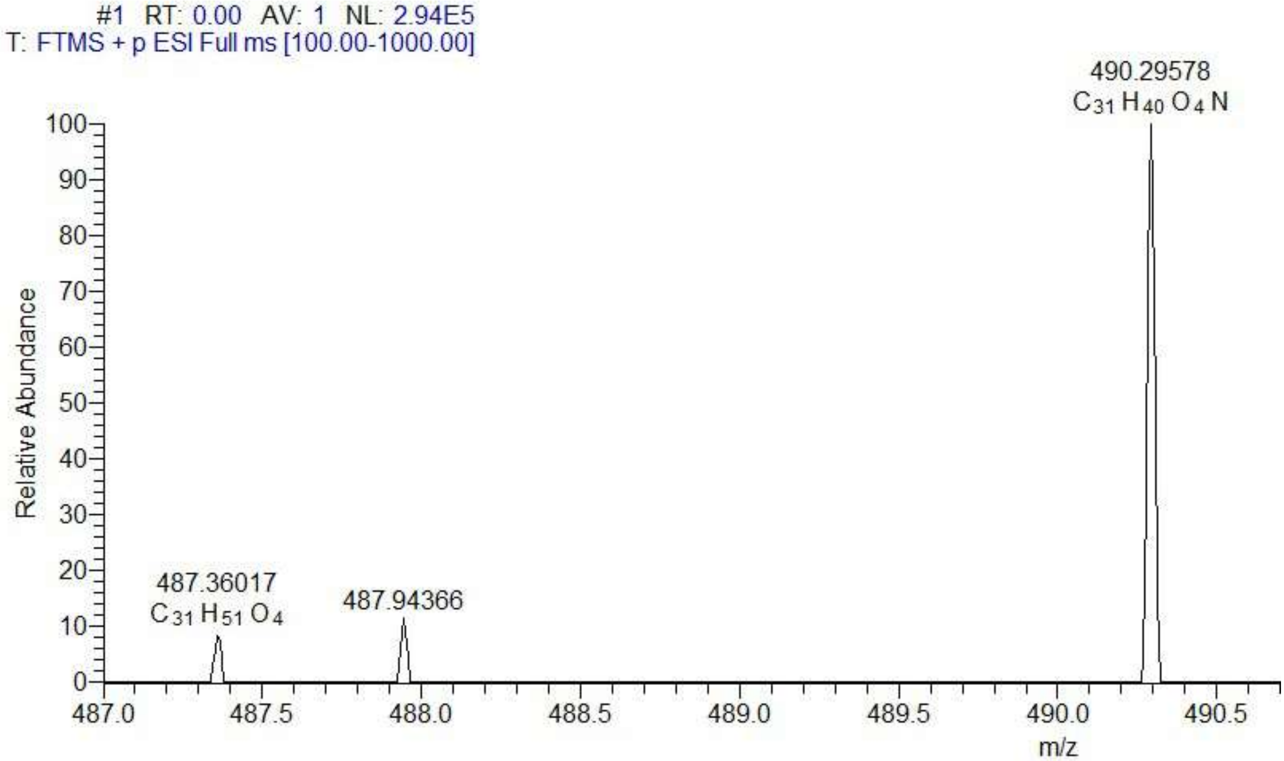

Figure S66. HRMS-ESI spectrum of the target compound (**4b**)

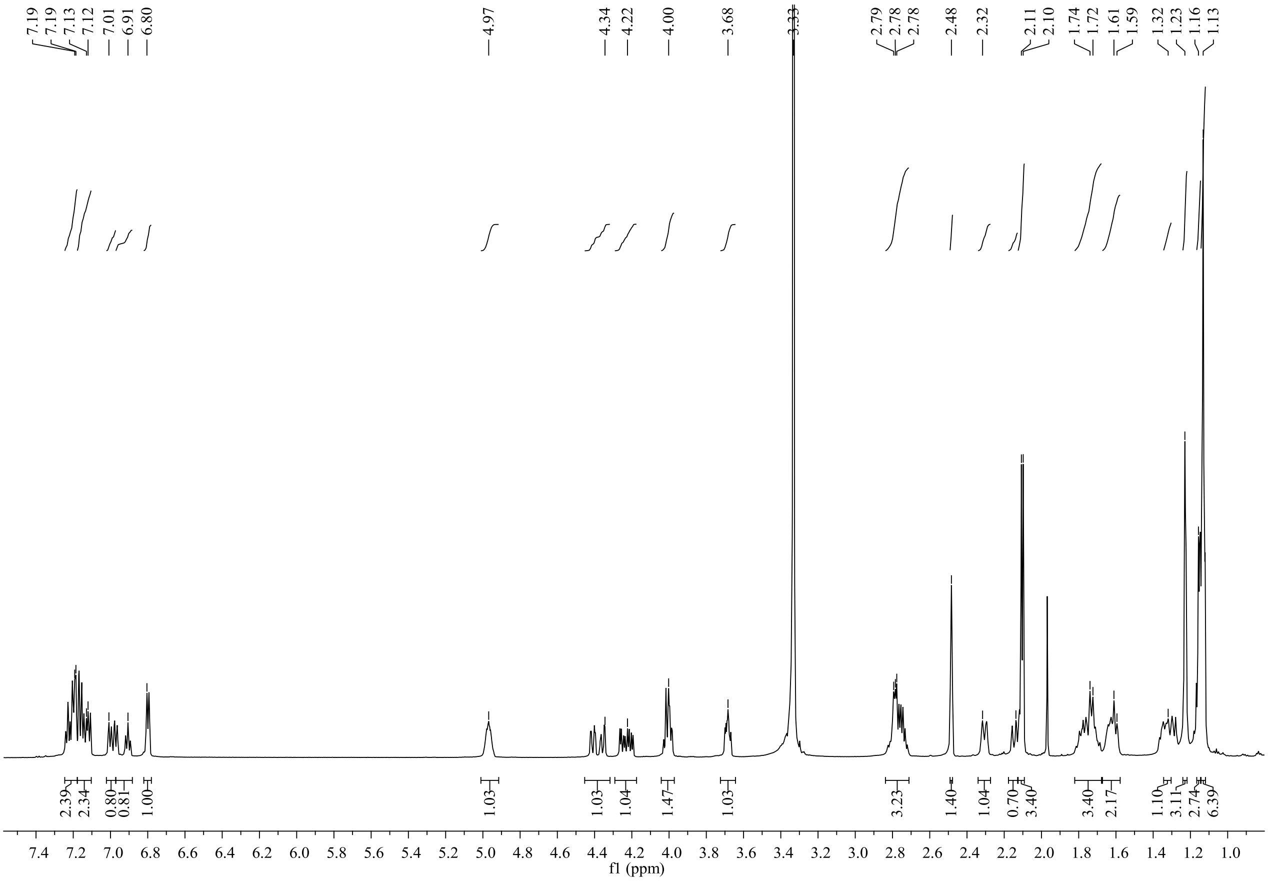

Figure S67. ^{1}H-NMR spectrum of the target compound (**4b**) in DMSO

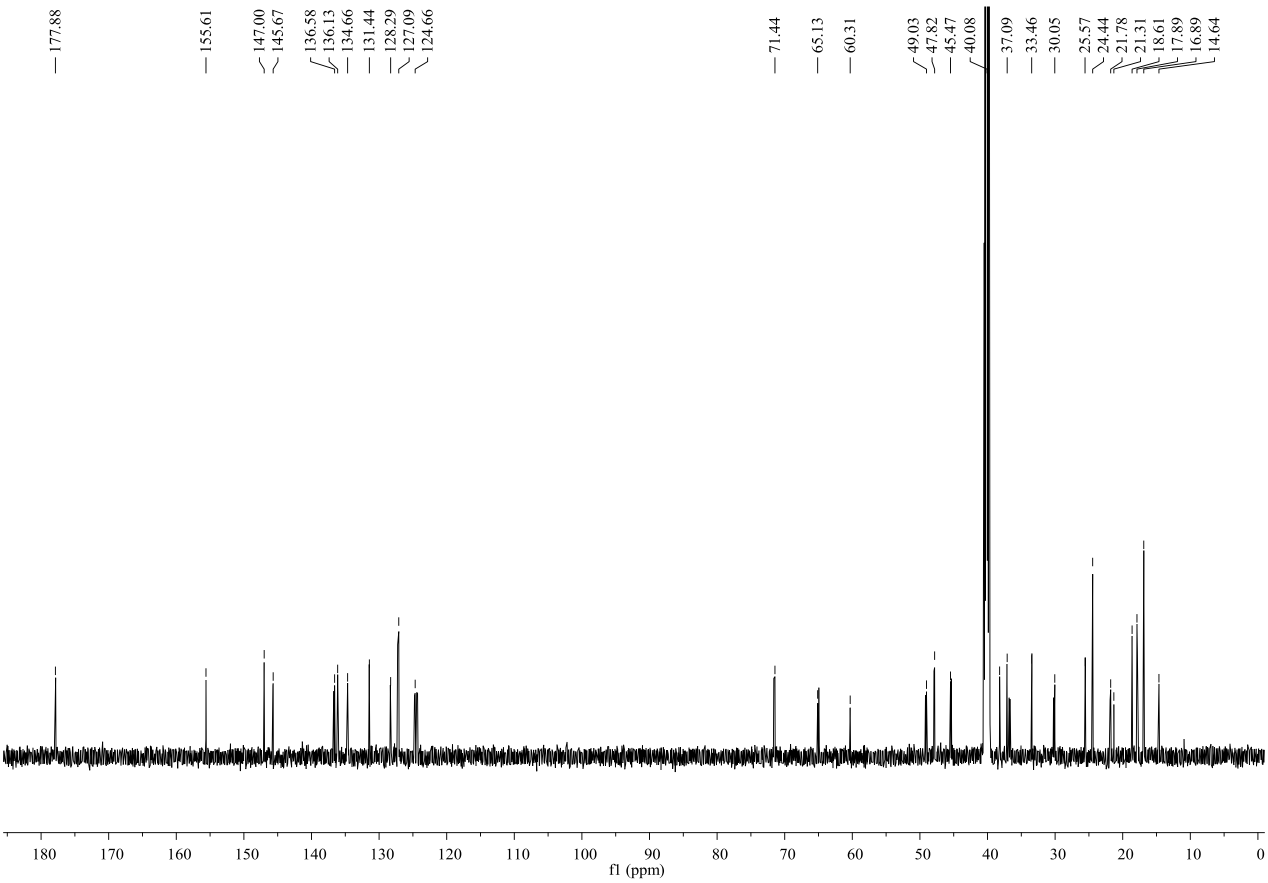

Figure S68. ^{13}C-NMR spectrum of the target compound (**4b**) in DMSO

Int. J. Mol. Sci. 2018, 19, 3116; doi:10.3390/ijms19103116

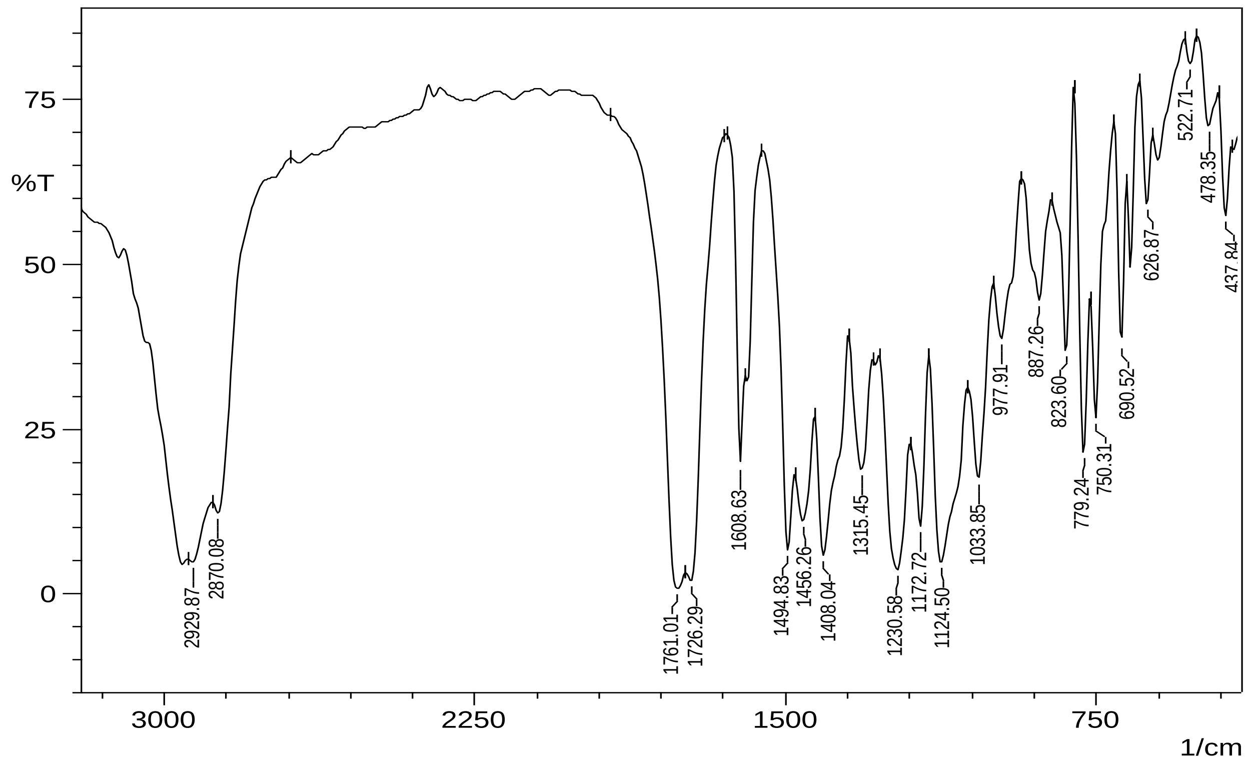

Figure S69. FTIR spectrum of the target compound (**4c**)

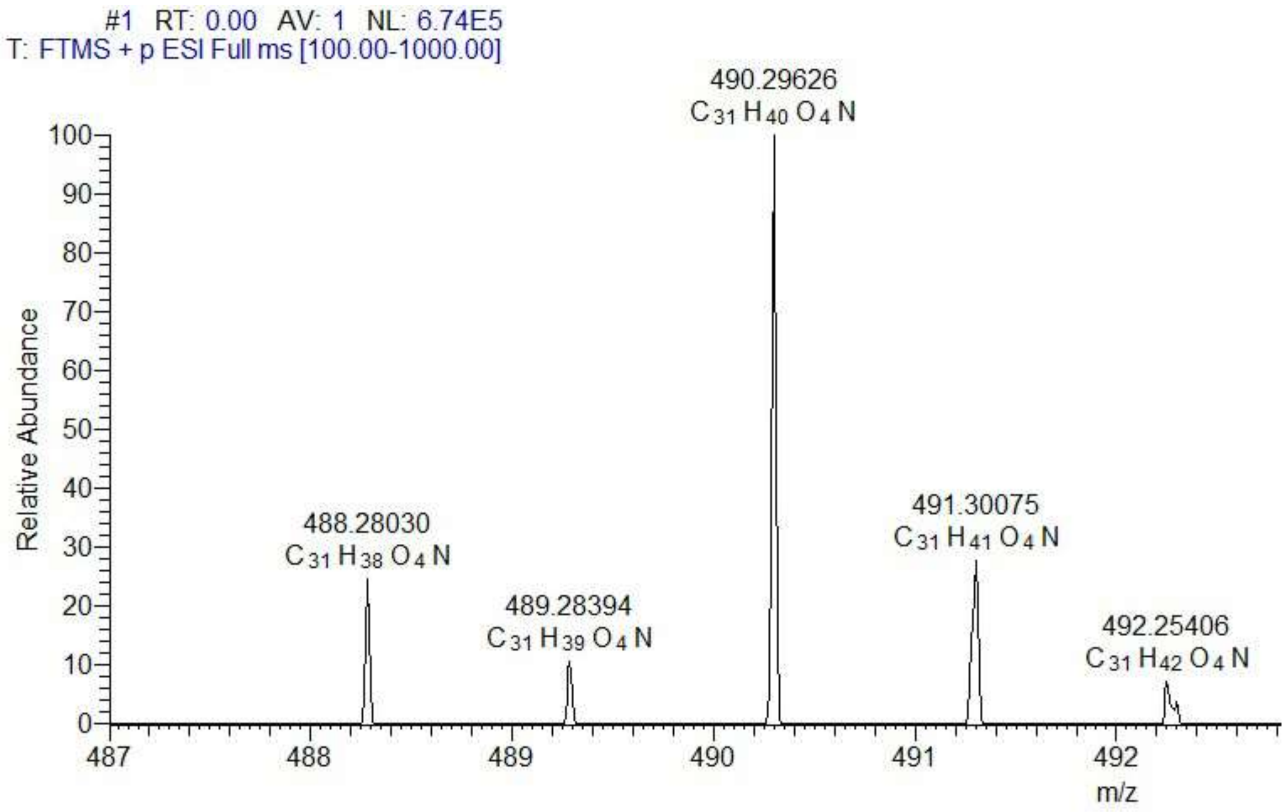

Figure S70. HRMS-ESI spectrum of the target compound (**4c**)

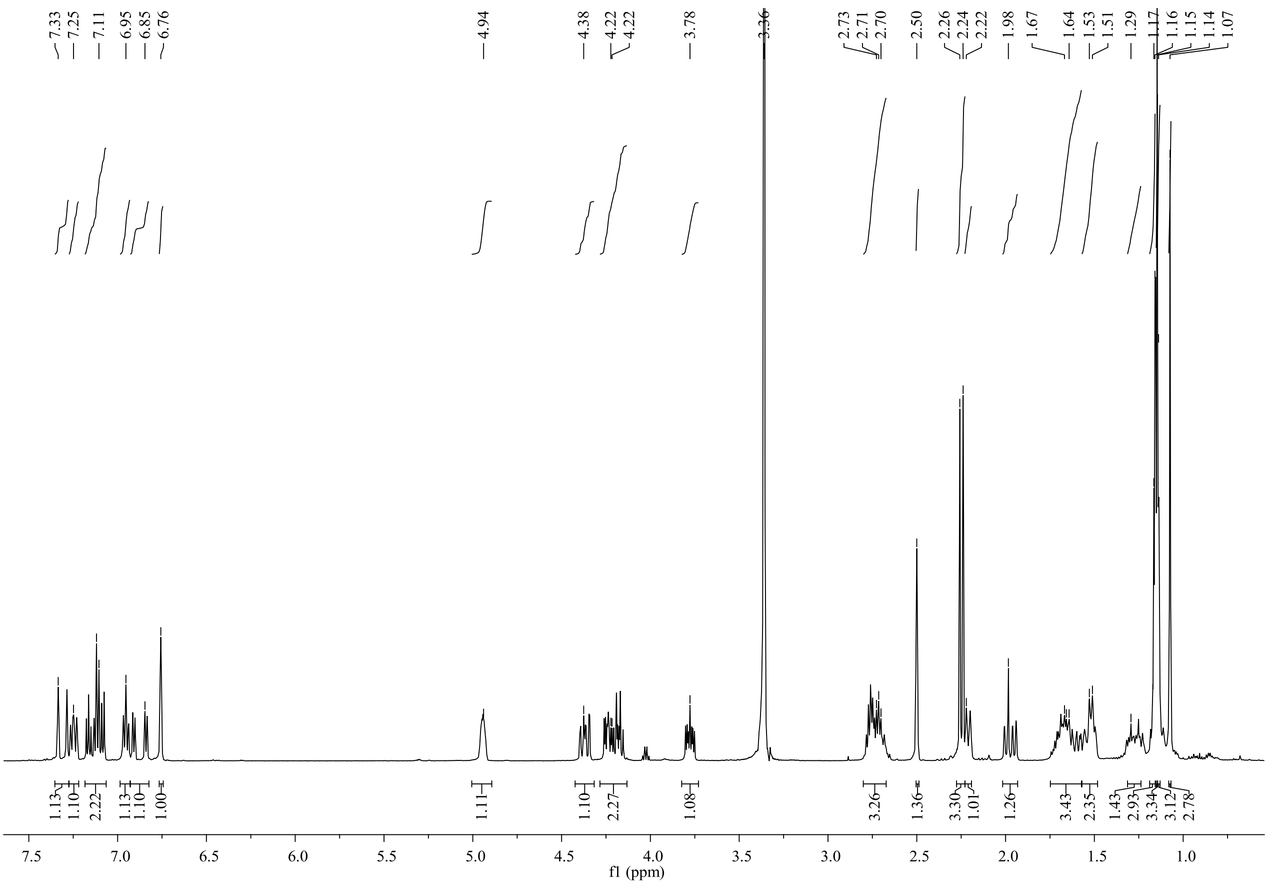

Figure S71. ^{1}H-NMR spectrum of the target compound (**4c**) in DMSO

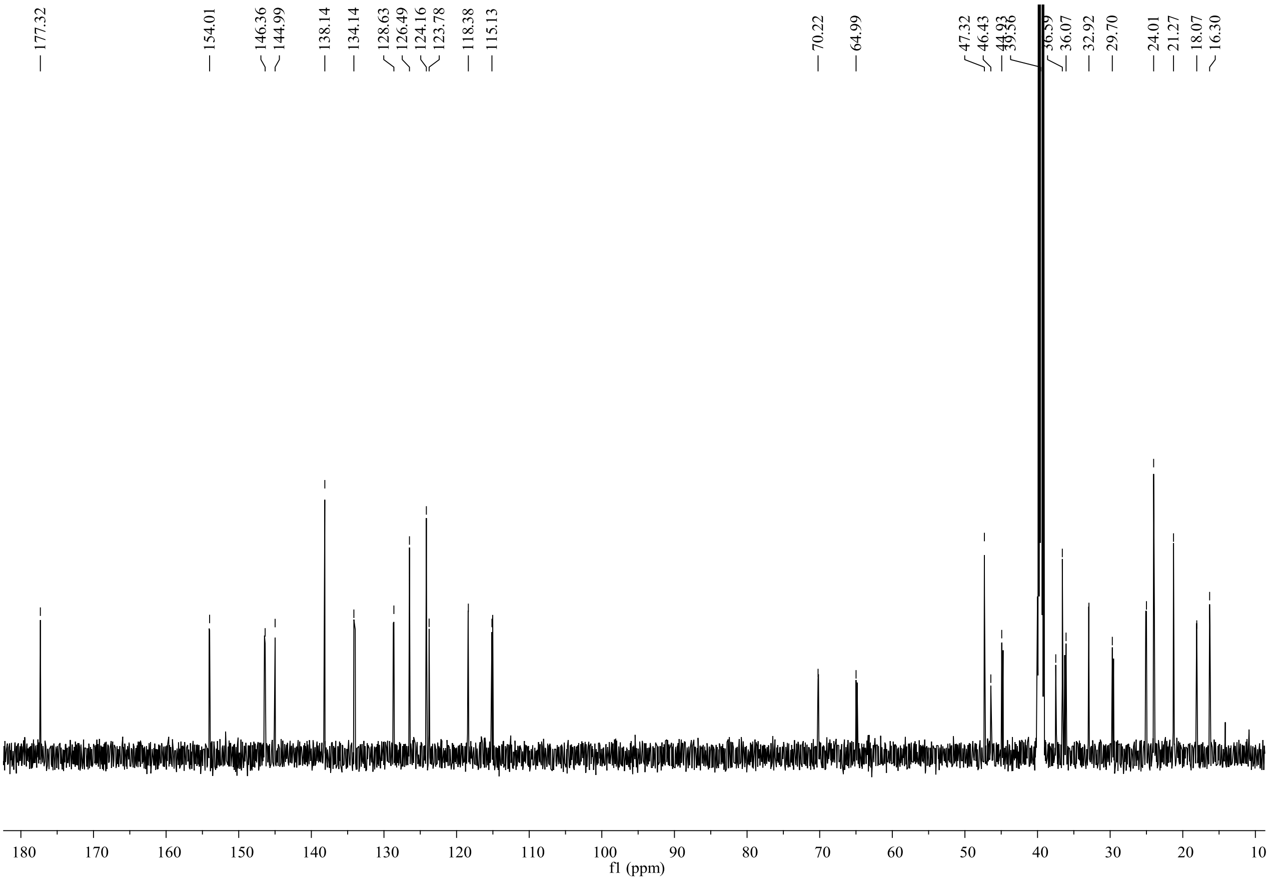

Figure S72. ^{13}C-NMR spectrum of the target compound (**4c**) in DMSO

Int. J. Mol. Sci. 2018, 19, 3116; doi:10.3390/ijms19103116

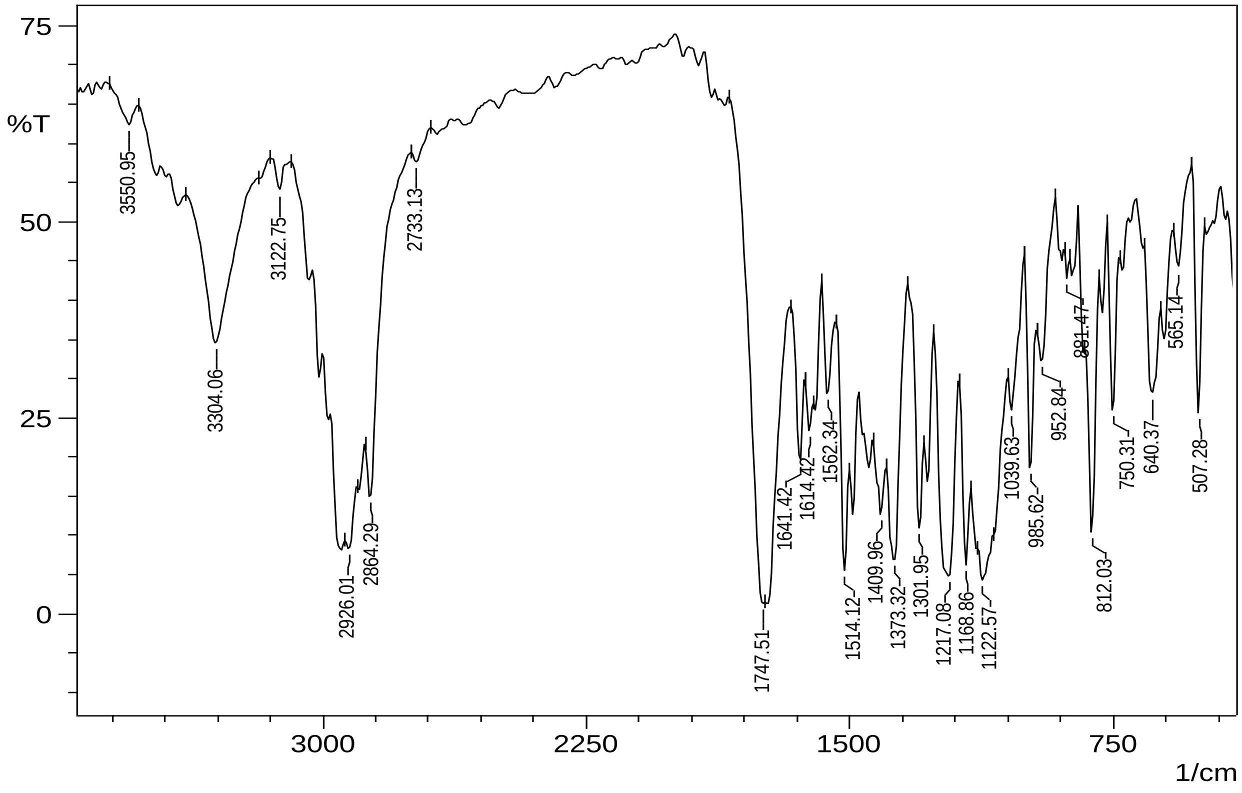

Figure S73. FTIR spectrum of the target compound (**4d**)

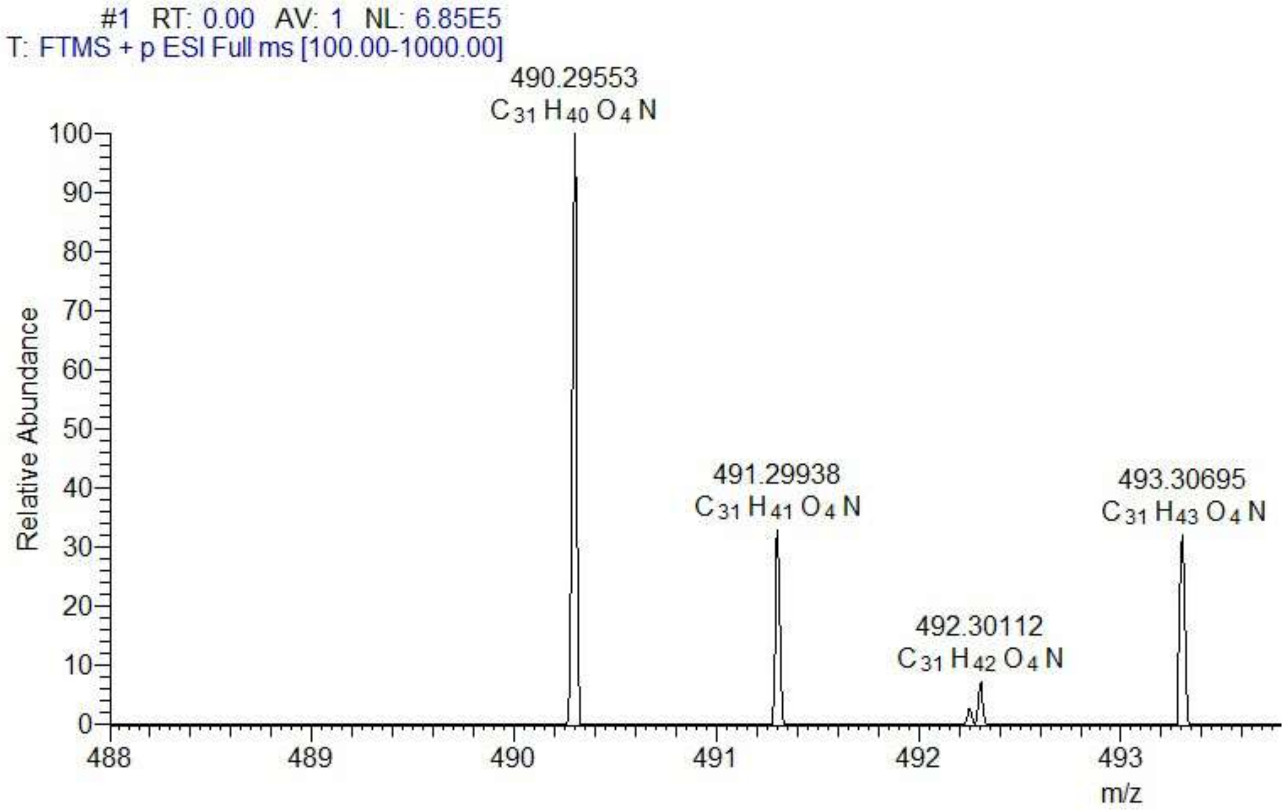

Figure S74. HRMS-ESI spectrum of the target compound (**4d**)

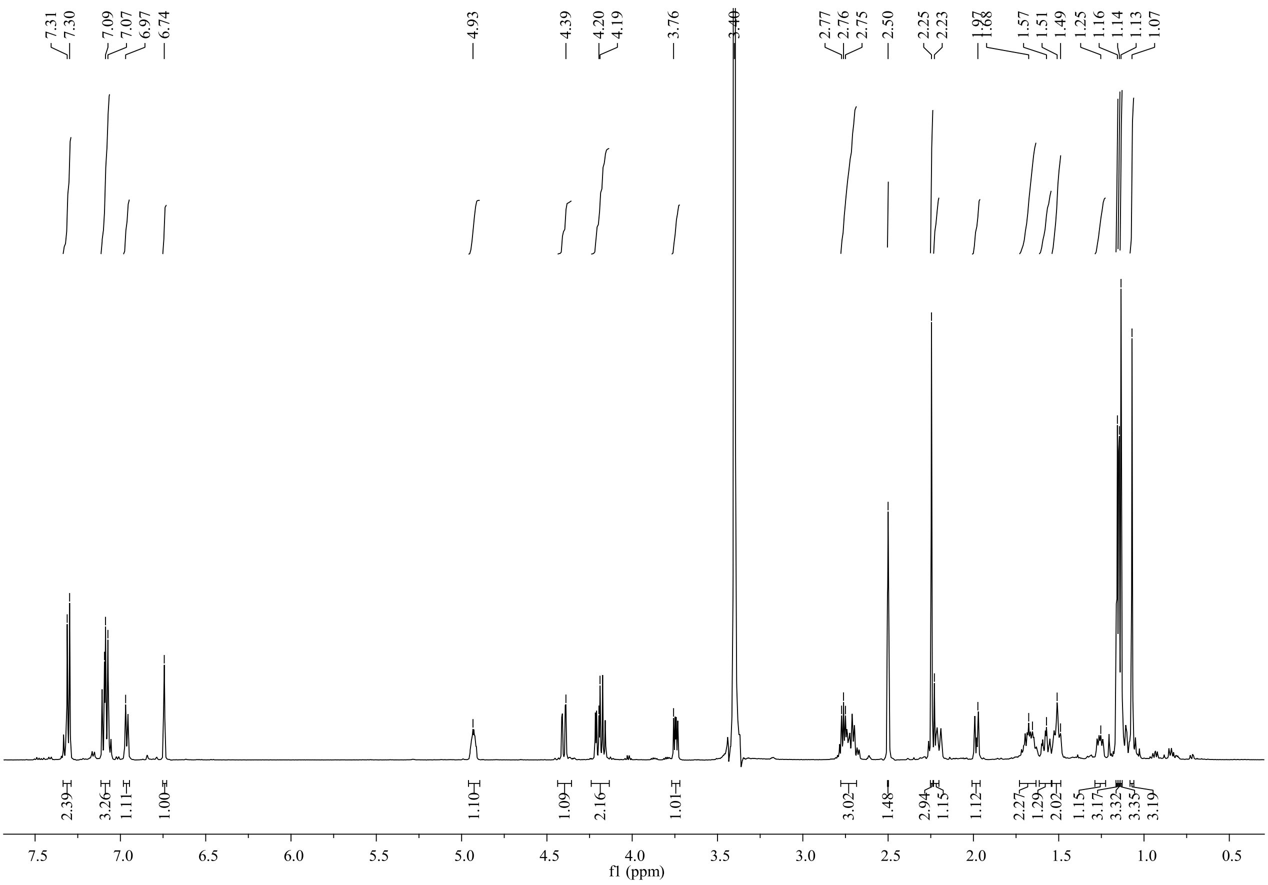

Figure S75. ^{1}H-NMR spectrum of the target compound (**4d**) in DMSO

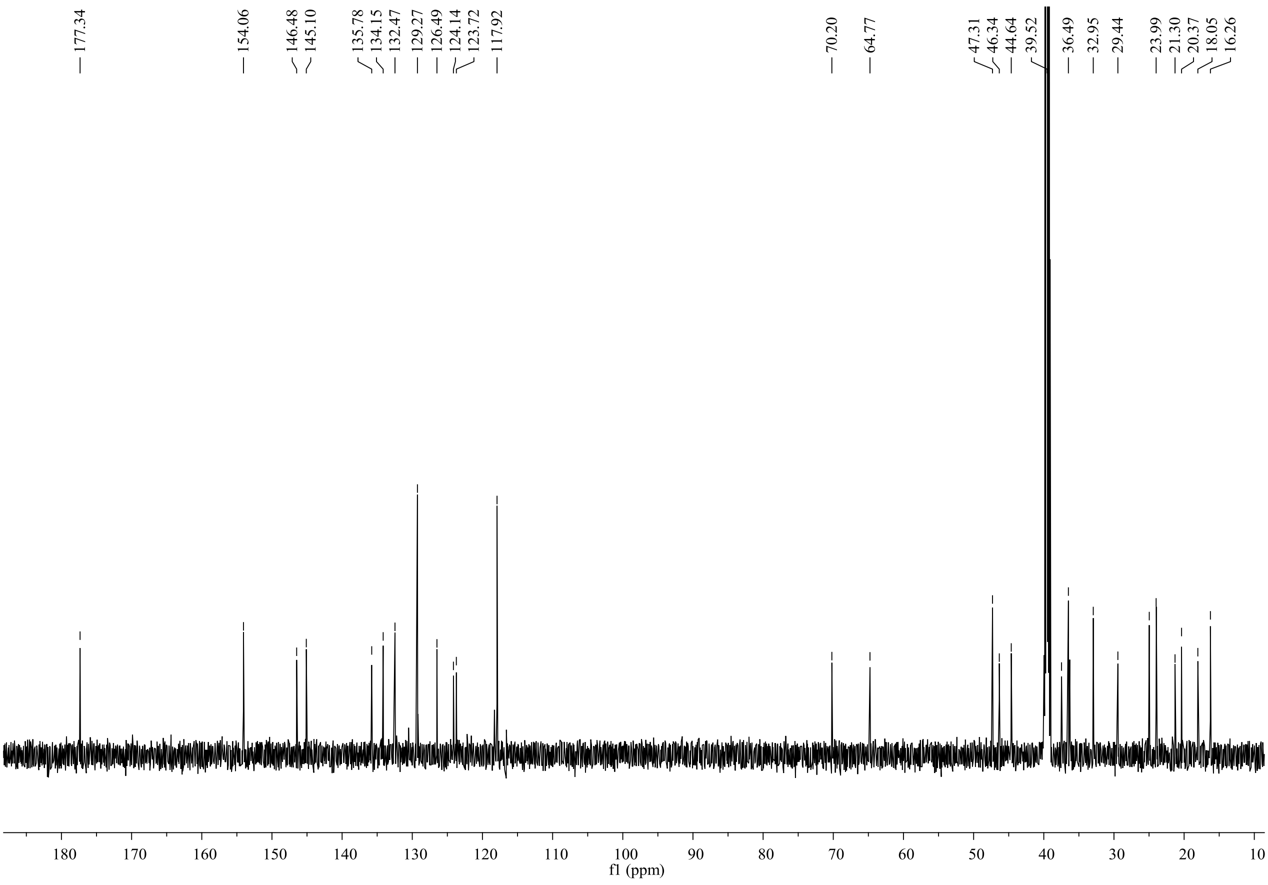

Figure S76. ^{13}C-NMR spectrum of the target compound (**4d**) in DMSO

Int. J. Mol. Sci. 2018, 19, 3116; doi:10.3390/ijms19103116

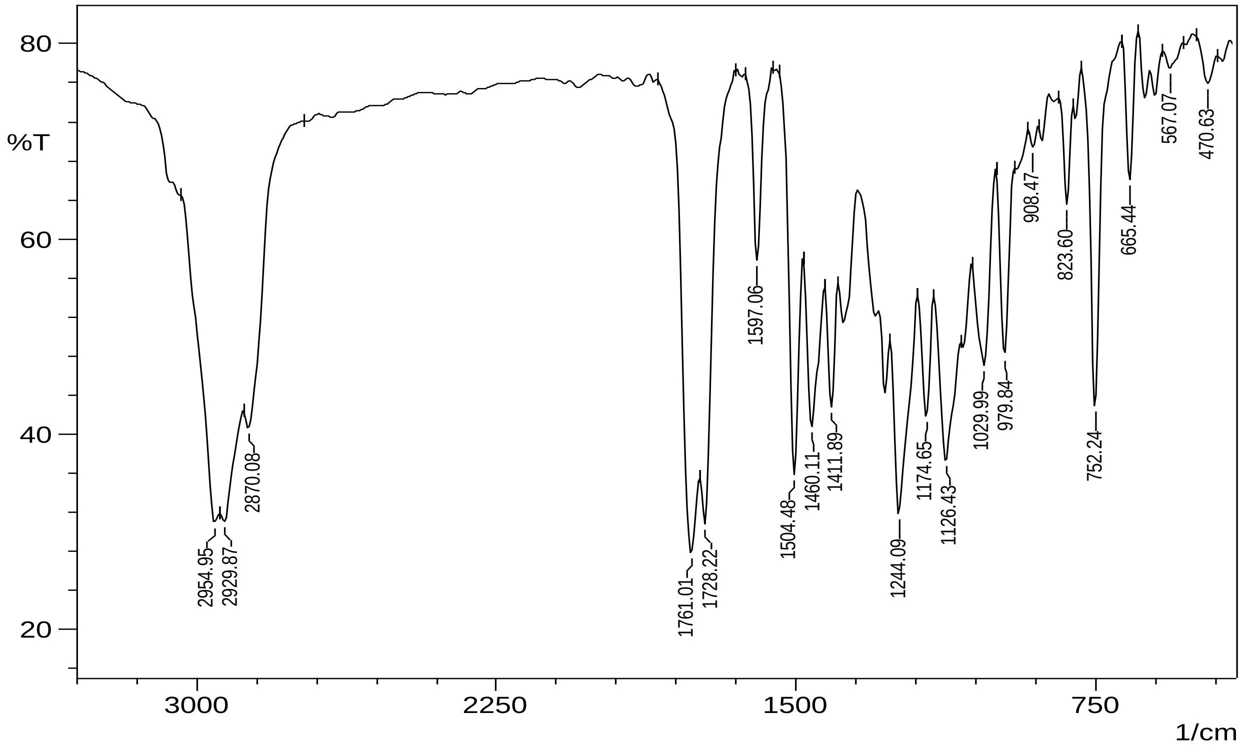

Figure S77. FTIR spectrum of the target compound (**4e**)

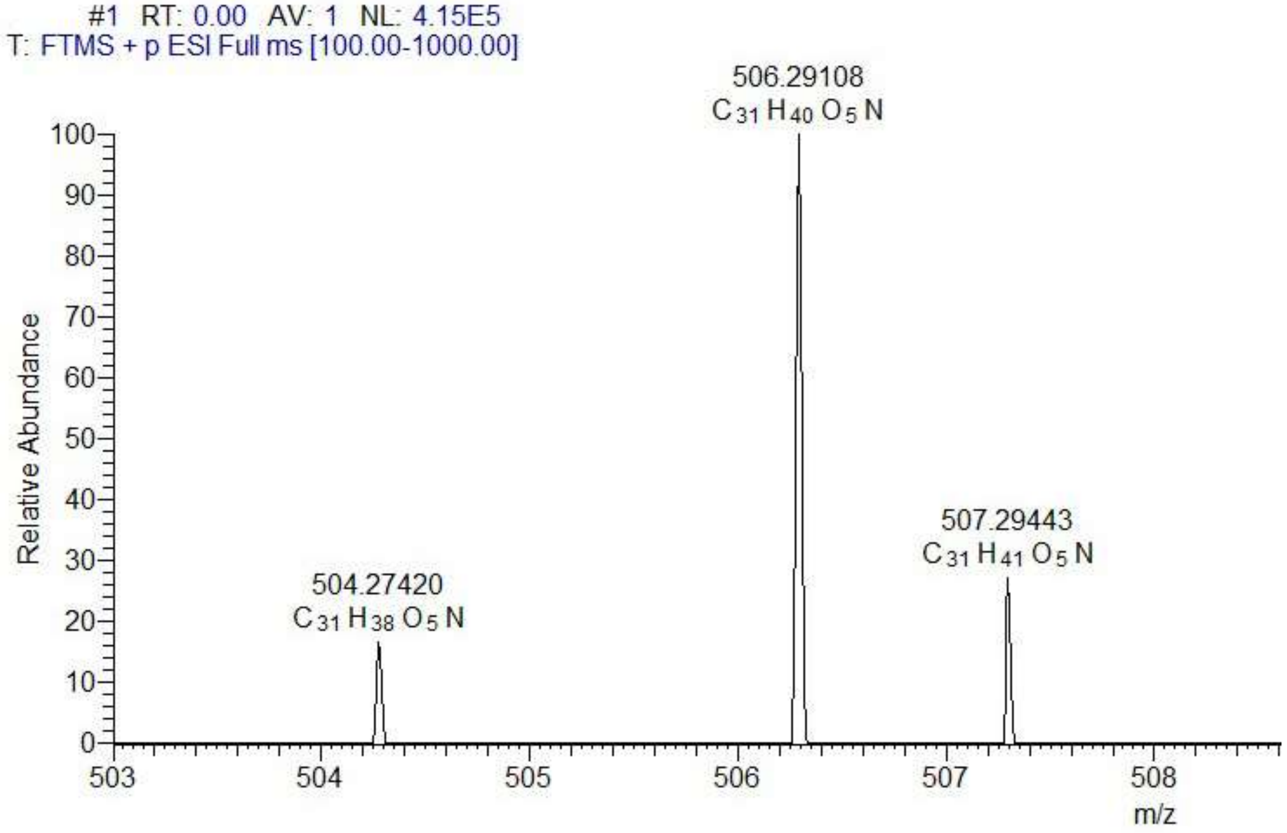

Figure S78. HRMS-ESI spectrum of the target compound (**4e**)

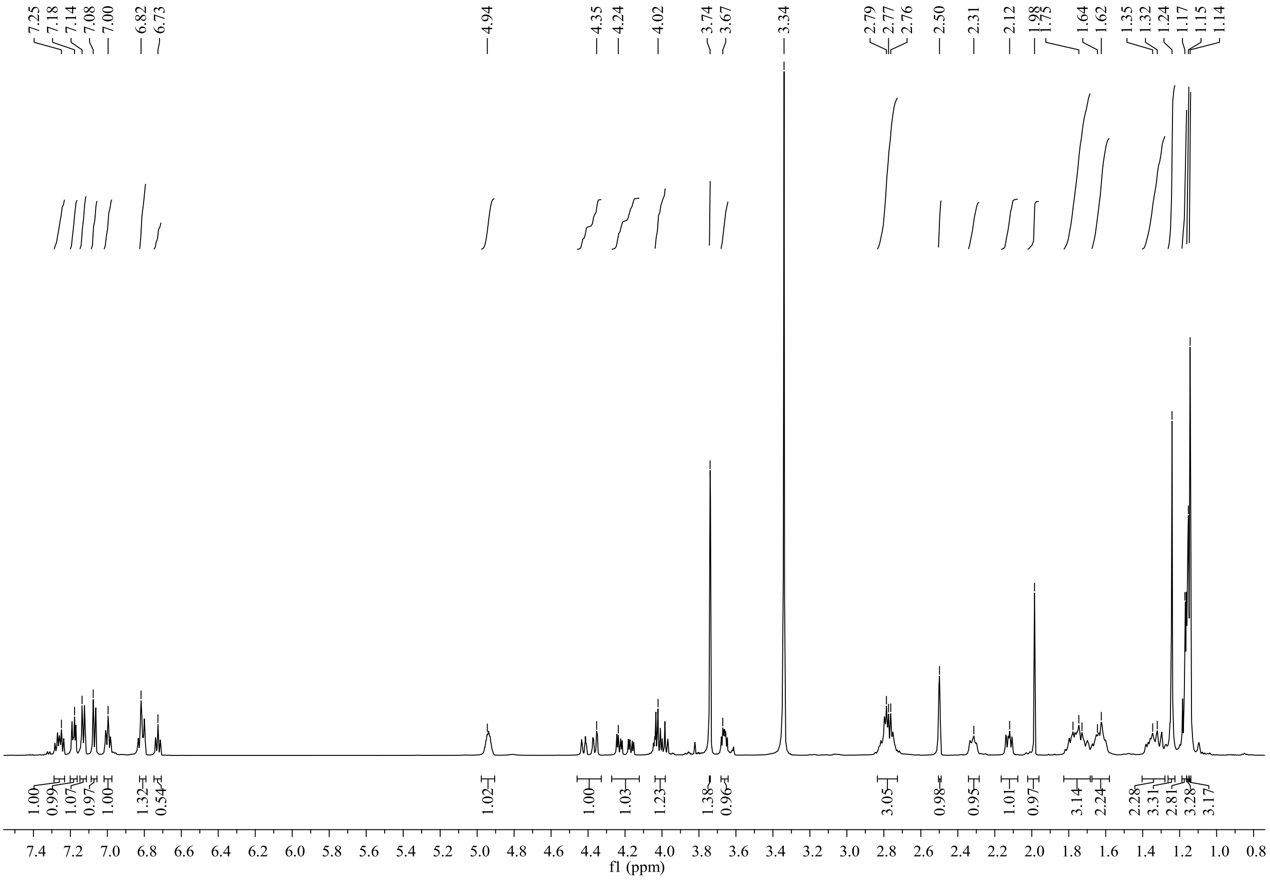

Figure S79. ^{1}H-NMR spectrum of the target compound (**4e**) in DMSO

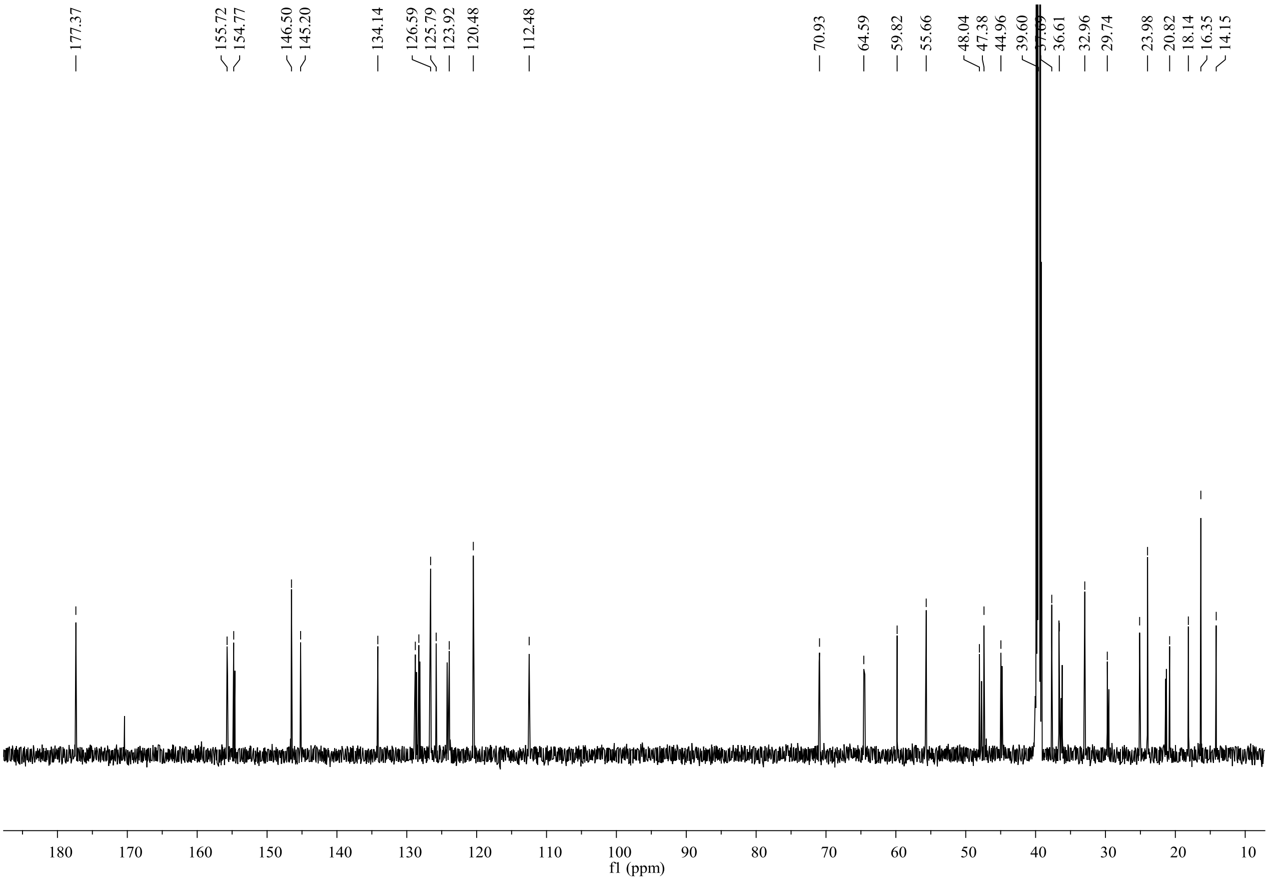

Figure S80. ^{13}C-NMR spectrum of the target compound (**4e**) in DMSO

Int. J. Mol. Sci. 2018, 19, 3116; doi:10.3390/ijms19103116

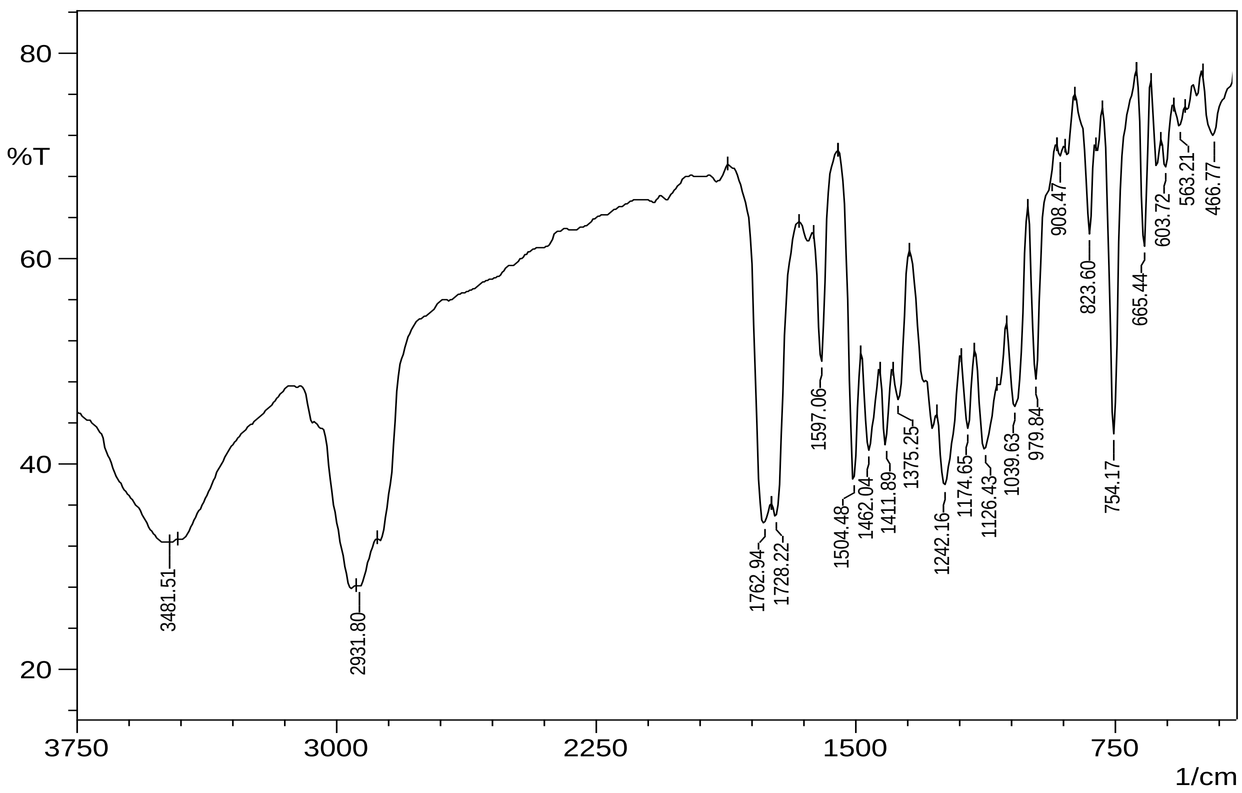

Figure S81. FTIR spectrum of the target compound (**4f**)

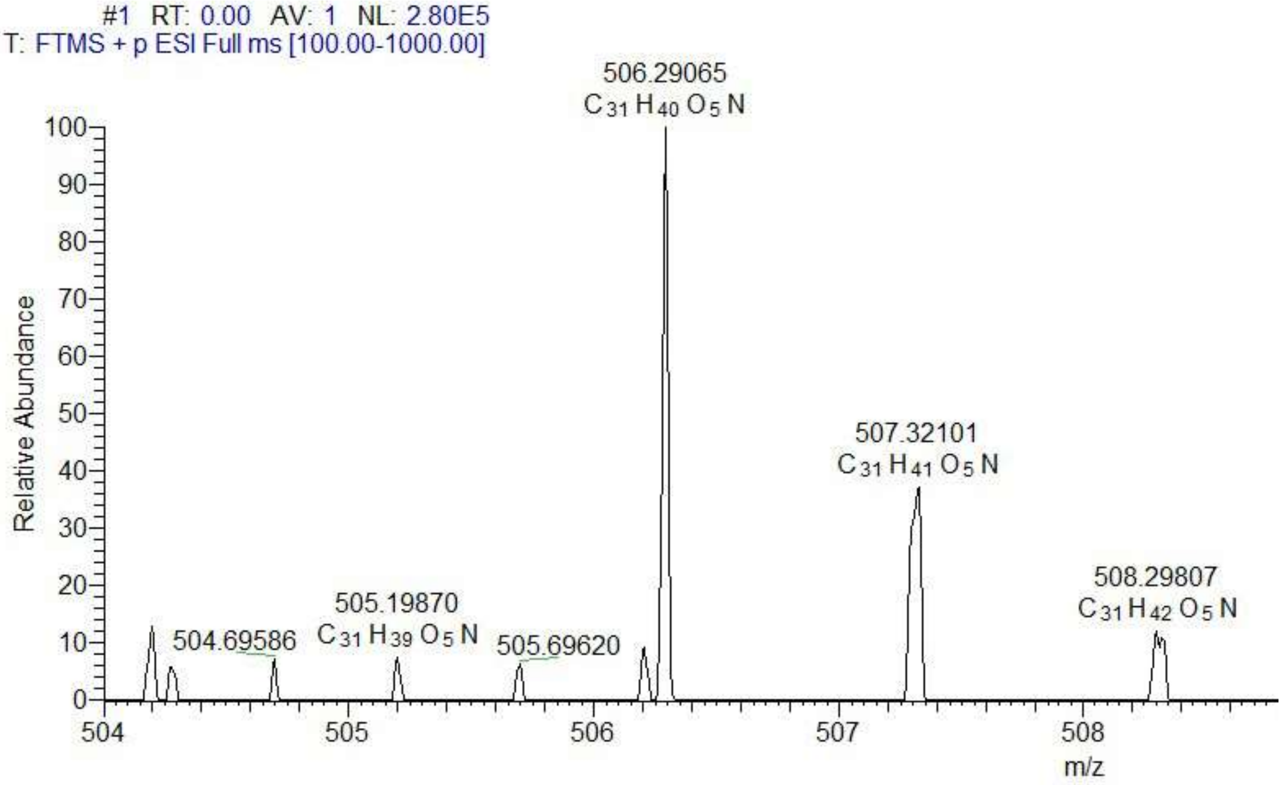

Figure S82. HRMS-ESI spectrum of the target compound (**4f**)

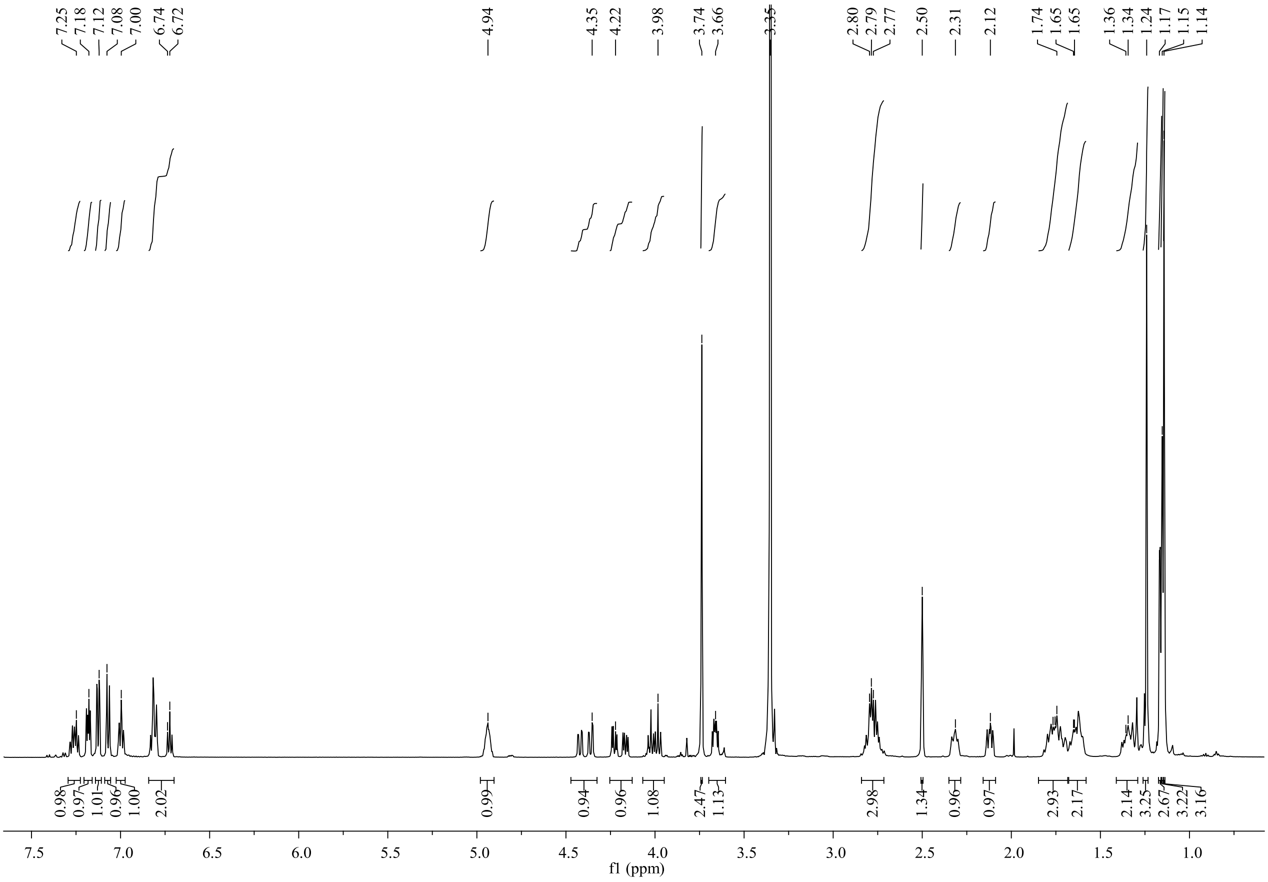

Figure S83. ^{1}H-NMR spectrum of the target compound (**4f**) in DMSO

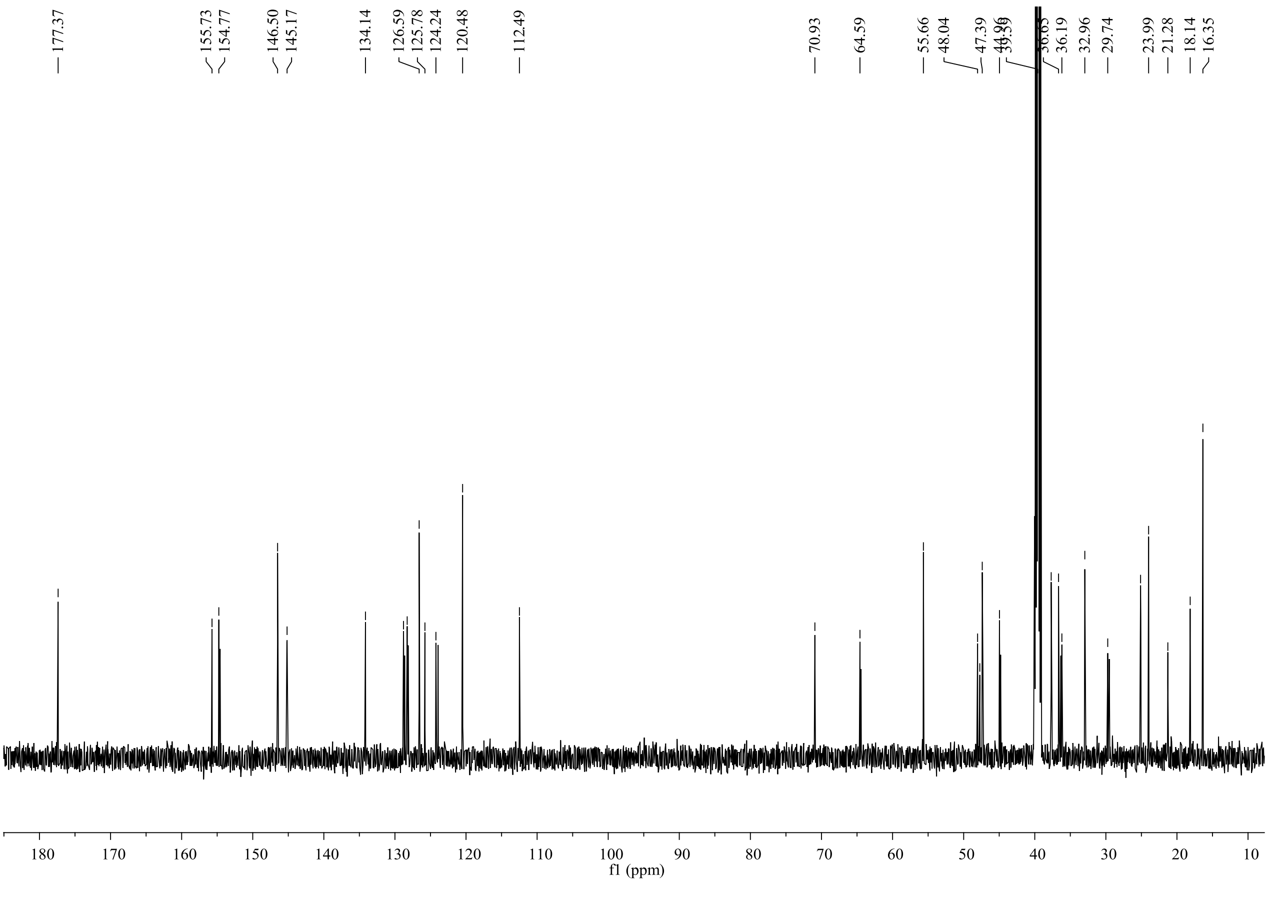

Figure S84. ^{13}C-NMR spectrum of the target compound (**4f**) in DMSO

Int. J. Mol. Sci. 2018, 19, 3116; doi:10.3390/ijms19103116

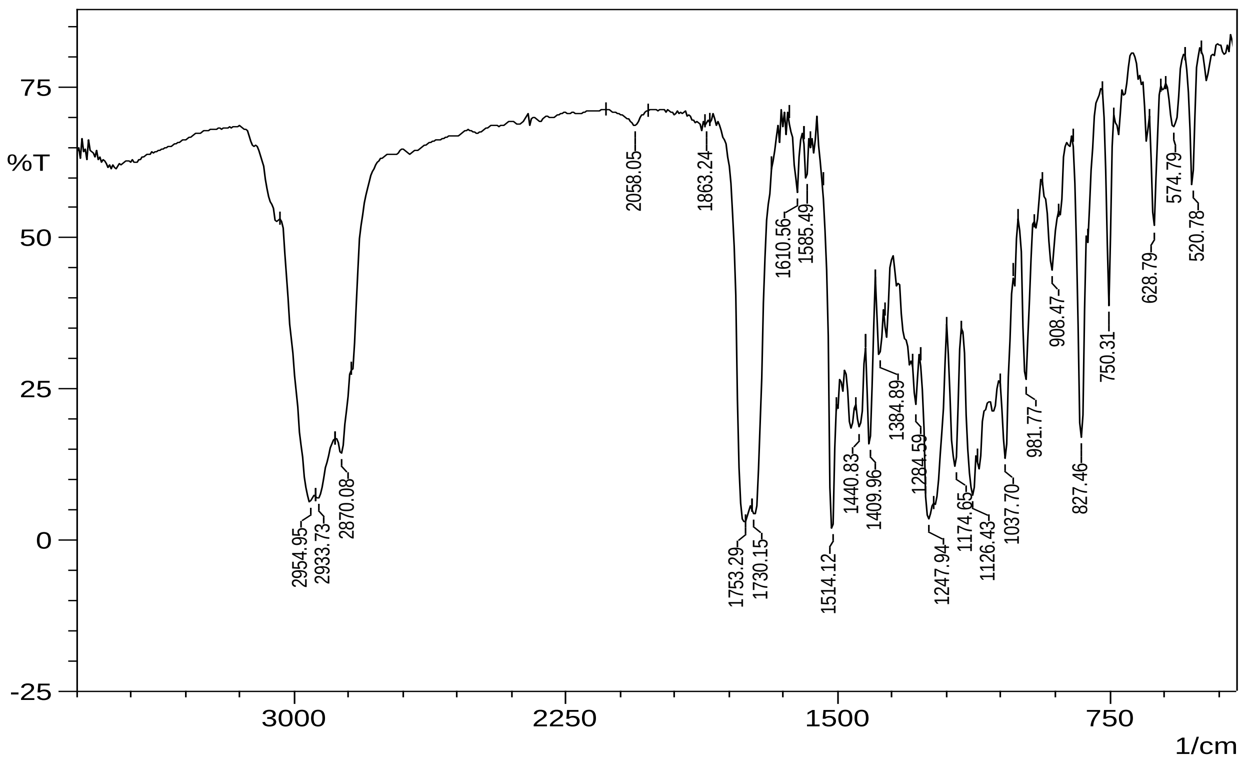

Figure S85. FTIR spectrum of the target compound (**4g**)

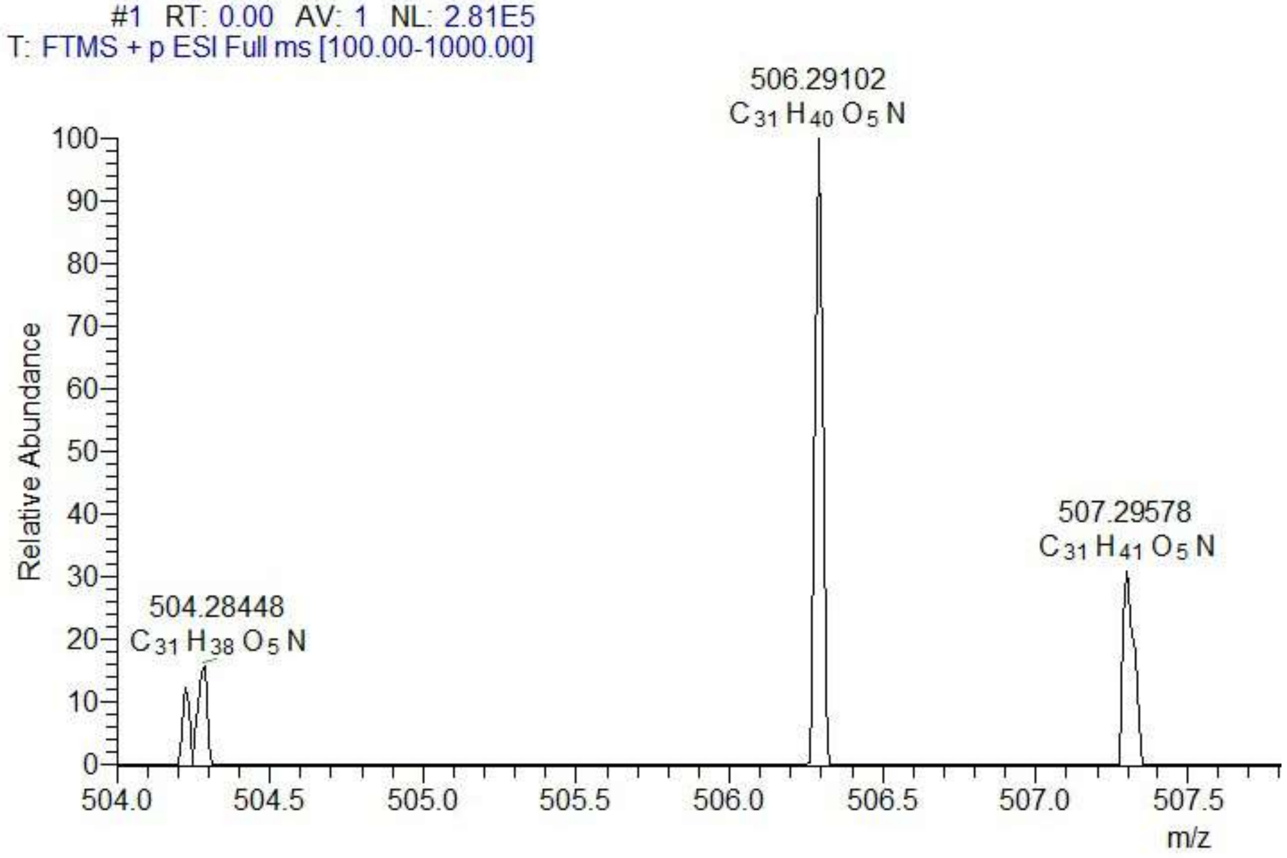

Figure S86. HRMS-ESI spectrum of the target compound (**4g**)

Int. J. Mol. Sci. 2018, 19, 3116; doi:10.3390/ijms19103116

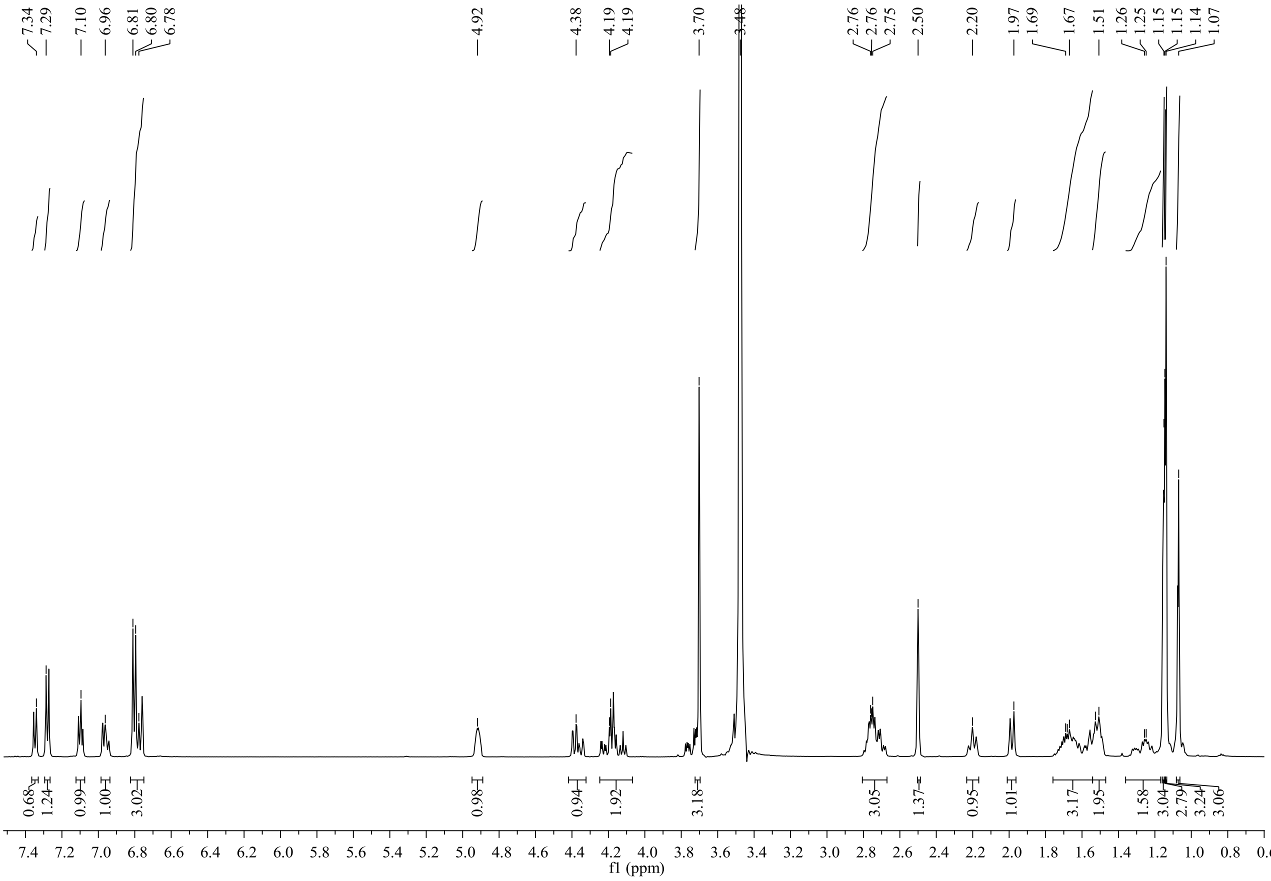

Figure S87. ^{1}H-NMR spectrum of the target compound (**4g**) in DMSO

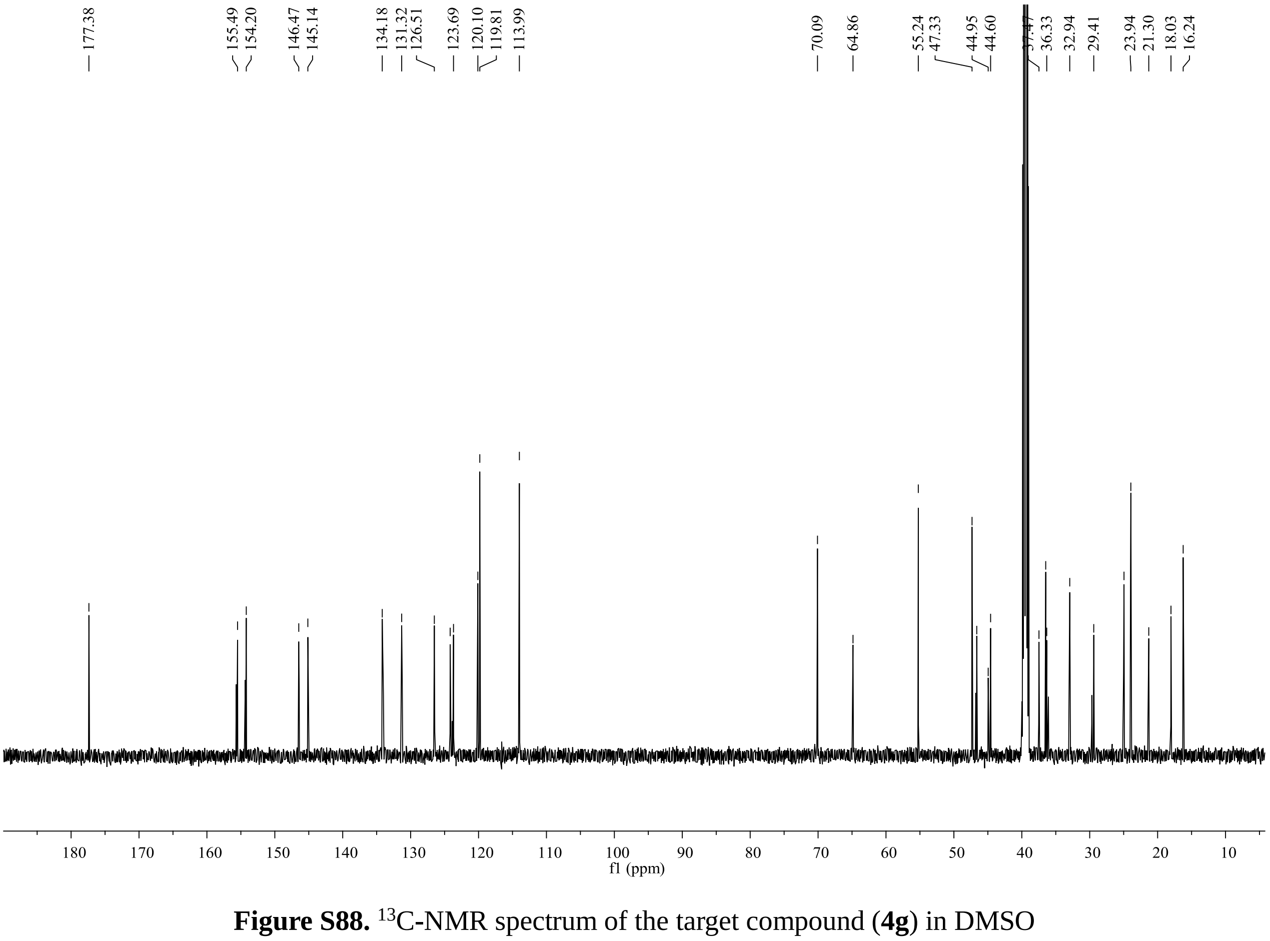

Figure S88. ^{13}C-NMR spectrum of the target compound (**4g**) in DMSO

Int. J. Mol. Sci. 2018, 19, 3116; doi:10.3390/ijms19103116

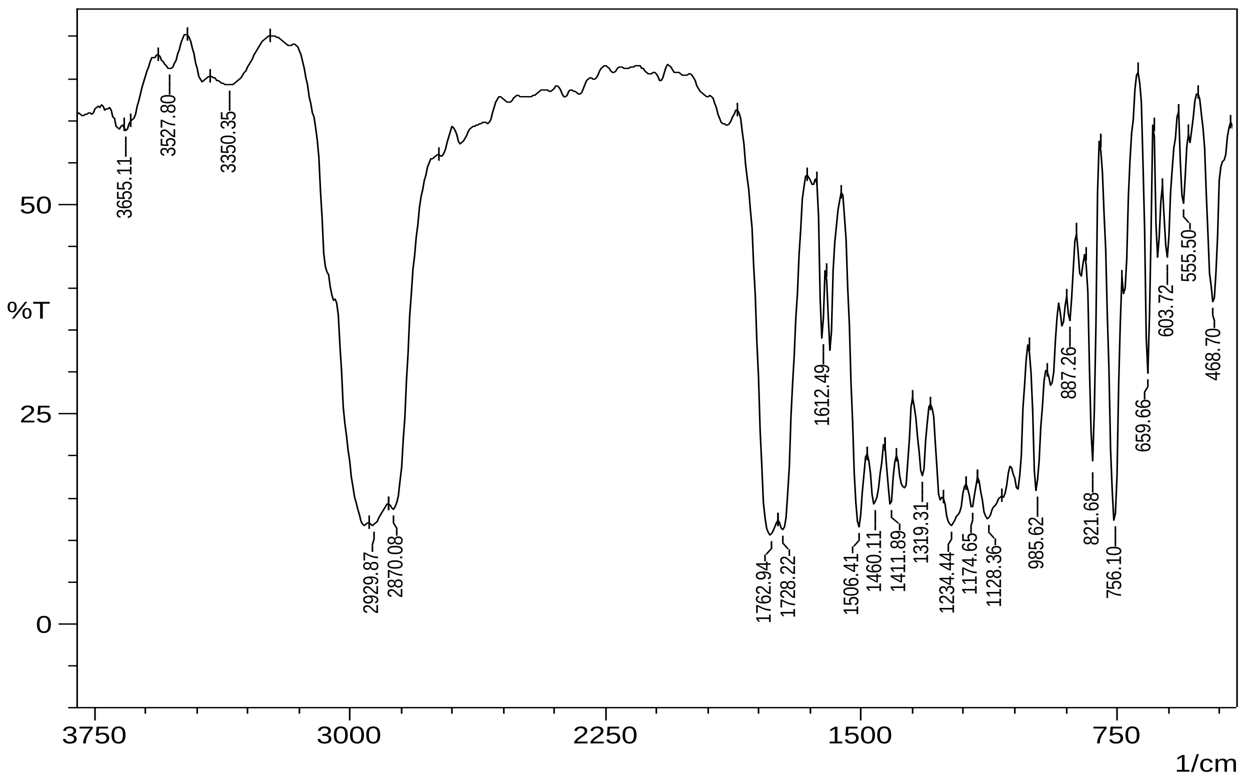

Figure S89. FTIR spectrum of the target compound (**4h**)

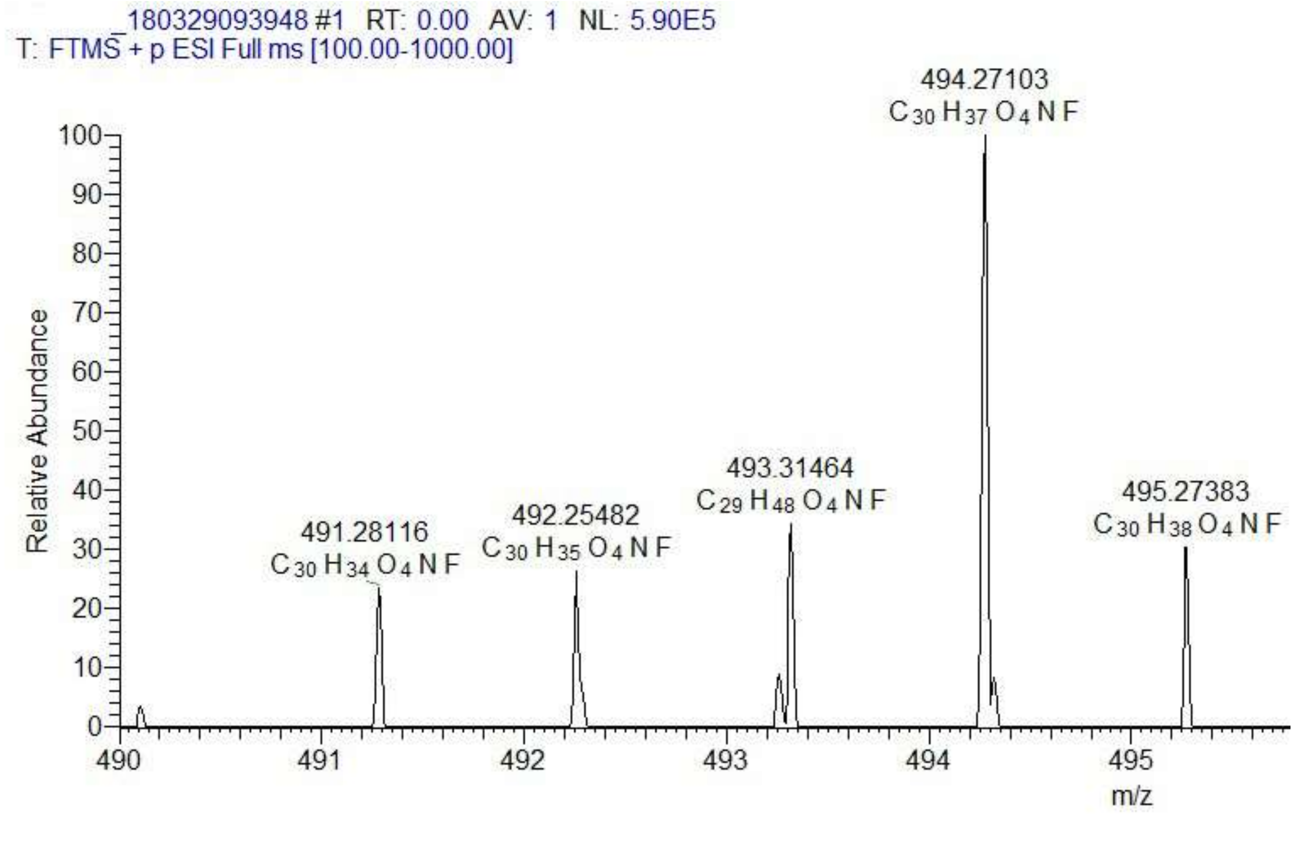

Figure S90. HRMS-ESI spectrum of the target compound (**4h**)

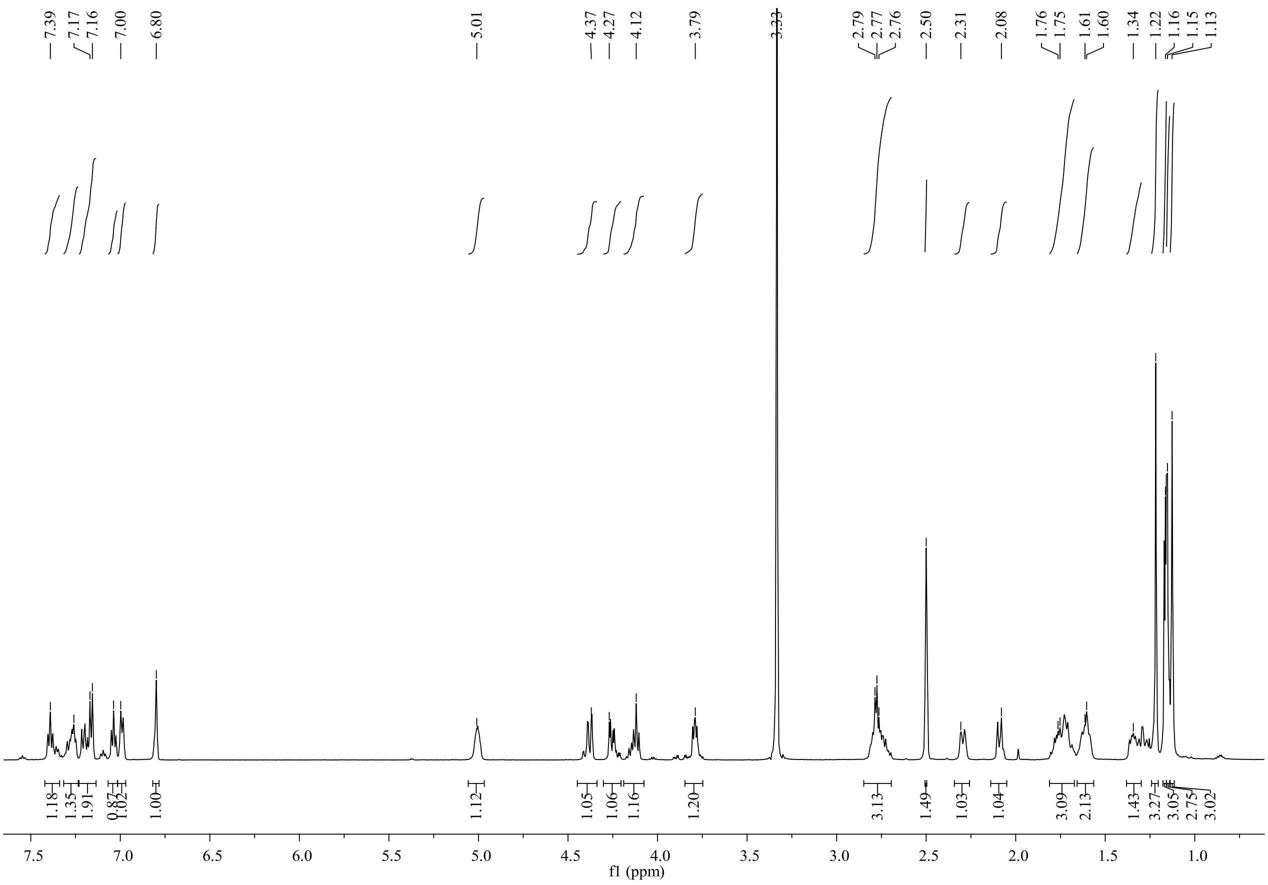

Figure S91. ^{1}H-NMR spectrum of the target compound (**4h**) in DMSO

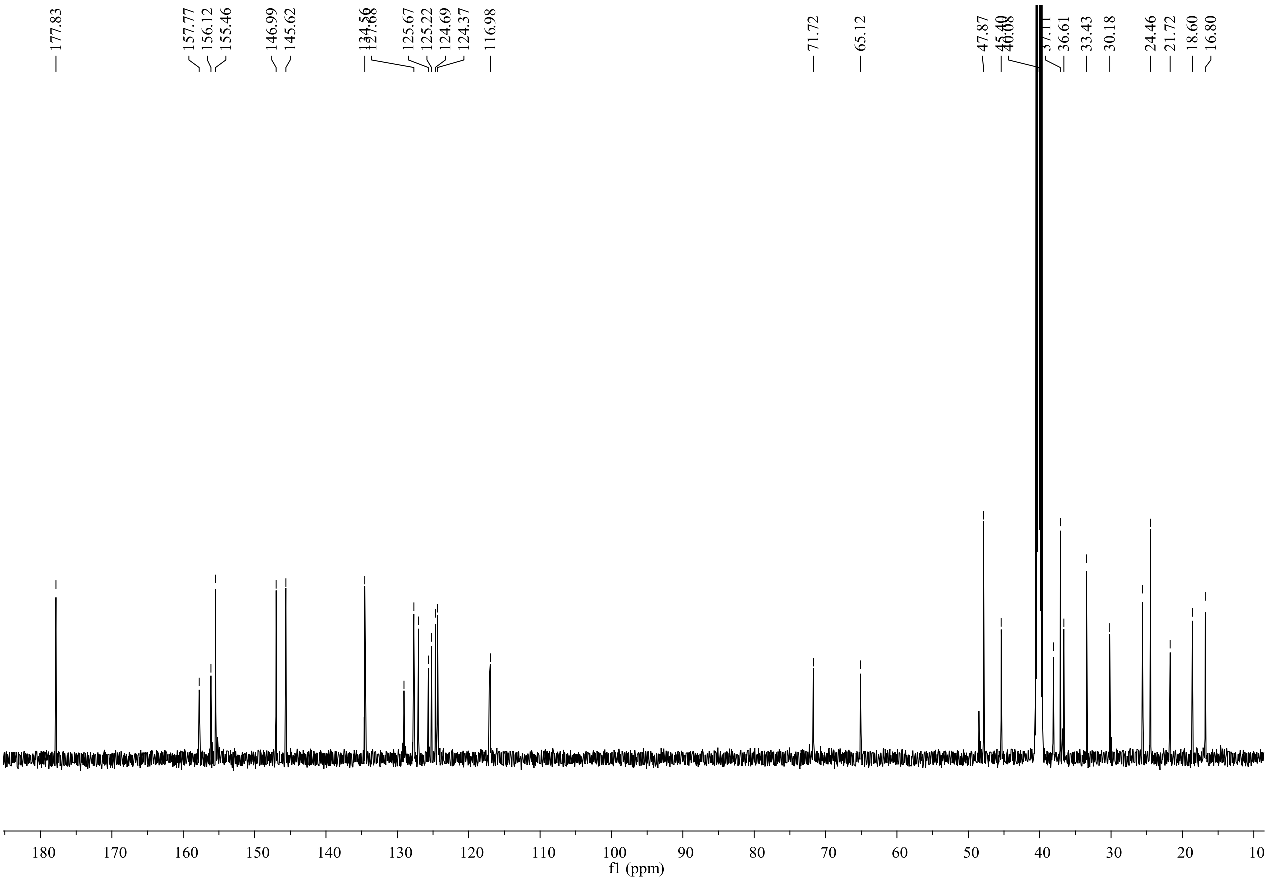

Figure S92. ^{13}C-NMR spectrum of the target compound (**4h**) in DMSO

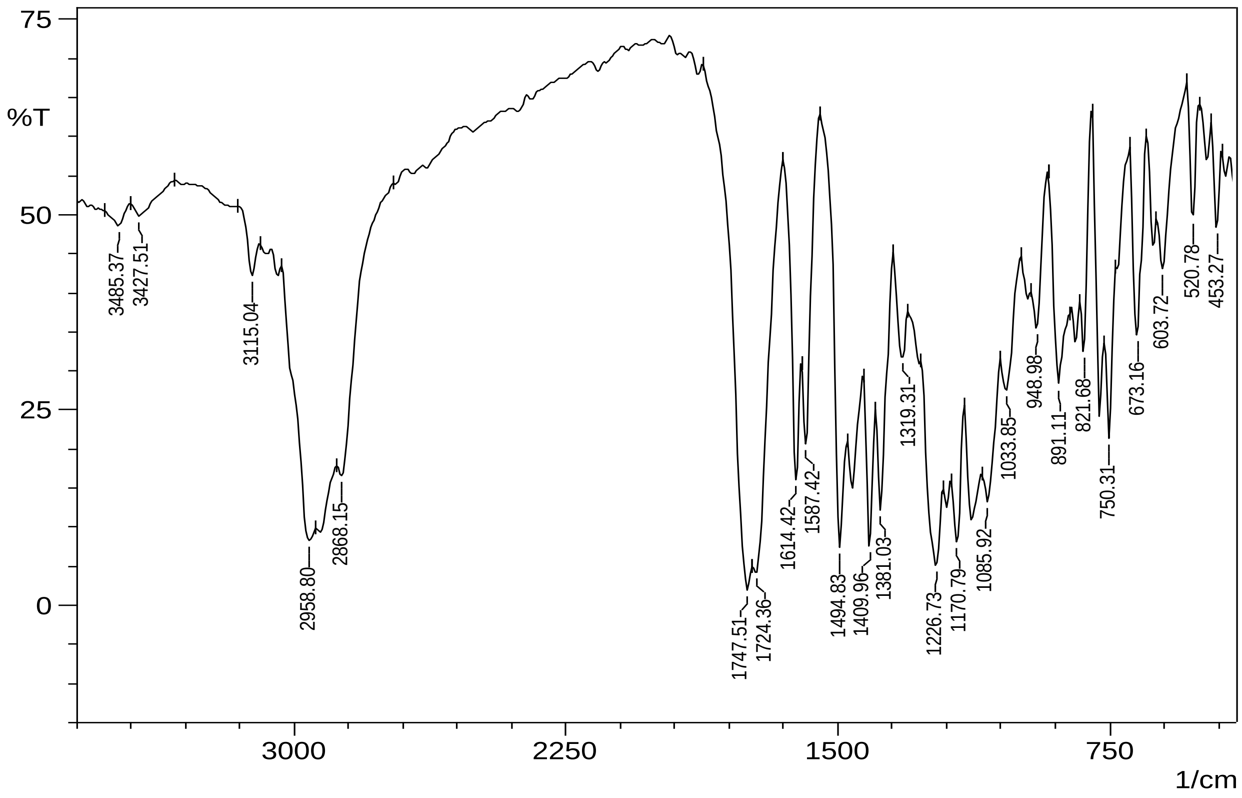

Figure S93. FTIR spectrum of the target compound (**4i**)

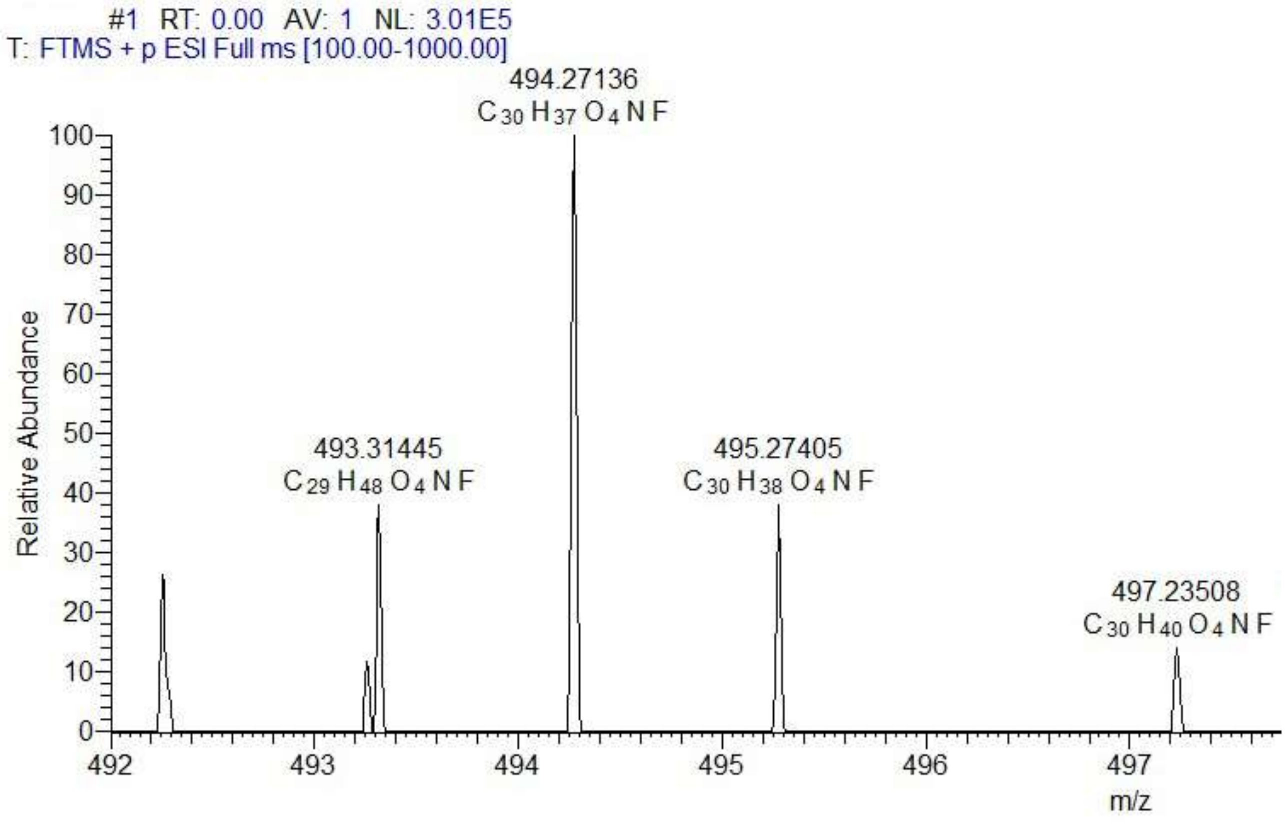

Figure S94. HRMS-ESI spectrum of the target compound (**4i**)

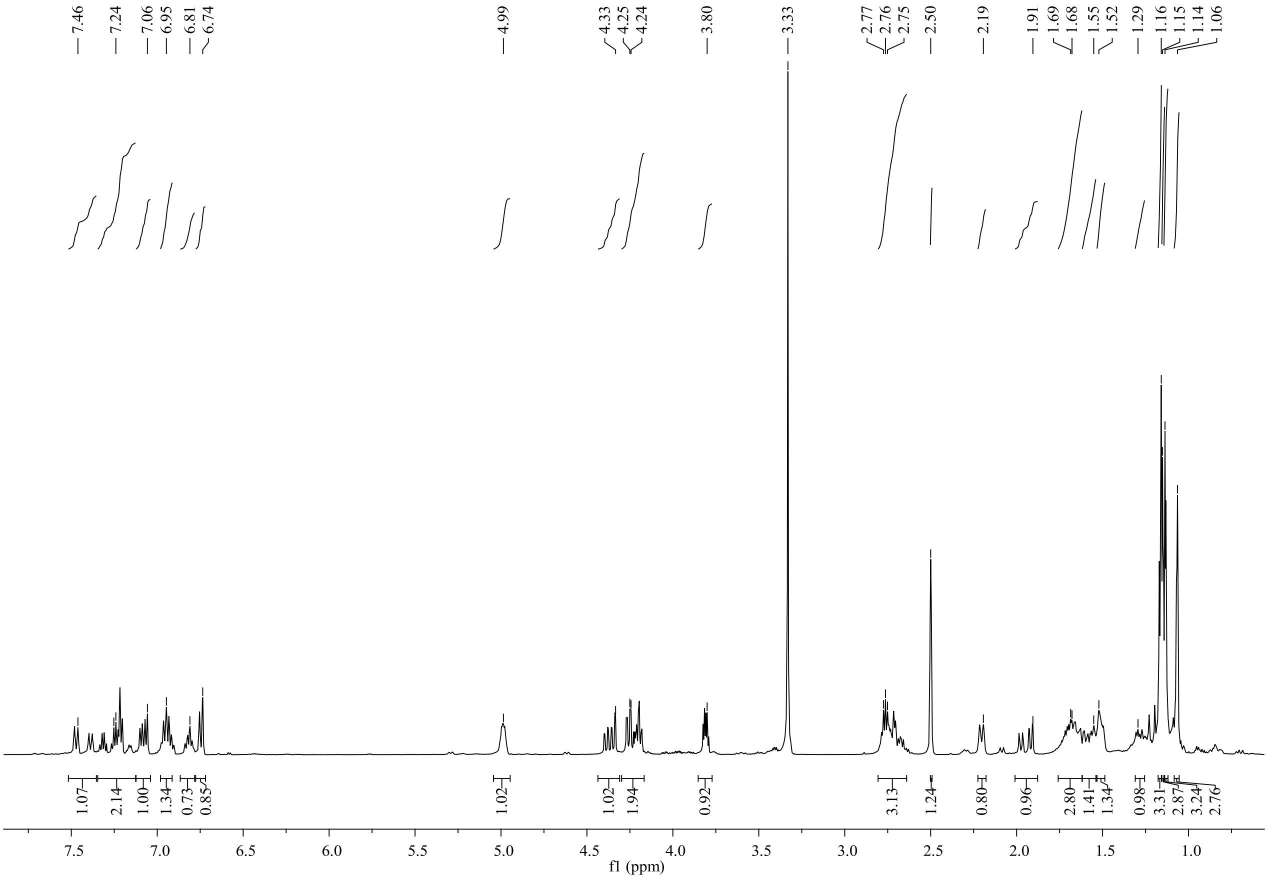

Figure S95. ^{1}H-NMR spectrum of the target compound (**4i**) in DMSO

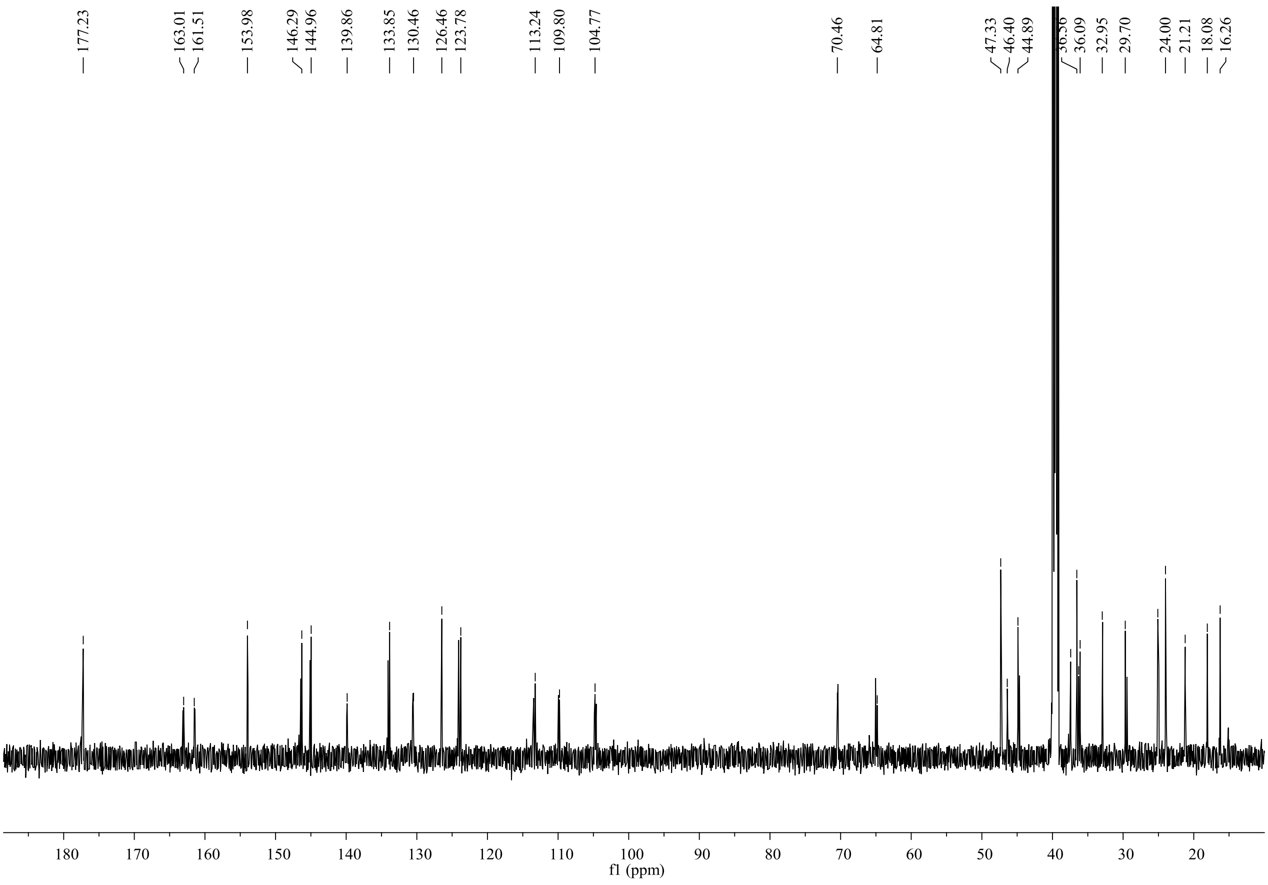

Figure S96. ^{13}C-NMR spectrum of the target compound (**4i**) in DMSO

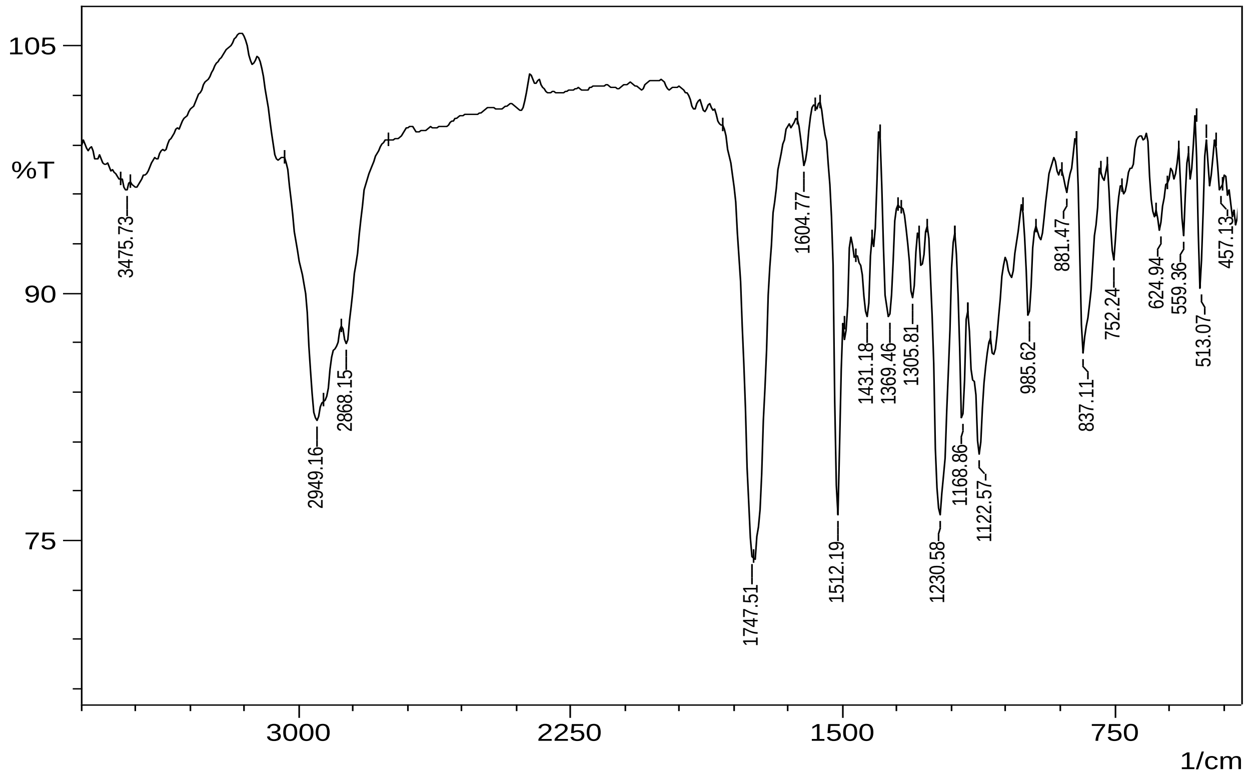

Figure S97. FTIR spectrum of the target compound (**4j**)

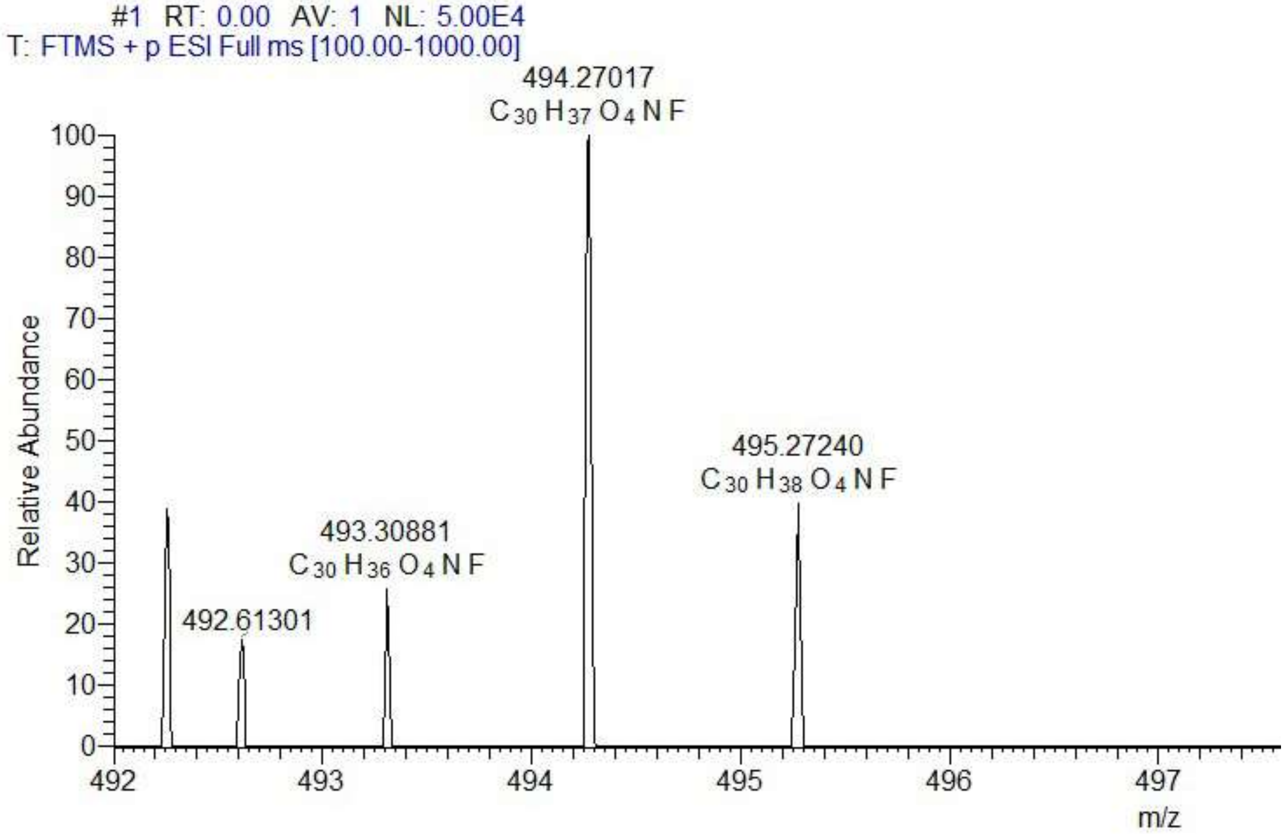

Figure S98. HRMS-ESI spectrum of the target compound (**4j**)

Int. J. Mol. Sci. 2018, 19, 3116; doi:10.3390/ijms19103116

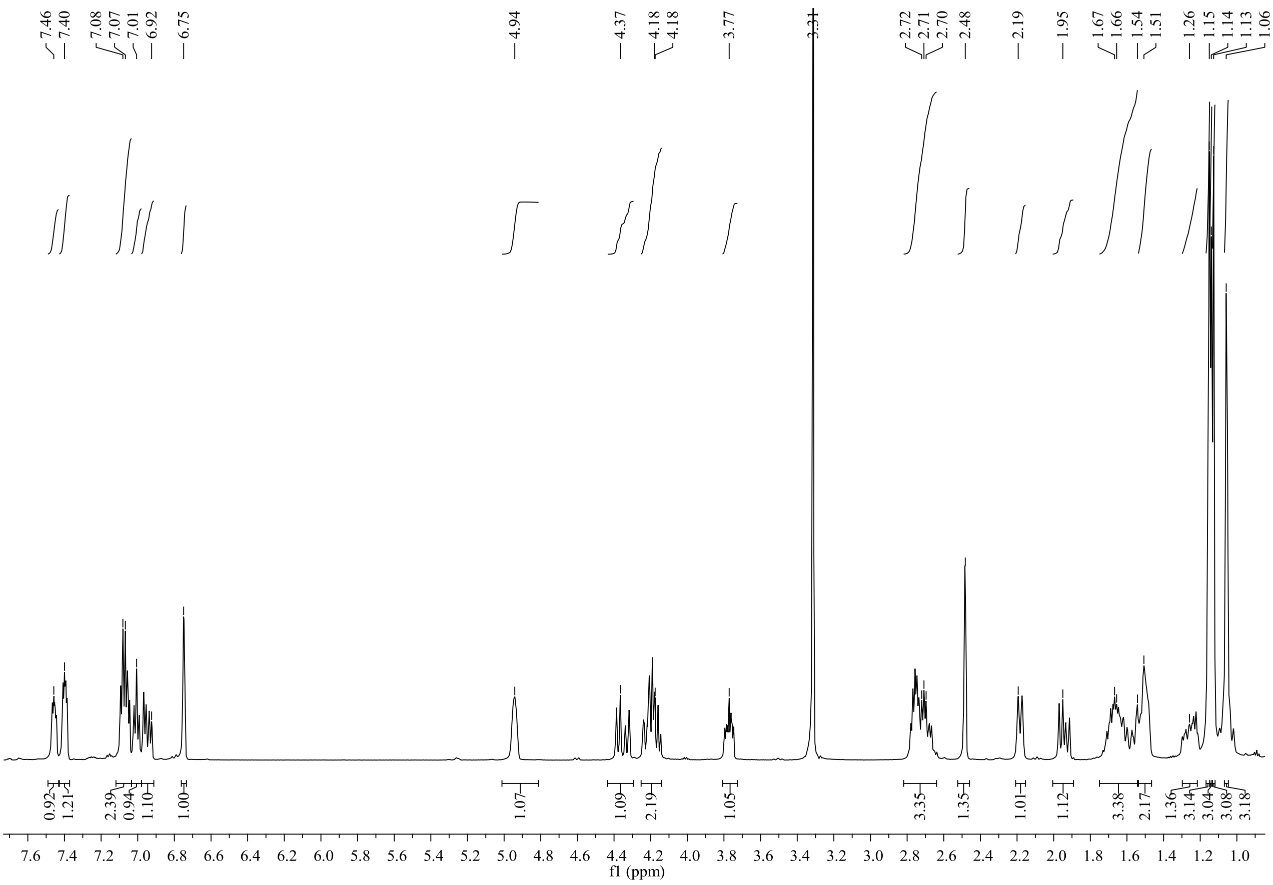

Figure S99. ^{1}H-NMR spectrum of the target compound (**4j**) in DMSO

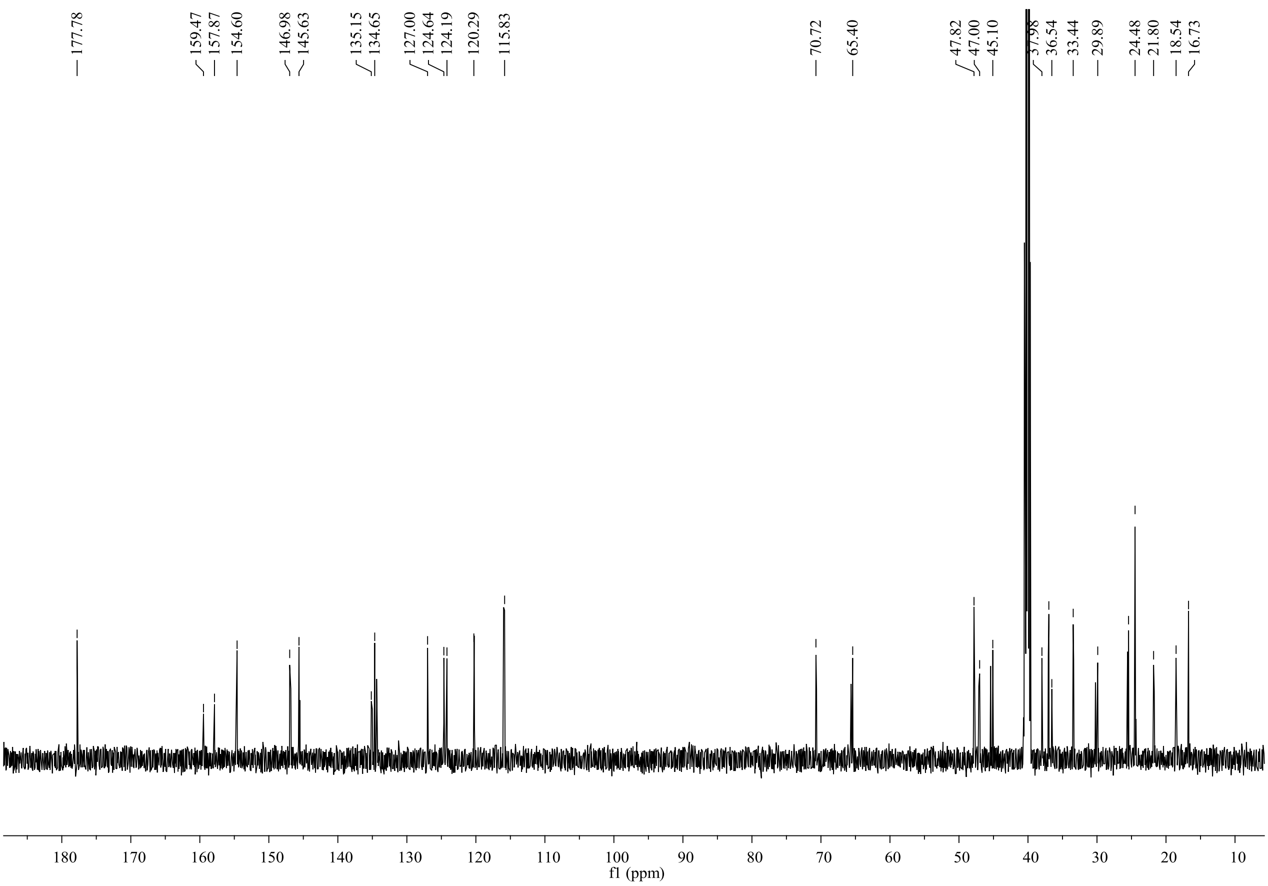

Figure S100. ^{13}C-NMR spectrum of the target compound (**4j**) in DMSO

Int. J. Mol. Sci. 2018, 19, 3116; doi:10.3390/ijms19103116

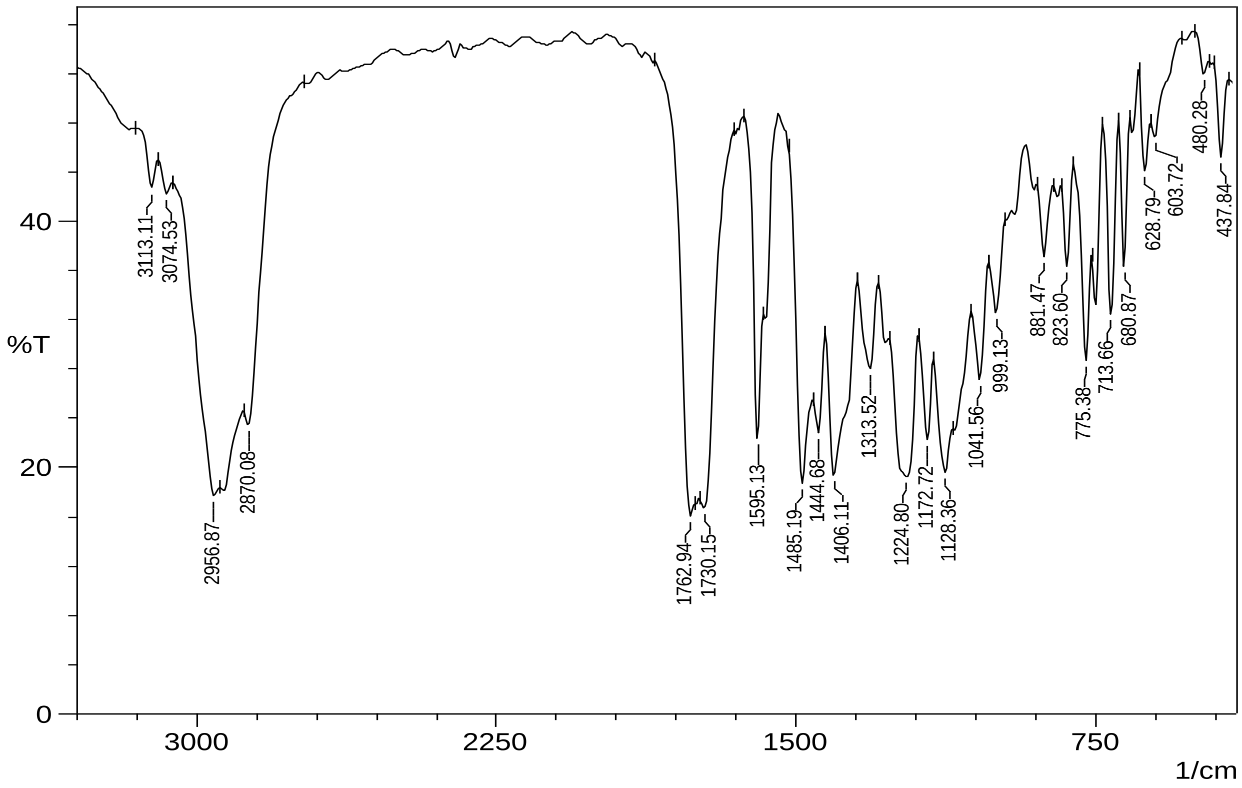

Figure S101. FTIR spectrum of the target compound (**4k**)

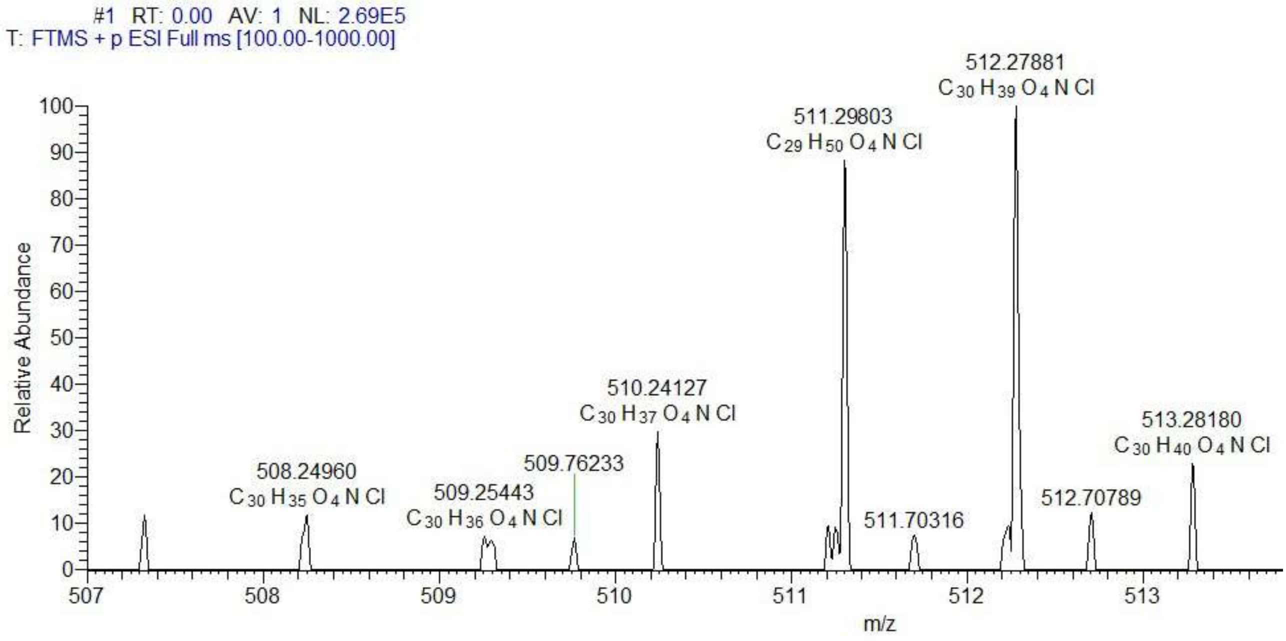

Figure S102. HRMS-ESI spectrum of the target compound (**4k**)

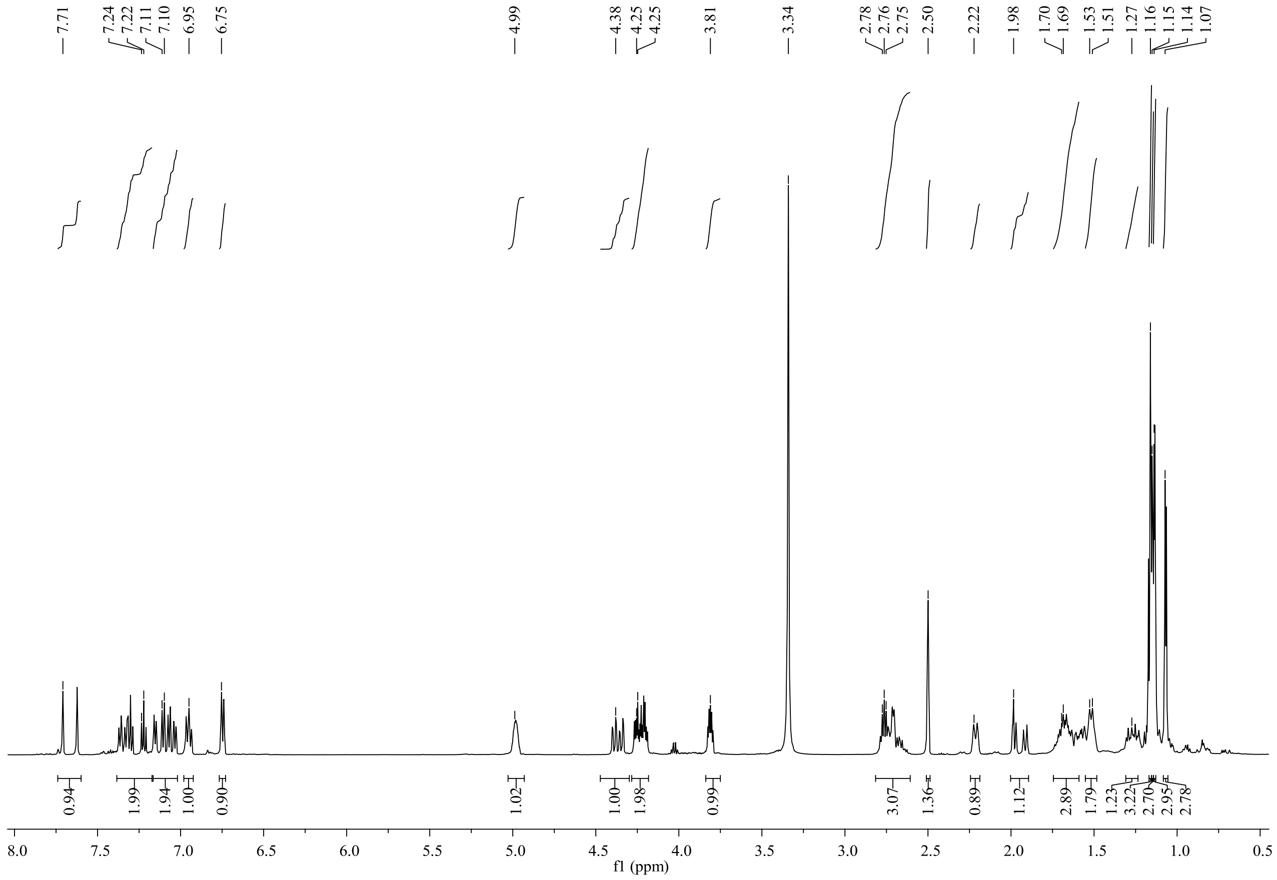

Figure S103. ^{1}H-NMR spectrum of the target compound (**4k**) in DMSO

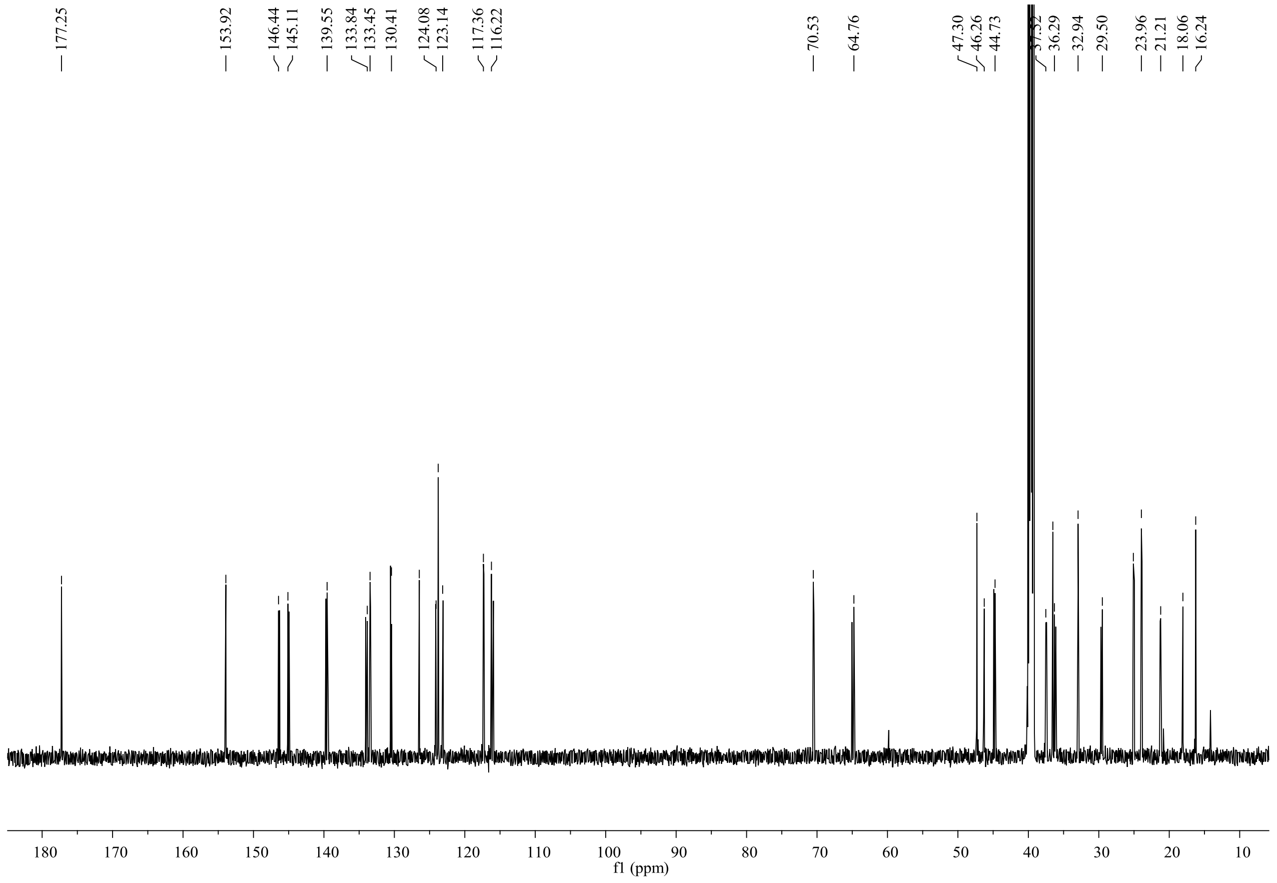

Figure S104. ^{13}C-NMR spectrum of the target compound (**4k**) in DMSO

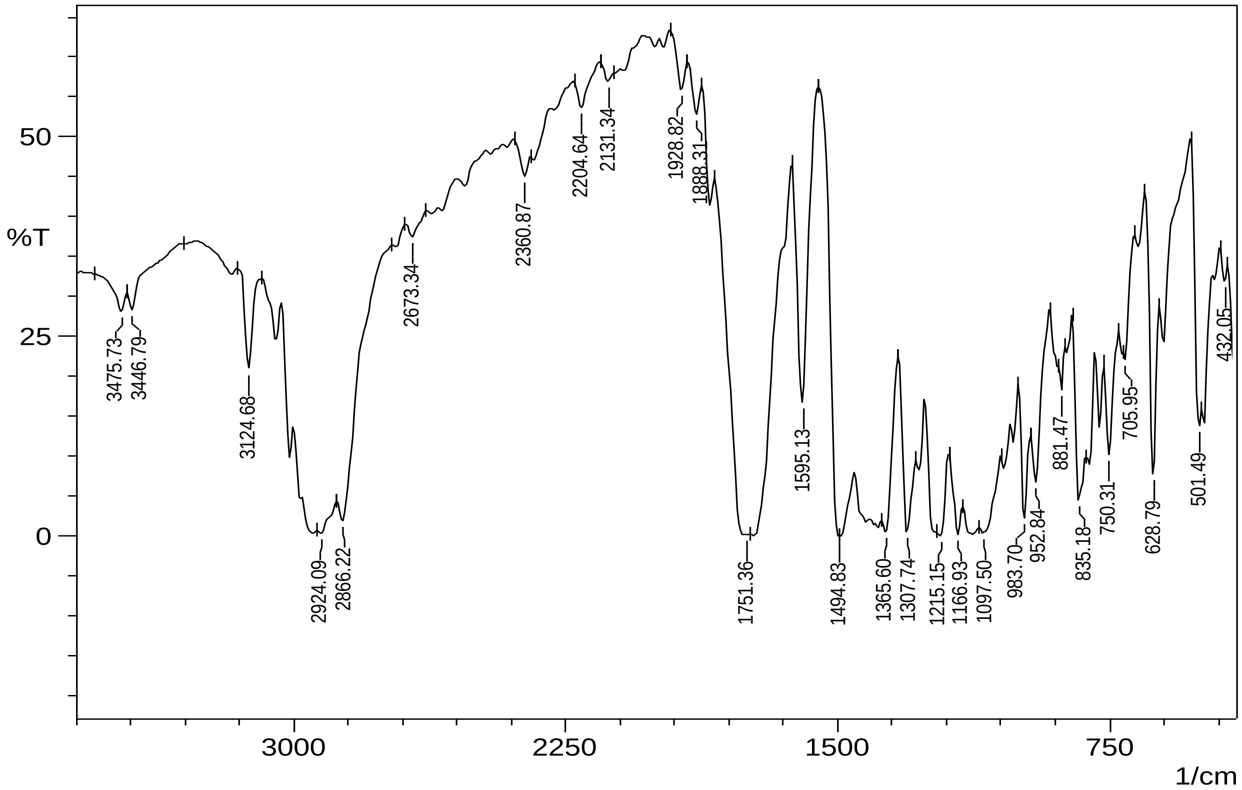

Figure S105. FTIR spectrum of the target compound (**4l**)

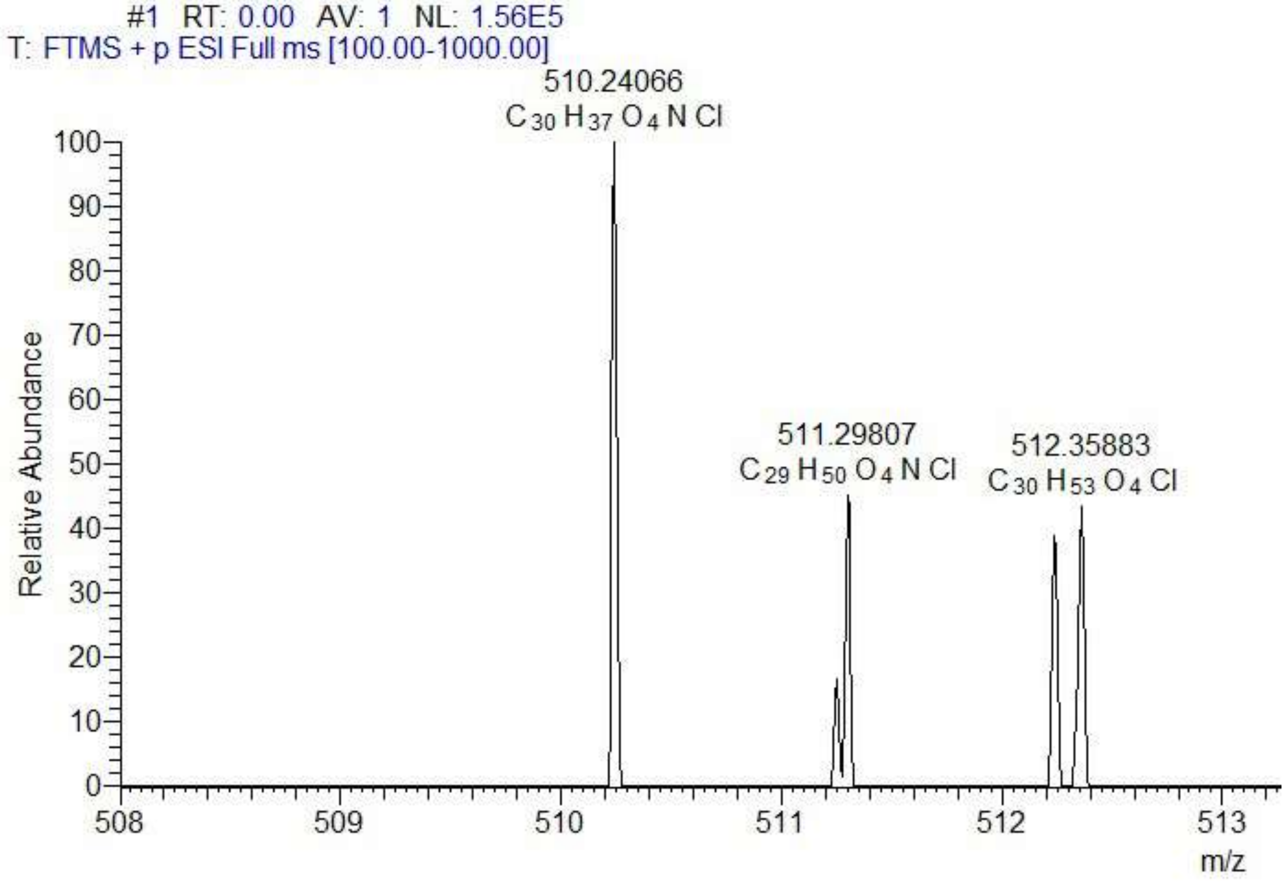

Figure S106. HRMS-ESI spectrum of the target compound (**4l**)

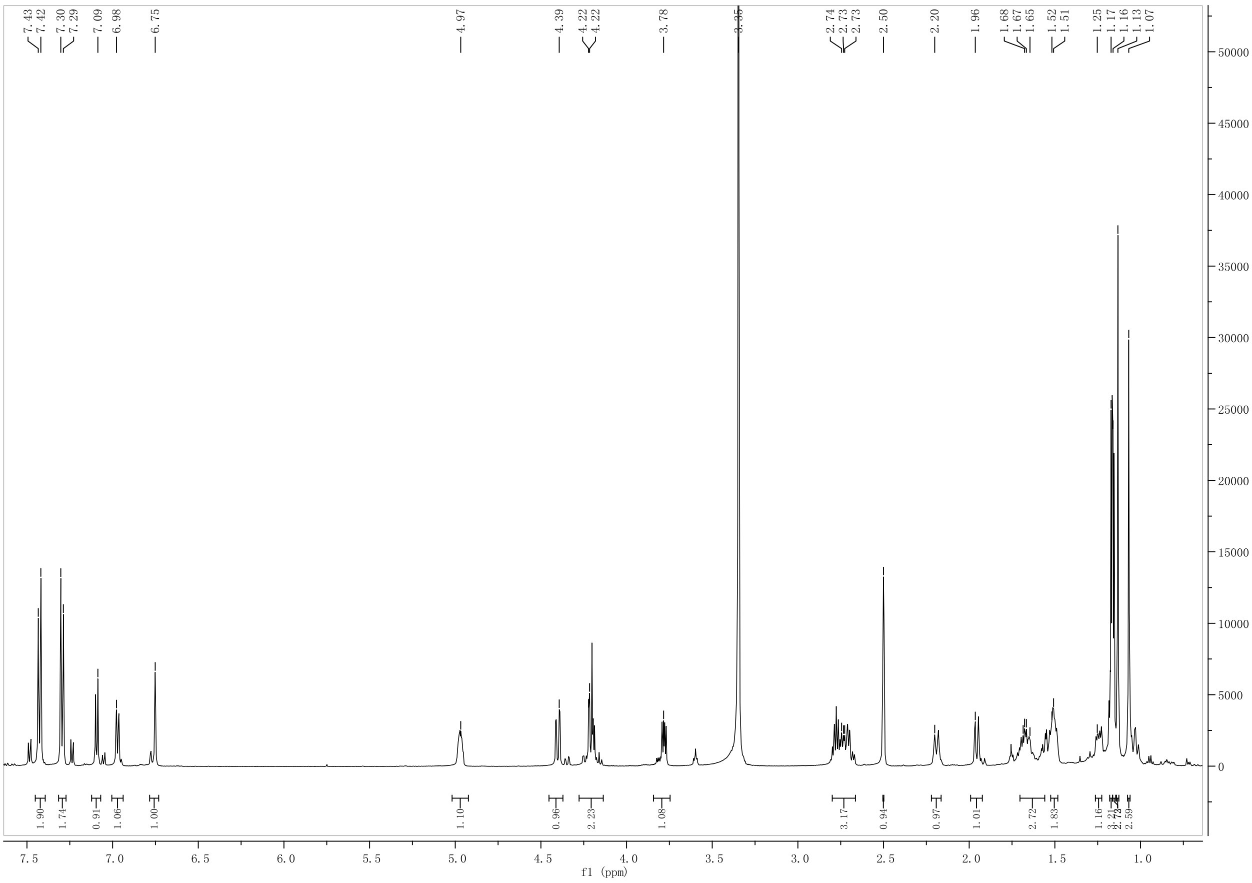

Figure S107. ^{1}H-NMR spectrum of the target compound (**4l**) in DMSO

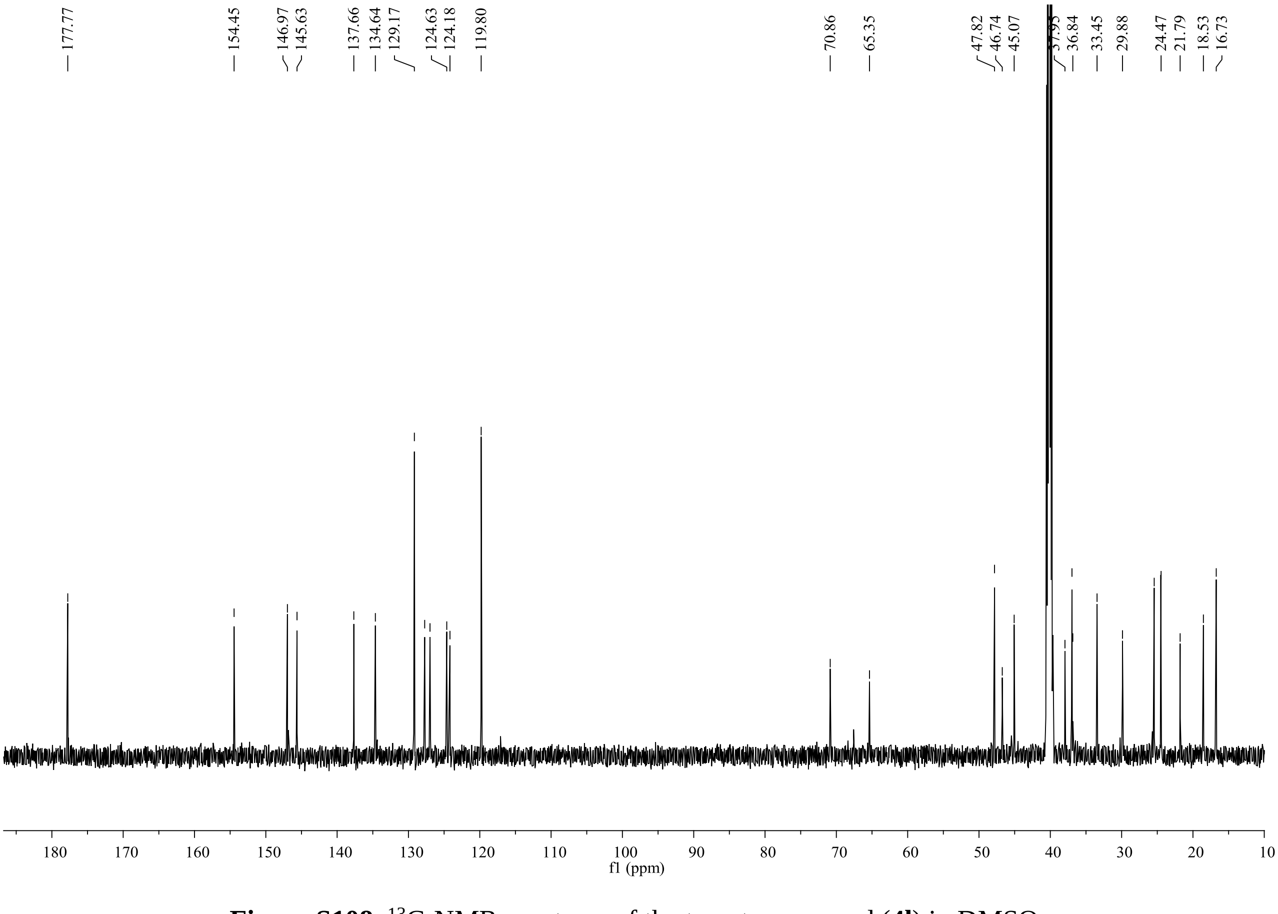

Figure S108. ^{13}C-NMR spectrum of the target compound (**4l**) in DMSO

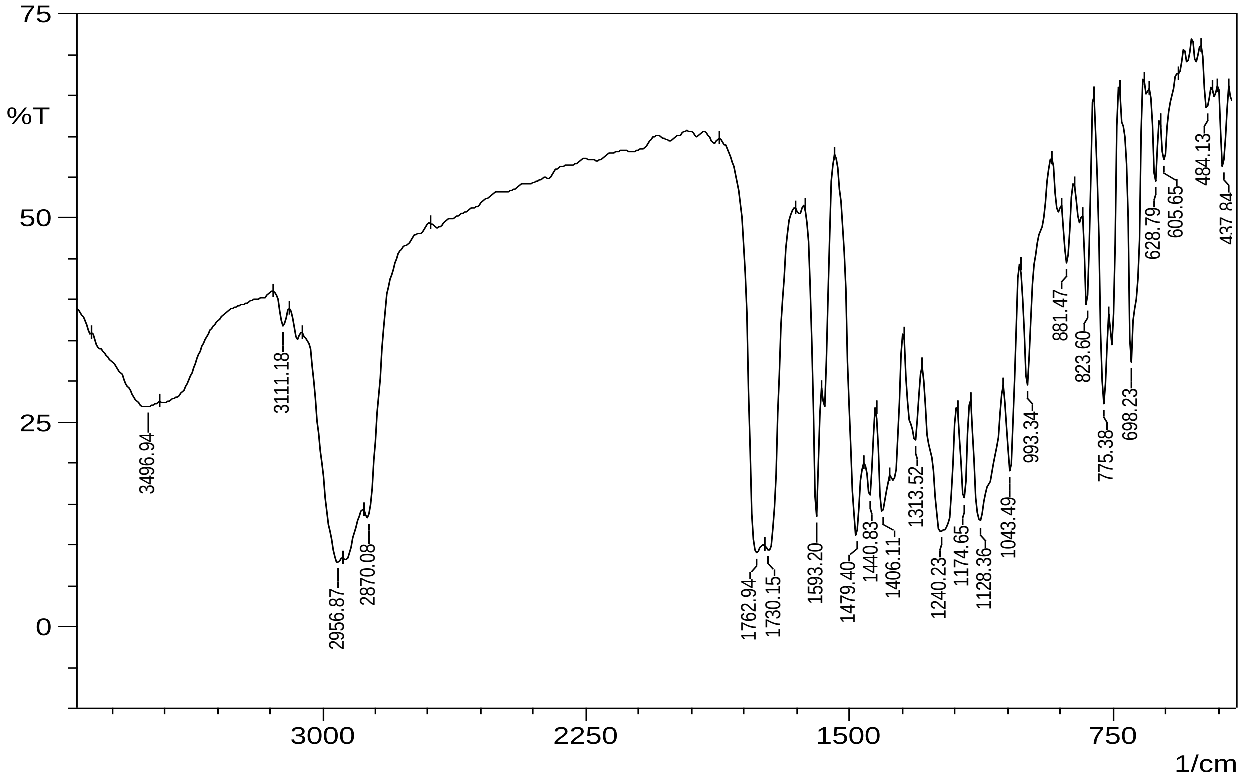

Figure S109. FTIR spectrum of the target compound (**4m**)

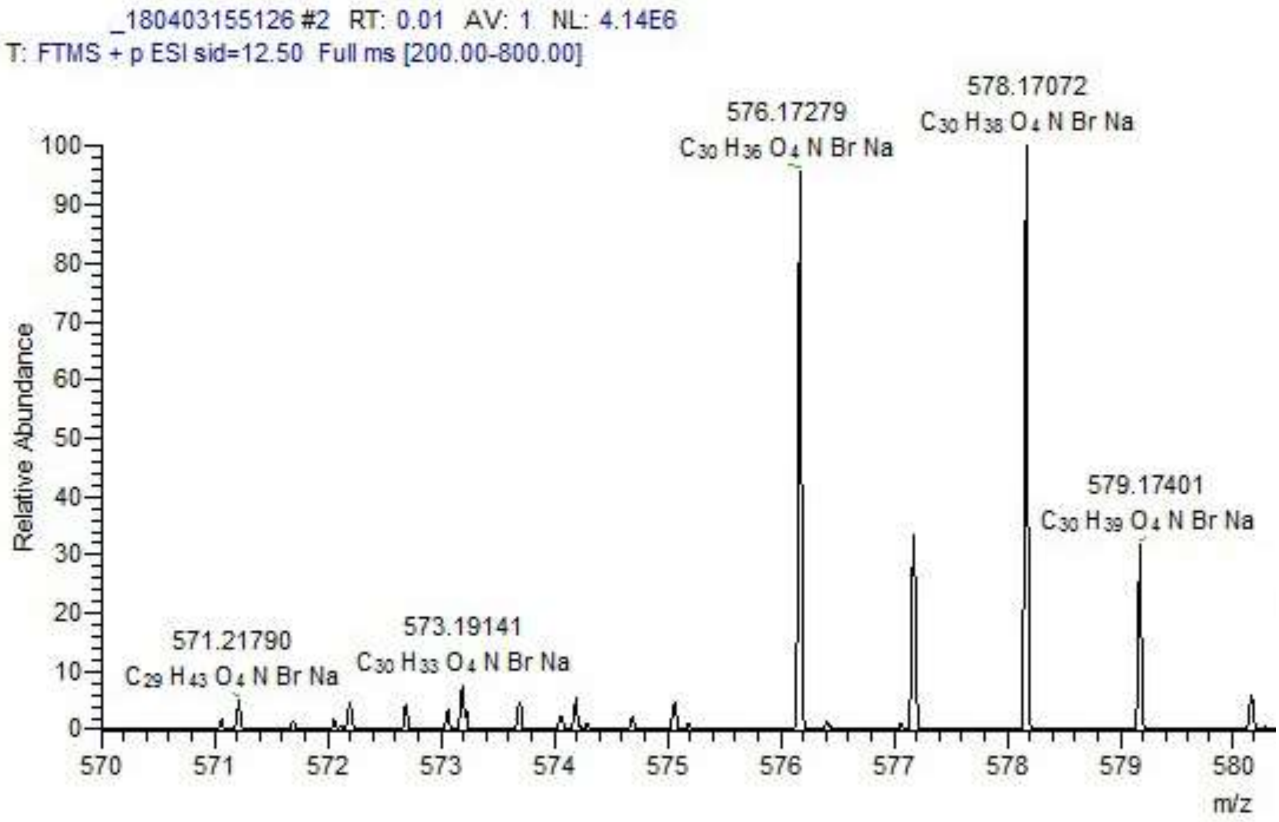

Figure S110. HRMS-ESI spectrum of the target compound (**4m**)

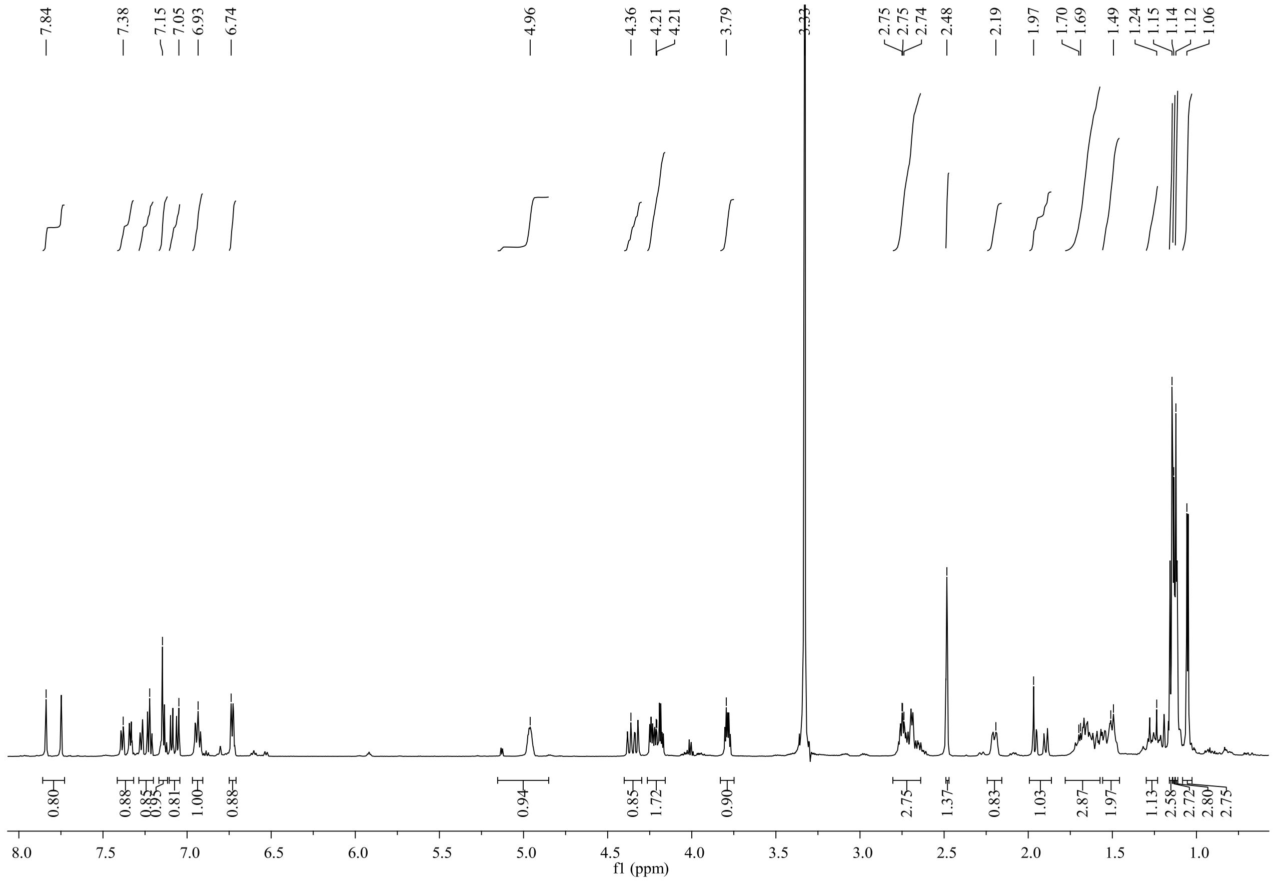

Figure S111. ^{1}H-NMR spectrum of the target compound (**4m**) in DMSO

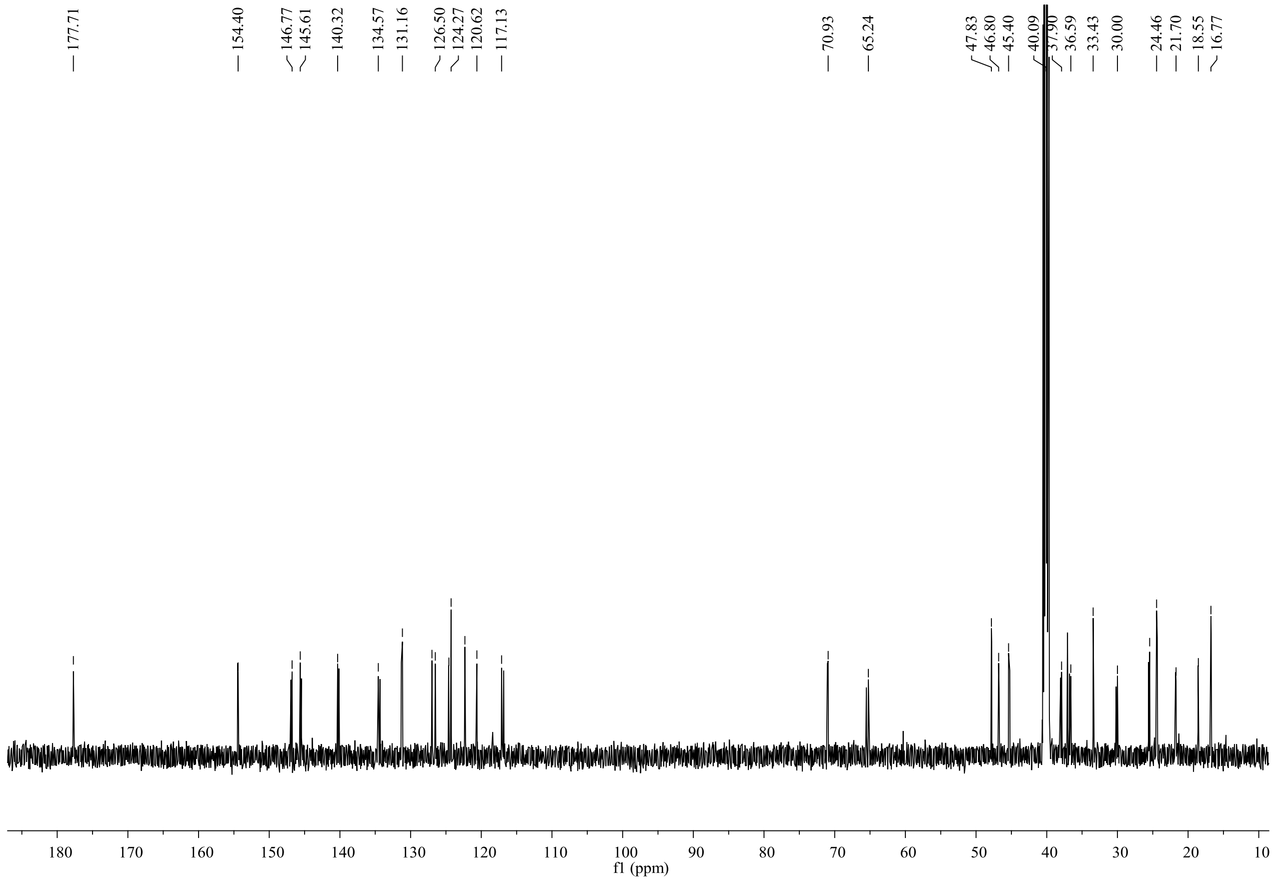

Figure S112. ^{13}C-NMR spectrum of the target compound (**4m**) in DMSO

Int. J. Mol. Sci. 2018, 19, 3116; doi:10.3390/ijms19103116

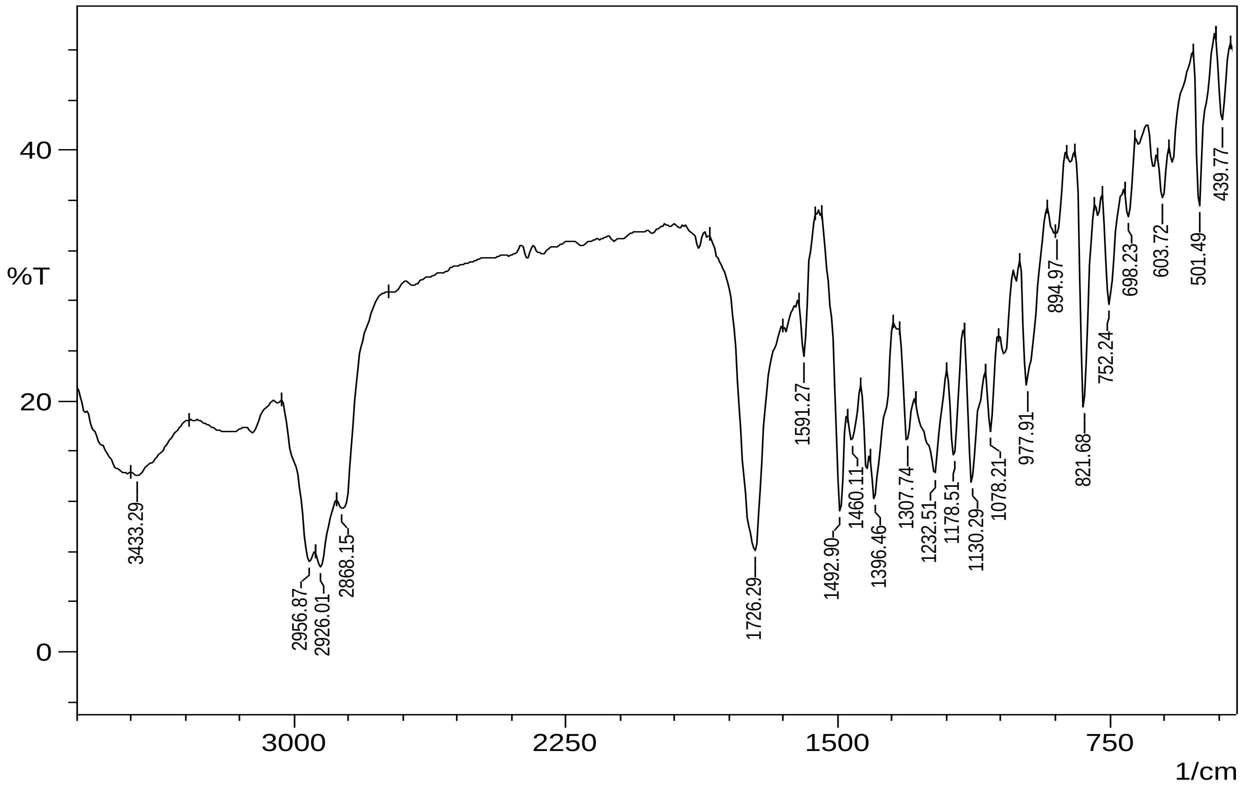

Figure S113. FTIR spectrum of the target compound (**4n**)

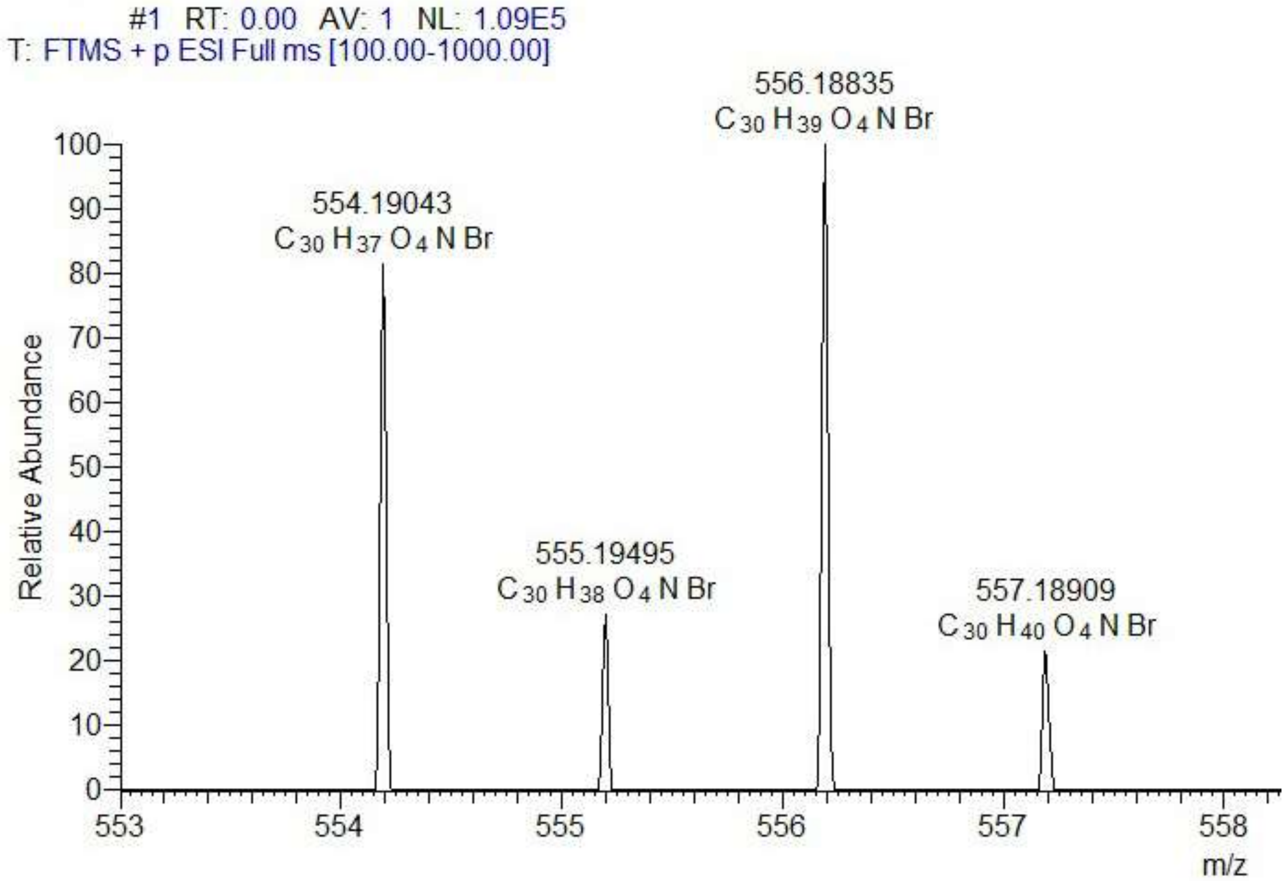

Figure S114. HRMS-ESI spectrum of the target compound (**4n**)

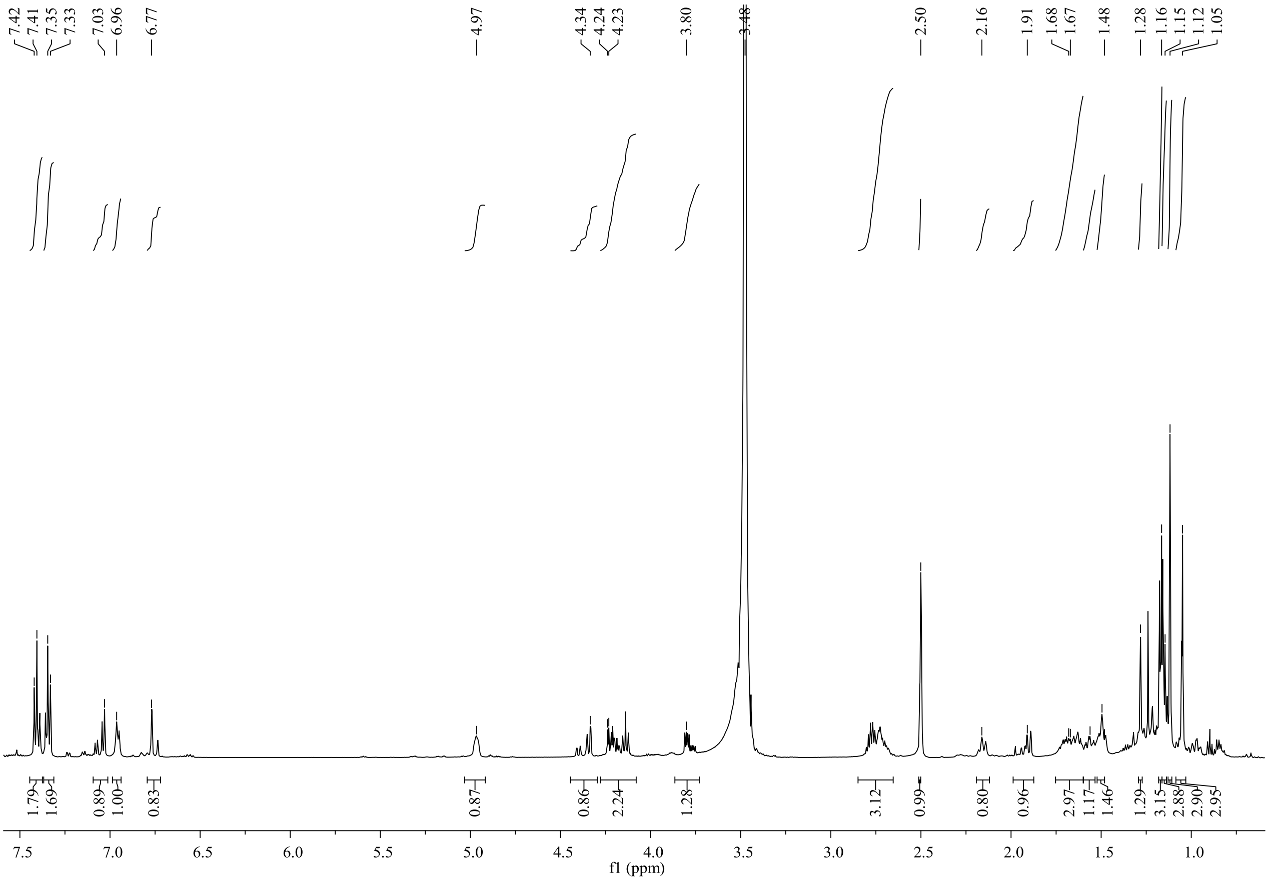

Figure S115. ^{1}H-NMR spectrum of the target compound (**4n**) in DMSO

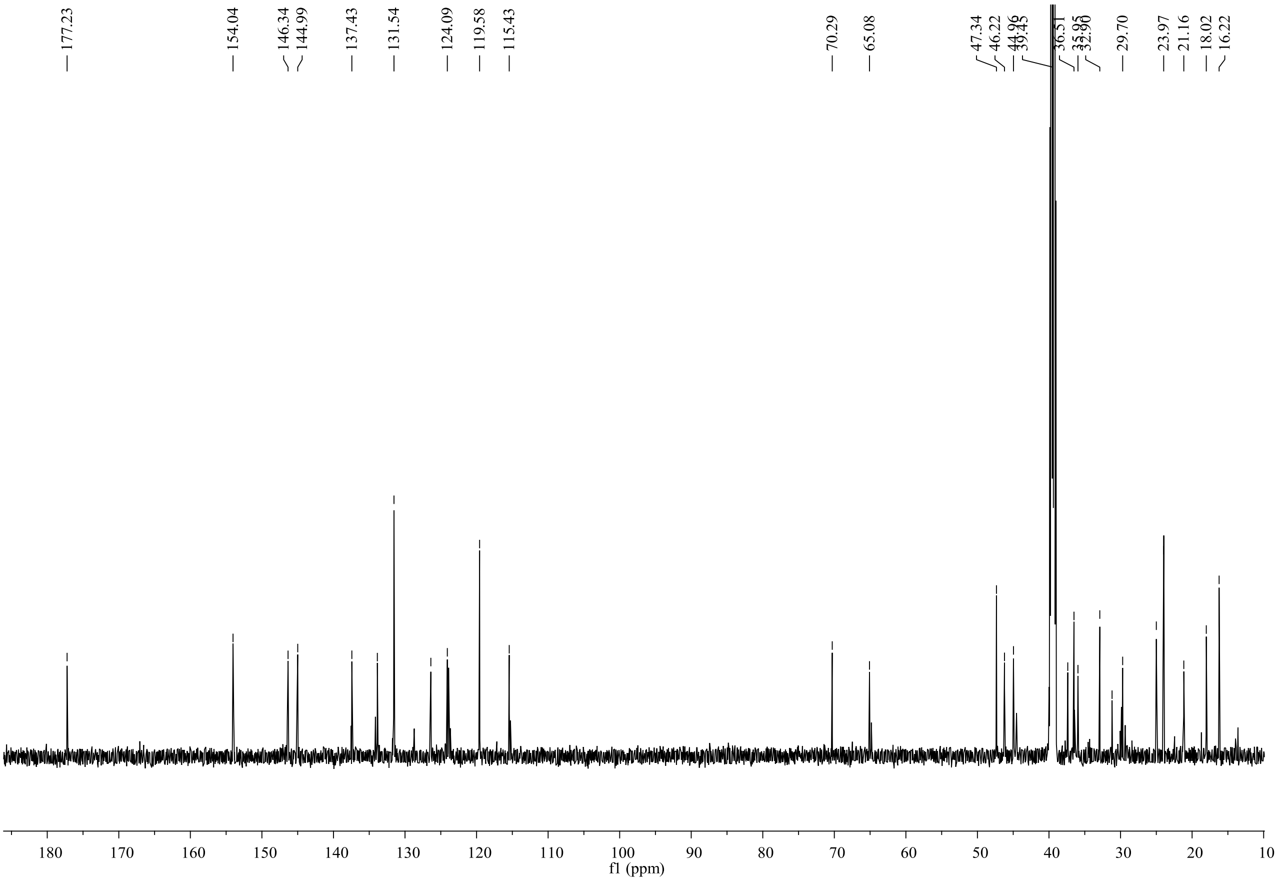

Figure S116. ^{13}C-NMR spectrum of the target compound (**4n**) in DMSO

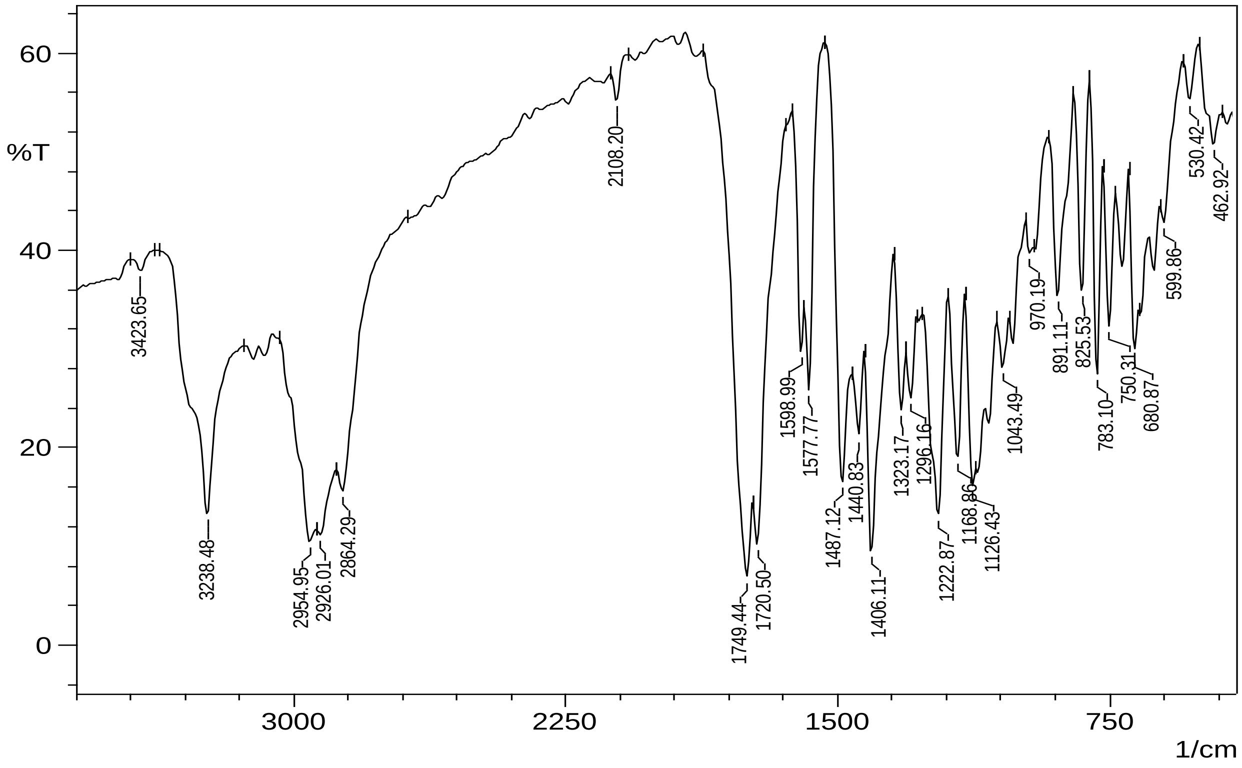

Figure S117. FTIR spectrum of the target compound (**4o**)

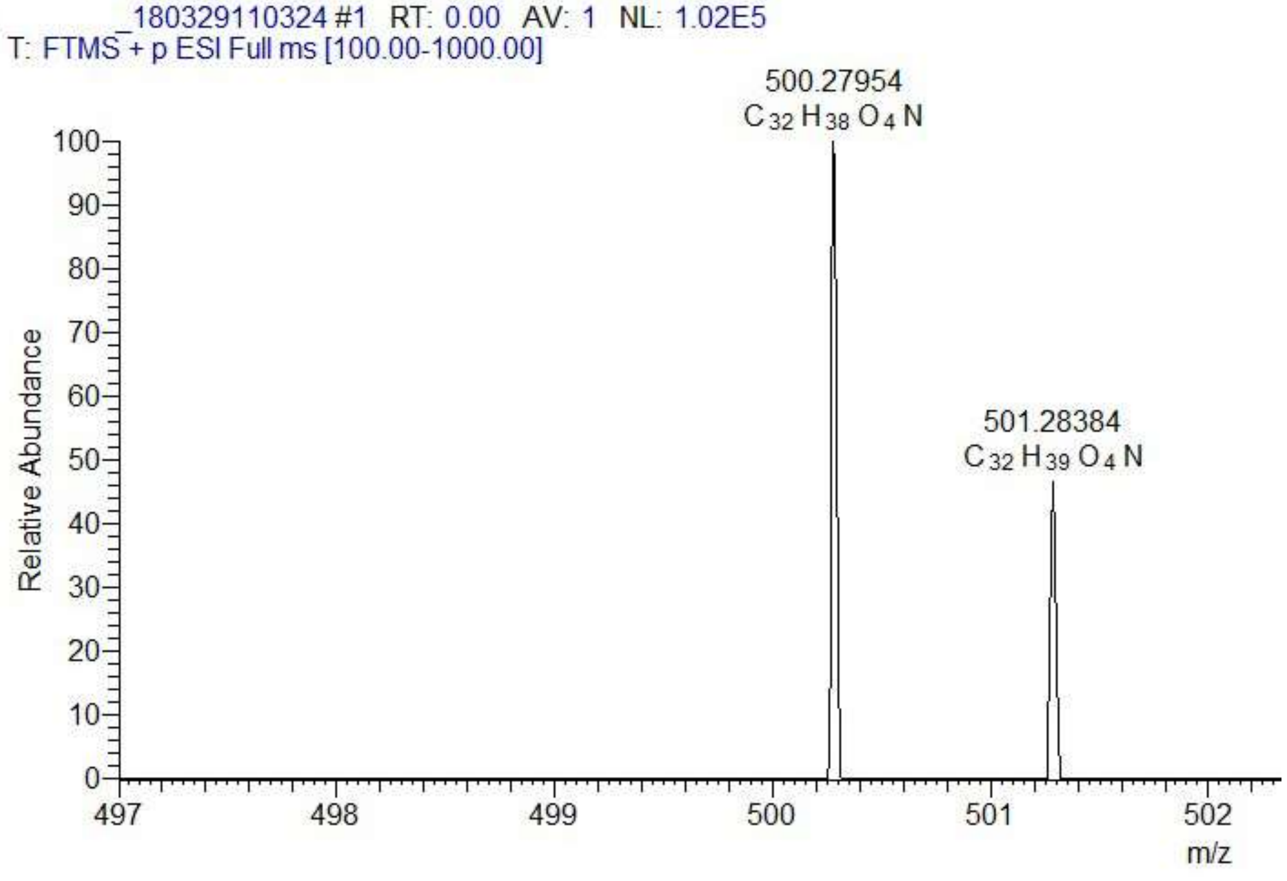

Figure S118. HRMS-ESI spectrum of the target compound (**4o**)

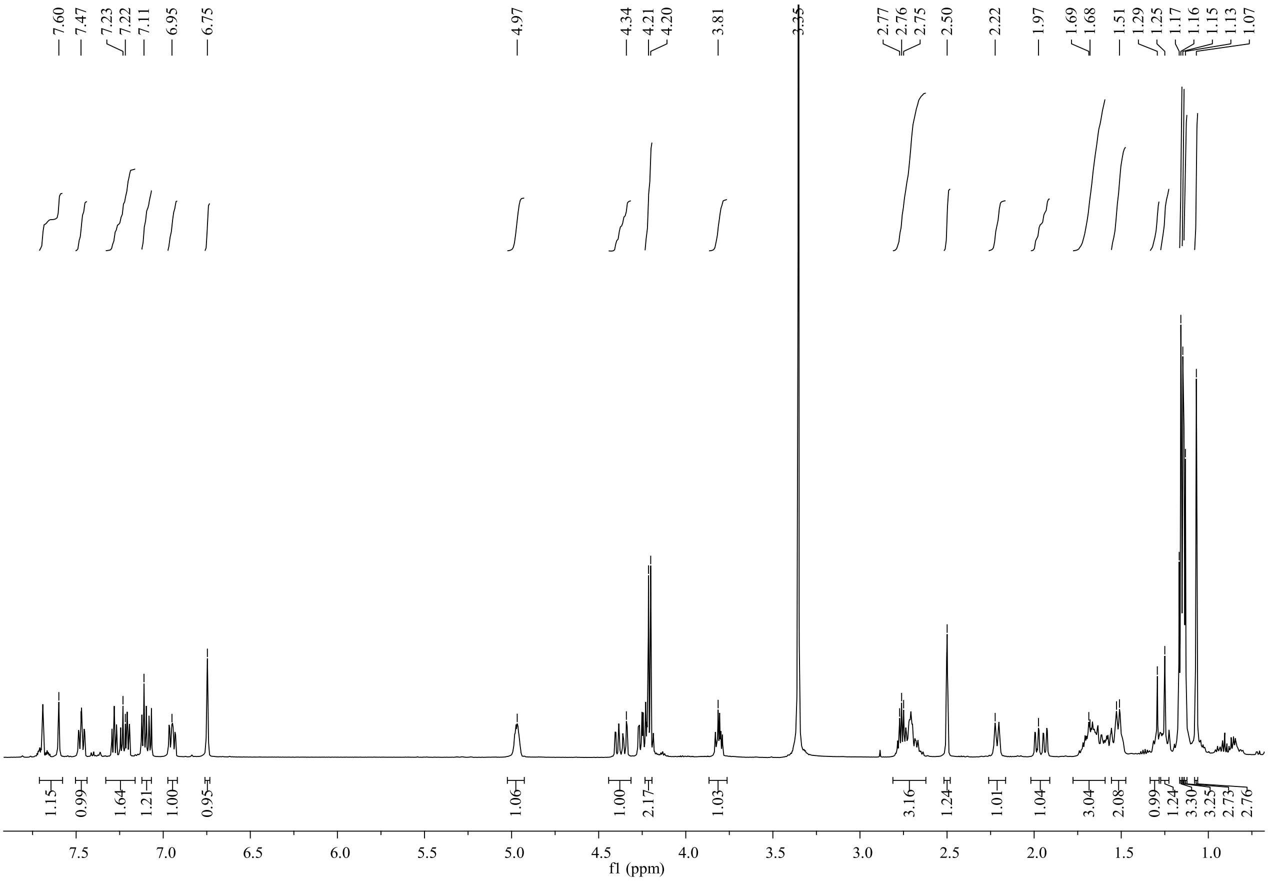

Figure S119. ^{1}H-NMR spectrum of the target compound (**4o**) in DMSO

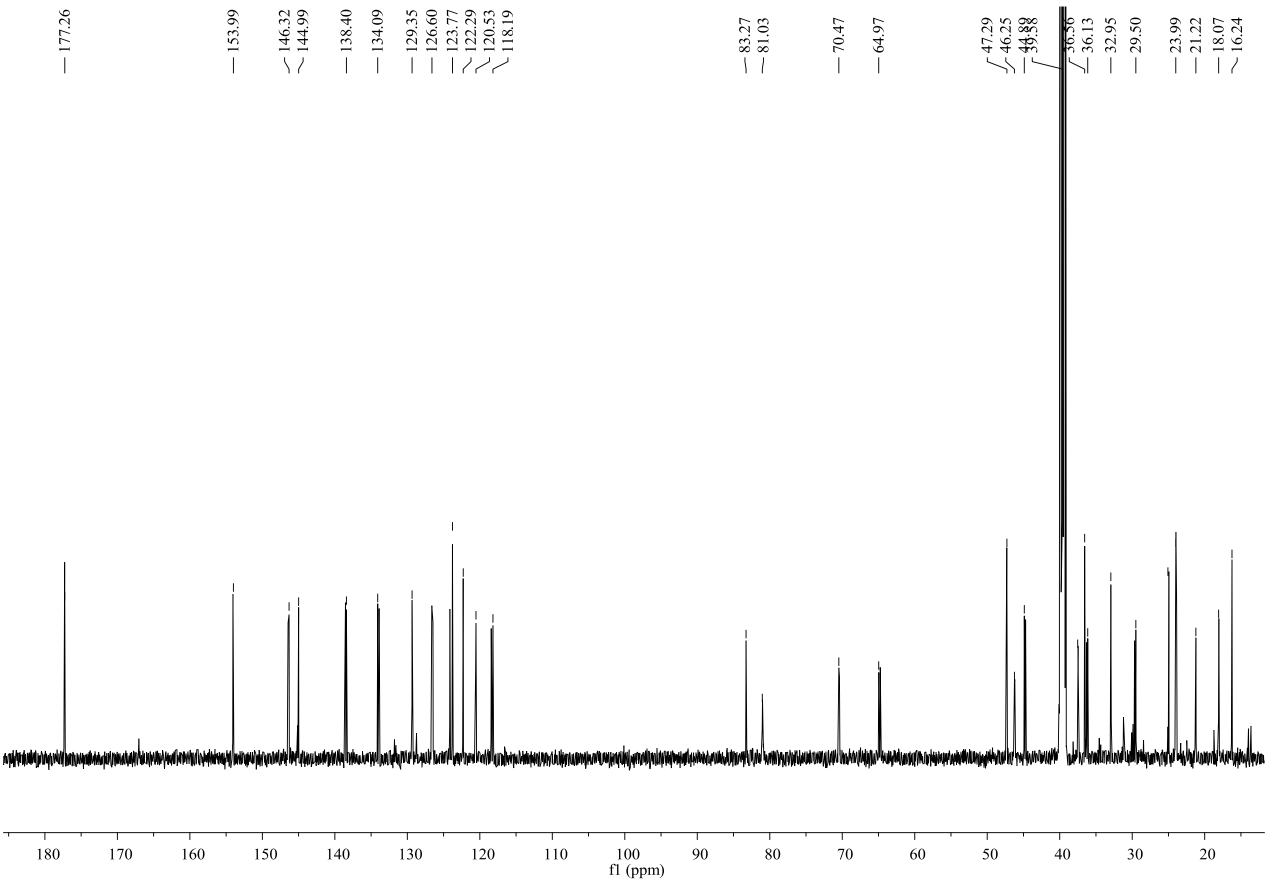

Figure S120. ^{13}C-NMR spectrum of the target compound (**4o**) in DMSO

Int. J. Mol. Sci. 2018, 19, 3116; doi:10.3390/ijms19103116
